# Discovery of a Novel and Potent Kir4.1 Inhibitor as a Safe and Rapid‐Onset Antidepressant Agent in Mice

**DOI:** 10.1002/advs.202509506

**Published:** 2025-12-03

**Authors:** Sisi Wang, Xiaoyu Zhou, Mengdan Li, Chao Zhang, Haiyan Xu, Jingyi He, Li Zhan, Yueling Gu, Hao Gu, Tianyu Tu, Hanfang Liu, Taotao Lu, Yueming Zheng, Jian Li, Zhaobing Gao, Yixiang Xu

**Affiliations:** ^1^ State Key Laboratory of Bioreactor Engineering Shanghai Frontiers Science Center of Optogenetic Techniques for Cell Metabolism Frontiers Science Center for Materiobiology and Dynamic Chemistry Shanghai Key Laboratory of New Drug Design School of Pharmacy East China University of Science and Technology Shanghai 200237 China; ^2^ State Key Laboratory of Drug Research Shanghai Institute of Materia Medica Chinese Academy of Sciences Shanghai 201203 China; ^3^ School of Pharmacy Henan University Kaifeng 475004 China; ^4^ Key Laboratory of Tropical Biological Resources of Ministry of Education School of Pharmaceutical Sciences Hainan University Haikou 570228 China; ^5^ Key Laboratory of Xinjiang Phytomedicine Resource and Utilization Ministry of Education School of Pharmacy Shihezi University Shihezi 832003 China; ^6^ Zhongshan Institute for Drug Discovery Shanghai Institute of Materia Medica Chinese Academy of Sciences Zhongshan 528437 China

**Keywords:** depression, Kir4.1 inhibitors, rapid‐onset, (*S*)‐ketamine, structural repurposing

## Abstract

Major depressive disorder is a serious psychiatric disorder for which novel and fast‐acting antidepressants are required. Targeted inhibition of the astrocytic inwardly rectifying potassium channel 4.1 (Kir4.1) in the lateral habenula could rapidly alleviate depression‐like behaviors. A previous study identified Kir4.1 as a promising target for achieving rapid‐onset antidepressant effects. The aim of this study is to identify novel Kir4.1 inhibitors with good druggability through structural modification of the lead compound EHop‐016, resulting in fifty derivatives. Among these, **JX3212** exhibits the most potent in vitro inhibitory activity against Kir4.1, with acceptable selectivity and excellent brain exposure. Notably, a single administration of **JX3212** results in rapid‐onset antidepressant effects within 1 h in multiple rodent models of depression, with comparable efficacy to (*S*)‐ketamine; this inhibitor‐like effect is abolished in mice with tamoxifen‐induced conditional Kir4.1 knockout in astrocytes. Additionally, **JX3212** demonstrates superior safety margins compared to both (*S*)‐ketamine and the conventional antidepressant imipramine in murine behavioral assays. In summary, **JX3212** functions as a selective Kir4.1 inhibitor with favorable druggability and stable antidepressant efficacy in preclinical models. This pharmacological profile supports the further development of **JX3212** as a promising therapeutic candidate for major depressive disorder.

## Graphical abstract

The preferred derivative **JX3212** demonstrates strong inhibitory activity against Kir4.1 with favorable druggability and shows significant antidepressant efficacy in vivo.

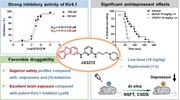

## 1 Introduction

Major depressive disorder, a common form of depression, manifests as a persistently low mood, loss of interest, fatigue, feelings of worthlessness, and recurrent suicidal ideation. This complex psychiatric disorder places a substantial burden on families and society owing to its high rates of prevalence, recurrence, disability, and suicide.^[^ ^1^, ^2^ ^]^

Most existing antidepressant medications were developed based on the monoamine hypothesis, which aims to increase neurotransmitter levels in the brain and elicit antidepressant effects. Despite their widespread use, traditional antidepressants often demonstrate a delayed onset of action (2–4 weeks), modest response rates (< 70% remission), and adverse effects, such as sexual dysfunction and weight gain, which complicate treatment for patients, particularly those with treatment‐resistant depression.^[^ ^3^, ^4^ ^]^ (*S*)‐ketamine, an antagonist of the glutamate *N*‐methyl‐*D*‐aspartate (NMDA) receptor, represents a recent therapeutic breakthrough in the field of depression because of its rapid‐onset characteristics, prompting further exploration of rapid‐acting drug interventions.^[^ ^5^ ^]^ Rapid‐onset antidepressant therapies not only shorten the duration of acute‐phase treatment but, more critically, reduce the persistent burden of depressive symptoms. This acceleration mitigates adverse psychosocial impacts on patients and their families and alleviates unnecessary healthcare expenditures. Furthermore, such interventions may confer life‐saving benefits by preventing suicides among vulnerable populations. However, the addictive and dissociative properties of (*S*)‐ketamine render it unsuitable for routine clinical use and raise concerns about its long‐term efficacy and safety.^[^ ^6^ ^]^ These unmet medical needs underscore the imperative to identify novel molecular targets that are capable of mediating rapid antidepressant responses with improved safety profiles.

The inwardly rectifying potassium channel 4.1 (Kir4.1, *Kcnj10*‐encoded) is exclusively expressed in the kidney and glial cells of the brain.^[^ ^7^, ^8^, ^9^ ^]^ As the principal potassium channel in astrocytes, Kir4.1 maintains extracellular K^+^ gradients through spatial buffering to regulate neuronal resting membrane potentials^[^ ^10^ ^]^ Owing to its capacity for spatial potassium buffering, Kir4.1 is linked to a range of neurological disorders, including epilepsy, amyotrophic lateral sclerosis, and Huntington's disease, suggesting its potential as a therapeutic target for certain conditions.^[^ ^11^ ^]^ Moreover, Kir4.1 dysfunction in the lateral habenula is associated with the onset and progression of depression. That is, Kir4.1 enhances the extracellular clearance of K^+^, leading to neuronal hyperpolarization and the subsequent activation of T‐type voltage‐sensitive calcium channels; this activation induces burst firing within neuronal clusters, amplifies electrical signaling, and increases the inhibition of downstream reward centers in the brain, contributing to the emergence of depressive symptoms.^[^ ^12^, ^13^ ^]^ Consequently, the development of Kir4.1 inhibitors aimed at downregulating the abnormal expression of Kir4.1 channels may offer new opportunities for the clinical treatment of depression.

Current Kir4.1 inhibitors remain limited to the repurposed agents, including aminoglycoside antibiotics, selective serotonin reuptake inhibitors, and antimalarial drugs, all of which demonstrate suboptimal target engagement and inadequate selectivity.^[^ ^14^, ^15^ ^]^ In a previous study, we presented compelling evidence that Kir4.1 may serve as a rapid‐acting and safe target for antidepressant medication.^[^ ^16^ ^]^ Specifically, the Kir4.1 inhibitor Lys05 showed a rapid‐acting antidepressant effect comparable to that of (*S*)‐ketamine. However, Lys05 is characterized by relatively low brain exposure and suboptimal drug properties, which may hinder its direct application in the treatment of depression.^[^ ^16^ ^]^ Specifically, intraperitoneal administration of Lys05 at 10 mg kg^−1^ resulted in a brain/plasma concentration ratio of merely 0.19 at 1‐h post‐injection, demonstrating exceptionally poor brain penetrability.^[^ ^16^ ^]^ Furthermore, Lys05 can induce Paneth cell dysfunction and manifest the intestinal phenotype of genetic autophagy deficiency, indicating certain safety concerns regarding the administration of this compound structure.^[^ ^17^ ^]^ These findings emphasize the need to develop potent and specific Kir4.1 inhibitors with good druggability, not only for further target validation but also for effective drug development.

In this study, we developed Kir4.1‐preferring inhibitors from the hit, EHop‐016, with a half‐maximal inhibitory concentration (IC_50_) value of 0.74 µm. EHop‐016, characterized by the chemical structure *N* ^4^‐(9‐ethyl‐9*H*‐carbazole‐3‐yl)‐*N* ^2^‐(3‐morpholinopropyl)pyrimidine‐2,4‐diamine, is a novel and specific inhibitor of Rac GTPase, exerting antitumor effects by inhibiting Rac1 activity.^[^ ^18^, ^19^ ^]^ Structural optimization of the carbazole‐pyrimidine scaffold yielded derivative **JX3212**, which exhibited the most favorable activity (IC_50_ = 0.28 µm) against Kir4.1, acceptable selectivity over several Kir isoforms and antidepressant targets in vitro, and excellent brain exposure (brain/plasma ratio = 10.72, blood–brain partitioning coefficient = 1.02). In well‐accepted depression models, including the novelty‐suppressed feeding test (NSFT) and chronic social defeat stress (CSDS) models, we observed that a single dose of **JX3212** produced rapid‐onset antidepressant effects 1 h after administration, with efficacy comparable to that of (*S*)‐ketamine. NSFT results in Kir4.1 conditional knockout mice indicated that **JX3212** exerts its antidepressant effects through Kir4.1. Critically, **JX3212** demonstrated superior safety margins compared to both the novel rapid‐acting antidepressant (*S*)‐ketamine and the conventional antidepressant imipramine in murine behavioral assays, particularly in the domains of cognition, motor function, and psychotomimetic‐like side effects. Collectively, the broad‐spectrum antidepressant effects, high brain exposure, and favorable safety profiles support the further development of **JX3212** as a promising candidate compound for treating depression.

## 2 Results

### 2.1 Discovery and Structural Optimization of Kir4.1 Inhibitor EHop‐016

**Figure 1:**
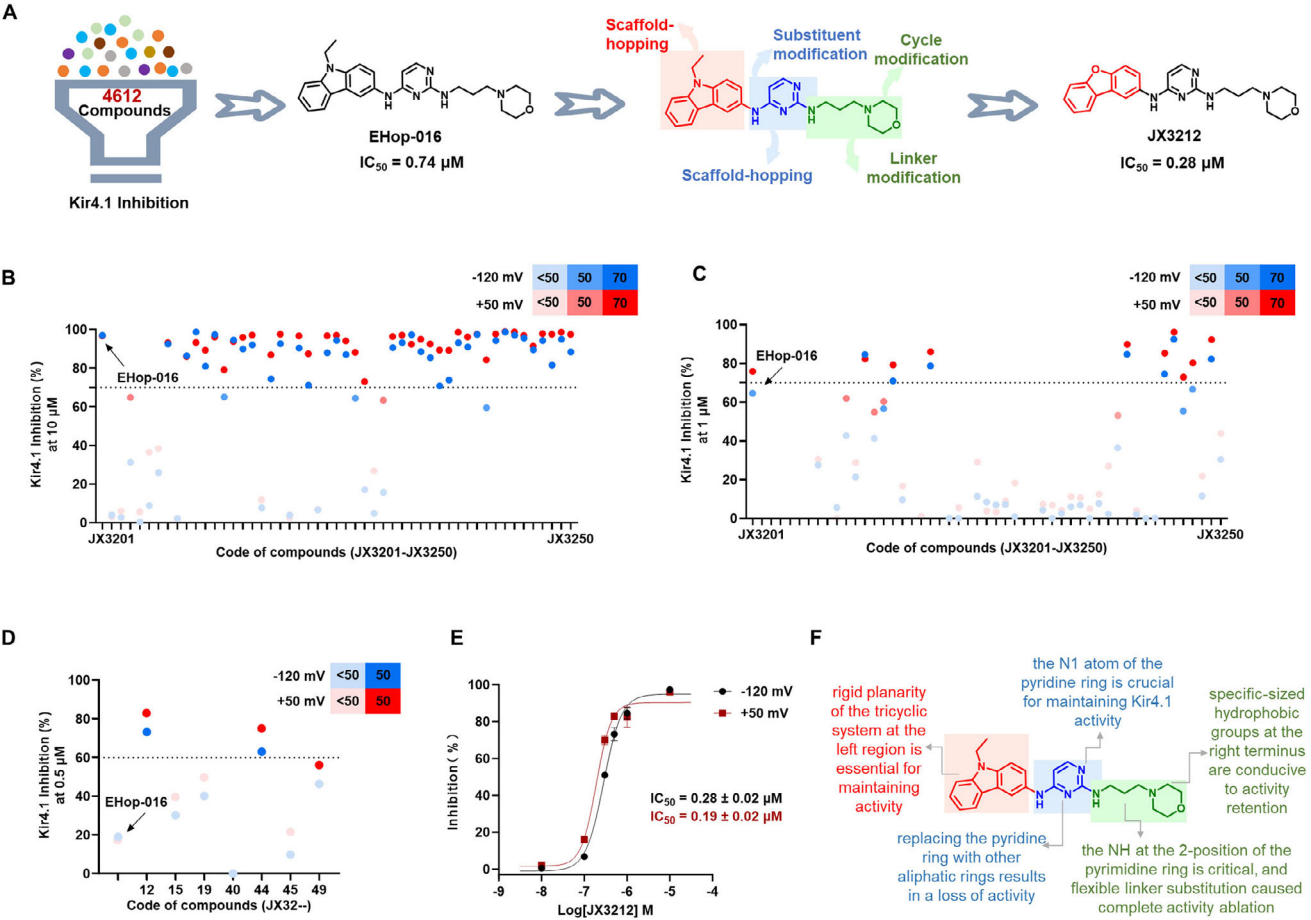
Structure‐guided optimization of EHop‐016 yields **JX3212** as a potent Kir4.1 inhibitor. A) Discovery and structural evolution of the Kir4.1 inhibitor EHop‐016 yields the active compound **JX3212**. B–D) Kir4.1 inhibitory activity of all derivatives at 10, 1, and 0.5 µm, respectively. E) Kir4.1 inhibitory IC_50_ values of **JX3212** (*n* = 3). F) Structure–activity relationship analysis. Data are shown as mean ± standard error of the mean (SEM).

Considering the substantial potential of Kir4.1 channels as therapeutic targets for depression, the aim of this study was to develop potent small‐molecule Kir4.1 inhibitors to expand the reservoir of potential rapid‐onset antidepressants. Previously, we established a thallium flux‐based high‐throughput screening platform to identify Kir4.1 inhibitors among 4612 chemically diverse compounds from an in‐house library, which included approved drugs and investigational agents at various developmental stages.^[^ ^16^ ^]^ EHop‐016, as a novel specific Rac GTPase inhibitor currently in preclinical development for breast cancer treatment, is selected as the lead compound without PAINS alerts after electrophysiological validation (**Figure** **1**A).^[^ ^18^, ^19^ ^]^ Whole‐cell patch‐clamp analysis demonstrated that EHop‐016 displayed favorable activities for channel inhibition in Chinese Hamster Ovary (CHO) cells transiently expressing Kir4.1, with IC_50_ values of 0.74 µm (−120 mV, inward currents) and 0.61 µm (+50 mV, outward currents) (Figure S1, Supporting Information).

To enhance the target‐specific inhibitory potency, systematic structural modifications were conducted on the EHop‐016 scaffold (Figure 1A). Initially, we performed skeletal rearrangement of the carbazole moiety at the left terminus, allowing its conversion into various monocyclic, bicyclic, and tricyclic configurations, yielding compounds **JX3201**–**JX3215**. Following the successful establishment of the advantageous dibenzofuran skeleton at the left terminus, we modified the central pyrimidine ring through both substitution and skeletal alterations to generate compounds **JX3216**–**JX3230**. After identifying the pyrimidine ring with the highest activity, we modified the linker and aliphatic ring segments at the right terminus to yield compounds **JX3231**–**JX3250**. The structures of all derivatives are illustrated in Figure S2 (Supporting Information).

### 2.2 Kir4.1 Inhibitory Profiling and Structure–Activity Relationships of Derivatives

Primary screening involved a systematic evaluation of Kir4.1 inhibitory potency across all derivatives at a concentration of 10 µm. Compounds demonstrating > 70% inhibition at this concentration underwent secondary pharmacological characterization through dose–response studies (1 and 0.5 µm). In general, 74% of derivatives (37 of 50) maintained > 70% inhibitory potency at 10 µm, whereas the proportion of active compounds decreased to 14% (7 of 50) at 1 µm, with only two analogs (**JX3212** and **JX3244**) retaining > 60% inhibition at 0.5 µm (Figure 1B–D). Among these, compound **JX3212** demonstrated enhanced potency with IC_50_ values of 0.28 µm (−120 mV) and 0.19 µm (+50 mV) (Figure 1E). Structural optimization of the lead compound EHop‐016 revealed **JX3212** as the optimal candidate, with a 2.6‐fold improvement in target activity.

The specific activity results for all derivatives are presented in Table S1 (Supporting Information). Based on the target activity evaluation, we summarized the structure–activity relationships of the derivatives as follows (Figure 1F). 1) Rigid planarity of the tricyclic system in the left region is essential for maintaining activity; modification of the carbazole ring to a monocyclic or bicyclic structure significantly reduces Kir4.1 inhibitory activity. 2) The N1 atom of the pyridine ring is crucial for maintaining Kir4.1 activity; replacing the pyridine ring with other aliphatic rings results in a loss of activity. 3) NH at the 2‐position of the pyrimidine ring, which serves as a hydrogen bond donor, is critical for target engagement and forms interactions with the Kir4.1 residue. 4) Flexible linker substitution caused complete activity ablation, whereas specific‐sized hydrophobic groups at the right terminus are conducive to activity retention, with piperidine and its analogs showing optimal activity. These structure–activity relationships are explicit and, to some extent, provide guidance for developing novel Kir4.1 inhibitors based on EHop‐016.

Notably, as the lead compound EHop‐016 exhibits intrinsic antitumor activity against MDA‐MB‐468 breast cancer cells (IC_50_ = 5.98 ± 0.67 µm), we systematically evaluated all synthesized derivatives in this cell line. Seven compounds displayed IC_50_ > 50 µM (Table S2, Supporting Information), indicating nearly complete loss of antitumor activities, whereas the optimized derivative **JX3212** retained partial antitumor activity (IC_50_ = 13.74 ± 0.61 µm). The ratio of the anti‐proliferation IC_50_ value in MDA‐MB‐468 cells to the inhibition IC_50_ value against Kir4.1 was used as the preliminary therapeutic index (TI) metric. The TI value of EHop‐016 was ≈8, whereas that of **JX3212** was ≈49. These data demonstrate that the antitumor effects of **JX3212** are significantly reduced compared with those of EHop‐016. Moreover, the effective concentration of **JX3212** for antidepressant‐related Kir4.1 inhibition lies below the antitumor concentration range, indicating a manageable off‐target risk for compound **JX3212**.

### 2.3 Target Selectivity of JX3212

**Figure 2:**
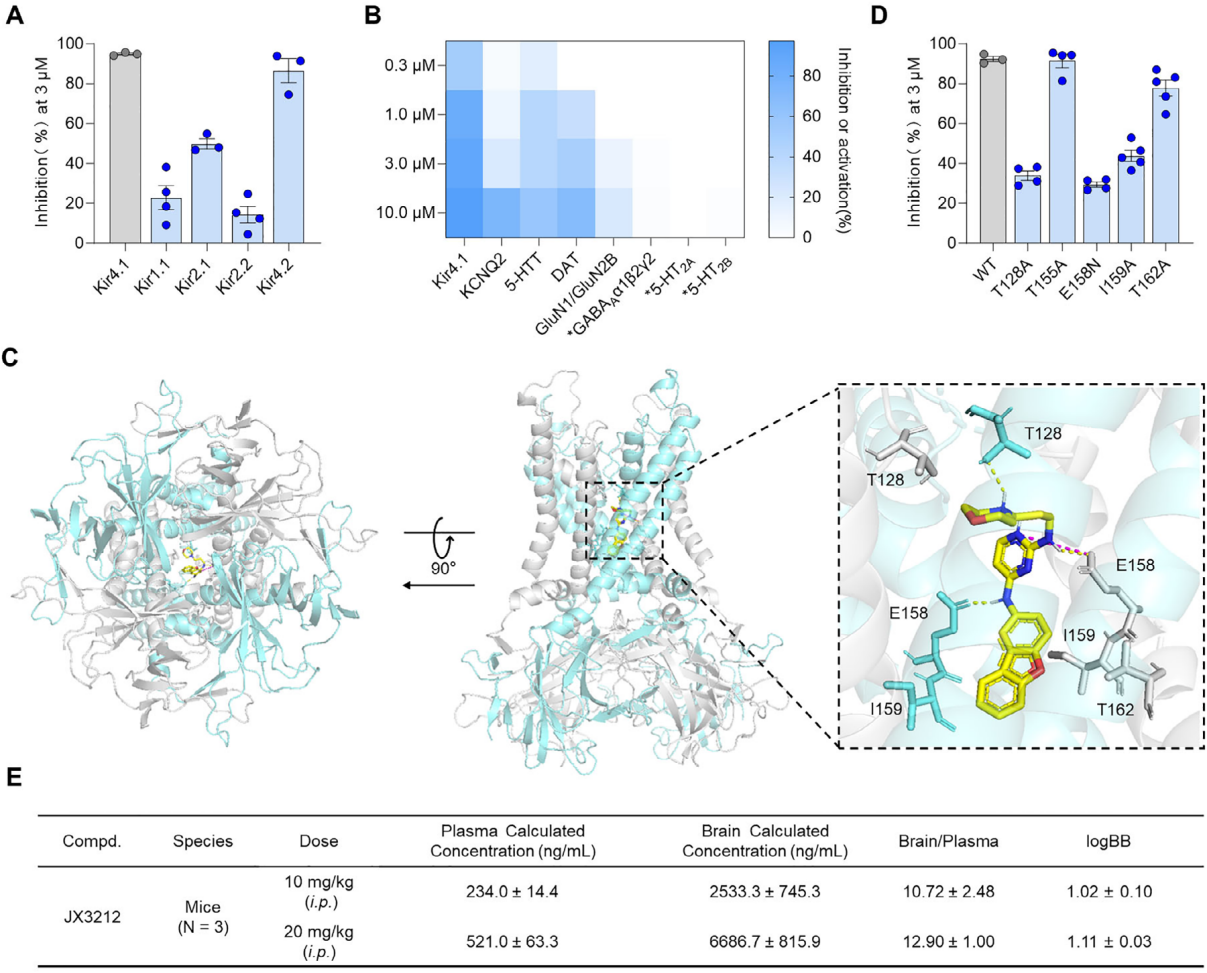
Target selectivity, binding mode, and brain penetration profiles of **JX3212**. A) **JX3212** inhibition of Kir isoforms (Kir4.1, Kir1.1, Kir2.1, Kir2.2 and Kir4.2) at 3 µm (*n* = 3–4). Data are shown as mean ± SEM. B) Heatmap showing effects of **JX3212** (0.3, 1.0, 3.0, and 10.0 µm) against a selected panel of channels, transporters, and receptors. The asterisks (*) indicate potentiation effects exerted at corresponding receptors. C) Top and side views of the predicted binding mode between Kir4.1 (PDB ID: 8I5M) and **JX3212**. D) Inhibition of Kir4.1 wild‐type (WT) and mutant channels measured at 3 µm (*n* = 3–5). Data are shown as mean ± SEM. E) Blood–brain penetration of **JX3212**. Data are shown as mean ± standard deviation.

To evaluate the target selectivity of the small‐molecule Kir4.1 inhibitor **JX3212**, we first tested its inhibitory activity against Kir channel subtypes (Kir1.1/2.1/2.2/4.2). Kir subtype specificity assessment at 3 µm revealed a selectivity profile of **JX3212**: minimal inhibition of Kir1.1 (17.78 ± 8.26%), Kir2.1 (49.90 ± 4.35%), and Kir2.2 (14.28 ± 8.36%) versus potent Kir4.2 blockade (86.57 ± 10.51%) (**Figure** **2**A). We next evaluated a panel of established antidepressant targets, including 5‐hydroxytryptamine transporter (5‐HTT), dopamine transporter (DAT), GABA_A_ receptors (GABA_A_α1β2γ2), 5‐HT_2A_/5‐HT_2B_ receptors, and NMDA receptor (GluN1/GluN2B) at concentrations of 0.3, 1, 3, and 10 µm (Figure 2B). The concentration–response curves and exemplar current traces for representative targets are provided in Figures S3 and S4 (Supporting Information). Compared to Kir4.1, **JX3212** exhibited weak or no effects on all examined targets (KCNQ2, IC_50_ = 5.99 µm, 5‐HTT, IC_50_ = 6.45 µm, DAT, IC_50_ = 5.20 µm, GABA_A_α1β2γ2, EC_50_ > 60 µm, 5‐HT_2A_/5‐HT_2B_, EC_50_ > 60 µm, GluN1/GluN2B, IC_50_ = 23.73 µm). Collectively, these data demonstrate that **JX3212** exhibits a certain degree of selectivity for Kir4.1.

### 2.4 Binding Mode of JX3212 with Kir4.1

To elucidate the binding mode of **JX3212**, molecular docking was performed using the Glide module (Schrödinger Suite) against the Kir4.1 protein structure (PDB ID: 8I5M). Subsequent analyses were conducted using PyMOL software. As illustrated in Figure 2C, the top‐down view (left) and side view (middle) of Kir4.1 depict the predicted positioning of **JX3212** within the transmembrane pore, whereas the close‐up view (right) highlights key interactions. The dibenzofuran moiety of **JX3212** formed hydrophobic interactions with I159, whereas its NH group, linked to the pyrimidine ring, acted as a hydrogen bond donor to form a hydrogen bond with E158. Additionally, the nitrogen atom at position 1 of the pyrimidine ring formed both a hydrogen bond and salt bridge with E158. Furthermore, the basic nitrogen center of the morpholine ring generated a strong hydrogen bond with T128. The docking results consistently identified the critical binding site atoms, confirming that the large planar moieties of dibenzofuran, pyrimidine ring, and morpholine ring are critical for maintaining biological activity, as supported by the structure–activity relationship analysis.

Furthermore, to evaluate the binding sites between the Kir4.1 protein and **JX3212**, we conducted a point mutation experiment on Kir4.1. Based on the predicted binding mode of **JX3212** to Kir4.1, and the reported binding modes of Kir4.1 selective inhibitors,^[^ ^16^ ^]^ we performed site‐directed point mutations on five selected amino acid residues. The inhibitory effects on both wild‐type (WT) and mutant forms were assessed using a whole‐cell patch clamp. Mutations at four Kir4.1 residues (T128A, E158N, I159A, and T162A) led to a reduction in the inhibitory effect of compound **JX3212** on Kir4.1 currents at 3 µm, most notably with T128A (33.92 ± 4.58% current inhibition vs WT 92.40 ± 2.25%), E158N (29.44 ± 2.66% current inhibition vs WT 92.40 ± 2.25%), and I159A (43.90 ± 6.15% current inhibition vs WT 92.40 ± 2.25%) mutations (Figure 2D). This residue‐specific functional mapping demonstrated that T128, E158, and I159 form critical interactions between **JX3212** pharmacophore and Kir4.1 amino acid residues, which confirms the reliability of the predicted binding mode of **JX3212** in Kir4.1, and establishes a structural basis for its channel‐modulating activity.

### 2.5 Brain Penetration of JX3212

Blood–brain barrier permeability is pivotal for determining the efficacy of central nervous system‐targeted antidepressants. Therefore, we assessed the brain penetration properties of **JX3212** in C57BL/6 mice following intraperitoneal administration at doses of 10 and 20 mg kg^−1^. The ability of a molecule to cross the blood–brain barrier is quantified by the blood–brain partitioning coefficient (logBB), which is the log10 ratio of the drug concentration in the brain tissue to that in the blood (logBB = log10[brain]/[blood]). Generally, compounds with logBB exceeding 0.3–0.5 are considered to possess adequate central nervous system permeability, and those with logBB > 1 are considered able to cross the blood–brain barrier freely.^[^ ^20^ ^]^ Pharmacokinetic analysis (Figure 2E) revealed that **JX3212** achieved superior brain partitioning: plasma concentration = 234.0 ± 14.4 ng mL^−1^ vs brain concentration = 2533.3 ± 745.3 ng mL^−1^ at 1 h post‐dosing with 10 mg kg^−1^, and plasma concentration = 521.3 ± 63.1 ng mL^−1^ vs brain concentration = 6,686.7 ± 815.9 ng mL^−1^ at 1 h post‐dosing with 20 mg kg^−1^, yielding a high total brain/plasma concentration ratio > 10 (logBB > 1) at both 10 and 20 mg kg^−1^. These results indicate favorable dose‐dependent brain exposure and stable penetration characteristics. Notably, **JX3212** exhibited a significantly higher brain/blood distribution ratio than the reported Kir4.1 inhibitor Lys05 (brain/plasma concentration ratio of 0.19 at 1 h post‐dosing with 10 mg kg^−1^).^[^ ^16^ ^]^ Consequently, compound **JX3212** exhibits a satisfactory central nervous system distribution with potential effects in the brain, warranting further in vivo pharmacodynamic evaluation.

### 2.6 Antidepressant Responses of JX3212 in Multiple Rodent Models

**Figure 3:**
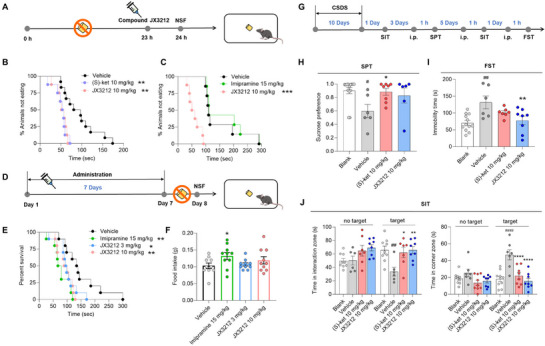
**JX3212** shows effective antidepressant activity in the novelty‐suppressed feeding test (NSFT) and chronic social defeat stress (CSDS) model. A) NSFT paradigm (single dose). B,C) The NSFT of **JX3212** and (*S*)‐ketamine (B) or imipramine (C) at a single dose (*n* = 8–12 and 7–8, respectively) determined by Kaplan–Meier survival analysis with Mantel–Cox test. D) NSFT paradigm (repeated doses). E) NSFT results for **JX3212** and imipramine at repeated doses (*n* = 10) determined by Kaplan–Meier survival analysis with Mantel–Cox test. F) Food intake in home cage (*n* = 10) determined by two‐tailed Student's *t*‐test. G) CSDS paradigm. H) Sucrose preference test (SPT) of mice after CSDS (*n* = 6–14) determined by one‐way analysis of variance (ANOVA) with Dunnett's test. I) Immobility time in the forced swim test (FST) of mice after CSDS (*n* = 6–12) determined by one‐way ANOVA with Dunnett's test. J) Social interaction test (SIT) of mice (*n* = 6–10) determined by two‐way ANOVA with Bonferroni's test. Data are shown as mean ± SEM. ^#^ *p* < 0.05, ^##^ *p* < 0.01, ^####^ *p* < 0.0001 vs. corresponding blank group. ^*^ *p* < 0.05, ^**^ *p* < 0.01, ^***^ *p* < 0.001, ^****^ *p* < 0.0001 vs. corresponding vehicle group.

To investigate the therapeutic potential of Kir4.1, we systematically evaluated the antidepressant effects of compound **JX3212** across various depression models. Initially, we employed the NSFT, which is used to study anxiety‐ and depression‐related behaviors.^[^ ^21^ ^]^ Notably, (*S*)‐ketamine has been reported to exhibit rapid antidepressant effects in this acute model.^[^ ^22^ ^]^ Our results demonstrated that a single dose of **JX3212** (10 mg kg^−1^) or, (*S*)‐ketamine (10 mg kg^−1^), significantly reduced the latency to feed at 1 h following intraperitoneal administration (**Figure** **3**B), indicating a rapid onset of antidepressant action. Additionally, the traditional antidepressant imipramine (15 mg kg^−1^) failed to produce antidepressant‐like effects after a single administration (Figure 3C). Following repeated administration for seven days, both imipramine (15 mg kg^−1^) and **JX3212** (3/10 mg kg^−1^) elicited significant antidepressant‐like responses (Figure 3E). Food intake in the **JX3212**‐treated group remained comparable to that in the vehicle group, effectively excluding appetite change as a confounding factor for the observed antidepressant effects. In contrast, imipramine administration induced a significant increase in food intake, which was likely attributable to its appetite‐stimulating side effects (Figure 3F).^[^ ^23^ ^]^ After confirming the rapid antidepressant efficacy of **JX3212**, we further investigated the dose–response study of **JX3212** at doses of 1, 3, and 10 mg kg^−1^ in NSFT. The results revealed a dose‐dependent antidepressant‐like phenotype, with 10 mg kg^−1^ identified as the effective threshold dose for this compound following acute administration (Figure S5A, Supporting Information). In the acute forced swim test (FST), **JX3212** consistently demonstrated dose‐dependent antidepressant effects, significantly reducing immobility time at both doses (30 and 60 mg kg^−1^) (Figure S5B, Supporting Information).

The rapid‐onset antidepressant activity of **JX3212** was further validated using the CSDS model, a well‐established paradigm that effectively simulates the impact of social factors on depression onset.^[^ ^24^ ^]^ To evaluate depression‐like behaviors, we used the FST and sucrose preference test (SPT) to quantify behavioral despair and anhedonia, respectively. Additionally, social avoidance outcomes from the CSDS model were analyzed by comparing the total interaction zone duration of C57BL/6 mice during social interaction test (SIT) sessions with and without the target. As expected, mice susceptible to the 10‐day paradigm of social defeat stress exhibited a reduced preference for sucrose solution and an increased duration of immobility compared to the blank group. In the SIT, the mice spent less time in the interaction zone and more time in the corners. Notably, these pathological behavioral changes were reversed following a single injection (intraperitoneal (*i.p*.), 10 mg kg^−1^) of **JX3212** or (*S*)‐ketamine. Specifically, after **JX3212** administration (10 mg kg^−1^), we observed a nonsignificant increase in the sucrose preference ratio of SPT, reduced immobility duration in the FST, and a significant decrease in the time spent in corners during the SIT. In a parallel experiment, (*S*)‐ketamine (*i.p*., 10 mg kg^−1^) resulted in increased sucrose preference in the SPT and a shorter duration spent in the corners, but ad no significant impact on the FST results (Figure 3H–J). Hence, **JX3212** effectively relieved core symptoms in CSDS depression models.

Furthermore, we employed the olfactory bulbectomy (OBX) model, a robust preclinical system that recapitulates depression pathologies and exhibits predictive validity for antidepressant action timing, to evaluate the in vivo antidepressant efficacy of **JX3212**.^[^ ^25^, ^26^ ^]^ Following a 14‐day post‐surgery recovery period, C57BL/6J mice were assessed for hallmark phenotypes using the open field test (Figure S6, Supporting Information). Consistent with previous studies,^[^ ^27^, ^28^ ^]^ OBX‐operated mice displayed hyperlocomotor activity, as demonstrated by a significant increase in total distance traveled, and a reduction in the percentage of distance traveled in the center compared to sham‐operated mice, indicative of depressive‐like and anxiety‐like behaviors, respectively. Treatment with **JX3212** (30 mg kg^−1^) did not affect the distance traveled by OBX mice but significantly alleviated anxiety‐like behavior within 1 h, as evidenced by the increased percentage of distance traveled in the center.

### 2.7 JX3212 Acts on Astrocytic Kir4.1 to Exert Rapid‐Onset Antidepressant Effects

**Figure 4:**
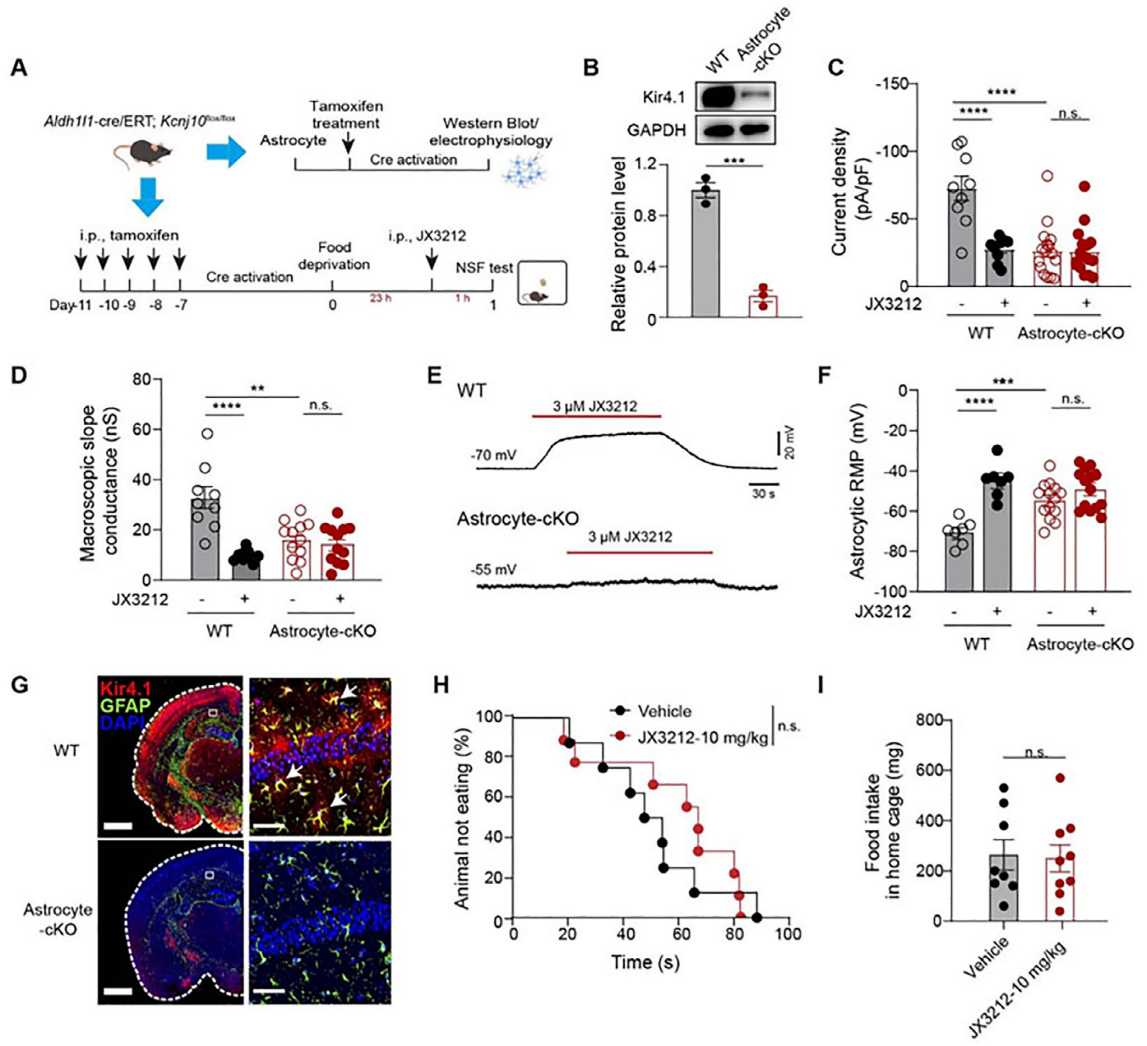
Verification of the **JX3212** mechanism. A) Schematic showing the design of the mechanism verification experiment with Kir4.1 cKO mice. B) Immunoblots (top) and quantification (bottom) showing Kir4.1 protein levels from primary cortical astrocytes of WT and Kir4.1 cKO mice (*n*  =  3 biological samples), determined by two‐tailed Student's *t*‐test. C) Current density versus voltage relationships for control and Kir4.1 cKO astrocytes in the absence or presence of 3 µm **JX3212** (*n* = 9–16), determined by two‐tailed Student's *t*‐test. D) Comparison of the effects of 3 µm **JX3212** on macroscopic slope conductance between control and Kir4.1 cKO astrocytes (*n* = 9–12), determined by two‐tailed Student's *t*‐test. E) Representative resting membrane potential traces of WT and Kir4.1 cKO astrocytes. F) Effects of **JX3212** on astrocytic resting membrane potentials (*n* = 7–14), determined by two‐tailed Student's *t*‐test. G) Representative immunostaining in WT and Kir4.1 cKO brain with the right as corresponding zoom‐in images of the white square area in left (scale bar, left 1000 mm, right 50 µm). The white arrows indicate Kir4.1‐labelled astrocytes. H) NSFT (*n* = 8–9), determined by Kaplan–Meier survival analysis with Mantel–Cox test. I) Food intake in home cage (*n* = 8–9) determined by two‐tailed Student's *t*‐test. Data are shown as mean ± SEM. n.s. *p* > 0.05, ^**^ *p* < 0.01, ^***^ *p* < 0.001, ^****^ *p* < 0.0001.

Astrocytic Kir4.1, which is largely responsible for buffering K^+^ in the brain, is implicated in the pathophysiology of depression. We hypothesized that astrocyte‐encoding Kir4.1 may mediate the therapeutic effects of systemic **JX3212** administration. Thus, we generated transgenic mice with conditional knockout of astrocyte‐specific *Kcnj10* (Kir4.1 cKO) by a crossing mouse line expressing the conditional floxed allele of *Kcnj10* (*Kcnj10* ^flow/flow^) with the inducible Cre line *Aldh1l1*‐Cre/ERT2 (**Figure** **4**A). Western blot analysis and immunostaining revealed a significant reduction in Kir4.1 protein in astrocytes from *Aldh1l1*‐Cre/ERT2*; Kcnj10* ^flow/flow^ mice, confirming effective Kir4.1 deficiency (Figure 4B,G). Additionally, astrocytes from Kir4.1 cKO mice exhibited significantly decreased potassium currents (Figure 4C), impaired macroscopic slope conductance (Figure 4D), and depolarized resting membrane potentials (Figure 4F) compared with their vehicle‐treated counterparts. Consistent with Kir4.1 inhibition, **JX3212** treatment notably decreased potassium currents in astrocytes and partially impaired the macroscopic slope conductance (Figure 4C,D). **JX3212** also caused marked depolarization at a concentration of 3 µm, as did 100 µm BaCl_2_, a selective Kir channel blocker in the sub‐millimolar range (Figure 4E; Figure S7, Supporting Information). In contrast, the effects induced by **JX3212** were abolished in tamoxifen‐treated Kir4.1 cKO astrocytes (Figure 4C–F). These results suggest that **JX3212** modulates native Kir4.1 channels in a specific manner. Kir4.1 cKO mice were subjected to NSFT after intraperitoneal injection of the vehicle or **JX3212**. Notably, astrocyte‐specific Kir4.1 cKO mice maintained comparable latencies between **JX3212**‐treated and vehicle‐treated groups at 10 mg kg^−1^, which is the dose that produced rapid‐onset antidepressant effects in WT mice (Figure 4H,I). These findings collectively demonstrate that the antidepressant effect of **JX3212** requires intact Kir4.1, establishing astrocytic Kir4.1 as the mechanism of action.

### 2.8 Multidimensional Safety Profiling of JX3212

Kir4.1 channels exhibit region‐specific expression patterns across brain areas, where they modulate neuronal excitability and network activity by regulating astrocytic potassium buffering. Spatial heterogeneity is functionally linked to emotional regulation, seizure susceptibility, and cognitive processing.^[^ ^29^, ^30^ ^]^ To address the regional expression profile of Kir4.1, we performed an immunofluorescence analysis across multiple brain regions, including the hippocampus, habenula, isocortex, etc. Kir4.1 is widely expressed throughout multiple regions (Figure S8, Supporting Information), suggesting that **JX3212** acts on Kir4.1 channels in a broad manner once it crosses the blood–brain barrier. Consequently, potential neurological side effects arising from the pharmacological inhibition of Kir4.1 by **JX3212** must be assessed.

**Figure 5:**
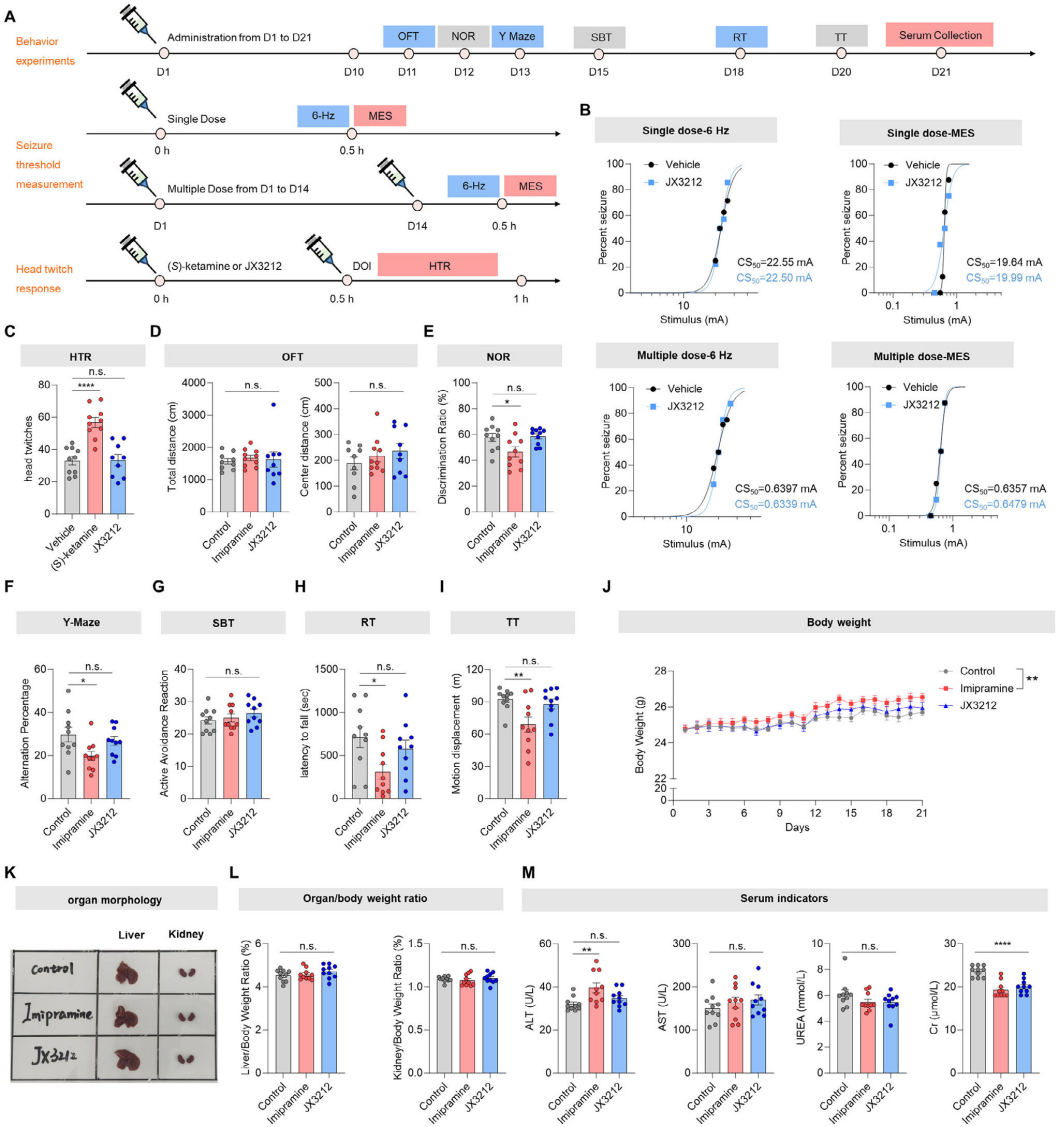
Safety study of **JX3212**. A) Outline of behavior experiments, seizure threshold measurement, and head‐twitch response. B) Seizure susceptibility of mice treated with a single injection and multiple dose (10 mg kg^−1^) of **JX3212** in the 6‐Hz seizure model and maximal electronic seizure (MES) model (*n* = 4–5). C) Head‐twitch response (HTR, *n* = 9–10). D) Open field test (OFT, *n* = 9–10). E) Novel object recognition (NOR, *n* = 10). F) Y‐Maze (*n* = 10). G) Shuttle box test (SBT, *n* = 10). H) Rotarod test (RT, *n* = 10). I) Treadmill test (TT, *n* = 10). J) Daily weight of all groups (*n* = 10). K) Liver and kidney organ morphology for each group after drug administration. L) Weight percentage of critical organs for all groups (*n* = 10). M) Biochemical parameters of liver and renal profiles (*n* = 10). AST, aspartate aminotransferase; ALT, alanine aminotransferase; Cr, creatinine. Two‐tailed Student's *t*‐test. Data are shown as mean ± SEM. n.s. *p* > 0.05, ^*^ *p* < 0.05, ^**^ *p* < 0.01, ^****^ *p* < 0.0001 vs. control.

First, a single or 14‐day consecutive administration of **JX3212** (10 mg kg^−1^) produced no seizure‐like effects in the maximal electronic seizure model or 6‐Hz seizure model (**Figure** **5**B), demonstrating that acute or chronic Kir4.1 inhibition induced by **JX3212** administration did not alter seizure susceptibility. Moreover, chronic administration of **JX3212** (3/10 mg kg^−1^) for 14 days did not elevate hippocampal levels of the pro‐inflammatory cytokines interleukin‐6 or tumor necrosis factor‐alpha (Figure S9, Supporting Information).

Although (*S*)‐ketamine demonstrates efficacy in treatment‐resistant depression, this rapid‐acting antidepressant is associated with significant adverse effects, including dissociative hallucinations and potential exacerbation of psychotic symptoms. To examine whether **JX3212** prevents this psychological side effect, we performed a head‐twitch response test.^[^ ^31^ ^]^ Mice were administered (*S*)‐ketamine or **JX3212** (10 mg kg^−1^), and the number of 1‐(2,5‐dimethoxy‐4‐iodophenyl)‐2‐aminopropane (DOI)‐induced head twitches was determined 30 min later. The results demonstrated that (*S*)‐ketamine‐treated mice displayed significantly more DOI‐induced head twitches over the entire 30‐min test period than the vehicle controls. In contrast, **JX3212** did not affect the number of DOI‐induced head twitches during the test period (Figure 5C).

To systematically evaluate the long‐term safety profile of **JX3212** (10 mg kg^−1^ per day), we performed comprehensive behavioral assays for anxiolytic, locomotor, cognitive, and aversive side effects following chronic administration of imipramine (10 mg kg^−1^ per day) as the active control (Figure 5A). The open field test results revealed no pronounced differences in the total distance traveled or the central distance traveled among all groups, suggesting that neither **JX3212** nor imipramine produced discernible anxiolytic or anxiogenic effects in mice (Figure 5D). The novel object recognition and Y‐maze tests revealed that imipramine treatment significantly reduced the discrimination ratio and alternation percentage compared to the vehicle group, whereas **JX3212** showed no effects (Figure 5E,F). In the shuttle box test, no significant differences were observed among groups regarding the number of active avoidance responses when mice were exposed to aversive stimuli (light and electric shock) (Figure 5G). These findings suggest that imipramine impairs learning and memory function in mice, while **JX3212** exhibits a more favorable safety profile. With respect to motor performance, the rotarod test and treadmill test revealed that chronic imipramine administration notably reduced falling latency and running distance compared with the vehicle controls, whereas **JX3212** showed no significant effects (Figure 5H,I). These findings demonstrate that long‐term imipramine treatment impairs motor coordination and endurance in mice, whereas **JX3212** exerts no measurable effect on either of these parameters.

Furthermore, during the 21‐day chronic administration period, imipramine‐treated mice exhibited a marked increase in body weight compared with the vehicle controls, which correlated with the observed hyperphagia in the NSFT. This weight gain was likely attributable to imipramine‐induced appetite stimulation, whereas **JX3212** had no evident effect on body weight (Figure 5J). Following terminal dissection, organ morphology remained normal among the experimental groups (Figure 5K), and no appreciable differences were observed in the liver‐to‐body weight ratio or kidney‐to‐body weight ratio (Figure 5L). Serum analysis revealed comparable aspartate aminotransferase and urea levels in all groups. However, the imipramine group showed a significant increase in alanine aminotransferase levels, suggesting a modest hepatotoxic effect. Both the imipramine‐ and **JX3212**‐treated groups showed marked decreases in creatinine levels, indicating potential renal effects (Figure 5M). The observed change in creatine levels may be attributed to the high renal distribution of **JX3212** (Table S3, Supporting Information) and its inhibitory effects on renal Kir4.1 channels.

Additionally, we evaluated the hERG inhibitory activity of **JX3212** and determined an IC_50_ value of 1.02 µm (Table S3, Supporting Information), suggesting potential cardiotoxicity. Finally, we conducted a 14‐day subacute toxicity study to evaluate the safety of high‐dose **JX3212** administration (100 mg kg^−1^; 10‐fold greater than the therapeutic dose; *n* = 6, *i.p*.). No mortality or significant changes in body weight or organ‐to‐body weight ratio were observed in mice administered **JX3212** compared to the control mice (Figure S10A,B, Supporting Information). Additionally, histomorphological analysis via hematoxylin‐eosin staining revealed no pronounced pathological changes in the liver, spleen or kidneys (Figure S10G, Supporting Information). Biochemical analyses of serum parameters showed a slight decrease in creatine levels in the **JX3212**‐treated group (Figure S10F, Supporting Information), consistent with observations from the 21‐day administration study (Figure 5M). In summary, these results suggested that compound **JX3212** showed acceptable safety profile. In summary, compound **JX3212** exhibited an acceptable safety profile. Thus, despite potential effects on cardiac and renal function, the overall safety profile of **JX3212** is superior to that of both (*S*)‐ketamine and imipramine in comparative assessments.

## 3 Discussion and Conclusion

Major depressive disorder has emerged as a significant global health and social issue, imposing considerable health and economic burden on society. Currently available antidepressants are limited by various drawbacks, including delayed onset of action, severe side effects, and limited efficacy in drug‐resistant populations. Consequently, safe and effective antidepressants with rapid‐onset effects and novel mechanisms of action are urgently required. Increasingly, preclinical studies have established Kir4.1 channel dysregulation as a pathological hallmark of depressive disorders and a molecular determinant of antidepressant efficacy. Previous research reported the preclinical validation of Kir4.1 as a druggable target for rapid‐acting antidepressants.^[^ ^16^ ^]^ Notwithstanding these mechanistic advances, potent Kir4.1 inhibitors with optimal drug‐like properties for depression therapeutics are still lacking.

In this study, we aimed to develop novel Kir4.1 inhibitors through systematic structural modifications of the screened lead compound EHop‐016, which is an anti‐tumor small molecule that demonstrates superior Kir4.1 inhibitory activity (IC_50_ = 0.74 ± 0.05 µm) (Figure S1, Supporting Information). We designed and synthesized 50 derivatives, and evaluated Kir4.1 inhibitory activity in vitro, which yielded **JX3212** as the most potent Kir4.1 inhibitor, with an IC_50_ value of 0.28 ± 0.02 µm (Figure 1E). **JX3212** exhibited a preference for Kir4.1 among all tested potential off‐targets, including Kir isoforms (Kir1.1/Kir2.1/Kir2.2), potassium channels distributed in astrocytes (KCNQ2), monoaminergic transporters (5‐HTT/DAT), and other established targets for rapid‐onset antidepressants (GABA_A_/5‐HT_2A_/5‐HT_2B_/NMDA receptors) (Figure 2A,B). Additionally, docking and site‐directed mutagenesis analyses revealed that **JX3212** robustly interacted with the residues E158, T128, and I159. Therefore, **JX3212** was identified as a specific small‐molecule inhibitor of Kir4.1. Effective brain penetration is a critical criterion for drugs targeting the central nervous system. The Kir4.1 inhibitor Lys05 reported in our previous study exhibits poor brain penetration, hindering its direct application in the treatment of depression. Conversely, **JX3212** exhibited excellent brain penetration with a high brain‐to‐blood distribution ratio of 10.72 (Figure 2E). The brain concentration of **JX3212** reached 2533.3 ± 745.3 ng mL^−1^ at 1 h post‐dosing with 10 mg kg^−1^, approximately equal to 6.3 µm, suggesting consistency between the in vivo responses and in vitro activity for Kir4.1 channels.

Importantly, **JX3212** demonstrated consistent antidepressant efficacy in multiple depression models, including FST, NSFT, CSDS and OBX. Notably, in the NSFT and CSDS models, which have high sensitivity to the timing of antidepressant action, a single dose of **JX3212** (10 mg kg^−1^) exhibited antidepressant effects comparable to those of (*S*)‐ketamine as early as 1‐h post‐administration (Figure 3B,H–J). However, the traditional antidepressant imipramine failed to show significant efficacy in NSFT after a single dose (Figure 3C), highlighting the advantage of **JX3212** for rapid‐onset antidepressant action. Furthermore, repeated administration of **JX3212** did not induce tolerance, underscoring its sustained therapeutic potential (Figure 2E).

The electrophysiological alterations observed in astrocytes from Kir4.1 cKO mice, including reduced potassium currents, impaired conductance, and depolarized resting membrane potentials, are consistent with the established role of Kir4.1, which maintains astrocytic membrane potential and potassium buffering capacity. Notably, **JX3212** treatment recapitulated these key features in WT astrocytes, whereas these effects were abolished in Kir4.1 cKO cells. This genetic‐pharmacological concordance robustly validates the specificity of **JX3212** for Kir4.1 (Figure 4). Critically, **JX3212**‐mediated behavioral effects were abolished in Kir4.1 cKO mice, wherein **JX3212** administration (10 mg kg^−1^, *i.p*.) failed to alter NSFT feeding latency compared with vehicle controls (Figure 4H). This complete abrogation of pharmacological efficacy in cKO models mechanistically demonstrates that the antidepressant efficacy of **JX3212** is strictly dependent on a functional Kir4.1 channel.

Safety is a critical priority in the development of novel antidepressants. Unlike (*S*)‐ketamine, the rapid‐acting antidepressant **JX3212** did not induce DOI‐mediated head‐twitch responses in mice, confirming its lack of psychotomimetic effects (Figure 5C). Compared to the traditional antidepressant imipramine, chronic administration of **JX3212** preserved cognitive function (memory/learning) and locomotor activity in mice, without inducing weight gain or hepatotoxicity (Figure 5D–M). Critically, Kir4.1 inhibition by **JX3212** at a therapeutic dose (10 mg kg^−1^) elicited no proconvulsant effects, either acutely or chronically (Figure 5B), and no overt toxicity was observed even at a higher dose (100 mg kg^−1^) (Figure S10, Supporting Information). Although **JX3212** exhibits potent hERG inhibition and a concomitant reduction in serum creatine levels, suggesting potential effects on cardiac and renal function, its overall safety profile remains favorable, particularly when compared to that of imipramine and (*S*)‐ketamine.

The following areas require further investigation. 1) Incomplete Kir channel profiling. Although we assessed **JX3212** against selected Kir subtypes (Kir1.1, Kir2.1, Kir2.2, and Kir4.2), the contribution of the remaining Kir isoforms (e.g., Kir4.x, Kir6.x) and heteromeric assemblies (Kir4.1/5.1) to antidepressant efficacy remains unexplored. Differential expression patterns of Kir channels across brain regions (e.g., prefrontal cortex vs. hippocampus) may underlie the variable therapeutic responses, warranting comprehensive electrophysiological profiling in future studies. 2) Cross‐species translation. Robust antidepressant‐like responses have been observed in mice; however, extrapolation to humans remains speculative. Future studies will aim to elucidate the expression patterns of Kir4.1 in human astrocytes and investigate potential species‐specific differences in astrocyte‐neuron coupling. 3) Sex bias. Although our study exclusively employed male mice as the animal model, we acknowledge this limitation given the well‐documented sex differences in depression prevalence, with higher incidence rates observed in females. Future studies should incorporate sex‐specific analyses to address this critical gap in translational research.

In summary, we successfully synthesized and evaluated a novel compound, **JX3212**, which acts as a specific inhibitor of the Kir4.1 channel. Through high brain penetration and good druggability, the compound **JX3212** could induce a rapid‐onset antidepressant effect comparable to that of (*S*)‐ketamine, thereby expanding the treatment options for patients with major depressive disorder. Furthermore, this study establishes a foundational basis for the continued development of Kir4.1 inhibitors as potential therapeutic agents for depression.

## 4 Experimental Section

### Synthesis and Characterization of **JX3201‒50**

The details of the synthesis and characterization of **JX3201**‒**50** employed in this manuscript are described in the Supporting Information.

### Plasmids and Mutagenesis

Plasmids encoding WT human potassium channels Kir1.1, Kir2.1, Kir2.2, Kir4.1, and Kir4.2 were obtained from the Beijing Genomics Institute. All point mutations were constructed using the QuickChange II Site‐Directed Mutagenesis Kit (Stratagene) and subsequently verified by sequencing.

### Cell Culture and Transfection

CHO‐K1 cells were maintained in DMEM/F12 growth medium containing 10% fetal bovine serum. Plasmids encoding the target genes (3600 ng) and enhanced green fluorescent protein (400 ng) were cotransfected into cells using Lipofectamine 2000 (Invitrogen) in accordance with the manufacturer's instructions. Cells were seeded onto poly‐d‐lysine (PDL)‐coated glass coverslips 1 h prior to electrophysiological recordings. All cells were cultured under standard conditions of 5% CO_2_ at 37 °C.

Astrocytes were isolated and cultured as previously described.^[^ ^16^ ^]^ Briefly, the cortices from P1‐P4 mouse pups were dissected and incubated for 25 min in Hank's Balanced Salt Solution (22.5 ml) with 2.5% trypsin (2.5 ml) at 37 °C. Cells were dissociated in cultured medium (DMEM/F12 supplemented with 10% fetal bovine serum and 1% penicillin and streptomycin) and filtered with a 70‐µm cell strainer. Cell suspensions were cultured in PDL‐pretreated 75‐cm^2^ flasks for the initial seven days, with half of the medium replaced every 2 days. Subsequently, the cultures were shaken at 220 rpm for 6 h at 37 °C to remove microglia and oligodendrocytes from the astrocyte layers. The astrocytes were harvested, reseeded in two new flasks, and cultured in an incubator for 14 days. For Kir4.1 deletion in cell cultures, 4‐OH tamoxifen (final concentration: 1 µm, Felixbio) was added into Kir4.1^flox/flox: Aldh1l1‐Cre/ERT2^ cortical cultures at DIV 2 and DIV 4, respectively, and completely removed at DIV7 when astrocytes were reseeded in fresh culture medium.

### Electrophysiological Recordings

Whole‐cell patch‐clamp recordings were obtained either in transiently transfected CHO‐K1 cells or in primary cultured astrocytes at room temperature using standard patch‐clamp techniques with an Axopatch‐700B amplifier (Axon Instruments). The signals were filtered at 2 kHz and sampled at 20 kHz using the Digidata 1440A interface (Axon Instruments). The resistance of borosilicate glass pipettes was typically 2–4 MΩ when filled with the internal solution. Drug application was facilitated using a BPS perfusion system (ALA Scientific Instruments). Macroscopic currents generated by expressed potassium channels were recorded under the voltage‐clamp conditions with an internal pipette solution composed of (in mM): 140 KCl, 5 NaCl, 1 CaCl_2_, 1 MgCl_2_, 10 HEPES, and 10 EGTA, pH 7.2 adjusted with KOH. The external solution contained (in mM): 140 NaCl, 5 KCl, 2 CaCl_2_, 1 MgCl_2_, 10 glucose, and 10 HEPES, pH 7.4 adjusted with NaOH. Currents were elicited with a hyperpolarized voltage at −120 mV from the holding potential of −80 mV, followed by a ramp voltage depolarizing to +50 mV. Astrocytic resting membrane potentials were recorded under current‐clamp conditions (I = 0 pA) using standard internal and external solutions, determined immediately after achieving whole‐cell mode.

For recordings of α1β2γ2 GABA_A_ receptors, CHO‐K1 cells stably expressing GABA_A_ subunits α1β2γ2 were voltage‐clamped at −60 mV. 1 µm γ‐aminobutyric acid (GABA) was applied by rapid solution exchange system in the presence or absence of test compounds to determine agonistic effects. The pipette solution contained (in mM): 140 KCl, 5 NaCl,1 MgCl_2_, 1 CaCl_2_, 10 EGTA and 10 HEPES (pH 7.3 adjusted with KOH). The bath solution contained: 142 NaCl, 8.1 KCl, 6 MgCl_2_, 1 CaCl_2_ and 10 HEPES (pH 7.4 adjusted with NaOH).

For recording of GluN1/GluN2B NMDA receptors, HEK293T cells stably expressing GluN1 and GluN2B subunits were voltage‐clamped at −60 mV. Currents were evoked by co‐application of glutamate (100 µm) and glycine (100 µm), and the resulting inhibition by the test compounds was measured to determine IC_50_ values. The pipette solution contained (in mM):115 CsF, 10 CsCl, 10 HEPES and 10 BAPTA (pH 7.2 adjusted by CsOH). The bath solution contained (in mM): 140 NaCl, 2.8 KCl, 1 CaCl_2_, 10 HEPES and 20 sucrose (pH 7.4 adjusted with NaOH).

### 5‐HT_2A_/5‐HT_2B_ Functional Assays

Cells with inducible expression of target receptor were cultured in 175‐cm^2^ flasks until ≈80% confluence, washed once with phosphate‐buffered saline (PBS), and dissociated using 0.25% trypsin–EDTA. After neutralization with culture medium and centrifugation (800 rpm, 3 min), the cells were resuspended, counted, and seeded onto poly‐D‐lysine‐coated 96‐well black plates at a density of 4 × 10^4^ cells/well. Expression of the target receptor was induced with 1 µg mL^−1^ doxycycline. After 30 min at room temperature, the plates were incubated at 37 °C, 5% CO_2_ for 18−24 h until reaching ≈90% confluence. The medium was replaced with Ca^2^⁺‐free extracellular buffer, washed once, and incubated with Fluo‐4 AM for 60 min at 37 °C in the dark. After washing twice with extracellular buffer, extracellular buffer was added (80 µL/well), and the cells were incubated for an additional 20 min at room temperature before fluorescence recording. Real‐time fluorescence changes were recorded using an FDSS/µCell system at 480 nm excitation and 540 nm emission with a sampling rate of 1 Hz. After a 10‐s baseline, the test compounds were added (20 µL/well), and fluorescence was recorded for 180 s. Subsequently, of receptor‐specific stimulus buffer was added (20 µL/well), and the signals were monitored for a total of 360 s.

The agonistic effect (%) of compounds was calculated as follows:

(1) $$\mathrm{Agonist}\%\;=\frac{\left(R_{\mathrm{compound}}-R_{\mathrm{control}}\right)}{R_{\mathrm{control}}}\;\times 100\%$$

where *R*_control_ is the maximal fluorescence ratio after the addition of 0.5% DMSO and *R*_compound_is the ratio obtained after compound treatment. Fluorescence response curves and EC_50_ values were used to evaluate the agonistic potency. The extracellular (Ca^2+^‐free) buffer contained (in mM): 140 NaCl, 5 KCl, 1 MgCl_2_, 10 glucose, 10 HEPES, 2 probenecid; pH adjusted to 7.4 with NaOH.

### hERG Inhibition

CHO‐hERG cells were cultured to 60–80% confluency in 175‐cm^2^ flasks, washed with PBS, and digested with detachin. After centrifugation, the cells were resuspended in medium at 2–5×10^6^ mL^−1^. Patch‐clamp recordings were performed using a QPatch with extracellular solution (140 NaCl, 5 KCl, 2 CaCl_2_, 1 MgCl_2_, 10 HEPES, 10 glucose, pH 7.4) and intracellular solution (145 KCl, 1 MgCl_2_, 5 EGTA, 10 HEPES, 5 Mg‐ATP, pH 7.3). Cells were clamped at −80 mV, stimulated with a 50 ms pre‐pulse to −50 mV, followed by 2 s depolarization to +40 mV, and 2 s repolarization to −50 mV, repeated every 5 s. After baseline recording, the test compound at various concentrations or 300 nM cisapride (positive control) was applied.

### Cytotoxicity Test

Cytotoxicity of all the tested compounds was determined using the cell counting kit‐8 (CCK‐8) assay kit (Beyotime, Shanghai, China). Briefly, MDA‐MB‐468 was cultured in L15 medium and plated in 96‐well plates at a density of 12,000 cells per well for 24 h at 37 °C in a 5% CO_2_ incubator. The tested compounds were dissolved in DMSO and diluted with culture medium (DMSO final concentration < 0.4%). Then the cells were treated with compounds (0.41, 1.23, 3.70, 11.11, 33.33, 100.00 µm) for 48 h. After treatments, removal of the supernatant liquor, 100 µL of new media diluted CCK‐8 solution (10% CCK‐8) was added into each well and incubated at 37 °C for 2 h. The absorbance of the mixture was directly measured at 450 nm. The results are presented as a percentage of the control.

### 5‐HTT/DAT Functional Assays

The 5‐HTT/DAT functional assays were commissioned by Elsevier Science and Technology Co., Ltd. (Beijing). A fluorescence‐based neurotransmitter assay was conducted using a Neurotransmitter Transporter Uptake Assay Kit (Molecular Devices, R8174), according to the manufacturer's protocol. Flp‐in T‐rex 293 cells transiently expressing the human norepinephrine transporter and HEK293 cells stably expressing the human dopamine or serotonin transporters were seeded in PDL‐coated 384‐well plates and incubated overnight. Subsequently, the medium was removed, and the cells were treated with 16 µL of compounds diluted in assay buffer for 0.5 h at 37 °C. Centanafadine and 0.1% DMSO served as positive and negative controls, respectively. Then, 16 µL of dye mix prepared in 1× Hanks’ Balanced Salt Solution was added to each well and incubated at 37 °C for 1 h. Finally, the data point was acquired via a fluorescence plate reader with excitation at 450 nm and emission at 528 nm.

### Molecular Docking

Molecular docking studies were performed using the Glide module in the Schrödinger software package 2021. The crystal structure of Kir4.1 (PDB ID: 8I5M) was obtained from the Protein Data Bank (https://www.rcsb.org/). Crystal structures were prepared using the Protein Preparation Wizard workflow. All water molecules were removed. A grid file for molecular docking was generated based on the binding sites. **JX3212** was prepared using LigPrep and docked using the Glide extra precision mode. Default settings were used for all other parameters. PyMOL was used to visualize molecular interactions between ligands and protein active‐site residues.

### Brain Penetration and Plasma/Tissue Distribution

Male C57BL/6J mice were used for both blood–brain penetration and plasma/tissue distribution studies, with mice assigned to three groups: 10 mg kg^−1^ **JX3212** (*n* = 3), 20 mg kg^−1^ **JX3212** (*n* = 3) for blood–brain penetration, and 10 mg kg^−1^ **JX3212** for plasma/tissue distribution (all administered via intraperitoneal injection), where mice in the blood–brain penetration groups were fasted for 12 h prior to drug administration and 2 h post‐administration; 1 h after drug administration, 0.2 mL of blood was collected from the retro‐orbital/orbital venous plexus into 1.5 mL heparinized tubes, centrifuged at 11 000 rpm for 5 min to obtain plasma (stored at −80 °C for blood–brain penetration groups), while whole brains were excised for blood–brain penetration groups and whole brains plus kidneys (rinsed with physiological saline, blotted dry on filter paper, and accurately weighed) for the plasma/tissue distribution group; plasma samples (20 µL) were mixed with 20 µL internal standard solution and 300 µL acetonitrile for protein precipitation, vortexed for 15 min, centrifuged at 3700 rpm for 15 min at 4 °C, and the supernatant was collected, whereas brain and kidney samples were homogenized in acetonitrile at 10× tissue weight (8000 rpm, 3 × 30 s), sonicated for 5 min, and processed identically to plasma samples; the concentrations of JX3212 in plasma and tissues were quantified by liquid chromatography–tandem mass spectrometry (LC−MS/MS), with lower limits of quantification set to 50 ng mL^−1^ for plasma and 50 ng g^−1^ for brain tissue.

### Western Blot Analysis

Mature astrocytes were washed with PBS, dissociated with 0.25% trypsin–EDTA, collected by centrifugation, resuspended in PBS, and snap‐frozen in liquid nitrogen. Total protein was extracted using RIPA buffer containing protease inhibitors. Protein concentration was determined using a bicinchoninic acid assay to ensure equal loading. Equal amounts of protein were separated by SDS sulfate‐polyacrylamide–PAGE and transferred onto PVDF membranes. Membranes were blocked with 5% non‐fat milk in TBST for 1 h at room temperature and incubated with the primary antibody at 4 °C overnight. After washing, membranes were incubated with secondary antibodies for 2 h at room temperature. The protein bands were visualized using enhanced chemiluminescence and imaged using a chemiluminescence detection system. Band intensities were quantified using ImageJ software. The expression level of Kir4.1, normalized to that of the internal control, was expressed relative to that of the WT group. The antibodies were used: rabbit anti‐Kir4.1 (1:2000, APC‐035, Alomone Laboratories), mouse anti‐GAPDH (glyceraldehyde 3‐phosphate dehydrogenase) (1:5000, 30201ES20, Yeasen), peroxidase‐conjugated goat anti‐rabbit IgG (H + L) (1:5000, 33101ES60, Yeasen), and peroxidase affiniPure goat anti‐mouse IgG (H + L) (1:5000, 33201ES60, Yeasen).

### Immunofluorescence Staining of Paraffin‐Embedded Brain Sections

The mice were maintained for an additional seven days before tissue collection. Mice were deeply anesthetized and transcardially perfused with PBS, followed by fixation with 4% paraformaldehyde. The brains were dissected and post‐fixed in 4% paraformaldehyde at 4 °C overnight, dehydrated through a graded ethanol series, cleared in xylene, and embedded in paraffin. Coronal brain sections (3–4 µm thick) were mounted on glass slides. The sections were deparaffinized in xylene, rehydrated using graded ethanol, quenched endogenous peroxidase activity with 3% H_2_O_2_ solutions, and subjected to antigen retrieval by boiling in EDTA buffer (pH 9.0) for 15 min. After cooling to room temperature, the sections were rinsed with PBS and followed by blocked with blocking solution (5% bovine serum albumin) for 20 min. The sections were sequentially incubated with the primary antibody against Kir4.1 or GFAP overnight at 4 °C and horseradish peroxidase‐conjugated secondary antibody for 1 h at room temperature in the dark, finally, the Cy3‐ or Alexa Fluor488‐labelled tyramide amplification reagent (Runnerbio) according to the manufacturer's instructions. The antibodies applied were rabbit anti‐Kir4.1 (1: 1000, APC‐035, Alomone Laboratories), anti‐GFAP (1:2000, ab7260, Abcam), and HRP‐conjugated goat anti‐rabbit IgG (1:2000, ab205718, Abcam). Nuclei were counterstained with DAPI (1 µg mL^−1^), and slides were mounted with antifade mounting medium before imaging.

### Animal Experiments

All experiments involving animals were conducted in compliance with the World Medical Association's Code of Ethics and adhered to the guidelines set forth by the Institutional Animal Care and Use Committee (IACUC). The protocols were reviewed by the IACUC and the Internal Ethics Committee of the Shanghai Institute of Materia Medica, which also approved the study (IACUC no. 2022‐02‐GZB‐11). The animals were kept in an environment with regulated temperature (22–24 °C) and stable humidity (55 ± 10%), subjected to a 12‐h light/dark cycle, and provided with standard chow and free access to water. All animal experiments were performed under blinded conditions, meaning that different researchers handled the administration of drugs and execution of behavioral tests. Behavioral assessments were performed between 10:00 and 16:00.

Male C57BL/6J mice (6–10 weeks old) and male CD‐1 mice (4–6 months old) were purchased from Shanghai Shengchang Biotechnology Co., Ltd. *Kcnj10* ^flox/flox^ mice were generated by the Cyagen Company (Suzhou, China). The Cas9 protein, two gRNAs (gRNA‐A1: TAAATGCCCGGGACAGCGTGAGG, gRNA‐A2: TTCTAGTGGTGGGCGTTTGGGGG) and a donor vector containing two loxP sequences flanking exon 2 of mouse *Kcnj10* were co‐injected into fertilized eggs. The embryos were transferred to recipient female mice to obtain F0 mice. The genotype was confirmed by PCR using two pairs of primers (F1: 5’‐GTGTCTTAGCAGGAATGATGTTGT‐3’, R1: 5’‐CCTTCCTTTTGATGGCATTGATCTT‐3’; F2: 5’‐ATGCTTGGAGATTCACATTCAGAG‐3’, R2: 5’‐CAGGGAACTTAGCTAGATGGAAGA‐3’) and sequencing. The F0 founder mice were mated with C57BL/6J mice to produce heterozygous F1 offspring. Subsequent intercrosses of F1 heterozygous mice ultimately produced F2 homozygous mice with the *Kcnj10* ^flox/flox^ genotype. Tamoxifen‐induced conditional transgenic mice with Kir4.1 deletion in astrocytes were generated by crossing *Aldh1l1*‐Cre/ERT2 mice with homozygous *Kcnj10* ^flox/flox^ mice.^[^ ^32^ ^]^ For tamoxifen induction, adult mice aged 8–12 weeks were intraperitoneally administered tamoxifen (75 mg kg^−1^, dissolved in corn oil) for 7 consecutive days to yield astrocyte‐specific Kir4.1 knockout animals. Behavioral testing was conducted seven days after the tamoxifen injection.

### Novelty‐Suppressed Feeding Test

The mice were individually housed in new cages and deprived of food for 24 h. The testing equipment was a brightly lit plastic box measuring 40 × 40 × 30 cm^3^ with a light intensity of 1100–1200 lux. Each mouse was positioned in a corner of the device with one food pellet located at the center. The latency to start chewing food was recorded within 5 min. Immediately thereafter, the mice were placed back in their home cage with pre‐measured food, and the amount consumed in the next 5 min was used as a baseline to account for appetite changes that might affect the outcome.

### Chronic Social Defeat Stress Model

The 10‐day CSDS paradigm was conducted as previously described.^[^ ^24^ ^]^ CD‐1 aggressive mice were singly housed in defeat cages on one side of a perforated plexiglass partition 24 h before the defeat session. Experimental C57BL/6J mice were placed daily on the resident cage side of a novel CD‐1 aggressor that rapidly initiated a physical attack on the intruder. After 10‐min of social defeat, the intruder mice were housed on the opposite side of the partition within a shared home cage, experiencing further sensory contact for the subsequent 24 h. In contrast, control mice were maintained in their home cages and allowed to explore the empty defeat cages for 10 min each day.

### Sucrose Preference Test

Mice were individually housed with two identical bottles containing either 1% sucrose solution or tap water for 24 h. The bottles were then replaced to eliminate potential positional bias, followed by an additional 24‐h period with the same solutions. Subsequently, the mice were provided with tap water for 24 h. During the experimental phase, new bottles filled with either 1% sucrose solution or tap water were presented for 24 h, and the bottles were changed every 12 h. The bottles were weighed both before and after the 24‐h test to assess consumption of each solution.

### Forced Swim Test

Mice were placed individually in a transparent glass cylinder (25 cm high, 10 cm wide) filled with water (temperature 23–25 °C) to a depth of 10 cm, and prevented from making contact with the bottom. Mice behaviors were videotaped from each side. The observation period lasted for 6 min, with the final 4 min used to measure immobile behavior, which was processed using the AnyMaze Tracking System (Stoelting).

### Social Interaction Test

C57BL/6J mice, comprising both control and experimental groups, were placed individually into the social open field with a plexiglass enclosure positioned along the side wall. Movement parameters, including the time spent in the interaction zone, corner zones, and locomotion, were monitored and analyzed over the two test sessions using the AnyMaze Tracking System. In the first session, the mice freely explored the arena for 2.5 min. Then, a new aggressive CD‐1 mouse was introduced into the plexiglass enclosure, and the experimental mice returned to the arena for the second 2.5‐min session. The social interaction ratio was determined as the ratio between the time the experimental mice spent in the interaction zone when the target CD‐1 was present and the time spent in the zone when it was absent. Mice with a social interaction ratio of ≥1 showed a behavioral profile similar to unstressed control mice and were termed resilient, whereas mice with a social interaction ratio <1 were termed susceptible.

### Olfactory Bulbectomy

Male C57BL/6J mice (8–12 weeks old) were anesthetized by intraperitoneal injection of tiletamine‐zolazepam‐xylazine. Following confirmation of anesthesia, a midline scalp incision was made to expose the skull, and the bregma was identified as the anatomical reference point. Using a stereotaxic frame (Stoelting), bilateral olfactory bulbs (4 mm anterior to the bregma) were aspirated through a 1‐mm burr hole drilled with a dental drill. Hemostatic sponges were applied to the burr holes, and the incision was closed with 5‐0 silk sutures. The sham‐operated controls underwent identical procedures without bulb aspiration. Prophylactic antibiotic therapy (25% ampicillin, 0.1 mg kg^−1^, *i.p*.) was administered daily for three consecutive days post‐surgery. The animals were monitored during a 14‐day recovery period prior to behavioral assessments. After euthanasia, successful bulbectomy was verified by gross anatomical inspection, and habenular tissues were dissected on ice for subsequent molecular analyses.

### Seizure Threshold Measurement in 6‐Hz Seizure Test

Male C57BL/6J mice were subjected to corneal electroshock stimulation using a Grass S48 stimulator (Grass Technologies) with standardized parameters: frequency, 6 Hz; rectangular pulse width, 0.2 ms; and duration, 3 s. Seizure activity was operationally defined as the occurrence of any of the following behaviors: forelimb clonus, postictal immobility, facial myoclonus, staring behavior, orofacial automatization (chewing/unilateral pawing), or Straub tail posture. Animals exhibiting full behavioral recovery within 7 s post‐stimulation were classified as seizure‐resistant. The seizure incidence was compared between the vehicle‐ and **JX3212**‐treated groups, with the CS_50_ threshold calculated as the minimal current intensity required to induce seizures in 50% of the subjects via probit analysis.

### Seizure Threshold Measurement in Maximal Electroshock

Male C57BL/6J mice underwent corneal electroshock stimulation using an Orchid Scientifics EC‐02 stimulator with a fixed pulse duration of 0.2 s. The current intensity was titrated in a stepwise manner (0.1 mA increments) based on real‐time behavioral responses during serial testing. Seizure severity was quantified using the hindlimb tonic extension score, with rigorous blinding applied to the vehicle‐ and **JX3212**‐treated groups. Subsequently, CS_50_ values were determined.

### DOI‐Induced Head Twitch

Male C57BL/6J mice were habituated to the testing chambers for 30 min and 24 h after receiving acute doses of (*S*)‐ketamine (10 mg kg^−1^, *i.p*.) or **JX3212** (10 mg kg^−1^, *i.p*.). Thirty minutes later, the mice received DOI at a dose of 5 mg kg^−1^ (*i.p*.), and the head‐twitch response was scored over 30 min.

### Open Field Test

This test was used to assess anxiety‐related behaviors in the mice. C57BL/6J mice were individually habituated to a 40 × 40 × 40 cm^3^ open field arena under dim light for 1 min. Locomotor activity during the final 5 min was digitally recorded (30 fps) and analyzed using Tracking Master (Future Life Science (Shanghai) Co., Ltd., Shanghai, China). The primary endpoints included the total ambulatory distance (cm) and center‐zone exploration frequency (20 × 20 cm^2^ central area).

### Novel Object Recognition Test

This test was designed to assess memory in rodents. The mice were individually exposed to familiar and novel objects. The test consists of three phases: habituation, training, and retention. During habituation, each animal freely traversed an empty cubic arena (≈40 cm per side) for 5 min, then was returned to its home cage. In the subsequent training phase, two identical rectangular objects were fixed 10 cm from opposite walls, and the mouse, introduced with its snout directed toward the center of the distal wall, was allowed to investigate the objects for 5 min. One hour later, in the retention phase, one of the familiar objects was exchanged for a geometrically distinct but comparably sized novel object, and exploration was recorded for 5 min. The positions of familiar and novel objects were systematically randomized across trials to prevent spatial bias. Object exploration was quantified as the duration of direct nose/mouth orientation toward objects within ≤ 2 cm, excluding instances of turning away or sitting on objects. Recognition memory was assessed using the discrimination ratio, calculated as N/(N + F) × 100%, where N represents the exploration time of the novel object and F denotes the exploration time of the familiar object.

### Y‐Maze

This test was designed to evaluate spatial working memory. Briefly, mice were individually placed in a symmetrical Y‐maze and allowed to explore for 8 min. Entries were counted only when both hind paws crossed the arm threshold. Correct spatial alternation was defined as an entry into all three arms in an uninterrupted sequence (e.g., ABC, BCA or CAB each representing one alternation). The alternation percentage was derived as: [correct alternations/(total entries–2)] × 100%. After each session, the walls and floor of the maze were wiped with 75% ethanol and allowed to dry before the next animal was tested.

### Shuttle Box Test

This test was designed to measure learning and memory function in rodents. The shuttle box consists of two compartments connected by a freely passable gate. The mice were allowed to explore the test chamber freely for 5 min before the test to eliminate exploratory reflexes. Each mouse was placed in one compartment of the shuttle box. The conditioning protocol began with a 5‐s conditioned stimulus presentation of light (200 lux) and auditory tone (100 dB, 1000 Hz), followed immediately by a 3‐s footshock (300 µA) unconditioned stimulus. Escape to the opposite compartment during the conditioned stimulus presentation was scored as an active avoidance response, whereas escape occurring only after shock onset was recorded as passive avoidance. Through repeated training sessions, the mice progressively acquired conditioned active avoidance responses, demonstrating memory formation. Each test session consisted of 40 trials with 10‐s inter‐trial intervals, with the number of active avoidance responses systematically recorded.

### Rotarod Test

The rotarod test was designed to evaluate the coordination and motor performance of rodents. The speed and time were set for the rod rotators. All experimental mice were allowed to adapt to a lower speed (5 rpm). For the formal test, the mice were placed on a rod spinning at 20 rpm, and the duration that the mice were able to hold on the rod at this speed was recorded. The maximum test duration was 20 min.

### Treadmill Test

This test was designed to quantify locomotor endurance in rodents. The time (300 s) and speed (maximum: 30 rpm) parameters for the treadmill were set using a digital panel and the mice were placed in lanes. Gentle electrical probes (0.3 mA) served as motivational stimuli. Total distance traveled was automatically recorded using rotary encoder integration.

### Repeat‐Dose Toxicity Studies

For the subacute toxicity study, mice were divided into two groups (*n* = 6): control group (*i.p*.) and **JX3212**‐treated group (100 mg kg^−1^, *i.p*.). The mice received daily treatments for 14 consecutive days and were monitored once daily for signs of toxicity. Individual body weights were measured daily throughout the observation period. At the end of this period, all mice were sacrificed and dissected. Macroscopic evaluation was performed, and the heart, liver, spleen, lungs, kidneys, and brain were excised for relative weight calculations. Representative organ fragments were embedded in formalin and paraffin, then fixed for subsequent histopathological analysis. Serum biochemical analyses included determination of creatinine, urea, alanine aminotransferase, and aspartate aminotransferase.

### Statistical Analyses

Data are expressed as the mean ± standard error of the mean or the mean ± standard deviation, with specified sample sizes (n) indicating biological replicates. Survival rates were analyzed using the Kaplan–Meier method. An unpaired two‐tailed Student's *t*‐test was used to assess statistical significance between two datasets. For comparisons involving more than two groups, statistical significance was determined using one‐way and two‐way analysis of variance with Dunnett's test and Bonferroni's test, respectively, using Prism 8 software.

## Conflict of Interest

The authors declare no conflicts of interest.

## Author Contributions

S.S.W., X.Y.Z., and M.D.L. contributed equally to this work. J.L., Z.B.G., and Y.X.X. designed the study. S.S.W., X.Y.Z., and Y.X.X. wrote the manuscript. S.S.W., C.Z., H.G., T.Y.T., H.F.L., and T.T.L. synthesized the derivatives. X.Y.Z., J.Y.H., and M.D.L. performed the in vitro experiments. S.S.W., X.Y.Z., H.Y.X., L.Z., and M.D.L. performed the in vivo experiments. X.Y.Z., Y.X.X., and Y.M.Z. analyzed and interpreted the data. All co‐authors contributed comments.

## Supporting information



Supporting Information

Supporting Information

## Data Availability

The data that support the findings of this study are available from the corresponding author upon reasonable request.
