# Correction to “Discovery of a Novel and Potent Kir4.1 Inhibitor as a Safe and Rapid‐Onset Antidepressant Agent in Mice”

**DOI:** 10.1002/advs.74813

**Published:** 2026-03-14

Wang S, Zhou X, Li M, Zhang C, Xu H, He J, Zhan L, Gu Y, Gu H, Tu T, Liu H, Lu T, Zheng Y, Li J, Gao Z, Xu Y. Discovery of a Novel and Potent Kir4.1 Inhibitor as a Safe and Rapid‐Onset Antidepressant Agent in Mice.

DOI: 10.1002/advs.202509506. *Adv. Sci*. 2025 February; 13(9):e09506.

1. In paragraph 1 of “2.7. JX3212 Acts on Astrocytic Kir4.1 to Exert Rapid‐Onset Antidepressant Effects” section, the term “*Kcnj10* ^flow/flow^” was incorrect. This should have read: “*Kcnj10* ^flox/flox^”.

2. In Figure 5B, the image depicting seizure susceptibility test results was incorrect. The CS_5_₀ data of “Single dose‐MES” and “Multiple dose‐6 Hz” were inadvertently swapped during figure assembly. This is now being corrected.

Original version:


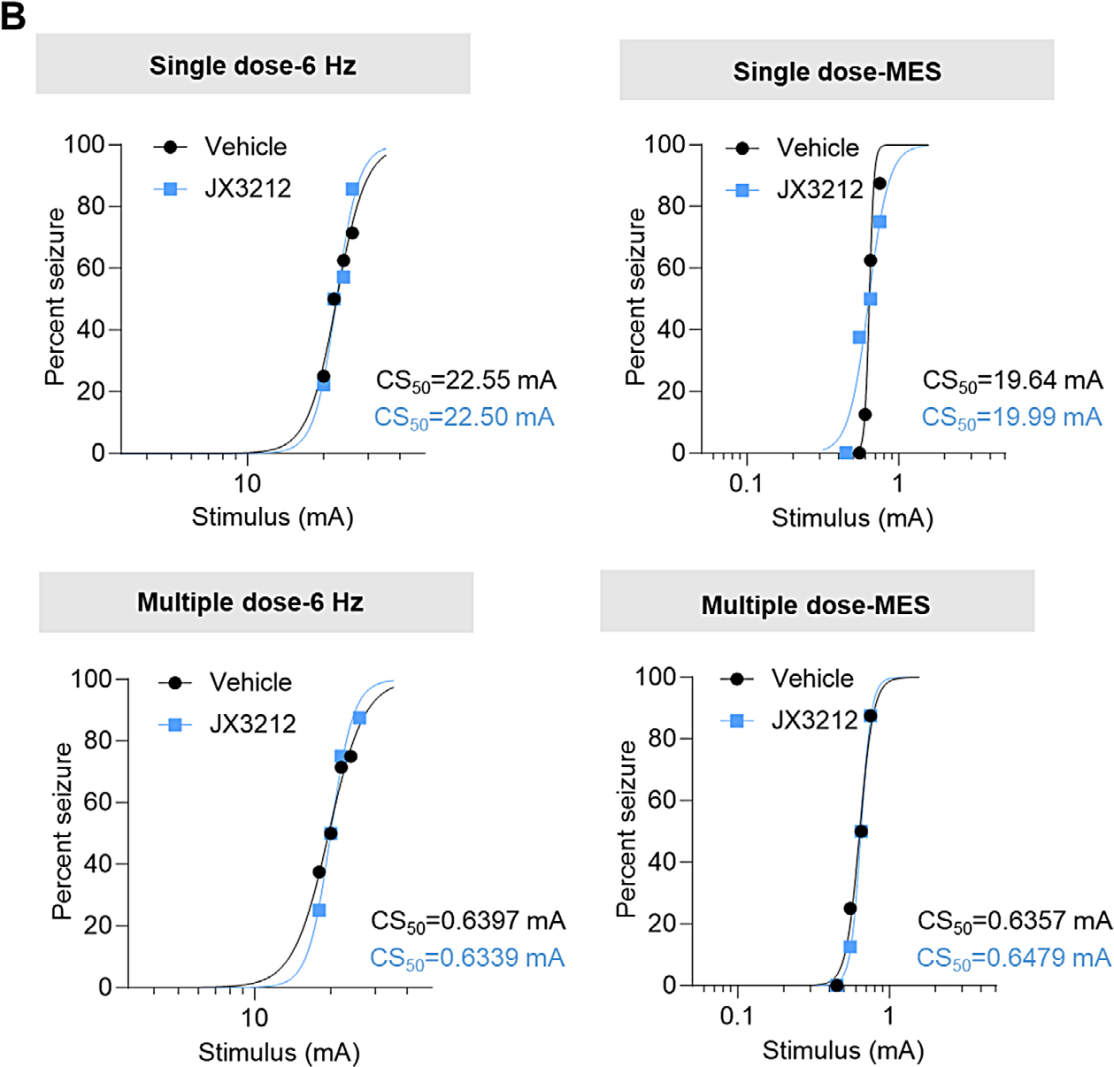


Revised version:


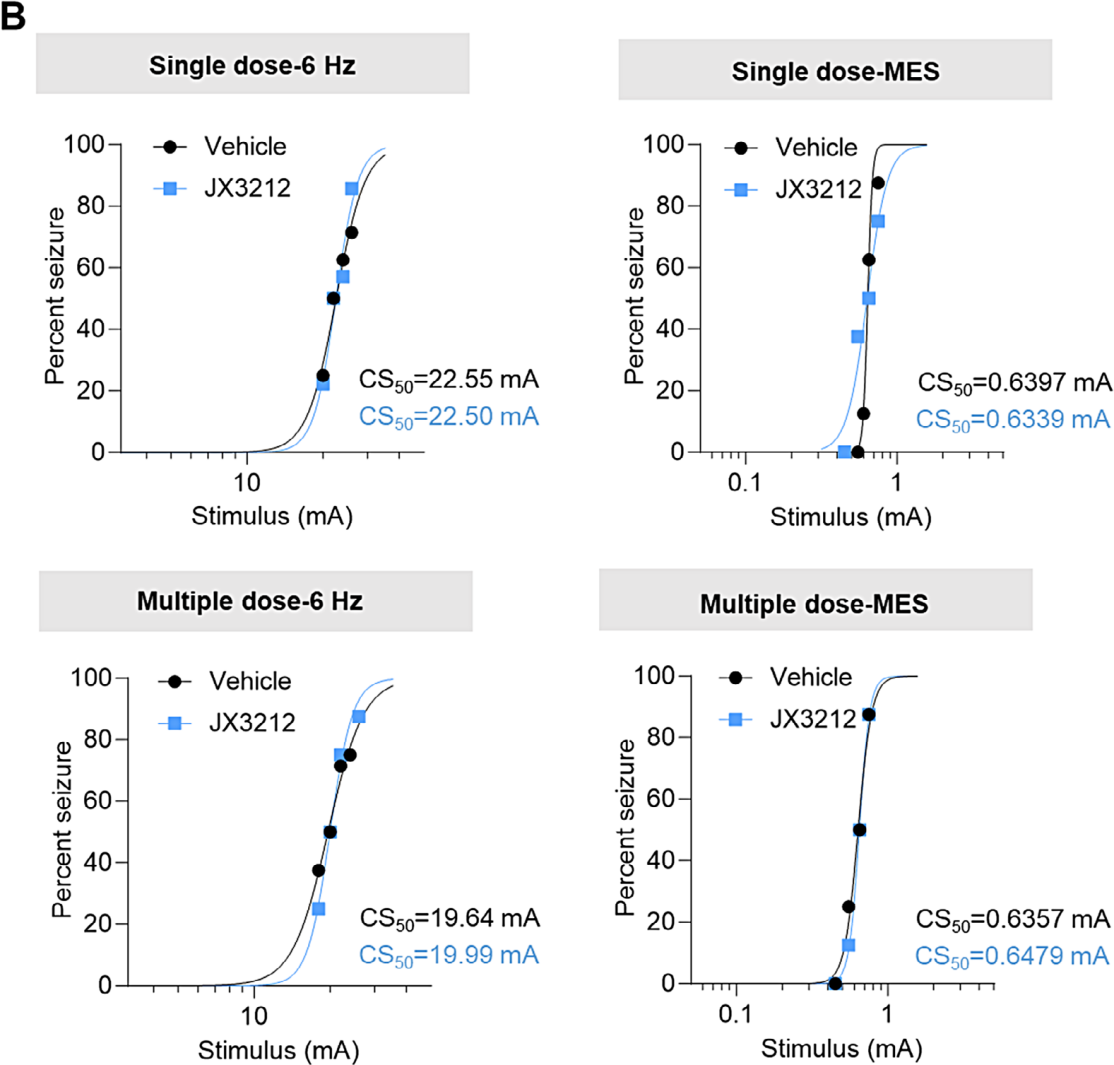


3. In paragraph 2 of “Animal Experiments” section in “4. Experimental Section”, the text “seven days” was incorrect. This should have read: “five days”.

4. In the Figure S4, the compound **JX3212** was erroneously referenced by its original identifier (**32078**). This has been corrected to “**JX3212**” in the revised figure.

Original version:


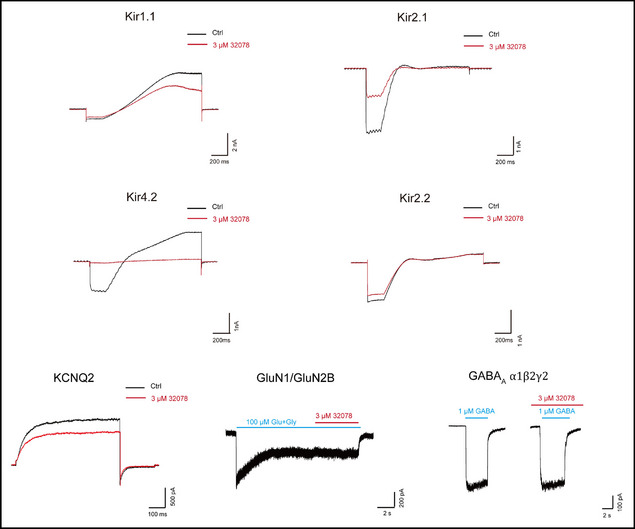


Revised version:


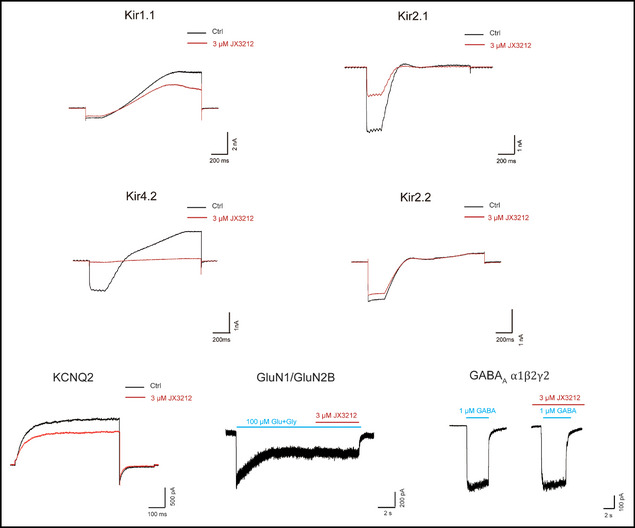


We apologize for these errors.

