# Supplementary material for: Discovery of a Novel and Potent Kir4.1 Inhibitor as a Safe and Rapid‐Onset Antidepressant Agent in Mice
Source: Adv Sci (Weinh). 2025 Dec 3;13(9):e09506. doi: 10.1002/advs.202509506 (PMC12903969; doi:10.1002/advs.202509506)
Supplement: Supplementary file 1 — Supporting Information [file ADVS-13-e09506-s002.docx]

**Supporting Information**

**Discovery of a Novel and Potent Kir4.1 Inhibitor as a Safe and Rapid-Onset Antidepressant Agent in Mice**

*Sisi Wang ^1^, Xiaoyu Zhou ^2^, Mengdan Li ^2,4^, Chao Zhang ^1^, Haiyan Xu ^2^, Jingyi He ^2^, Li Zhan ^2^, Yueling Gu ^2^, Hao Gu ^1^, Tianyu Tu ^1^, Hanfang Liu ^1^, Taotao Lu ^1^,* *Yueming Zheng ^2^, Jian Li ^*1,3,5^, Zhaobing Gao ^*2,4,6^ & Yixiang Xu ^*1^*

^1^State Key Laboratory of Bioreactor Engineering, Shanghai Frontiers Science Center of Optogenetic Techniques for Cell Metabolism, Frontiers Science Center for Materiobiology and Dynamic Chemistry, Shanghai Key Laboratory of New Drug Design, School of Pharmacy, East China University of Science and Technology, Shanghai 200237, China.

^2^State Key Laboratory of Drug Research, Shanghai Institute of Materia Medica, Chinese Academy of Sciences, Shanghai 201203, China.

^3^Key Laboratory of Tropical Biological Resources of Ministry of Education, School of Pharmaceutical Sciences, Hainan University, Haikou 570228, China.

^4^School of Pharmacy, Henan University, Kaifeng 475004, China.

^5^Key Laboratory of Xinjiang Phytomedicine Resource and Utilization, Ministry of Education, School of Pharmacy, Shihezi University, Shihezi 832003, China.

^6^Zhongshan Institute for Drug Discovery, Shanghai Institute of Materia Medica, Chinese Academy of Sciences, Zhongshan 528437, China.

Sisi Wang, Xiaoyu Zhou, Mengdan Li contributed equally to this work.

* Corresponding authors: Yixiang Xu (yixxu@ecust.edu.cn), Zhaobing Gao (zbgao@simm.ac.cn), Jian Li (jianli@ecust.edu.cn)

**Table of Contents**

**Supplementary Figures** S3

Figure S1. S3

Figure S2. S3

Figure S3. S4

Figure S4. S5

Figure S5. S5

Figure S6. S6

Figure S7. S6

Figure S8. S7

Figure S9. S7

Figure S10. S8

**Supplementary Tables** S9

Table S1 S9

Table S2 S12

Table S3 S13

**Supplementary Schemes** S14

Scheme S1 S14

Scheme S2 S16

Scheme S3 S20

**Characterization of the compound structure** S23

**NMR spectra-^1^H** S50

**NMR spectra-^13^C** S89

**HRMS spectra** S104

**HPLC traces** S125

**Supplementary Figures**


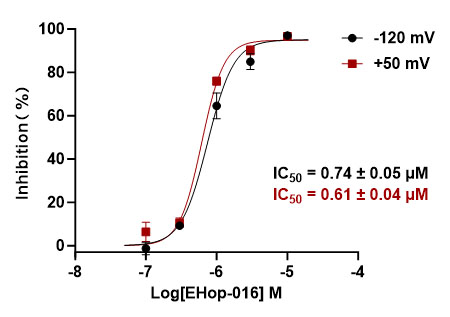


**Figure S1** Concentration-response curves illustrating the inhibition of Kir4.1 currents by EHop-016 in Kir4.1-transfected CHO-K1 cells (n = 2–5). Data are shown as mean ± SEM.

**
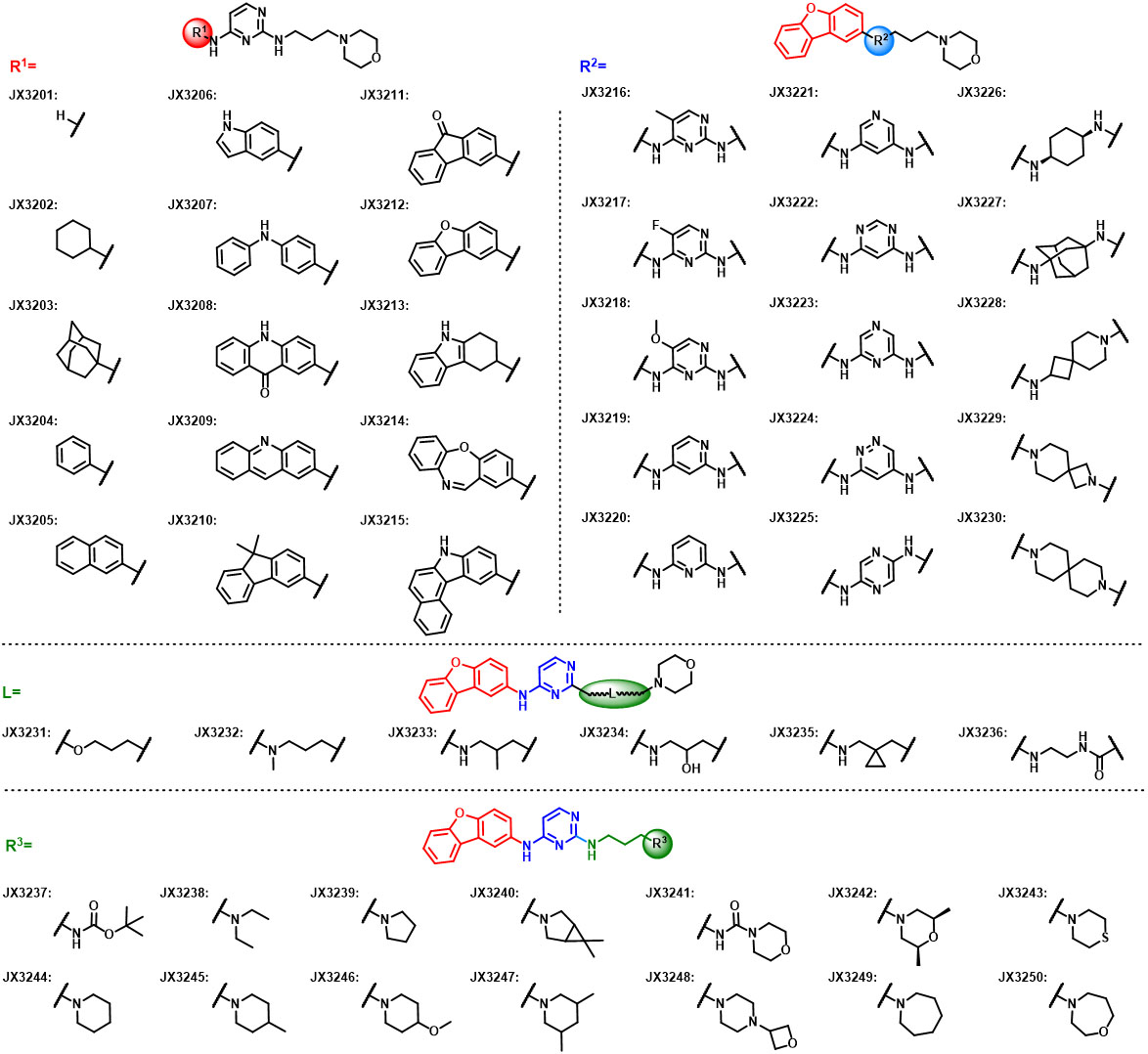
**

**Figure S2** The structures of all synthesized compounds.

**
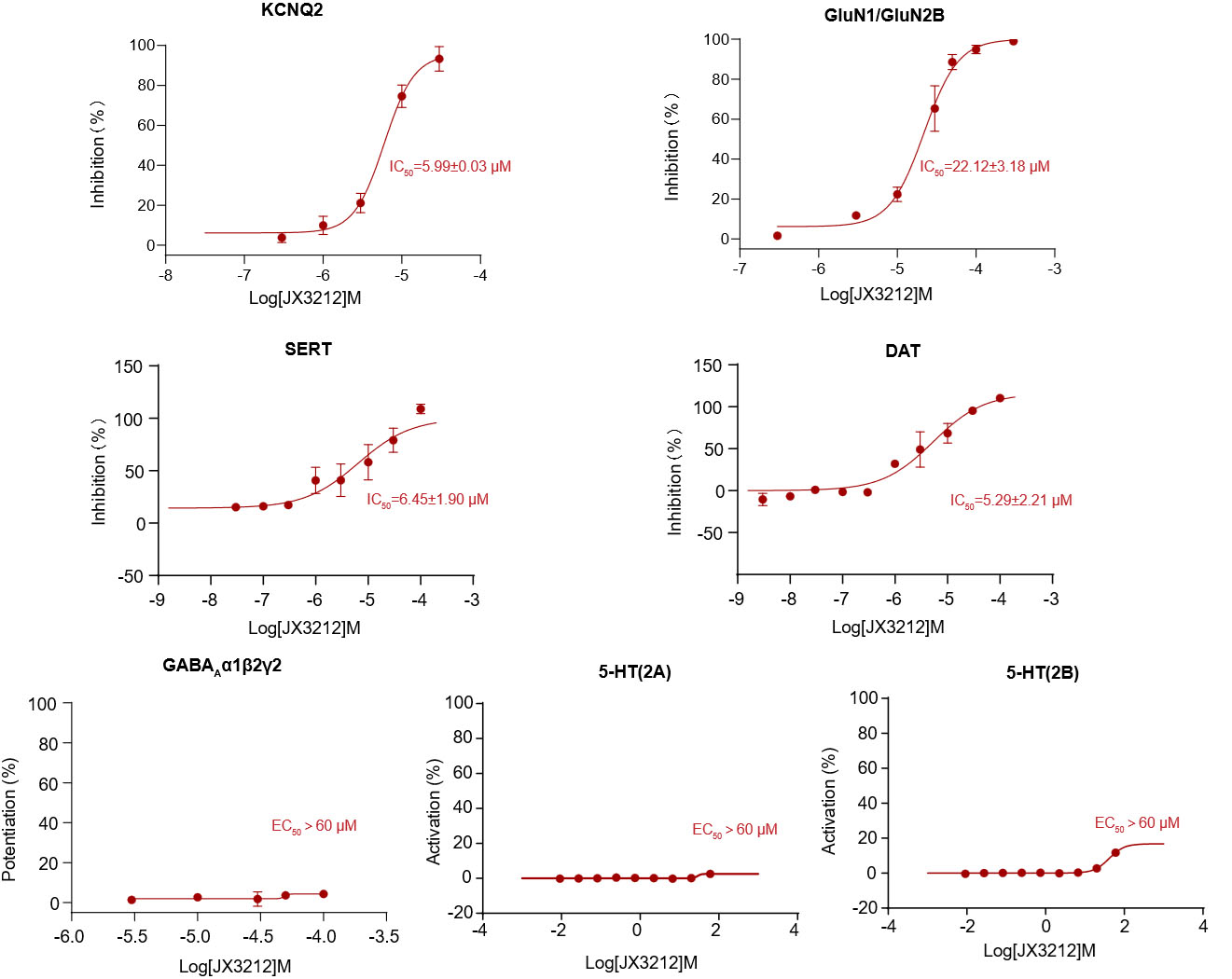
**

**Figure S3** The concentration-response curves for the representative targets. n = 2–4. Data are shown as mean ± SEM.

**
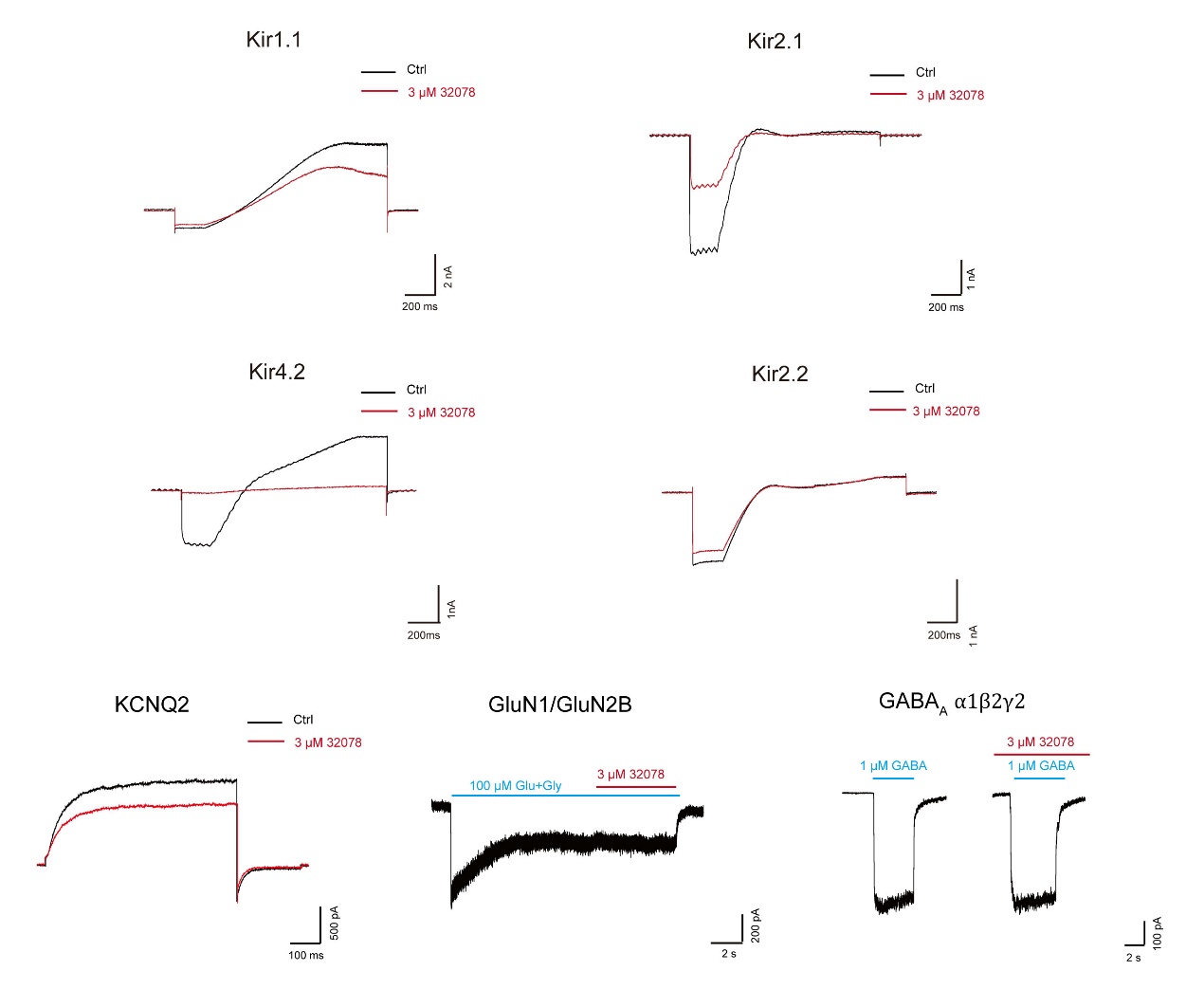
**

**Figure S4** The exemplar current traces for the representative targets.

**
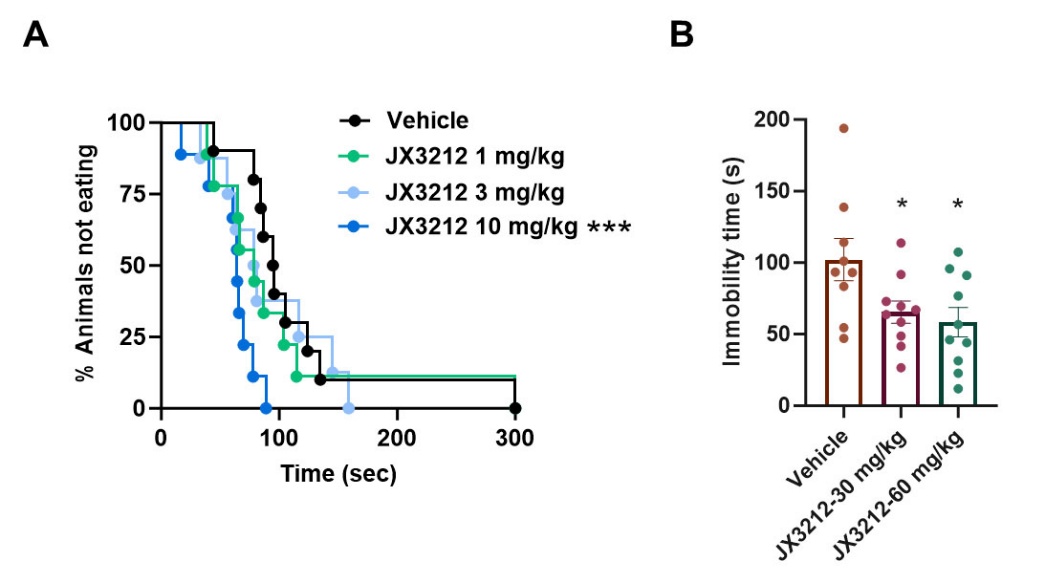
**

**Figure S5** Antidepressant-like efficacy of **JX3212** in NSFT and FST. (A) Dose-dependent antidepressant-like activity of **JX3212** (1/3/10 mg/kg) in the NSFT (n = 8–10). (B) Dose-dependent antidepressant-like activity of **JX3212** (30/60 mg/kg) in the FST (n = 9–10). Two-tailed Student’s *t*-test. Data are shown as mean ± SEM. *p < 0.05, ***p < 0.001 *vs* Vehicle group.

**
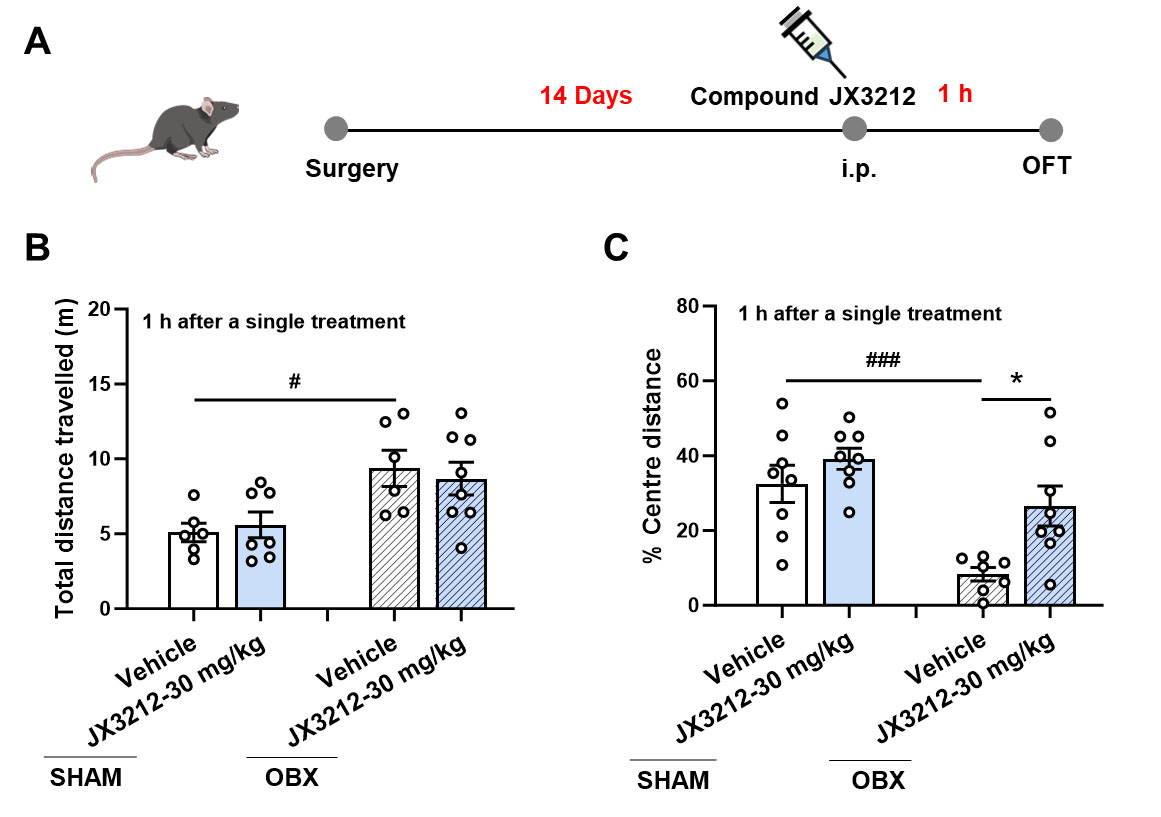
**

**Figure S6** A single dose of **JX3212** protects against depression-like behavior 1 h after administration in OBX model. (A) Establishment of the olfactory bulbectomy (OBX) model. (B) Total distance travelled for different groups of mice (n = 6–8). (C) Percentage of central distance in different groups of mice (n = 7–8). Data are shown as mean ± SEM. *p < 0.05; #p < 0.05; ###p < 0.001; two-way ANOVA with Bonferroni post hoc test.


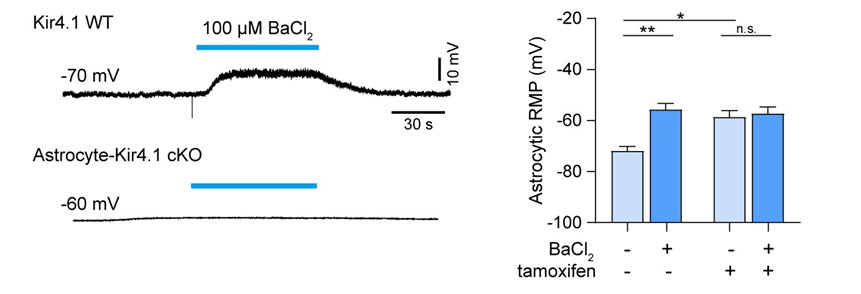


**Figure S7** Conditional knockout of Kir4.1 in astrocytes abolished the effects of BaCl_2_ on resting membrane potentials. (A) The representative RMP traces in response to 100 μM BaCl_2_ treatment from *Aldh1l1*-cre/ERT; *Kcnj10^flox/flox^* astrocytes in the presence (Astrocyte-Kir4.1 cKO) or absence (Kir4.1 WT) of tamoxifen induction. (B) The effects of 100 μM BaCl_2_ on astrocytic RMPs. Data are shown as mean ± SEM. n.s., p > 0.05; *p < 0.05; **p < 0.01; two-way ANOVA with Tukey’s test.


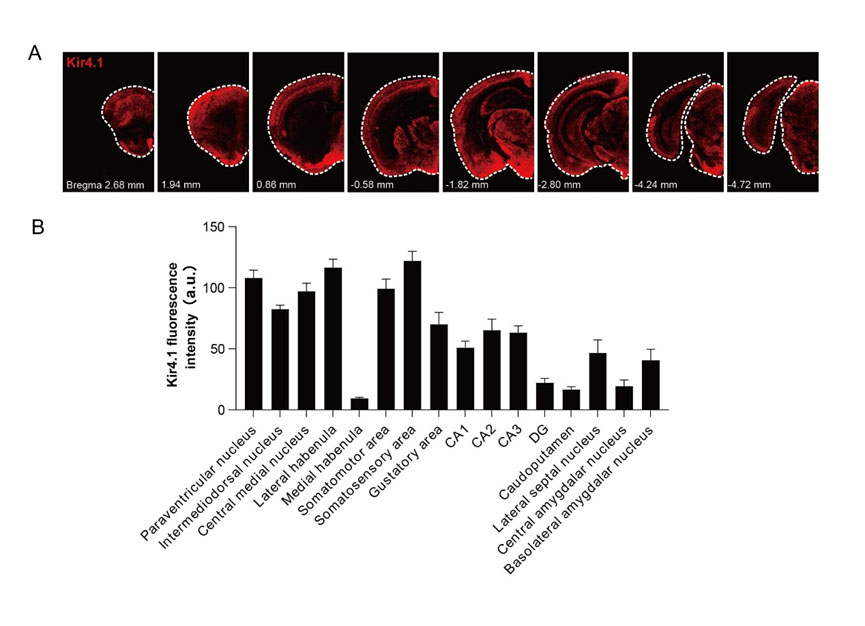


**Figure S8** Distribution of Kir4.1 immunoreactivity in mouse brain (n = 4). Data are shown as mean ± SEM.


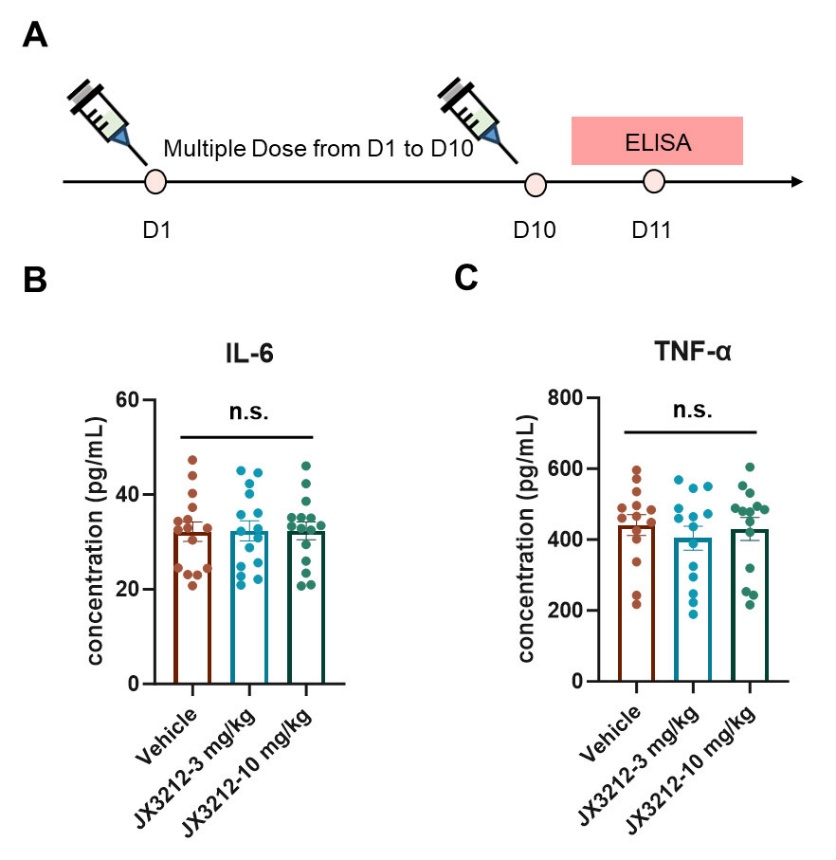


**Figure S9** Evaluation of inflammatory cytokine levels following prolonged administration of **JX3212** (A) Experimental design schematic. (B) IL-6 levels (n = 15). (C) TNF-α levels (n = 14). Two-tailed Student’s *t*-test. The data are shown as mean ± SEM. n.s., p > 0.05 *vs* corresponding Vehicle group.


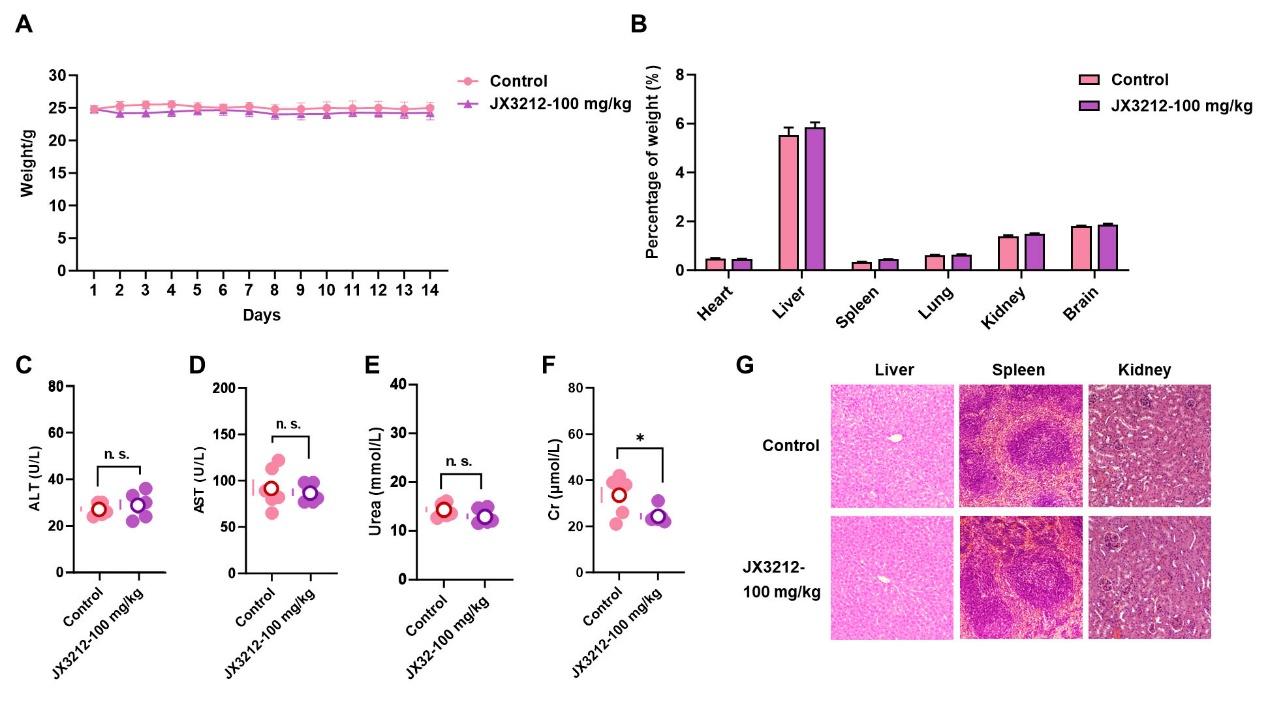


**Figure S10**. Safety studies of **JX3212**. (A) Daily weight of the repeat-dose toxicity. (B)The weight percentage of critical organ of the repeat-dose toxicity after 14 days. Two-way ANOVA with Tukey’s test. (C-F) Biochemical parameters of liver profile and renal profile. AST, aspartate aminotransferase; ALT, alanine aminotransferase; Cr, creatinine. Two-tailed Student’s *t*-test. (G) Representative photomicrographs of HE-staining of liver, spleen and kidney sections (magnification: 400×). n = 6. Data are shown as mean ± SEM. n.s. p > 0.05, *p < 0.05.

**Supplementary Tables**

**Table S1.** Evaluation of Kir4.1 inhibitory activities

| Compd. | Kir4.1 Inhibition ± SD (%)  at 10 μM | | Kir4.1 Inhibition ± SD (%)  at 1 μM | | Kir4.1 Inhibition ± SD (%)  at 0.5 μM | |
| --- | --- | --- | --- | --- | --- | --- |
|  | -120 mV | +50 mV | -120 mV | +50 mV | -120 mV | +50 mV |
| EHop-016 | 96.96 ± 1.86 | 96.57 ± 1.38 | 64.57 ± 5.93 | 75.97 ± 1.81 | 19.03 ± 3.75 | 17.24 ± 2.53 |
| **JX3201** | 4.04 ± 7.66 | 3.02 ± 1.37 | -- | -- | -- | -- |
| **JX3202** | 2.79 ± 2.22 | 6.08 ± 3.06 | -- | -- | -- | -- |
| **JX3203** | 31.27 ± 3.29 | 64.79 ± 0.85 | -- | -- | -- | -- |
| **JX3204** | 0.47 ± 2.29 | 5.73 ± 2.33 | -- | -- | -- | -- |
| **JX3205** | 8.88 ± 10.21 | 36.46 ± 10.25 | -- | -- | -- | -- |
| **JX3206** | 25.94 ± 1.96 | 38.41 ± 5.37 | -- | -- | -- | -- |
| **JX3207** | 92.47 ± 0.40 | 93.00 ± 1.73 | 27.52 ± 8.17 | 30.53 ± 6.87 | -- | -- |
| **JX3208** | 2.21 ± 9.84 | 1.43 ± 3.10 | -- | -- | -- | -- |
| **JX3209** | 86.37 ± 4.85 | 85.93 ± 3.90 | 5.58 ± 4.14 | -0.09 ± 6.09 | -- | -- |
| **JX3210** | 98.69 ± 0.66 | 93.19 ± 3.42 | 42.74 ± 2.34 | 62.05 ± 4.20 | -- | -- |
| **JX3211** | 80.97 ± 2.36 | 89.18 ± 1.72 | 21.32 ± 8.61 | 28.82 ±5.45 | -- | -- |
| **JX3212** | 97.33 ± 1.01 | 96.00 ± 1.52 | 84.54 ± 3.09 | 82.46 ± 5.51 | 73.16 ± 3.59 | 82.79 ± 1.51 |
| **JX3213** | 65.07 ± 4.67 | 79.09 ± 3.70 | 41.24 ± 4.03 | 54.96 ± 3.87 | -- | -- |
| **JX3214** | 94.52 ± 0.70 | 93.62 ± 4.14 | 56.63 ± 3.90 | 60.42 ± 6.32 | -- | -- |
| **JX3215** | 89.85 ± 1.62 | 95.83 ± 0.42 | 70.96 ± 2.19 | 79.32 ± 3.86 | 30.29 ± 5.33 | 39.48 ± 5.16 |
| **JX3216** | 91.97 ± 1.98 | 97.02 ± 0.67 | 9.55 ± 3.62 | 16.75 ± 8.93 | -- | -- |
| **JX3217** | 7.77 ± 6.65 | 11.92 ± 10.14 | -- | -- | -- | -- |
| **JX3218** | 74.34 ± 4.51 | 86.77 ± 1.76 | 3.97 ± 2.03 | 1.15 ± 2.32 | -- | -- |
| **JX3219** | 92.59 ± 5.66 | 97.44 ± 0.60 | 78.73 ± 8.16 | 86.07 ± 3.36 | 40.16 ± 4.35 | 49.61 ± 6.47 |
| **JX3220** | 3.98 ± 1.60 | 2.61 ± 1.87 | -- | -- | -- | -- |
| **JX3221** | 90.38 ± 5.72 | 96.57 ± 1.14 | -4.84 ± 3.28 | -2.41 ± 3.20 | -- | -- |
| **JX3222** | 71.12 ± 6.03 | 87.35 ± 6.40 | -0.94 ± 6.65 | 5.55 ± 2.74 | -- | -- |
| **JX3223** | 6.62 ± 0.29 | 7.01 ± 4.11 | -- | -- | -- | -- |
| **JX3224** | 87.90 ± 2.73 | 96.66 ± 1.05 | 11.30 ± 5.07 | 29.13 ± 11.80 | -- | -- |
| **JX3225** | 94.26 ± 2.60 | 96.89 ± 1.28 | 8.36 ± 3.94 | 3.73 ± 5.09 | -- | -- |
| **JX3226** | 86.98 ± 0.69 | 94.03 ± 1.98 | 6.91 ± 6.13 | 3.35 ± 4.46 | -- | -- |
| **JX3227** | 64.42 ± 3.05 | 88.04 ± 4.11 | 7.11 ± 1.17 | 9.02 ± 5.70 | -- | -- |
| **JX3228** | 17.10 ± 6.30 | 72.97 ± 3.72 | 0.88 ± 2.32 | 18.35 ± 3.79 | -- | -- |
| **JX3229** | 4.90 ± 7.34 | 26.81 ± 9.64 | -- | -- | -- | -- |
| **JX3230** | 15.64 ± 0.73 | 63.30 ± 6.48 | -- | -- | -- | -- |
| **JX3231** | 90.55 ± 2.35 | 96.28 ± 1.23 | 4.14 ± 5.52 | 7.39 ± 3.45 | -- | -- |
| **JX3232** | 93.16 ± 1.48 | 96.94 ± 0.69 | -2.25 ± 0.47 | 6.81 ± 4.50 | -- | -- |
| **JX3233** | 97.20 ± 1.69 | 92.25 ± 1.15 | 2.58 ± 3.95 | 4.34 ± 8.43 | -- | -- |
| **JX3234** | 88.43 ± 2.42 | 94.83 ± 0.75 | 5.91 ± 6.31 | 11.25 ± 5.26 | -- | -- |
| **JX3235** | 85.37 ± 5.46 | 92.37 ± 3.68 | 6.83 ± 3.85 | 10.78 ± 2.21 | -- | -- |
| **JX3236** | 70.77 ± 2.50 | 89.24 ± 0.50 | -4.25 ± 7.82 | 5.00 ± 5.21 | -- | -- |
| **JX3237** | 73.84 ± 0.93 | 89.10 ± 3.59 | 7.65 ± 4.73 | 12.49 ± 7.25 | -- | -- |
| **JX3238** | 93.07 ± 2.15 | 98.50 ± 0.23 | 2.21 ± 3.14 | 27.00 ± 12.31 | -- | -- |
| **JX3239** | 90.89 ± 4.50 | 96.01 ± 2.25 | 36.41 ± 5.31 | 53.19 ± 3.51 | -- | -- |
| **JX3240** | 97.35 ± 0.65 | 97.46 ± 0.83 | 84.69 ± 4.13 | 89.95 ± 1.44 | -0.29 ± 1.05 | -0.92 ± 3.58 |
| **JX3241** | 59.53 ± 3.66 | 84.18 ± 2.69 | 1.97 ± 2.44 | 4.09 ± 2.08 | -- | -- |
| **JX3242** | 94.20 ± 1.77 | 97.51 ± 1.92 | -3.38 ± 9.30 | -0.93 ± 7.76 | -- | -- |
| **JX3243** | 98.60 ± 0.56 | 98.74 ± 0.36 | 0.17 ± 1.63 | -1.39 ± 1.64 | -- | -- |
| **JX3244** | 97.01 ± 1.09 | 98.66 ± 0.25 | 74.53 ± 6.63 | 85.39 ± 3.98 | 62.99 ± 5.95 | 74.88 ± 2.12 |
| **JX3245** | 95.46 ± 2.80 | 96.84 ± 0.61 | 92.48 ± 0.80 | 96.35 ± 0.66 | 9.76 ± 2.44 | 21.40 ± 6.07 |
| **JX3246** | 89.45 ± 2.33 | 91.30 ± 3.50 | 55.38 ± 3.73 | 73.02 ± 5.77 | -- | -- |
| **JX3247** | 94.20 ± 2.26 | 97.75 ± 0.84 | 66.64 ± 2.75 | 80.39 ± 8.36 | -- | -- |
| **JX3248** | 81.52 ± 2.87 | 97.46 ± 0.59 | 11.46 ± 6.63 | 21.94 ± 8.36 | -- | -- |
| **JX3249** | 94.94 ± 1.88 | 98.49 ± 0.04 | 82.25 ± 2.24 | 92.39 ± 1.44 | 46.37 ± 8.18 | 56.02 ± 5.59 |
| **JX3250** | 88.32 ± 4.59 | 97.39 ± 1.14 | 30.40 ± 8.64 | 43.95 ± 9.96 | -- | -- |

**Table S2.** Evaluation of breast cancer cell inhibitory activities of all compounds

| Compd. | IC_50_ ± SD (μM) for  MDA-MB-468 Inhibition | Compd. | IC_50_ ± SD (μM) for  MDA-MB-468 Inhibition |
| --- | --- | --- | --- |
| EHop-016 | 5.98 ± 0.67 | **JX3226** | 19.47 ± 1.64 |
| **JX3201** | > 50 | **JX3227** | > 50 |
| **JX3202** | 21.06 ± 2.08 | **JX3228** | 11.23 ± 0.74 |
| **JX3203** | 22.15 ± 5.05 | **JX3229** | 29.49 ± 1.09 |
| **JX3204** | > 50 | **JX3230** | > 50 |
| **JX3205** | 16.90 ± 1.65 | **JX3231** | 19.69 ± 0.50 |
| **JX3206** | > 50 | **JX3232** | 6.77 ± 0.68 |
| **JX3207** | 12.26 ± 1.32 | **JX3233** | 10.44 ± 0.01 |
| **JX3208** | 16.76 ± 0.51 | **JX3234** | 17.08 ± 0.83 |
| **JX3209** | 38.35 ± 4.21 | **JX3235** | 11.33 ± 0.32 |
| **JX3210** | 2.66 ± 0.51 | **JX3236** | 21.95 ± 1.01 |
| **JX3211** | 29.79 ± 0.50 | **JX3237** | 4.84 ± 0.06 |
| **JX3212** | **13.74 ± 0.61** | **JX3238** | 10.34 ± 3.80 |
| **JX3213** | 17.74 ± 3.05 | **JX3239** | 11.05 ± 0.81 |
| **JX3214** | 4.64 ± 0.41 | **JX3240** | 5.37 ± 0.22 |
| **JX3215** | 6.72 ± 0.28 | **JX3241** | 21.09 ± 1.53 |
| **JX3216** | 17.94 ± 0.65 | **JX3242** | 5.38 ± 0.11 |
| **JX3217** | > 50 | **JX3243** | 7.11 ± 0.62 |
| **JX3218** | 13.27 ± 0.30 | **JX3244** | 6.86 ± 0.23 |
| **JX3219** | 8.74 ± 0.36 | **JX3245** | 5.95 ± 0.04 |
| **JX3220** | 8.74 ± 0.36 | **JX3246** | 9.97 ± 0.62 |
| **JX3221** | 21.20 ± 1.06 | **JX3247** | 6.03 ± 0.24 |
| **JX3222** | 44.04 ± 1.48 | **JX3248** | > 50 |
| **JX3223** | 23.02 ± 3.41 | **JX3249** | 5.10 ± 0.21 |
| **JX3224** | 21.60 ± 0.80 | **JX3250** | 12.22 ± 0.87 |
| **JX3225** | 31.64 ± 5.27 |  |  |

| Compd. | Species | Dose | Plasma Calculated  Concentration (ng/mL) | Kidney Calculated Concentration (ng/mL) | Kidney/Plasma | IC_50_ (μM) for  hERG Inhibition |
| --- | --- | --- | --- | --- | --- | --- |
| **JX3212** | Mice  (N = 3) | 10 mg/kg  (*i.p.*) | 234.0 ± 14.4 | 18433.0 ± 680.7 | 78.87 ± 2.74 | 1.02 |

**Table S3.** Renal distribution profile and hERG inhibition of **JX3212**

**Supplementary Schemes**

**Scheme S1** Synthetic route of derivatives **JX3201–15**. (a) DIPEA, sec-butanol, microwave, 150 ℃, 2 h; (b) tBuONa, Pd_2_(dba)_3_, XPhos, tolune, 120 °C, 12 h; (c) tBuONa, BrettPhos Pd G3, BrettPhos, 1,4-Dioxane, 120 °C, 12 h; (d) DIPEA, 2-Propanol, 80 °C, 2 h.

**General synthesis of target compounds JX3201-10, JX3212-14**

To a mixture containing various substituted anilines (1.0 eq) and 2,4-dichloropyrimidine (1.5 eq) in isopropanol, DIPEA (3.0 eq) was introduced. This blend was stirred at 80 °C for a duration of 12 h. Upon completion of the reaction, the resulting mixture was concentrated under reduced pressure and subsequently extracted with water and ethyl acetate, followed by treatment with brine. The organic phase was dried over Na_2_SO_4_, filtered, and concentrated *in vacuo* to yield the desired crude products. These products were purified through silica gel column chromatography, resulting in the corresponding intermediates **JX3202a-10a**, and **JX3212a-14a**. For the next step, a solution of intermediates **JX3202a-10a**, **JX3212a-14a** and starting material 2-chloropyrimidin-4-amine (1.0 eq) in 2-butanol was treated with 3-morpholinopropan-1-amine (2.0 eq) and DIPEA (3.0 eq). The reaction mixture was then heated to 150 °C for 2 h using microwave conditions, with the pressure set to 10 bar. Following the reaction's completion, the solution was concentrated under reduced pressure. The resulting crude oil was extracted with water and ethyl acetate, and the combined organic layers were washed with brine, dried using anhydrous Na_2_SO_4_, filtered, and concentrated *in vacuo*. The final residue was purified using flash column chromatography on silica gel, yielding compounds **JX3202-10**, **JX3212-14** and **JX3201** successively.

**Synthesis of target compound JX3211**

Compound **JX3201** (1.2 eq), 3-bromo-9*H*-fluoren-9-one (1.0 eq), sodium tert-butoxide (2.0 eq), Pd_2_(dba)_3_ (0.05 eq), and XPhos (0.1 eq) were dissolved in toluene and then stirred at 120 °C for 12 h under a nitrogen atmosphere. Once the reaction was completed, the reaction product was washed three times with ethyl acetate and water, and the obtained organic layer was dried over anhydrous Na_2_SO_4_ and then dried under reduced pressure. The obtained product was subjected to column chromatography to obtain target compound **JX3211**.

**Synthesis of target compound JX3215**

Compound **JX3201** (1.2 eq), 10-bromo-7*H*-benzo[c]carbazole (1.0 eq), sodium tert-butoxide (2.0 eq), BrettPhos Pd G3 (0.05 eq), and BrettPhos (0.1 eq) were dissolved in 1,4-Dioxane and then stirred at 120 °C for 12 h under a nitrogen atmosphere. Once the reaction was completed, the reaction product was washed three times with ethyl acetate and water, and the obtained organic layer was dried over anhydrous Na_2_SO_4_ and then dried under reduced pressure. The obtained product was subjected to column chromatography to obtain target compound **JX3215**.

**Scheme S2** Synthetic route of derivatives **JX3216–30**. (a) DIPEA, 2-Propanol, 80 °C, 2 h; (b) DIPEA, sec-butanol, microwave, 150 ℃, 2 h; (c) KN(SiMe_3_)_2_, 1,4-Dioxane, r.t., 2 h; (d) tBuONa, Pd_2_(dba)_3_, BINAP, tolune, 120 °C, 12 h; (e) K_2_CO_3_, Pd_2_(dba)_3_, XantPhos, 1,4-Dioxane, 120 °C, 12 h; (f) K_2_CO_3_, 1,4-Dioxane, 80 °C, 5 h; (g) Cs_2_CO_3_, Pd_2_(dba)_3_, XantPhos, tolune, 120 °C, 12 h; (h) tBuONa, BrettPhos Pd G3, BrettPhos, 1,4-Dioxane, 120 °C, 12 h; (i) K_2_CO_3_, DMF, 80 °C, 8 h; (j) CF_3_COOH, DCM, r.t., 0.5 h; (k) tBuONa, Pd_2_(dba)_3_, XantPhos, 1,4-Dioxane, 120 °C, 12 h; (l) DIPEA, ACN, 80 °C, 5 h.

**General synthesis of target compounds JX3216-18**

Intermediates **JX3216a-18a** were obtained from dibenzo[b,d]furan-2-amine and differently substituted 2,4-dichloro-pyrimidines by using similar synthetic routes as those of **JX3202a**. Compounds **JX3216-18** were prepared following the general procedure of **JX3202**.

**Synthesis of target compound JX3219**

2-chloropyridin-4-amine (1.2 eq), 2-bromodibenzo[b,d]furan (1.0 eq), Cs_2_CO_3_ (2.0 eq), Pd_2_(dba)_3_ (0.05 eq), and XantPhos (0.1 eq) were dissolved in toluene and then stirred at 120°C for 12 h under a nitrogen atmosphere. Once the reaction was completed, the reaction product was washed three times with ethyl acetate and water, and the obtained organic layer was dried over anhydrous Na_2_SO_4_ and then dried under reduced pressure. The obtained product was subjected to column chromatography to obtain target compound **JX3219a**. Then **JX3219a** (1.0 eq), 3-morpholinopropan-1-amine (1.2 eq), tBuONa (2.0 eq), BrettPhos Pd G3 (0.05 eq), and BrettPhos (0.1 eq) were dissolved in 1,4-Dioxane and then stirred at 120 °C for 12 h under a nitrogen atmosphere. The post-processing was the same as above to obtain target compound **JX3219**.

**Synthesis of target compound JX3220**

Dibenzo[b,d]furan-2-amine (1.0 eq), 2,6-dibromopyridine (1.2 eq), and potassium bis(trimethylsilyl)amide (1.5 eq) were dissolved in 1,4-Dioxane and then stirred at room temperature for 2 h. Once the reaction was completed, the reaction product was washed with ethyl acetate and water, and the obtained organic layer was concentrated, and subjected to column chromatography to obtain intermediate **JX3220a**. Then **JX3220a** (1.0 eq), 3-morpholinopropan-1-amine (1.2 eq), tBuONa (2.0 eq), Pd_2_(dba)_3_ (0.05 eq), and BINAP (0.1 eq) were dissolved in toluene and then stirred at 120 °C for 12 h under a nitrogen atmosphere. The post-processing was the same as above to obtain target compound **JX3220**.

**Synthesis of target compound JX3221**

Intermediate **JX3221a** were obtained from 3,5-dibromopyridine and 3-morpholinopropan-1-amine by using similar synthetic routes as those of **JX3220**. Then **JX3220a** (1.0 eq), dibenzo[b,d]furan-2-amine (1.2 eq), tBuONa (2 eq), Pd_2_(dba)_3_ (0.05 eq), and XantPhos (0.1 eq) were dissolved in 1,4-Dioxane and then stirred at 120 °C for 12 h under a nitrogen atmosphere. The post-processing was the same as above to obtain target compound **JX3220**.

**Synthesis of target compound JX3222**

Intermediate **JX3222a** were obtained from dibenzo[b,d]furan-2-amine and 4,6-dichloropyrimidine by using similar synthetic routes as those of **JX3216a**. Compound **JX3222** were prepared following the general procedure of **JX3216**.

**Synthesis of target compound JX3223**

Dibenzo[b,d]furan-2-amine (1.0 eq), 2,6-dichloropyrazine (1.2 eq), K_2_CO_3_ (2.0 eq), Pd_2_(dba)_3_ (0.05 eq), and XantPhos (0.1 eq) were dissolved in 1,4-Dioxane and then stirred at 120 °C for 12 h under a nitrogen atmosphere. Once the reaction was completed, the reaction product was washed three times with ethyl acetate and water, and the obtained organic layer was dried over anhydrous Na_2_SO_4_ and then dried under reduced pressure. The obtained product was subjected to column chromatography to obtain target compound **JX3223a**. Then **JX3223a** (1.0 eq), 3-morpholinopropan-1-amine (1.2 eq), and K_2_CO_3_ (2.0 eq) were dissolved in 1,4-Dioxane and then stirred at 80 °C for 5 h. The post-processing was the same as above to obtain target compound **JX3223**.

**Synthesis of target compound JX3224**

Intermediate **JX3224a** were obtained from 3,5-dichloropyridazine and 3-morpholinopropan-1-amine by using similar synthetic routes as those of **JX3219**. Compound **JX3224** obtained from **JX3224a** and dibenzo[b,d]furan-2-amine by using similar synthetic routes as those of **JX3219a**.

**Synthesis of target compound JX3225**

3-morpholinopropan-1-amine (1.0 eq), 2,5-dibromopyrazine (1.2 eq) and DIPEA (3.0 eq) were dissolved in acetonitrile and then stirred at 80 °C for 5 h. Once the reaction was completed, the reaction product was washed three times with ethyl acetate and water, and the obtained organic layer was dried over anhydrous Na_2_SO_4_ and then dried under reduced pressure. The obtained product was subjected to column chromatography to obtain target compound **JX3225a**. Compound **JX3225** obtained from **JX3225a** and dibenzo[b,d]furan-2-amine by using similar synthetic routes as those of **JX3219a**.

**General synthesis of target compounds JX3226-30**

Intermediates **JX3226a-30a** were obtained from dibenzo[b,d]furan-2-amine and different primary amines by using similar synthetic routes as those of **JX3219**. 4-(3-bromopropyl)morpholine (1.0 eq), **JX3227a** (1.2 eq) and K_2_CO_3_ (3.0 eq) were dissolved in DMF and then stirred at 80 °C for 8 h. When the reaction was completed, the reaction product was washed three times with ethyl acetate and water, and the obtained organic layer was dried over anhydrous Na_2_SO_4_ and then dried under reduced pressure. The obtained product was subjected to column chromatography to obtain target compound **JX3227**. Intermediates **JX3226a** and **JX3228-30a** were deprotected under 20% trifluoroacetic acid conditions and then reacted with 4-(3-bromopropyl)morpholine by using similar synthetic routes as those of **JX3227**.

**Scheme S3** Synthetic route of derivatives **JX3231–50**. (a) Na, n-butanol, 100 °C, 6 h; (b) DIPEA, sec-butanol, microwave, 150 ℃, 2 h; (c) CF_3_COOH, DCM, r.t., 0.5 h; (d) NEt_3_, DCM, r.t., 12 h; (e) K_2_CO_3_, acetone, 50 °C, 24 h; (f) NEt_3_, NaI, THF, 80 °C, 24 h; (g) hydrazine hydrate, EtOH, r.t., 24 h.

**Synthesis of target compound JX3231**

After sodium (20.0 eq) was dissolved in n-butanol, **JX3212a** (1.0 eq) and 3-morpholinopropan-1-ol (1.0 eq) was added and the mixture was stirred at 100 °C for 6 h. After the reaction was finished, water was added thereto and the mixture was stirred for 0.5 h, neutralized with concentrated hydrochloric acid, and subsequently extracted with water and ethyl acetate, followed by treatment with brine. The organic phase was dried over Na_2_SO_4_, filtered, and concentrated *in vacuo* to yield the desired crude products. These products were purified through silica gel column chromatography, resulting in the compound **JX3231**.

**General synthesis of target compounds JX3232, JX3236-37, JX3241**

Compounds **JX3232**, **JX3237** and intermediates **JX3236** were obtained from **JX3212a** and differently substituted amines by using similar synthetic routes as those of **JX3212**. Intermediates **JX3236a** and **JX3237** were then deprotected under 20% trifluoroacetic acid conditions and reacted with morpholine-4-carbonyl chloride (1.0 eq), triethylamine (6.0 eq) in DCM for 12 h at room temperature. The resulting solution was poured into water, and then extracted with ethyl acetate, dried over Na_2_SO_4_, and concentrated under vacuum to afford corresponding compounds **JX3236** and **JX3241**.

**General synthesis of target compounds JX3233-35**

To a stirred solution of isoindoline-1,3-dione (1.0 eq) in acetone, differently substituted 1,3-dibromo propane (3.0 eq), K_2_CO_3_ (3.0 eq) were added. The reaction mixture was stirred at 50 °C for 24 h. After completion of the reaction, the resulting solution was poured into ice cold water, which was then extracted with ethyl acetate, dried over Na_2_SO_4_, and concentrated under vacuum to afford intermediates **JX3233a-35a**. To a stirred solution of **JX3233a-35a** (1.0 eq) in THF, morpholine (1.1 eq), triethylamine (1.0 eq) and NaI (1.0 eq) were added. The reaction mixture was stirred at 80 °C for 24 h. The resulting solution was poured into ice cold water, which was then extracted with ethyl acetate, dried over Na_2_SO_4_, and concentrated under vacuum to afford intermediates **JX3233b-35b**. To a stirred solution of **JX3233b-35b** in EtOH, hydrazine hydrate (6.0 eq) was added at room temperature. The reaction was continued for 24 h. The precipitate obtained was filtered, and the filtrate was evaporated under vacuum. DCM was added to the residue obtained and washed with water. The organic layer was separated, dried over Na_2_SO_4_, and concentrated under vacuum to yield **JX3233c-35c**. Compounds **JX3233-35** were prepared following the general procedure of **JX3232**.

**General synthesis of target compounds JX3238-40, JX3242-50**

Intermediates **JX3238a-40a** and **JX3242a-50a** were obtained from starting material 2-(3-bromopropyl)isoindoline-1,3-dione and different secondary amines by using similar synthetic routes as those of **JX3233b-35b**. Intermediates **JX3238b-40b** and **JX3242b-50b** were obtained following the general procedure of **JX3233c-35c**. Compounds **JX3238-40** and **JX3242-50** were prepared following the general procedure of **JX3233-35**.

**Characterization of the compound structure**

*N^2^-(3-morpholinopropyl)pyrimidine-2,4-diamine* (**JX3201**)

White solid, 50% yield. **^1^H NMR (400 MHz, DMSO-*d*_6_)** *δ* 7.64 (d, *J* = 5.6 Hz, 1H), 6.25 (d, *J* = 24.8 Hz, 3H), 5.66 (d, *J* = 5.6 Hz, 1H), 3.56 (t, *J* = 4.7 Hz, 4H), 3.19 (q, *J* = 6.6 Hz, 2H), 2.39–2.22 (m, 6H), 1.62 (p, *J* = 7.1 Hz, 2H). **^13^C NMR (151 MHz, DMSO-*d*_6_)** *δ* 164.31, 162.78, 156.03, 95.24, 66.68, 56.71, 53.87, 39.52, 26.60. **HRMS (EI)** m/z calcd C_11_H_19_N_5_O [M]^+^ 237.1590, found 237.1593. **HPLC purity:** 99.2% (RT = 5.676 min).

*2-chloro-N-cyclohexylpyrimidin-4-amine* (**JX3202a**)

White solid, 70% yield. **^1^H NMR (400 MHz, DMSO-*d*_6_)** *δ* 7.82 (dd, *J* = 25.2, 6.8 Hz, 2H), 6.40 (d, *J* = 5.9 Hz, 1H), 3.75 (d, *J* = 10.3 Hz, 1H), 1.89–1.79 (m, 2H), 1.70 (dt, *J* = 12.8, 3.8 Hz, 2H), 1.58 (dt, *J* = 12.7, 3.8 Hz, 1H), 1.39–1.10 (m, 5H).

*N^4^-cyclohexyl-N^2^-(3-morpholinopropyl)pyrimidine-2,4-diamine* (**JX3202**)

White solid, 36% yield. **^1^H NMR (400 MHz, DMSO-*d*_6_)** *δ* 7.58 (d, *J* = 5.7 Hz, 1H), 6.64 (s, 1H), 6.32 (d, *J* = 6.0 Hz, 1H), 5.65 (d, *J* = 5.8 Hz, 1H), 3.77–3.66 (m, 1H), 3.56 (t, *J* = 4.6 Hz, 4H), 3.19 (q, *J* = 6.6 Hz, 2H), 2.37–2.24 (m, 6H), 1.91–1.80 (m, 2H), 1.76–1.52 (m, 5H), 1.39–1.10 (m, 5H). **^13^C NMR (151 MHz, DMSO-*d*_6_)** *δ* 162.66, 162.24, 155.13, 95.84, 66.71, 56.87, 53.87, 48.59, 40.53, 33.03, 26.72, 25.87, 25.21. **HRMS (EI)** m/z calcd C_17_H_29_N_5_O [M]^+^ 319.2372, found 319.2378. **HPLC purity:** 99.3% (RT = 7.807 min).

*N-((1s,3s)-adamantan-1-yl)-2-chloropyrimidin-4-amine* (**JX3203a**)

White solid, 75% yield. **^1^H NMR (400 MHz, DMSO-*d*_6_)** *δ* 7.84 (d, *J* = 6.0 Hz, 1H), 7.47 (s, 1H), 6.45 (d, *J* = 5.9 Hz, 1H), 2.05 (d, *J* = 5.0 Hz, 9H), 1.65 (t, *J* = 2.6 Hz, 6H).

*N^4^-((1s,3s)-adamantan-1-yl)-N^2^-(3-morpholinopropyl)pyrimidine-2,4-diamine* (**JX3203**)

White solid, 52% yield. **^1^H NMR (400 MHz, DMSO-*d*_6_)** *δ* 7.52 (d, *J* = 5.8 Hz, 1H), 6.40–6.30 (m, 2H), 5.67 (d, *J* = 5.8 Hz, 1H), 3.55 (t, *J* = 4.6 Hz, 4H), 3.21 (q, *J* = 6.6 Hz, 2H), 2.30 (t, *J* = 7.2 Hz, 6H), 2.08 (d, *J* = 2.9 Hz, 6H), 2.04 (d, *J* = 3.2 Hz, 3H), 1.65 (t, *J* = 3.3 Hz, 8H). **^13^C NMR (151 MHz, DMSO-*d*_6_)** *δ* 162.89, 161.97, 154.78, 154.73, 66.69, 56.93, 55.40, 53.90, 51.43, 41.73, 40.54, 36.63, 29.46. **HRMS (EI)** m/z calcd C_21_H_33_N_5_O [M]^+^ 371.2685, found 371.2688. **HPLC purity:** 98.6% (RT = 4.908 min).

*2-chloro-N-phenylpyrimidin-4-amine* (**JX3204a**)

White solid, 65% yield. **^1^H NMR (400 MHz, DMSO-*d*_6_)** *δ* 10.02 (s, 1H), 8.16 (d, *J* = 5.9 Hz, 1H), 7.58 (d, *J* = 7.9 Hz, 2H), 7.38 (dd, *J* = 8.5, 7.3 Hz, 2H), 7.17–7.03 (m, 1H), 6.76 (d, *J* = 5.9 Hz, 1H).

*N^2^-(3-morpholinopropyl)-N^4^-phenylpyrimidine-2,4-diamine* (**JX3204**)

White solid, 22% yield. **^1^H NMR (400 MHz, DMSO-*d*_6_)** *δ* 9.10 (s, 1H), 7.83 (d, *J* = 5.7 Hz, 1H), 7.76–7.69 (m, 2H), 7.33–7.18 (m, 2H), 6.94 (tt, *J* = 7.4, 1.2 Hz, 1H), 6.78 (t, *J* = 5.7 Hz, 1H), 5.97 (d, *J* = 5.7 Hz, 1H), 3.56 (t, *J* = 4.5 Hz, 4H), 3.28 (q, *J* = 6.6 Hz, 2H), 2.39–2.24 (m, 6H), 1.70 (q, *J* = 7.1 Hz, 2H). **^13^C NMR (151 MHz, DMSO-*d*_6_)** *δ* 162.47, 161.01, 156.66, 141.16, 128.98, 121.81, 119.64, 96.23, 66.70, 56.82, 53.88, 40.52, 26.49. **HRMS (ESI)** m/z calcd C_17_H_23_N_5_O [M+H]^+^ 314.1981, found 314.1982. **HPLC purity:** 97.7% (RT = 7.077 min).

*2-chloro-N-(naphthalen-2-yl)pyrimidin-4-amine* (**JX3205a**)

White solid, 57% yield. **^1^H NMR (400 MHz, DMSO-*d*_6_)** *δ* 10.27 (s, 1H), 8.25–8.16 (m, 2H), 7.97–7.79 (m, 3H), 7.65 (dd, *J* = 9.1, 2.2 Hz, 1H), 7.47 (dt, *J* = 27.5, 7.3 Hz, 2H), 6.87 (d, *J* = 5.9 Hz, 1H).

*N^2^-(3-morpholinopropyl)-N^4^-(naphthalen-2-yl)pyrimidine-2,4-diamine* (**JX3205**)

White solid, 33% yield. **^1^H NMR (400 MHz, DMSO-*d*_6_)** *δ* 9.37 (s, 1H), 8.61 (s, 1H), 7.92–7.74 (m, 4H), 7.64 (d, *J* = 8.3 Hz, 1H), 7.44 (t, *J* = 7.5 Hz, 1H), 7.34 (t, *J* = 7.4 Hz, 1H), 6.89 (t, *J* = 5.7 Hz, 1H), 6.07 (d, *J* = 5.7 Hz, 1H), 3.60–3.51 (m, 4H), 3.40–3.33 (m, 2H), 2.37 (dd, *J* = 14.5, 7.0 Hz, 6H), 1.80–1.70 (m, 2H). **^13^C NMR (151 MHz, DMSO-*d*_6_)** *δ* 162.57, 161.08, 156.95, 138.79, 134.33, 129.28, 128.47, 127.87, 127.46, 126.63, 124.19, 121.06, 114.86, 96.65, 66.68, 56.84, 53.90, 40.53, 26.60. **HRMS (ESI)** m/z calcd C_21_H_25_N_5_O [M+H]^+^ 364.2137, found 364.2138. **HPLC purity:** 99.5% (RT = 8.479 min).

*N-(2-chloropyrimidin-4-yl)-1H-indol-5-amine* (**JX3206a**)

White solid, 62% yield. **^1^H NMR (400 MHz, DMSO-*d*_6_)** *δ* 11.12 (s, 1H), 9.81 (s, 1H), 8.04 (d, *J* = 5.9 Hz, 1H), 7.66 (s, 1H), 7.49–7.27 (m, 2H), 7.11 (s, 1H), 6.61 (d, *J* = 6.0 Hz, 1H), 6.42 (t, *J* = 2.6 Hz, 1H).

*N^4^-(1H-indol-5-yl)-N^2^-(3-morpholinopropyl)pyrimidine-2,4-diamine* (**JX3206**)

White solid, 30% yield. **^1^H NMR (400 MHz, DMSO-*d*_6_)** *δ* 10.95 (s, 1H), 8.81 (s, 1H), 7.93 (s, 1H), 7.75 (d, *J* = 5.7 Hz, 1H), 7.37–7.25 (m, 2H), 7.19 (dd, *J* = 8.6, 2.1 Hz, 1H), 6.61 (t, *J* = 5.8 Hz, 1H), 6.34 (t, *J* = 2.4 Hz, 1H), 5.90 (d, *J* = 5.8 Hz, 1H), 3.56 (t, *J* = 4.6 Hz, 4H), 3.28 (q, *J* = 6.6 Hz, 2H), 2.35 (t, *J* = 6.8 Hz, 6H), 1.69 (p, *J* = 7.1 Hz, 2H). **^13^C NMR (151 MHz, DMSO-*d*_6_)** *δ* 162.44, 161.62, 155.85, 132.76, 132.61, 128.17, 126.14, 116.62, 112.13, 111.60, 101.39, 96.08, 66.66, 56.87, 53.85, 40.51, 26.51. **HRMS (ESI)** m/z calcd C_19_H_24_N_6_O [M+H]^+^ 353.2084, found 353.2089. **HPLC purity:** 98.7% (RT = 5.709 min).

*N^1^-(2-chloropyrimidin-4-yl)-N^4^-phenylbenzene-1,4-diamine* (**JX3207a**)

White solid, 50% yield. **^1^H NMR (400 MHz, DMSO-*d*_6_)** *δ* 9.84 (s, 1H), 8.16 (s, 1H), 8.08 (d, *J* = 5.9 Hz, 1H), 7.40 (s, 2H), 7.29–7.18 (m, 2H), 7.07 (ddd, *J* = 18.4, 7.7, 1.6 Hz, 4H), 6.80 (tt, *J* = 7.3, 1.2 Hz, 1H), 6.65 (d, *J* = 5.9 Hz, 1H).

*N^4^-(1H-indol-5-yl)-N^2^-(3-morpholinopropyl)pyrimidine-2,4-diamine* (**JX3207**)

White solid, 30% yield. **^1^H NMR (400 MHz, DMSO-*d*_6_)** *δ* 8.93 (s, 1H), 7.96 (s, 1H), 7.77 (d, *J* = 5.7 Hz, 1H), 7.58 (d, *J* = 8.7 Hz, 2H), 7.18 (dd, *J* = 8.5, 7.2 Hz, 2H), 7.04–6.96 (m, 4H), 6.74 (tt, *J* = 7.3, 1.1 Hz, 1H), 6.69 (t, *J* = 5.7 Hz, 1H), 5.91 (d, *J* = 5.7 Hz, 1H), 3.56 (t, *J* = 4.7 Hz, 4H), 3.26 (q, *J* = 6.6 Hz, 2H), 2.33 (t, *J* = 7.0 Hz, 6H), 1.72–1.63 (m, 2H). **^13^C NMR (151 MHz, DMSO-*d*_6_)** *δ* 162.48, 161.02, 156.30, 144.92, 137.89, 134.16, 129.57, 121.27, 119.13, 118.76, 115.91, 96.03, 66.70, 56.86, 53.89, 40.53, 26.55. **HRMS (EI)** m/z calcd C_23_H_28_N_6_O [M]^+^ 404.2319, found 404.2330. **HPLC purity:** 99.6% (RT = 8.863 min).

*2-((2-chloropyrimidin-4-yl)amino)acridin-9(10H)-one* (**JX3208a**)

Yellow solid, 60% yield. **^1^H NMR (400 MHz, DMSO-*d*_6_)** *δ* 11.82 (s, 1H), 10.21 (s, 1H), 8.45–8.39 (m, 1H), 8.23 (dd, *J* = 8.2, 1.5 Hz, 1H), 8.17 (d, *J* = 5.8 Hz, 1H), 8.03–7.97 (m, 1H), 7.73 (ddd, *J* = 8.3, 6.8, 1.5 Hz, 1H), 7.57 (dd, *J* = 17.8, 8.6 Hz, 2H), 7.26 (t, *J* = 7.5 Hz, 1H), 6.76 (d, *J* = 5.8 Hz, 1H).

*2-((2-((3-morpholinopropyl)amino)pyrimidin-4-yl)amino)acridin-9(10H)-one* (**JX3208**)

Yellow solid, 38% yield. **^1^H NMR (400 MHz, DMSO-*d*_6_)** *δ* 11.68 (s, 1H), 9.34 (s, 1H), 8.21 (d, *J* = 8.1 Hz, 3H), 7.84 (d, *J* = 5.6 Hz, 1H), 7.70 (t, *J* = 7.4 Hz, 1H), 7.51 (t, *J* = 7.9 Hz, 2H), 7.22 (t, *J* = 7.5 Hz, 1H), 6.78 (s, 1H), 5.98 (d, *J* = 5.7 Hz, 1H), 3.54 (s, 4H), 3.34 (s, 2H), 2.42–2.24 (m, 6H), 1.80–1.62 (m, 2H). **^13^C NMR (151 MHz, DMSO-*d*_6_)** *δ* 176.83, 162.54, 160.95, 156.65, 141.06, 136.64, 135.10, 133.49, 127.45, 126.45, 121.30, 121.06, 120.32, 117.96, 117.74, 114.86, 96.10, 66.69, 56.78, 53.87, 40.52, 26.60. **HRMS (ESI)** m/z calcd C_24_H_26_N_6_O_2_ [M+H]^+^ 431.2190, found 431.2196. **HPLC purity:** 97.2% (RT = 9.406 min).

*N-(2-chloropyrimidin-4-yl)acridin-2-amine* (**JX3209a**)

White solid, 65% yield. **^1^H NMR (400 MHz, DMSO-*d*_6_)** *δ* 10.49 (s, 1H), 9.04 (s, 1H), 8.57 (d, *J* = 2.4 Hz, 1H), 8.28 (d, *J* = 5.8 Hz, 1H), 8.16 (ddd, *J* = 14.6, 8.8, 1.5 Hz, 3H), 7.95 (dd, *J* = 9.4, 2.4 Hz, 1H), 7.82 (ddd, *J* = 8.6, 6.6, 1.4 Hz, 1H), 7.62 (ddd, *J* = 8.1, 6.6, 1.1 Hz, 1H), 6.95 (d, *J* = 5.9 Hz, 1H).

*N^4^-(acridin-2-yl)-N^2^-(3-morpholinopropyl)pyrimidine-2,4-diamine* (**JX3209**)

White solid, 40% yield. **^1^H NMR (400 MHz, DMSO-*d*_6_)** *δ* 9.62 (s, 1H), 8.84 (s, 2H), 8.16–8.04 (m, 3H), 7.98–7.89 (m, 2H), 7.76 (ddd, *J* = 8.5, 6.6, 1.5 Hz, 1H), 7.59 (ddd, *J* = 8.0, 6.6, 1.2 Hz, 1H), 7.04 (s, 1H), 6.13 (d, *J* = 5.7 Hz, 1H), 3.56 (s, 4H), 3.36 (s, 2H), 2.46–2.31 (m, 6H), 1.79 (s, 2H). **^13^C NMR (151 MHz, DMSO-*d*_6_)** *δ* 13C NMR (151 MHz, DMSO) δ 162.53, 160.95, 157.12, 147.48, 146.15, 138.17, 134.22, 129.70, 129.41, 128.35, 127.62, 127.30, 127.07, 126.27, 112.66, 96.89, 66.69, 56.87, 53.90, 40.53, 26.52. **HRMS (EI)** m/z calcd C_24_H_26_N_6_O [M]^+^ 414.2168, found 414.2164. **HPLC purity:** 99.5% (RT = 8.886 min).

*2-chloro-N-(9,9-dimethyl-9H-fluoren-3-yl)pyrimidin-4-amine* (**JX3210a**)

White solid, 72% yield. **^1^H NMR (400 MHz, DMSO-*d*_6_)** *δ* 10.09 (s, 1H), 8.16 (d, *J* = 5.9 Hz, 1H), 8.01–7.95 (m, 1H), 7.79–7.73 (m, 1H), 7.59–7.53 (m, 2H), 7.47 (d, *J* = 8.1 Hz, 1H), 7.40–7.31 (m, 2H), 6.78 (d, *J* = 5.8 Hz, 1H), 1.45 (s, 6H).

*N^4^-(9,9-dimethyl-9H-fluoren-3-yl)-N^2^-(3-morpholinopropyl)pyrimidine-2,4-diamine* (**JX3210**)

White solid, 45% yield. **^1^H NMR (400 MHz, DMSO-*d*_6_)** *δ* 9.19 (s, 1H), 8.35 (s, 1H), 7.84 (d, *J* = 5.7 Hz, 1H), 7.73 (s, 1H), 7.57–7.51 (m, 1H), 7.49–7.40 (m, 2H), 7.37–7.29 (m, 2H), 6.79 (t, *J* = 5.7 Hz, 1H), 6.00 (d, *J* = 5.7 Hz, 1H), 3.52 (s, 4H), 2.33 (dd, *J* = 14.5, 7.3 Hz, 6H), 1.73 (p, *J* = 7.2 Hz, 2H), 1.42 (s, 6H). **^13^C NMR (151 MHz, DMSO-*d*_6_)** *δ* 162.52, 161.13, 156.56, 154.38, 146.92, 146.85, 140.30, 139.24, 127.77, 127.36, 127.09, 123.24, 123.09, 119.32, 117.04, 111.52, 66.64, 56.78, 53.88, 46.42, 40.54, 27.46, 26.64. **HRMS (EI)** m/z calcd C_26_H_31_N_5_O [M]^+^ 429.2529, found 429.2527. **HPLC purity:** 99.1% (RT = 9.148 min).

*3-((2-((3-morpholinopropyl)amino)pyrimidin-4-yl)amino)-9H-fluoren-9-one* (**JX3211**)

Yellow solid, 33% yield. **^1^H NMR (400 MHz, DMSO-*d*_6_)** *δ* 9.76 (s, 1H), 8.41 (s, 1H), 7.96 (d, *J* = 5.6 Hz, 1H), 7.77 (s, 1H), 7.56 (dd, *J* = 15.9, 8.5 Hz, 4H), 7.39 (t, *J* = 7.6 Hz, 1H), 7.07 (s, 1H), 6.10 (d, *J* = 5.7 Hz, 1H), 3.54 (s, 4H), 2.35 (d, *J* = 13.6 Hz, 6H), 1.76 (d, *J* = 7.4 Hz, 2H). **HRMS (ESI)** m/z calcd C_24_H_25_N_5_O_2_ [M+Na]^+^ 438.1900, found 438.1902. **HPLC purity:** 98.3% (RT = 4.328 min).

*2-chloro-N-(dibenzo[b,d]furan-2-yl)pyrimidin-4-amine* (**JX3212a**)

White solid, 70% yield. **^1^H NMR (400 MHz, DMSO-*d*_6_)** *δ* 10.16 (s, 1H), 8.32–8.26 (m, 1H), 8.19–8.09 (m, 2H), 7.73 (t, *J* = 8.5 Hz, 2H), 7.63 (d, *J* = 8.8 Hz, 1H), 7.55 (ddd, *J* = 8.4, 7.3, 1.4 Hz, 1H), 7.42 (td, *J* = 7.5, 1.0 Hz, 1H), 6.77 (d, *J* = 5.9 Hz, 1H).

*N^4^-(dibenzo[b,d]furan-2-yl)-N^2^-(3-morpholinopropyl)pyrimidine-2,4-diamine* (**JX3212**)

White solid, 50% yield. **^1^H NMR (400 MHz, DMSO-*d*_6_)** *δ* 9.28 (s, 1H), 8.74 (s, 1H), 8.08 (s, 1H), 7.85 (d, *J* = 5.7 Hz, 1H), 7.68 (d, *J* = 8.2 Hz, 1H), 7.64–7.55 (m, 2H), 7.52 (ddd, *J* = 8.4, 7.2, 1.4 Hz, 1H), 7.39 (td, *J* = 7.5, 1.0 Hz, 1H), 6.84 (t, *J* = 5.6 Hz, 1H), 6.00 (d, *J* = 5.7 Hz, 1H), 3.52 (s, 4H), 2.52 (s, 1H), 2.43–2.19 (m, 7H), 1.73 (t, *J* = 7.2 Hz, 2H). **^13^C NMR (151 MHz, DMSO-*d*_6_)** *δ* 161.12, 156.44, 156.40, 151.21, 136.85, 127.93, 124.41, 123.94, 123.89, 123.26, 120.50, 120.17, 114.98, 112.17, 111.93, 111.82, 66.66, 56.79, 53.88, 40.54, 26.65. **HRMS (EI)** m/z calcd C_23_H_25_N_5_O_2_ [M]^+^ 403.2008, found 403.2006. **HPLC purity:** 99.4% (RT = 4.674 min).

*N-(2-chloropyrimidin-4-yl)-2,3,4,9-tetrahydro-1H-carbazol-3-amine* (**JX3213a**)

White solid, 70% yield. **^1^H NMR (400 MHz, CDCl_3_)** *δ* 8.01 (s, 1H), 7.84 (s, 1H), 7.44 (d, *J* = 7.7 Hz, 1H), 7.31 (dt, *J* = 8.0, 1.0 Hz, 1H), 7.13 (dddd, *J* = 24.2, 8.2, 7.2, 1.2 Hz, 2H), 6.26 (s, 1H), 3.19 (ddt, *J* = 15.4, 4.9, 1.6 Hz, 1H), 2.96–2.79 (m, 2H), 2.75 (dd, *J* = 15.5, 6.0 Hz, 1H), 2.16 (s, 2H), 1.81 (s, 1H).

*N^2^-(3-morpholinopropyl)-N^4^-(2,3,4,9-tetrahydro-1H-carbazol-3-yl)pyrimidine-2,4-diamine* (**JX3213**)

White solid, 40% yield. **^1^H NMR (400 MHz, DMSO-*d*_6_)** *δ* 10.71 (s, 1H), 7.66–7.58 (m, 1H), 7.28 (dd, *J* = 30.9, 7.8 Hz, 2H), 7.02–6.87 (m, 3H), 6.40 (s, 1H), 5.74 (d, *J* = 5.8 Hz, 1H), 3.50 (s, 4H), 3.35 (s, 2H), 3.20 (qd, *J* = 6.8, 2.4 Hz, 2H), 3.05 (dd, *J* = 14.7, 5.0 Hz, 1H), 2.83 (t, *J* = 6.0 Hz, 2H), 2.27 (t, *J* = 7.0 Hz, 6H), 2.11 (d, *J* = 12.0 Hz, 1H), 1.84 (dq, *J* = 12.4, 7.9 Hz, 1H), 1.64 (p, *J* = 7.0 Hz, 2H). **^13^C NMR (151 MHz, DMSO-*d*_6_)** *δ* 162.70, 162.59, 155.10, 136.65, 134.03, 127.70, 120.68, 118.54, 117.60, 117.07, 111.05, 107.24, 66.83, 66.65, 56.84, 53.84, 40.38, 29.17, 27.98, 26.71, 21.89. **HRMS (EI)** m/z calcd C_23_H_30_N_6_O [M]^+^ 406.2481, found 406.2478. **HPLC purity:** 99.6% (RT = 3.678 min).

*N-(2-chloropyrimidin-4-yl)dibenzo[b,f][1,4]oxazepin-2-amine* (**JX3214a**)

White solid, 45% yield. **^1^H NMR (400 MHz, DMSO-*d*_6_)** *δ* 10.37 (s, 1H), 8.48 (s, 1H), 8.26 (d, *J* = 5.8 Hz, 1H), 7.95 (t, *J* = 7.5 Hz, 2H), 7.85 (ddd, *J* = 9.4, 7.1, 1.9 Hz, 2H), 7.65 (dd, *J* = 7.8, 3.1 Hz, 1H), 7.63 (s, 1H), 7.46 (tt, *J* = 7.5, 5.8 Hz, 2H), 6.86 (d, *J* = 5.8 Hz, 1H).

*N^4^-(dibenzo[b,f][1,4]oxazepin-2-yl)-N^2^-(3-morpholinopropyl)pyrimidine-2,4-diamine* (**JX3214**)

White solid, 28% yield. **^1^H NMR (400 MHz, DMSO-*d*_6_)** *δ* 9.48 (s, 1H), 7.94–7.67 (m, 6H), 7.54–7.38 (m, 4H), 6.91 (s, 1H), 3.46 (s, 6H), 2.21 (s, 6H), 1.75–1.69 (m, 2H). **^13^C NMR (101 MHz, CDCl_3_)** *δ* 162.73, 162.17, 160.87, 157.02, 150.71, 141.98, 139.92, 129.63, 127.90, 125.26, 124.68, 123.69, 122.37, 120.04, 119.75, 110.59, 66.96, 57.16, 53.74, 40.50, 25.90. **HRMS (ESI)** m/z calcd C_24_H_26_N_6_O_2_ [M+H]^+^ 431.2190, found 431.2197. **HPLC purity:** 96.4% (RT = 5.719 min).

*N^4^-(7H-benzo[c]carbazol-10-yl)-N^2^-(3-morpholinopropyl)pyrimidine-2,4-diamine* (**JX3215**)

White solid, 30% yield. **^1^H NMR (400 MHz, DMSO-*d*_6_)** *δ* 11.68 (s, 1H), 9.13 (s, 1H), 8.82 (s, 1H), 8.66 (d, *J* = 8.3 Hz, 1H), 8.07–8.01 (m, 1H), 7.89 (d, *J* = 8.8 Hz, 1H), 7.82 (d, *J* = 5.7 Hz, 1H), 7.71 (dd, *J* = 18.7, 8.5 Hz, 3H), 7.57 (d, *J* = 8.6 Hz, 1H), 7.45 (t, *J* = 7.4 Hz, 1H), 6.69 (s, 1H), 6.00 (d, *J* = 5.7 Hz, 1H), 3.45 (s, 6H), 2.24 (d, *J* = 20.6 Hz, 6H), 1.69 (p, *J* = 7.0 Hz, 2H). **^13^C NMR (101 MHz, CDCl_3_)** *δ* 137.92, 136.28, 130.86, 129.75, 129.28, 127.90, 127.05, 124.40, 123.16, 122.94, 112.67, 111.50, 66.96, 57.01, 53.72, 40.31, 26.06. **HRMS (ESI)** m/z calcd C_27_H_28_N_6_O [M+H]^+^ 453.2397, found 453.2393. **HPLC purity:** 99.0% (RT = 5.009 min).

*2-chloro-N-(dibenzo[b,d]furan-2-yl)-5-methylpyrimidin-4-amine* (**JX3216a**)

White solid, 55% yield. **^1^H NMR (400 MHz, DMSO-*d*_6_)** *δ* 9.09 (s, 1H), 8.27 (d, *J* = 2.1 Hz, 1H), 8.14–8.09 (m, 1H), 8.06 (d, *J* = 1.1 Hz, 1H), 7.75–7.66 (m, 3H), 7.58–7.52 (m, 1H), 7.42 (td, *J* = 7.5, 0.9 Hz, 1H), 2.21 (s, 3H).

*N^4^-(dibenzo[b,d]furan-2-yl)-5-methyl-N^2^-(3-morpholinopropyl)pyrimidine-2,4-diamine* (**JX3216**)

White solid, 44% yield. **^1^H NMR (400 MHz, DMSO-*d*_6_)** *δ* 8.64 (d, *J* = 2.2 Hz, 1H), 8.21 (s, 1H), 8.08 (d, *J* = 7.5 Hz, 1H), 7.77 (dd, *J* = 8.9, 2.2 Hz, 1H), 7.74–7.66 (m, 2H), 7.61 (d, *J* = 8.9 Hz, 1H), 7.54–7.48 (m, 1H), 7.39 (td, *J* = 7.5, 1.0 Hz, 1H), 6.53 (t, *J* = 5.7 Hz, 1H), 3.48 (t, *J* = 4.6 Hz, 4H), 3.25 (q, *J* = 6.6 Hz, 2H), 2.25 (d, *J* = 8.0 Hz, 6H), 2.07 (s, 3H), 1.65 (p, *J* = 7.2 Hz, 2H). **^13^C NMR (101 MHz, CDCl_3_)** *δ* 161.24, 159.44, 156.79, 155.68, 152.55, 134.36, 127.29, 124.40, 124.30, 122.61, 121.44, 120.54, 113.51, 111.81, 111.42, 102.89, 66.97, 57.11, 53.72, 40.54, 26.13, 13.22. **HRMS (ESI)** m/z calcd C_24_H_27_N_5_O_2_ [M+H]^+^ 418.2238, found 418.2240. **HPLC purity:** 99.9% (RT = 5.575 min).

*2-chloro-N-(dibenzo[b,d]furan-2-yl)-5-fluoropyrimidin-4-amine* (**JX3217a**)

White solid, 55% yield. **^1^H NMR (400 MHz, DMSO-*d*_6_)** *δ* 10.24–10.17 (m, 1H), 8.40–8.30 (m, 2H), 8.10 (dd, *J* = 7.8, 1.3 Hz, 1H), 7.79–7.69 (m, 3H), 7.56 (ddd, *J* = 8.4, 7.3, 1.4 Hz, 1H), 7.43 (td, *J* = 7.5, 1.0 Hz, 1H).

*N^4^-(dibenzo[b,d]furan-2-yl)-5-fluoro-N^2^-(3-morpholinopropyl)pyrimidine-2,4-diamine* (**JX3217**)

White solid, 52% yield. **^1^H NMR (400 MHz, DMSO-*d*_6_)** *δ* 9.34–9.25 (m, 1H), 8.74 (d, *J* = 2.2 Hz, 1H), 8.10 (s, 1H), 7.94 (d, *J* = 3.9 Hz, 1H), 7.77 (dd, *J* = 8.9, 2.2 Hz, 1H), 7.69 (d, *J* = 8.2 Hz, 1H), 7.64 (d, *J* = 8.9 Hz, 1H), 7.55–7.49 (m, 1H), 7.40 (t, *J* = 7.5 Hz, 1H), 6.86 (t, *J* = 5.7 Hz, 1H), 3.59–3.41 (m, 4H), 3.27 (q, *J* = 6.6 Hz, 2H), 2.39–2.13 (m, 6H), 1.68 (p, *J* = 7.0 Hz, 2H). **HRMS (ESI)** m/z calcd C_23_H_24_FN_5_O_2_ [M+H]^+^ 422.1987, found 422.1986. **HPLC purity:** 99.8% (RT = 4.539 min).

*2-chloro-N-(dibenzo[b,d]furan-2-yl)-5-methoxypyrimidin-4-amine* (**JX3218a**)

White solid, 65% yield. **^1^H NMR (400 MHz, DMSO-*d*_6_)** *δ* 9.51 (s, 1H), 8.39 (d, *J* = 2.2 Hz, 1H), 8.08 (dd, *J* = 7.6, 1.2 Hz, 1H), 7.95 (s, 1H), 7.78 (dd, *J* = 8.9, 2.2 Hz, 1H), 7.71 (dd, *J* = 8.6, 1.7 Hz, 2H), 7.54 (ddd, *J* = 8.4, 7.4, 1.4 Hz, 1H), 7.42 (td, *J* = 7.5, 1.0 Hz, 1H), 3.97 (s, 3H).

*N^4^-(dibenzo[b,d]furan-2-yl)-5-methoxy-N^2^-(3-morpholinopropyl)pyrimidine-2,4-diamine* (**JX3218**)

White solid, 48% yield. **^1^H NMR (400 MHz, DMSO-*d*_6_)** *δ* 9.34–9.25 (m, 1H), 8.74 (d, *J* = 2.2 Hz, 1H), 8.10 (s, 1H), 7.94 (d, *J* = 3.9 Hz, 1H), 7.77 (dd, *J* = 8.9, 2.2 Hz, 1H), 7.69 (d, *J* = 8.2 Hz, 1H), 7.64 (d, *J* = 8.9 Hz, 1H), 7.55–7.49 (m, 1H), 7.40 (t, *J* = 7.5 Hz, 1H), 6.86 (t, *J* = 5.7 Hz, 1H), 3.59–3.41 (m, 4H), 3.27 (q, *J* = 6.6 Hz, 2H), 2.39–2.13 (m, 6H), 1.68 (p, *J* = 7.0 Hz, 2H). **HRMS (ESI)** m/z calcd C_24_H_27_N_5_O_3_ [M+H]^+^ 434.2187, found 434.2190. **HPLC purity:** 99.1% (RT = 5.835 min).

*2-chloro-N-(dibenzo[b,d]furan-2-yl)pyridin-4-amine* (**JX3219a**)

White solid, 44% yield. **^1^H NMR (400 MHz, DMSO-*d*_6_)** *δ* 9.16 (s, 1H), 8.20 (dt, *J* = 7.6, 1.2 Hz, 1H), 8.04 (d, *J* = 2.3 Hz, 1H), 7.98 (d, *J* = 5.8 Hz, 1H), 7.76–7.69 (m, 2H), 7.54 (ddd, *J* = 8.4, 7.3, 1.4 Hz, 1H), 7.41 (td, *J* = 7.5, 1.0 Hz, 1H), 7.35 (dd, *J* = 8.8, 2.3 Hz, 1H), 6.84 (dd, *J* = 5.8, 2.1 Hz, 1H), 6.78 (d, *J* = 2.1 Hz, 1H).

*N^4^-(dibenzo[b,d]furan-2-yl)-N^2^-(3-morpholinopropyl)pyridine-2,4-diamine* (**JX3219**)

White solid, 45% yield. **^1^H NMR (400 MHz, DMSO-*d*_6_)** *δ* 8.40 (s, 1H), 8.14 (dd, *J* = 7.8, 1.3 Hz, 1H), 7.90 (d, *J* = 2.3 Hz, 1H), 7.70–7.64 (m, 3H), 7.52 (ddd, *J* = 8.4, 7.3, 1.4 Hz, 1H), 7.38 (td, *J* = 7.5, 1.0 Hz, 1H), 7.28 (dd, *J* = 8.8, 2.3 Hz, 1H), 6.14 (dt, *J* = 6.3, 3.2 Hz, 2H), 6.01 (d, *J* = 2.0 Hz, 1H), 3.53 (t, *J* = 4.6 Hz, 4H), 3.17 (q, *J* = 6.5 Hz, 2H), 2.30 (t, *J* = 7.1 Hz, 6H), 1.63 (p, *J* = 7.0 Hz, 2H). **^13^C NMR (101 MHz, CDCl_3_)** *δ* 159.25, 156.80, 153.73, 153.32, 146.81, 134.86, 127.61, 125.14, 123.85, 123.37, 122.83, 120.72, 115.51, 112.30, 111.84, 101.32, 88.78, 66.91, 56.81, 53.67, 41.02, 25.73. **HRMS (ESI)** m/z calcd C_24_H_26_N_4_O_2_ [M+H]^+^ 403.2129, found 403.2127. **HPLC purity:** 96.9% (RT = 5.430 min).

*6-bromo-N-(dibenzo[b,d]furan-2-yl)pyridin-2-amine* (**JX3220a**)

White solid, 38% yield. **^1^H NMR (400 MHz, DMSO-*d*_6_)** *δ* 9.49 (s, 1H), 8.34 (d, *J* = 2.1 Hz, 1H), 8.08–8.03 (m, 1H), 7.72–7.61 (m, 3H), 7.55–7.47 (m, 2H), 7.41 (td, *J* = 7.5, 1.0 Hz, 1H), 6.94 (d, *J* = 7.4 Hz, 1H), 6.84 (d, *J* = 8.2 Hz, 1H).

*N^2^-(dibenzo[b,d]furan-2-yl)-N^6^-(3-morpholinopropyl)pyridine-2,6-diamine* (**JX3220**)

White solid, 42% yield. **^1^H NMR (400 MHz, DMSO-*d*_6_)** *δ* 8.80–8.73 (m, 2H), 8.02 (dd, *J* = 7.8, 1.3 Hz, 1H), 7.66 (d, *J* = 8.2 Hz, 1H), 7.55 (d, *J* = 1.4 Hz, 2H), 7.49 (ddd, *J* = 8.4, 7.3, 1.3 Hz, 1H), 7.37 (td, *J* = 7.5, 1.0 Hz, 1H), 7.21 (t, *J* = 7.9 Hz, 1H), 6.37 (t, *J* = 5.5 Hz, 1H), 5.98 (d, *J* = 7.7 Hz, 1H), 5.86 (d, *J* = 7.9 Hz, 1H), 3.52 (t, *J* = 4.5 Hz, 4H), 3.36 (s, 2H), 2.35 (dt, *J* = 24.5, 6.0 Hz, 6H), 1.77 (p, *J* = 7.1 Hz, 2H). **HRMS (ESI)** m/z calcd C_24_H_26_N_4_O_2_ [M]^+^ 403.2129, found 403.2126. **HPLC purity:** 98.0% (RT = 11.972 min).

*5-bromo-N-(3-morpholinopropyl)pyridin-3-amine* (**JX3221a**)

White solid, 52% yield. **^1^H NMR (400 MHz, DMSO-*d*_6_)** *δ* 7.93 (d, *J* = 2.4 Hz, 1H), 7.77 (d, *J* = 2.0 Hz, 1H), 7.09 (t, *J* = 2.2 Hz, 1H), 6.25 (t, *J* = 5.6 Hz, 1H), 3.59 (t, *J* = 4.6 Hz, 4H), 3.12–3.03 (m, 2H), 2.36 (d, *J* = 6.6 Hz, 6H), 1.67 (t, *J* = 6.9 Hz, 2H).

*N^3^-(dibenzo[b,d]furan-2-yl)-N^5^-(3-morpholinopropyl)pyridine-3,5-diamine* (**JX3221**)

White solid, 36% yield. **^1^H NMR (400 MHz, DMSO-*d*_6_)** *δ* 8.13–8.08 (m, 2H), 7.82 (d, *J* = 2.3 Hz, 1H), 7.66 (dt, *J* = 8.3, 0.8 Hz, 1H), 7.62–7.58 (m, 2H), 7.50 (ddd, *J* = 8.4, 7.3, 1.4 Hz, 1H), 7.45 (d, *J* = 2.3 Hz, 1H), 7.36 (td, *J* = 7.5, 1.0 Hz, 1H), 7.23 (dd, *J* = 8.8, 2.4 Hz, 1H), 6.62 (t, *J* = 2.3 Hz, 1H), 3.52 (t, *J* = 4.6 Hz, 4H), 3.02 (q, *J* = 6.4 Hz, 2H), 2.37–2.26 (m, 6H), 1.72–1.63 (m, 2H). **^13^C NMR (101 MHz, CDCl_3_)** *δ* 156.81, 152.37, 145.24, 141.90, 137.29, 128.18, 127.96, 127.38, 125.09, 124.04, 122.64, 121.07, 120.67, 112.29, 111.78, 104.84, 67.01, 57.55, 53.72, 43.05, 25.07. **HRMS (ESI)** m/z calcd C_24_H_26_N_4_O_2_ [M]^+^ 403.2129, found 403.2126. **HPLC purity:** 100.0% (RT = 4.271 min).

*6-chloro-N-(dibenzo[b,d]furan-2-yl)pyrimidin-4-amine* (**JX3222a**)

White solid, 60% yield. **^1^H NMR (400 MHz, DMSO-*d*_6_)** *δ* 10.03 (s, 1H), 8.49 (s, 1H), 8.37 (d, *J* = 2.3 Hz, 1H), 8.15 (d, *J* = 7.7 Hz, 1H), 7.71 (dd, *J* = 8.5, 2.9 Hz, 2H), 7.62 (dd, *J* = 8.8, 2.3 Hz, 1H), 7.57–7.51 (m, 1H), 7.41 (t, *J* = 7.5 Hz, 1H), 6.80 (s, 1H).

*N^4^-(dibenzo[b,d]furan-2-yl)-N^6^-(3-morpholinopropyl)pyrimidine-4,6-diamine* (**JX3222**)

White solid, 46% yield. **^1^H NMR (400 MHz, DMSO-*d*_6_)** *δ* 8.98 (s, 1H), 8.26 (s, 1H), 8.10 (dd, *J* = 7.8, 1.2 Hz, 2H), 7.68 (d, *J* = 8.3 Hz, 1H), 7.62 (d, *J* = 8.8 Hz, 1H), 7.55–7.50 (m, 2H), 7.38 (td, *J* = 7.5, 1.0 Hz, 1H), 6.88 (t, *J* = 5.5 Hz, 1H), 5.74 (s, 1H), 3.55 (t, *J* = 4.6 Hz, 4H), 3.21 (s, 2H), 2.33 (dd, *J* = 5.7, 3.4 Hz, 6H), 1.65 (q, *J* = 7.0 Hz, 2H). **^13^C NMR (101 MHz, CDCl_3_)** *δ* 163.21, 162.12, 158.32, 156.82, 156.57, 153.57, 133.89, 127.63, 125.18, 123.88, 123.78, 122.84, 120.74, 116.15, 112.33, 111.86, 66.91, 56.95, 53.67, 40.49, 25.29. **HRMS (ESI)** m/z calcd C_23_H_25_N_5_O_2_ [M+H]^+^ 404.2081, found 404.2084. **HPLC purity:** 99.8% (RT = 4.861 min).

*N^2^-(dibenzo[b,d]furan-2-yl)-N^6^-(3-morpholinopropyl)pyrazine-2,6-diamine* (**JX3223**)

White solid, 38% yield. **^1^H NMR (400 MHz, DMSO-*d*_6_)** *δ* 8.47 (s, 1H), 8.25 (s, 1H), 8.18–8.14 (m, 2H), 7.81 (d, *J* = 8.7 Hz, 1H), 7.75 (d, *J* = 8.3 Hz, 1H), 7.66 (s, 1H), 7.56 (ddd, *J* = 8.4, 7.3, 1.4 Hz, 1H), 7.45 (dd, *J* = 8.7, 2.3 Hz, 1H), 7.41 (td, *J* = 7.5, 0.9 Hz, 1H), 7.34 (s, 1H), 3.45 (t, *J* = 4.6 Hz, 4H), 3.14–3.07 (m, 2H), 2.17 (d, *J* = 6.8 Hz, 6H), 1.57–1.50 (m, 2H). **^13^C NMR (101 MHz, CDCl_3_)** *δ* 156.86, 154.95, 153.05, 151.13, 146.21, 136.79, 135.53, 135.42, 127.83, 125.88, 124.67, 123.73, 123.02, 120.96, 113.14, 111.91, 66.95, 57.45, 53.61, 41.01, 24.84. **HRMS (ESI)** m/z calcd C_23_H_25_N_5_O_2_ [M+H]^+^ 404.2081, found 404.2080. **HPLC purity:** 96.3% (RT = 6.332 min).

*6-chloro-N-(3-morpholinopropyl)pyridazin-4-amine* (**JX3224a**)

White solid, 62% yield. **^1^H NMR (400 MHz, DMSO-*d*_6_)** *δ* 8.51 (s, 1H), 7.43 (s, 1H), 6.73 (d, *J* = 2.5 Hz, 1H), 3.58 (t, *J* = 4.7 Hz, 4H), 3.15 (q, *J* = 6.2 Hz, 2H), 2.32 (q, *J* = 5.8 Hz, 6H), 1.67 (p, *J* = 6.9 Hz, 2H).

*N^2^-(dibenzo[b,d]furan-2-yl)-N^6^-(3-morpholinopropyl)pyrazine-2,6-diamine* (**JX3224**)

White solid, 40% yield. **^1^H NMR (400 MHz, DMSO-*d*_6_)** *δ* 8.85 (s, 1H), 8.51 (d, *J* = 1.5 Hz, 1H), 8.16 (d, *J* = 2.4 Hz, 1H), 8.07 (d, *J* = 7.6 Hz, 1H), 7.67 (d, *J* = 8.2 Hz, 1H), 7.61 (d, *J* = 1.8 Hz, 2H), 7.50 (d, *J* = 1.3 Hz, 1H), 7.38 (s, 1H), 6.76 (s, 1H), 6.00 (d, *J* = 2.4 Hz, 1H), 3.58–3.54 (m, 4H), 3.07 (q, *J* = 6.4 Hz, 2H), 2.38–2.31 (m, 6H), 1.72 (p, *J* = 6.9 Hz, 2H). **HRMS (ESI)** m/z calcd C_23_H_25_N_5_O_2_ [M+H]^+^ 404.2081, found 404.2077. **HPLC purity:** 99.4% (RT = 4.715 min).

*5-bromo-N-(3-morpholinopropyl)pyrazin-2-amine* (**JX3225a**)

White solid, 50% yield. **^1^H NMR (400 MHz, DMSO-*d*_6_)** *δ* 8.07 (s, 1H), 7.73 (s, 1H), 7.31 (t, *J* = 5.6 Hz, 1H), 3.56 (t, *J* = 4.6 Hz, 4H), 3.24 (q, *J* = 6.6 Hz, 2H), 2.32 (d, *J* = 8.2 Hz, 6H), 1.67 (p, *J* = 7.0 Hz, 2H).

*N^2^-(dibenzo[b,d]furan-2-yl)-N^5^-(3-morpholinopropyl)pyrazine-2,5-diamine* (**JX3225**)

White solid, 40% yield. **^1^H NMR (400 MHz, DMSO-*d*_6_)** *δ* 8.65 (s, 1H), 8.16 (d, *J* = 2.3 Hz, 1H), 8.05 (d, *J* = 7.5 Hz, 1H), 7.83 (d, *J* = 1.5 Hz, 1H), 7.67 (d, *J* = 1.5 Hz, 1H), 7.63 (d, *J* = 8.2 Hz, 1H), 7.54 (d, *J* = 8.9 Hz, 1H), 7.48 (ddd, *J* = 8.4, 7.3, 1.4 Hz, 1H), 7.42 (dd, *J* = 8.9, 2.3 Hz, 1H), 7.35 (td, *J* = 7.5, 1.0 Hz, 1H), 6.32 (t, *J* = 5.6 Hz, 1H), 3.58 (t, *J* = 4.6 Hz, 4H), 3.22 (q, *J* = 6.5 Hz, 2H), 2.35 (q, *J* = 5.5 Hz, 6H), 1.70 (p, *J* = 7.1 Hz, 2H). **^13^C NMR (101 MHz, CDCl_3_)** *δ* 156.78, 151.85, 150.09, 144.71, 137.35, 129.89, 127.78, 127.18, 124.90, 124.23, 122.50, 120.72, 119.34, 112.11, 111.70, 110.10, 66.83, 57.36, 53.67, 41.63, 25.35. **HRMS (ESI)** m/z calcd C_23_H_25_N_5_O_2_ [M+H]^+^ 404.2081, found 404.2083. **HPLC purity:** 95.1% (RT = 4.484 min).

*tert-butyl ((1s,4s)-4-(dibenzo[b,d]furan-2-ylamino)cyclohexyl)carbamate* (**JX3226a**)

White solid, 30% yield. **^1^H NMR (400 MHz, DMSO-*d*_6_)** *δ* 8.06–8.00 (m, 1H), 7.59 (d, *J* = 8.2 Hz, 1H), 7.48–7.39 (m, 2H), 7.33 (td, *J* = 7.5, 1.0 Hz, 1H), 7.20 (d, *J* = 2.4 Hz, 1H), 6.86 (dd, *J* = 8.9, 2.4 Hz, 1H), 6.81–6.60 (m, 1H), 5.34 (d, *J* = 7.0 Hz, 1H), 3.44 (s, 1H), 2.54 (s, 1H), 1.69 (s, 4H), 1.41 (s, 9H), 1.40 (s, 4H).

*(1s,4s)-N^1^-(dibenzo[b,d]furan-2-yl)-N^4^-(3-morpholinopropyl)cyclohexane-1,4-diamine* (**JX3226**)

White solid, 25% yield. **^1^H NMR (400 MHz, DMSO-*d*_6_)** *δ* 8.01 (dd, *J* = 7.8, 1.3 Hz, 1H), 7.57 (d, *J* = 8.2 Hz, 1H), 7.47–7.36 (m, 2H), 7.33–7.28 (m, 1H), 7.19 (d, *J* = 2.4 Hz, 1H), 6.85 (dd, *J* = 8.9, 2.4 Hz, 1H), 5.38 (d, *J* = 7.9 Hz, 1H), 3.56 (t, *J* = 4.6 Hz, 4H), 3.43 (s, 1H), 2.62–2.52 (m, 4H), 2.32 (dd, *J* = 9.3, 5.3 Hz, 6H), 1.74–1.61 (m, 4H), 1.58 (t, *J* = 7.9 Hz, 6H). **HRMS (ESI)** m/z calcd C_25_H_33_N_3_O_2_ [M+H]^+^ 408.2646, found 408.2643. **HPLC purity:** 99.0% (RT = 6.737 min).

*(1s,3r,5R,7S)-N^1^-(dibenzo[b,d]furan-2-yl)adamantane-1,3-diamine* (**JX3227a**)

White solid, 25% yield. **^1^H NMR (400 MHz, DMSO-*d*_6_)** *δ* 8.05 (dd, *J* = 7.9, 1.3 Hz, 1H), 7.60 (d, *J* = 8.2 Hz, 1H), 7.48–7.40 (m, 3H), 7.33 (td, *J* = 7.5, 1.0 Hz, 1H), 7.00 (dd, *J* = 8.8, 2.3 Hz, 1H), 4.65 (s, 1H), 2.14 (d, *J* = 4.8 Hz, 2H), 1.79–1.70 (m, 4H), 1.62 (s, 2H), 1.53 (d, *J* = 12.2 Hz, 1H), 1.45 (d, *J* = 2.9 Hz, 5H).

*(1s,3r,5R,7S)-N^1^-(dibenzo[b,d]furan-2-yl)-N^3^-(3-morpholinopropyl)adamantane-1,3-diamine* (**JX3227**)

White solid, 20% yield. **^1^H NMR (400 MHz, DMSO-*d*_6_)** *δ* 8.05 (dd, *J* = 7.7, 1.3 Hz, 1H), 7.60 (d, *J* = 8.2 Hz, 1H), 7.48–7.45 (m, 2H), 7.42 (d, *J* = 8.9 Hz, 1H), 7.33 (td, *J* = 7.5, 1.0 Hz, 1H), 7.00 (dd, *J* = 8.8, 2.2 Hz, 1H), 4.71 (s, 1H), 3.53 (t, *J* = 4.6 Hz, 4H), 2.56 (s, 2H), 2.30 (q, *J* = 7.7 Hz, 6H), 2.18 (s, 2H), 1.78 (q, *J* = 12.2 Hz, 4H), 1.68 (s, 2H), 1.52 (d, *J* = 14.7 Hz, 8H). **HRMS (ESI)** m/z calcd C_29_H_37_N_3_O_2_ [M+H]^+^ 460.2959, found 460.2956. **HPLC purity:** 95.9% (RT = 6.974 min).

*tert-butyl 2-(dibenzo[b,d]furan-2-ylamino)-7-azaspiro[3.5]nonane-7-carboxylate* (**JX3228a**)

White solid, 35% yield. **^1^H NMR (400 MHz, DMSO-*d*_6_)** *δ* 8.06–8.01 (m, 1H), 7.60–7.55 (m, 1H), 7.46–7.38 (m, 2H), 7.31 (td, *J* = 7.5, 1.0 Hz, 1H), 7.06 (d, *J* = 2.4 Hz, 1H), 6.75 (dd, *J* = 8.8, 2.4 Hz, 1H), 5.83 (d, *J* = 6.7 Hz, 1H), 3.92 (q, *J* = 7.3 Hz, 1H), 3.22 (t, *J* = 5.6 Hz, 2H), 2.41–2.32 (m, 2H), 1.65–1.55 (m, 4H), 1.50–1.45 (m, 2H), 1.40 (d, *J* = 1.5 Hz, 9H).

*N-(dibenzo[b,d]furan-2-yl)-7-(3-morpholinopropyl)-7-azaspiro[3.5]nonan-2-amine* (**JX3228**)

White solid, 25% yield. **^1^H NMR (400 MHz, DMSO-*d*_6_)** *δ* 8.03 (dd, *J* = 7.7, 1.3 Hz, 1H), 7.58 (d, *J* = 8.2 Hz, 1H), 7.47–7.37 (m, 2H), 7.31 (td, *J* = 7.5, 1.0 Hz, 1H), 7.06 (d, *J* = 2.3 Hz, 1H), 6.75 (dd, *J* = 8.9, 2.4 Hz, 1H), 5.83 (d, *J* = 6.6 Hz, 1H), 3.90 (p, *J* = 7.3 Hz, 1H), 3.56 (t, *J* = 4.6 Hz, 4H), 2.50–2.06 (m, 14H), 1.79–1.42 (m, 8H). **HRMS (ESI)** m/z calcd C_27_H_35_N_3_O_2_ [M+H]^+^ 434.2802, found 434.2805. **HPLC purity:** 98.0% (RT = 5.943 min).

*tert-butyl 7-(dibenzo[b,d]furan-2-yl)-2,7-diazaspiro[3.5]nonane-2-carboxylate* (**JX3229a**)

White solid, 45% yield. **^1^H NMR (400 MHz, DMSO-*d*_6_)** *δ* 8.10 (d, *J* = 7.6 Hz, 1H), 7.68 (d, *J* = 2.5 Hz, 1H), 7.62 (d, *J* = 8.2 Hz, 1H), 7.55–7.44 (m, 2H), 7.35 (t, *J* = 7.5 Hz, 1H), 7.18 (dd, *J* = 9.0, 2.6 Hz, 1H), 3.62 (s, 4H), 3.13 (d, *J* = 5.6 Hz, 4H), 1.84 (t, *J* = 5.5 Hz, 4H), 1.39 (s, 9H).

*4-(3-(7-(dibenzo[b,d]furan-2-yl)-2,7-diazaspiro[3.5]nonan-2-yl)propyl)morpholine* (**JX3229**)

White solid, 40% yield. **^1^H NMR (400 MHz, DMSO-*d*_6_)** *δ* 8.10 (dd, *J* = 7.7, 1.3 Hz, 1H), 7.67 (d, *J* = 2.6 Hz, 1H), 7.62 (d, *J* = 8.2 Hz, 1H), 7.52 (d, *J* = 9.0 Hz, 1H), 7.47 (td, *J* = 7.8, 1.4 Hz, 1H), 7.35 (t, *J* = 7.4 Hz, 1H), 7.17 (dd, *J* = 9.0, 2.6 Hz, 1H), 3.56 (t, *J* = 4.6 Hz, 4H), 3.14–3.09 (m, 4H), 3.01 (s, 4H), 2.46 (t, *J* = 6.6 Hz, 2H), 2.35–2.22 (m, 6H), 1.87–1.79 (m, 4H), 1.45 (q, *J* = 7.2 Hz, 2H). **HRMS (ESI)** m/z calcd C_26_H_33_N_3_O_2_ [M+H]^+^ 420.2646, found 420.2649. **HPLC purity:** 95.5% (RT = 6.559 min).

*4-(3-(9-(dibenzo[b,d]furan-2-yl)-3,9-diazaspiro[5.5]undecan-3-yl)propyl)morpholine* (**JX3230**)

White solid, 45% yield. **^1^H NMR (400 MHz, DMSO-*d*_6_)** *δ* 8.10 (dd, *J* = 7.7, 1.3 Hz, 1H), 7.67 (d, *J* = 2.5 Hz, 1H), 7.62 (d, *J* = 8.2 Hz, 1H), 7.52 (d, *J* = 9.0 Hz, 1H), 7.47 (ddd, *J* = 8.4, 7.3, 1.4 Hz, 1H), 7.35 (td, *J* = 7.5, 1.1 Hz, 1H), 7.17 (dd, *J* = 9.0, 2.6 Hz, 1H), 3.56 (t, *J* = 4.6 Hz, 4H), 3.16 (t, *J* = 5.7 Hz, 4H), 2.46–2.16 (m, 12H), 1.55 (dt, *J* = 40.6, 5.7 Hz, 10H). **^13^C NMR (101 MHz, CDCl_3_)** *δ* 162.73, 162.17, 160.87, 157.02, 150.71, 141.98, 139.92, 129.63, 127.90, 125.26, 124.68, 123.69, 122.37, 120.04, 119.75, 110.59, 66.96, 57.16, 53.74, 40.50, 25.90. **HRMS (ESI)** m/z calcd C_28_H_37_N_3_O_2_ [M+H]^+^ 448.2959, found 448.2955. **HPLC purity:** 96.7% (RT = 8.761 min).

*N-(dibenzo[b,d]furan-2-yl)-2-(3-morpholinopropoxy)pyrimidin-4-amine* (**JX3231**)

White solid, 22% yield. **^1^H NMR (400 MHz, DMSO-*d*_6_)** *δ* 9.73 (s, 1H), 8.44 (d, *J* = 2.1 Hz, 1H), 8.10–8.05 (m, 2H), 7.72–7.61 (m, 3H), 7.53 (ddd, *J* = 8.4, 7.3, 1.4 Hz, 1H), 7.40 (td, *J* = 7.5, 1.0 Hz, 1H), 6.43 (d, *J* = 5.8 Hz, 1H), 4.32 (t, *J* = 6.7 Hz, 2H), 3.53 (t, *J* = 4.6 Hz, 4H), 2.40 (t, *J* = 7.1 Hz, 2H), 2.33 (d, *J* = 5.3 Hz, 4H), 1.89 (p, *J* = 6.9 Hz, 2H). **HRMS (ESI)** m/z calcd C_23_H_24_N_4_O_3_ [M+H]^+^ 405.1921, found 405.1917. **HPLC purity:** 98.0% (RT = 4.871 min).

*N^4^-(dibenzo[b,d]furan-2-yl)-N^2^-methyl-N^2^-(3-morpholinopropyl)pyrimidine-2,4-diamine* (**JX3232**)

White solid, 28% yield. **^1^H NMR (400 MHz, DMSO-*d*_6_)** *δ* 9.34 (s, 1H), 8.63 (s, 1H), 8.01 (dd, *J* = 7.7, 1.4 Hz, 1H), 7.92 (d, *J* = 5.7 Hz, 1H), 7.68 (d, *J* = 8.2 Hz, 1H), 7.65–7.58 (m, 2H), 7.52 (ddd, *J* = 8.4, 7.2, 1.3 Hz, 1H), 7.39 (td, *J* = 7.5, 1.0 Hz, 1H), 6.02 (d, *J* = 5.7 Hz, 1H), 3.64 (t, *J* = 7.2 Hz, 2H), 3.49 (s, 4H), 3.13 (s, 3H), 2.26 (s, 6H), 1.75 (t, *J* = 7.3 Hz, 2H). **HRMS (ESI)** m/z calcd C_24_H_27_N_5_O_2_ [M+H]^+^ 418.2238, found 418.2231. **HPLC purity:** 98.8% (RT = 6.254 min).

*N^4^-(dibenzo[b,d]furan-2-yl)-N^2^-(2-methyl-3-morpholinopropyl)pyrimidine-2,4-diamine* (**JX3233**)

White solid, 20% yield. **^1^H NMR (400 MHz, DMSO-*d*_6_)** *δ* 9.31 (s, 1H), 8.70 (s, 1H), 8.03 (s, 1H), 7.85 (d, *J* = 5.7 Hz, 1H), 7.68 (d, *J* = 8.2 Hz, 1H), 7.61 (s, 2H), 7.54–7.49 (m, 1H), 7.39 (t, *J* = 7.5 Hz, 1H), 6.93 (t, *J* = 5.7 Hz, 1H), 6.00 (d, *J* = 5.7 Hz, 1H), 3.53 (s, 4H), 2.46–2.16 (m, 6H), 2.16–2.08 (m, 1H), 2.03 (q, *J* = 6.7 Hz, 1H), 1.22 (d, *J* = 2.9 Hz, 1H), 0.90 (d, *J* = 6.5 Hz, 3H). **^13^C NMR (101 MHz, CDCl_3_)** *δ* 162.23, 162.01, 156.80, 156.66, 153.21, 133.86, 127.51, 124.84, 123.97, 122.75, 120.68, 115.07, 112.45, 112.25, 111.96, 111.82, 67.48, 67.00, 65.42, 54.13, 29.61, 17.19. **HRMS (ESI)** m/z calcd C_24_H_27_N_5_O_2_ [M+H]^+^ 418.2238, found 418.2236. **HPLC purity:** 99.7% (RT = 6.682 min).

*1-((4-(dibenzo[b,d]furan-2-ylamino)pyrimidin-2-yl)amino)-3-morpholinopropan-2-ol* (**JX3234**)

White solid, 18% yield. **^1^H NMR (400 MHz, DMSO-*d*_6_)** *δ* 9.34 (s, 1H), 8.77 (s, 1H), 8.17 (s, 1H), 7.86 (d, *J* = 5.7 Hz, 1H), 7.68 (d, *J* = 8.2 Hz, 1H), 7.65–7.46 (m, 3H), 7.39 (t, *J* = 7.5 Hz, 1H), 6.78 (s, 1H), 6.03 (d, *J* = 5.7 Hz, 1H), 4.92 (d, *J* = 128.7 Hz, 1H), 3.92–3.80 (m, 1H), 3.68–3.39 (m, 6H), 2.36 (tdd, *J* = 19.3, 12.3, 6.8 Hz, 6H). **HRMS (ESI)** m/z calcd C_23_H_25_N_5_O_3_ [M+H]^+^ 420.2030, found 420.2028. **HPLC purity:** 98.9% (RT = 4.877 min).

*N^4^-(dibenzo[b,d]furan-2-yl)-N^2^-((1-(morpholinomethyl)cyclopropyl)methyl)pyrimidine-2,4-diamine* (**JX3235**)

White solid, 20% yield. **^1^H NMR (400 MHz, DMSO-*d*_6_)** *δ* 9.30 (s, 1H), 8.65 (s, 1H), 8.07 (s, 1H), 7.84 (d, *J* = 5.7 Hz, 1H), 7.68 (d, *J* = 8.2 Hz, 1H), 7.62 (s, 2H), 7.55–7.48 (m, 1H), 7.40 (t, *J* = 7.5 Hz, 1H), 6.77 (s, 1H), 6.01 (d, *J* = 5.7 Hz, 1H), 3.55 (s, 4H), 3.45 (s, 2H), 2.35 (d, *J* = 36.8 Hz, 6H), 0.53 (q, *J* = 4.1 Hz, 2H), 0.26 (q, *J* = 4.1 Hz, 2H). **^13^C NMR (101 MHz, CDCl_3_)** *δ* 162.44, 161.98, 156.81, 156.68, 153.26, 133.79, 127.52, 124.85, 123.96, 122.91, 122.76, 120.67, 115.17, 111.98, 111.83, 94.36, 67.00, 65.87, 54.15, 29.70, 17.58, 10.20. **HRMS (ESI)** m/z calcd C_25_H_27_N_5_O_2_ [M+H]^+^ 430.2238, found 430.2241. **HPLC purity:** 99.6% (RT = 7.300 min).

*N-(2-((4-(dibenzo[b,d]furan-2-ylamino)pyrimidin-2-yl)amino)ethyl)morpholine-4-carboxamide* (**JX3236**)

White solid, 60% yield. **^1^H NMR (400 MHz, DMSO-*d*_6_)** *δ* 10.78 (s, 1H), 8.59 (d, *J* = 6.3 Hz, 1H), 8.30 (s, 1H), 8.15 (s, 1H), 7.89 (d, *J* = 7.0 Hz, 1H), 7.73 (t, *J* = 8.6 Hz, 3H), 7.56 (ddd, *J* = 8.4, 7.3, 1.4 Hz, 1H), 7.42 (td, *J* = 7.5, 0.9 Hz, 1H), 6.74 (s, 1H), 6.32 (d, *J* = 7.0 Hz, 1H), 3.58–3.43 (m, 6H), 3.20 (s, 4H). **HRMS (ESI)** m/z calcd C_23_H_24_N_6_O_3_ [M+H]^+^ 433.1983, found 433.1987. **HPLC purity:** 97.9% (RT = 4.020 min).

*tert-butyl (3-((4-(dibenzo[b,d]furan-2-ylamino)pyrimidin-2-yl)amino)propyl)carbamate* (**JX3237**)

White solid, 45% yield. **^1^H NMR (400 MHz, DMSO-*d*_6_)** *δ* 9.30 (s, 1H), 8.70 (s, 1H), 8.11 (s, 1H), 7.85 (d, *J* = 5.7 Hz, 1H), 7.67 (d, *J* = 8.2 Hz, 1H), 7.61 (s, 2H), 7.54–7.48 (m, 1H), 7.39 (t, *J* = 7.5 Hz, 1H), 6.86–6.73 (m, 2H), 6.01 (d, *J* = 5.7 Hz, 1H), 3.01 (q, *J* = 6.7 Hz, 2H), 1.69 (s, 2H), 1.37 (s, 9H). **HRMS (ESI)** m/z calcd C_24_H_27_N_5_O_3_ [M+H]^+^ 434.2187, found 434.2183. **HPLC purity:** 95.8% (RT = 5.055 min).

*N^4^-(dibenzo[b,d]furan-2-yl)-N^2^-(3-(diethylamino)propyl)pyrimidine-2,4-diamine* (**JX3238**)

White solid, 45% yield. **^1^H NMR (400 MHz, DMSO-*d*_6_)** *δ* 9.38 (s, 1H), 8.73 (s, 1H), 8.12 (s, 1H), 7.87 (d, *J* = 5.7 Hz, 1H), 7.68 (d, *J* = 8.3 Hz, 1H), 7.61 (s, 2H), 7.52 (ddd, *J* = 8.4, 7.3, 1.4 Hz, 1H), 7.40 (t, *J* = 7.4 Hz, 1H), 6.94 (s, 1H), 6.05 (d, *J* = 5.7 Hz, 1H), 2.72 (d, *J* = 33.1 Hz, 6H), 1.80 (d, *J* = 9.0 Hz, 2H), 1.03 (s, 6H). **HRMS (ESI)** m/z calcd C_23_H_27_N_5_O [M]^+^ 390.2288, found 390.2285. **HPLC purity:** 98.7% (RT = 7.060 min).

*N^4^-(dibenzo[b,d]furan-2-yl)-N^2^-(3-(pyrrolidin-1-yl)propyl)pyrimidine-2,4-diamine* (**JX3239**)

White solid, 44% yield. **^1^H NMR (400 MHz, DMSO-*d*_6_)** *δ* 9.30 (s, 1H), 8.77 (s, 1H), 8.08 (s, 1H), 7.85 (d, *J* = 5.7 Hz, 1H), 7.68 (d, *J* = 8.2 Hz, 1H), 7.64–7.48 (m, 3H), 7.40 (d, *J* = 7.5 Hz, 1H), 6.84 (t, *J* = 5.8 Hz, 1H), 6.01 (d, *J* = 5.7 Hz, 1H), 2.45 (d, *J* = 7.1 Hz, 2H), 2.39 (s, 4H), 1.75 (q, *J* = 7.2 Hz, 2H), 1.62 (s, 4H). **HRMS (ESI)** m/z calcd C_23_H_25_N_5_O [M]^+^ 388.2132, found 388.2133. **HPLC purity:** 96.7% (RT = 5.740 min).

*N^4^-(dibenzo[b,d]furan-2-yl)-N^2^-(3-(6,6-dimethyl-3-azabicyclo[3.1.0]hexan-3-yl)propyl)pyrimidine-2,4-diamine* (**JX3240**)

White solid, 28% yield. **^1^H NMR (400 MHz, DMSO-*d*_6_)** *δ* 9.41 (s, 1H), 8.72 (s, 1H), 8.14 (s, 1H), 7.86 (d, *J* = 5.7 Hz, 1H), 7.68 (d, *J* = 8.2 Hz, 1H), 7.66–7.56 (m, 2H), 7.55–7.48 (m, 1H), 7.40 (t, *J* = 7.5 Hz, 1H), 6.93 (s, 1H), 6.05 (d, *J* = 5.8 Hz, 1H), 2.80 (s, 4H), 2.50 (s, 2H), 1.79 (s, 2H), 1.43 (s, 2H), 1.02 (d, *J* = 47.2 Hz, 6H).9.30 (s, 1H), 8.77 (s, 1H), 8.08 (s, 1H), 7.85 (d, *J* = 5.7 Hz, 1H), 7.68 (d, *J* = 8.2 Hz, 1H), 7.64–7.48 (m, 3H), 7.40 (d, *J* = 7.5 Hz, 1H), 6.84 (t, *J* = 5.8 Hz, 1H), 6.01 (d, *J* = 5.7 Hz, 1H), 2.45 (d, *J* = 7.1 Hz, 2H), 2.39 (s, 4H), 1.75 (q, *J* = 7.2 Hz, 2H), 1.62 (s, 4H). **^13^C NMR (101 MHz, CDCl_3_)** *δ* 161.90, 156.71, 153.04, 133.90, 127.43, 124.65, 123.98, 122.76, 122.56, 120.75, 114.75, 111.84, 111.73, 95.51, 53.51, 39.14, 29.46, 27.39, 26.45, 23.04, 14.32. **HRMS (ESI)** m/z calcd C_26_H_29_N_5_O [M+H]^+^ 428.2445, found 428.2441. **HPLC purity:** 97.0% (RT = 5.793 min).

*N-(3-((4-(dibenzo[b,d]furan-2-ylamino)pyrimidin-2-yl)amino)propyl)morpholine-4-carboxamide* (**JX3241**)

White solid, 40% yield. **^1^H NMR (400 MHz, DMSO-*d*_6_)** *δ* 10.11 (s, 1H), 8.67 (s, 1H), 8.20–8.01 (m, 1H), 7.86 (d, *J* = 6.2 Hz, 1H), 7.67 (dd, *J* = 17.6, 9.7 Hz, 3H), 7.53 (ddd, *J* = 8.4, 7.3, 1.4 Hz, 1H), 7.41 (td, *J* = 7.5, 1.0 Hz, 1H), 6.58 (s, 1H), 6.15 (d, *J* = 6.3 Hz, 1H), 3.50 (s, 4H), 3.23 (t, *J* = 4.7 Hz, 4H), 3.14 (q, *J* = 6.5 Hz, 2H), 1.73 (s, 2H). **HRMS (ESI)** m/z calcd C_24_H_26_N_6_O_3_ [M+H]^+^ 447.2139, found 447.2140. **HPLC purity:** 96.1% (RT = 3.985 min).

*N^4^-(dibenzo[b,d]furan-2-yl)-N^2^-(3-((2S,6R)-2,6-dimethylmorpholino)propyl)pyrimidine-2,4-diamine* (**JX3242**)

White solid, 34% yield. **^1^H NMR (400 MHz, DMSO-*d*_6_)** *δ* 9.30 (s, 1H), 8.72 (s, 1H), 8.11 (d, *J* = 19.9 Hz, 1H), 7.85 (d, *J* = 5.7 Hz, 1H), 7.68 (d, *J* = 8.3 Hz, 1H), 7.63–7.55 (m, 2H), 7.52 (ddd, *J* = 8.4, 7.3, 1.4 Hz, 1H), 7.42–7.35 (m, 1H), 6.84 (t, *J* = 5.7 Hz, 1H), 6.01 (d, *J* = 5.7 Hz, 1H), 3.48 (s, 2H), 2.71 (s, 2H), 2.34 (s, 2H), 1.73 (p, *J* = 7.1 Hz, 2H), 1.52 (s, 2H), 0.98 (s, 6H). **HRMS (ESI)** m/z calcd C_25_H_29_N_5_O_2_ [M+H]^+^ 432.2394, found 432.2390. **HPLC purity:** 95.8% (RT = 5.559 min).

*N^4^-(dibenzo[b,d]furan-2-yl)-N^2^-(3-thiomorpholinopropyl)pyrimidine-2,4-diamine* (**JX3243**)

White solid, 30% yield. **^1^H NMR (400 MHz, DMSO-*d*_6_)** *δ* 9.30 (s, 1H), 8.73 (s, 1H), 8.08 (s, 1H), 7.85 (d, *J* = 5.6 Hz, 1H), 7.68 (d, *J* = 8.2 Hz, 1H), 7.61 (d, *J* = 2.7 Hz, 2H), 7.55–7.49 (m, 1H), 7.40 (t, *J* = 7.5 Hz, 1H), 6.85 (t, *J* = 5.7 Hz, 1H), 6.01 (d, *J* = 5.7 Hz, 1H), 2.58 (s, 8H), 2.38 (t, *J* = 7.1 Hz, 2H), 1.72 (p, *J* = 7.0 Hz, 2H). **HRMS (ESI)** m/z calcd C_23_H_25_N_5_OS [M+H]^+^ 420.1853, found 420.1836. **HPLC purity:** 99.5% (RT = 6.704 min).

*N^4^-(dibenzo[b,d]furan-2-yl)-N^2^-(3-(piperidin-1-yl)propyl)pyrimidine-2,4-diamine* (**JX3244**)

White solid, 40% yield. **^1^H NMR (400 MHz, DMSO-*d*_6_)** *δ* 9.29 (s, 1H), 8.73 (s, 1H), 8.09 (s, 1H), 7.85 (d, *J* = 5.6 Hz, 1H), 7.68 (d, *J* = 8.2 Hz, 1H), 7.60 (s, 2H), 7.52 (t, *J* = 7.7 Hz, 1H), 7.39 (t, *J* = 7.5 Hz, 1H), 6.85 (t, *J* = 5.3 Hz, 1H), 6.01 (d, *J* = 5.7 Hz, 1H), 2.37–2.18 (m, 6H), 1.72 (t, *J* = 7.1 Hz, 2H), 1.45 (s, 4H), 1.37–1.29 (m, 2H). **^13^C NMR (101 MHz, CDCl_3_)** *δ* 162.22, 162.06, 156.86, 156.80, 153.28, 133.78, 127.51, 124.86, 123.98, 122.98, 122.78, 120.75, 115.26, 112.00, 111.80, 56.88, 54.31, 40.16, 25.77, 25.03, 23.79. **HRMS (ESI)** m/z calcd C_24_H_27_N_5_O [M+H]^+^ 402.2288, found 402.2284. **HPLC purity:** 99.7% (RT = 7.751 min).

*N^4^-(dibenzo[b,d]furan-2-yl)-N^2^-(3-(4-methylpiperidin-1-yl)propyl)pyrimidine-2,4-diamine* (**JX3245**)

White solid, 28% yield. **^1^H NMR (400 MHz, DMSO-*d*_6_)** *δ* 9.31 (s, 1H), 8.74 (s, 1H), 8.08 (s, 1H), 7.85 (d, *J* = 5.7 Hz, 1H), 7.68 (d, *J* = 8.2 Hz, 1H), 7.63–7.56 (m, 2H), 7.52 (ddd, *J* = 8.4, 7.3, 1.3 Hz, 1H), 7.39 (t, *J* = 7.5 Hz, 1H), 6.88–6.82 (m, 1H), 6.01 (d, *J* = 5.7 Hz, 1H), 2.84 (s, 2H), 2.38 (s, 2H), 1.87 (s, 2H), 1.74 (t, *J* = 7.2 Hz, 2H), 1.53 (s, 2H), 1.37–1.19 (m, 3H), 1.09 (s, 2H), 0.82 (d, *J* = 6.5 Hz, 3H). **^13^C NMR (101 MHz, CDCl_3_)** *δ* 162.27, 162.05, 156.89, 156.78, 153.19, 133.91, 127.47, 124.82, 124.00, 122.87, 122.75, 120.73, 115.13, 111.96, 111.78, 94.57, 56.76, 53.94, 40.33, 33.83, 30.61, 26.37, 21.70. **HRMS (ESI)** m/z calcd C_25_H_29_N_5_O [M+H]^+^ 416.2445, found 416.2438. **HPLC purity:** 98.1% (RT = 8.440 min).

*N^4^-(dibenzo[b,d]furan-2-yl)-N^2^-(3-(4-methoxypiperidin-1-yl)propyl)pyrimidine-2,4-diamine* (**JX3246**)

White solid, 30% yield. **^1^H NMR (400 MHz, DMSO-*d*_6_)** *δ* 9.30 (s, 1H), 8.72 (s, 1H), 8.08 (s, 1H), 7.85 (d, *J* = 5.7 Hz, 1H), 7.68 (d, *J* = 8.2 Hz, 1H), 7.61 (s, 2H), 7.56–7.48 (m, 1H), 7.40 (t, *J* = 7.5 Hz, 1H), 6.86 (s, 1H), 6.01 (d, *J* = 5.7 Hz, 1H), 3.35 (s, 2H), 3.18 (s, 3H), 3.09 (s, 1H), 2.64 (s, 2H), 2.33 (t, *J* = 7.1 Hz, 2H), 1.98 (s, 2H), 1.72 (q, *J* = 7.3 Hz, 4H), 1.42–1.30 (m, 2H). **^13^C NMR (101 MHz, CDCl_3_)** *δ* 162.40, 162.06, 157.14, 156.78, 153.20, 133.94, 127.47, 124.82, 124.00, 122.88, 122.74, 120.70, 115.11, 111.99, 111.79, 94.47, 56.68, 55.49, 51.12, 40.54, 30.81, 29.70, 26.65. **HRMS (ESI)** m/z calcd C_25_H_29_N_5_O_2_ [M+H]^+^ 432.2394, found 432.2390. **HPLC purity:** 98.4% (RT = 5.331 min).

*N^4^-(dibenzo[b,d]furan-2-yl)-N^2^-(3-(3,5-dimethylpiperidin-1-yl)propyl)pyrimidine-2,4-diamine* (**JX3247**)

White solid, 30% yield. **^1^H NMR (400 MHz, DMSO-*d*_6_)** *δ* 9.33 (s, 1H), 8.72 (s, 1H), 8.04 (d, *J* = 5.8 Hz, 1H), 7.86 (d, *J* = 5.7 Hz, 1H), 7.68 (d, *J* = 8.2 Hz, 1H), 7.60 (d, *J* = 1.9 Hz, 2H), 7.52 (ddd, *J* = 8.4, 7.2, 1.4 Hz, 1H), 7.39 (t, *J* = 7.5 Hz, 1H), 6.89 (s, 1H), 6.02 (d, *J* = 5.7 Hz, 1H), 2.33 (s, 6H), 1.87–1.72 (m, 4H), 1.23 (s, 2H), 0.88 (s, 6H). **^13^C NMR (101 MHz, CDCl_3_)** *δ* 162.19, 161.55, 156.82, 140.22, 136.28, 135.96, 135.21, 134.71, 126.98, 124.42, 123.12, 122.96, 121.91, 121.65, 115.02, 60.72, 56.51, 41.39, 40.24, 30.21, 25.68, 19.32. **HRMS (ESI)** m/z calcd C_26_H_31_N_5_O [M+H]^+^ 430.2601, found 430.2602. **HPLC purity:** 95.1% (RT = 13.663 min).

*N^4^-(dibenzo[b,d]furan-2-yl)-N^2^-(3-(4-(oxetan-3-yl)piperazin-1-yl)propyl)pyrimidine-2,4-diamine* (**JX3248**)

White solid, 30% yield. **^1^H NMR (400 MHz, DMSO-*d*_6_)** *δ* 9.28 (s, 1H), 8.71 (s, 1H), 8.09 (s, 1H), 7.85 (d, *J* = 5.7 Hz, 1H), 7.68 (d, *J* = 8.2 Hz, 1H), 7.64–7.56 (m, 2H), 7.55–7.49 (m, 1H), 7.39 (t, *J* = 7.5 Hz, 1H), 6.82 (s, 1H), 6.01 (d, *J* = 5.7 Hz, 1H), 4.45 (d, *J* = 6.5 Hz, 2H), 4.36 (t, *J* = 6.0 Hz, 2H), 2.44–2.11 (m, 10H), 1.76–1.68 (m, 2H). **HRMS (ESI)** m/z calcd C_26_H_30_N_6_O_2_ [M+H]^+^ 459.2503, found 459.2504. **HPLC purity:** 95.1% (RT = 4.839 min).

*N^2^-(3-(azepan-1-yl)propyl)-N^4^-(dibenzo[b,d]furan-2-yl)pyrimidine-2,4-diamine* (**JX3249**)

White solid, 28% yield. **^1^H NMR (400 MHz, DMSO-*d*_6_)** *δ* 9.31 (s, 1H), 8.74 (s, 1H), 8.07 (s, 1H), 7.87 (dd, *J* = 5.8, 2.9 Hz, 1H), 7.68 (d, *J* = 8.3 Hz, 1H), 7.61 (d, *J* = 1.9 Hz, 2H), 7.52 (ddd, *J* = 8.4, 7.3, 1.4 Hz, 1H), 7.39 (t, *J* = 7.5 Hz, 1H), 6.88 (t, *J* = 5.6 Hz, 1H), 6.02 (d, *J* = 5.7 Hz, 1H), 3.64–3.39 (m, 6H), 2.54 (s, 2H), 1.71 (p, *J* = 7.0 Hz, 2H), 1.50 (d, *J* = 18.6 Hz, 8H). **^13^C NMR (101 MHz, CDCl_3_)** *δ* 162.45, 162.03, 157.22, 156.80, 153.22, 133.92, 127.48, 124.84, 124.02, 122.91, 122.75, 120.72, 115.17, 111.97, 111.80, 94.45, 56.61, 55.68, 40.74, 29.70, 27.82, 26.93. **HRMS (ESI)** m/z calcd C_25_H_29_N_5_O [M+H]^+^ 416.2445, found 416.2442. **HPLC purity:** 95.7% (RT = 8.661 min).

*N^2^-(3-(1,4-oxazepan-4-yl)propyl)-N^4^-(dibenzo[b,d]furan-2-yl)pyrimidine-2,4-diamine* (**JX3250**)

White solid, 26% yield. **^1^H NMR (400 MHz, DMSO-*d*_6_)** *δ* 9.30 (s, 1H), 8.73 (s, 1H), 8.08 (s, 1H), 7.85 (d, *J* = 5.7 Hz, 1H), 7.68 (d, *J* = 8.2 Hz, 1H), 7.64–7.56 (m, 2H), 7.56–7.48 (m, 1H), 7.40 (t, *J* = 7.5 Hz, 1H), 6.87 (t, *J* = 5.7 Hz, 1H), 6.01 (d, *J* = 5.7 Hz, 1H), 3.61 (s, 4H), 2.57 (d, *J* = 26.5 Hz, 6H), 1.74 (q, *J* = 10.2 Hz, 4H), 1.24 (d, *J* = 11.3 Hz, 2H). **HRMS (ESI)** m/z calcd C_24_H_27_N_5_O_2_ [M+H]^+^ 418.2238, found 418.2237. **HPLC purity:** 98.7% (RT = 5.684 min).

**NMR spectra-^1^H**

^1^H spectrum of compound **JX3201** (DMSO-*d_6_*)


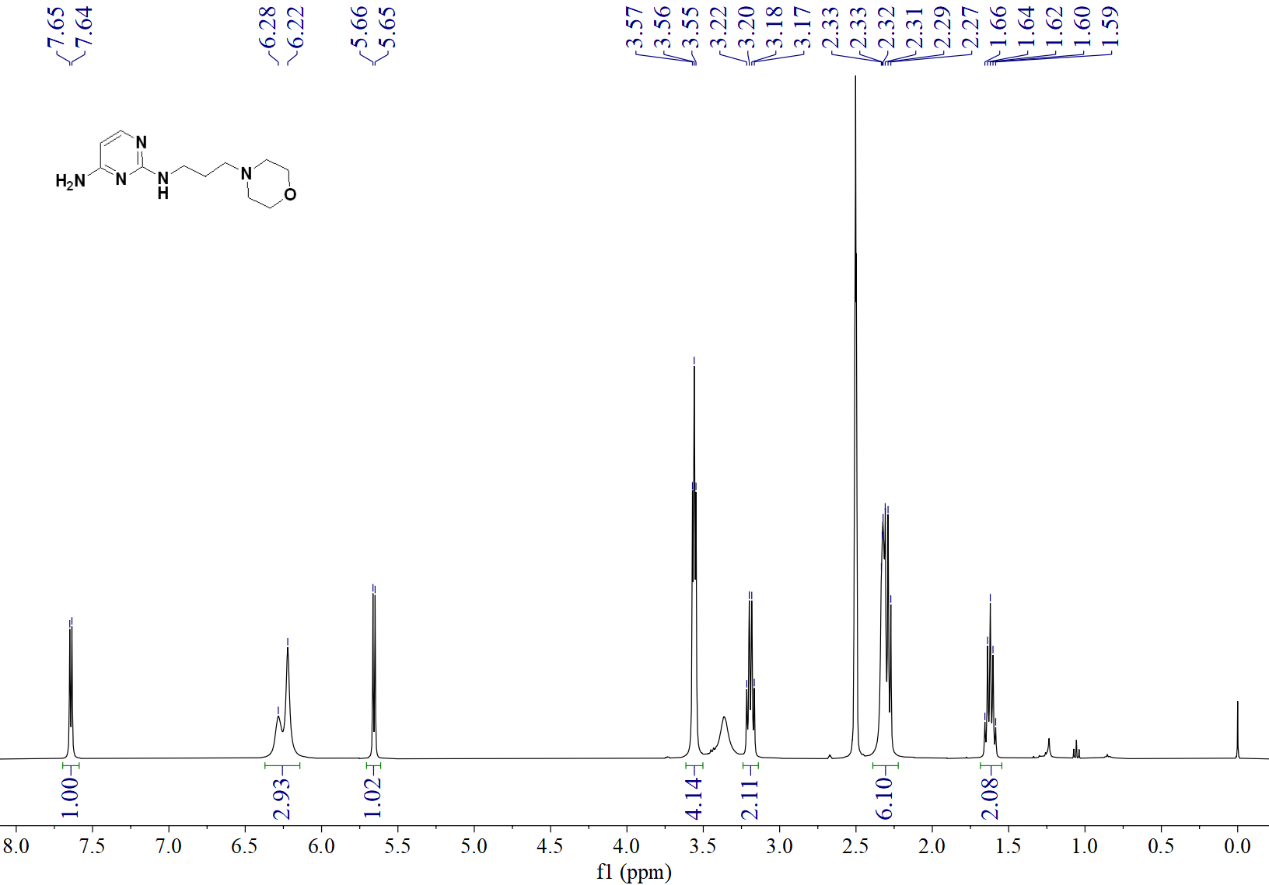


^1^H spectrum of compound **JX3202a** (DMSO-*d_6_*)


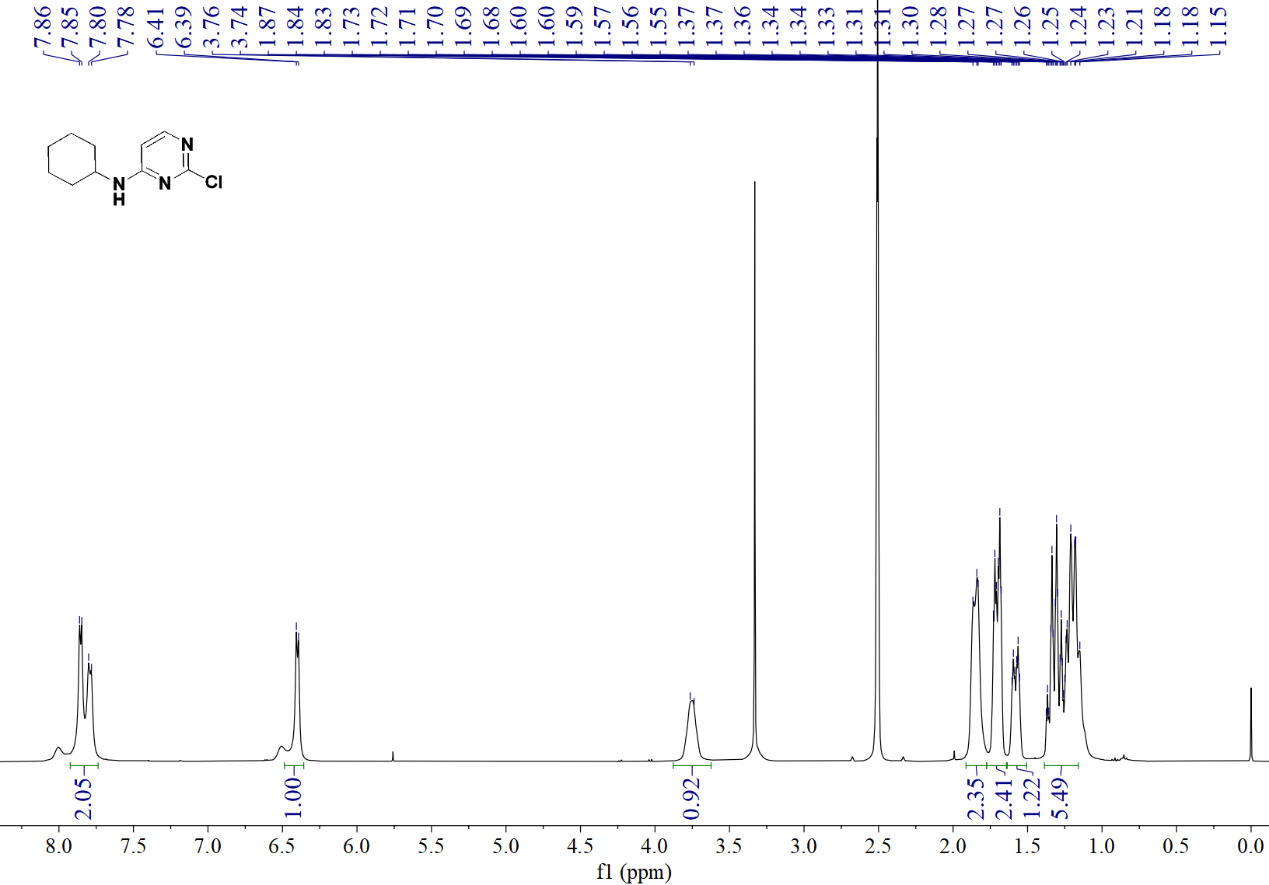


^1^H spectrum of compound **JX3202** (DMSO-*d_6_*)


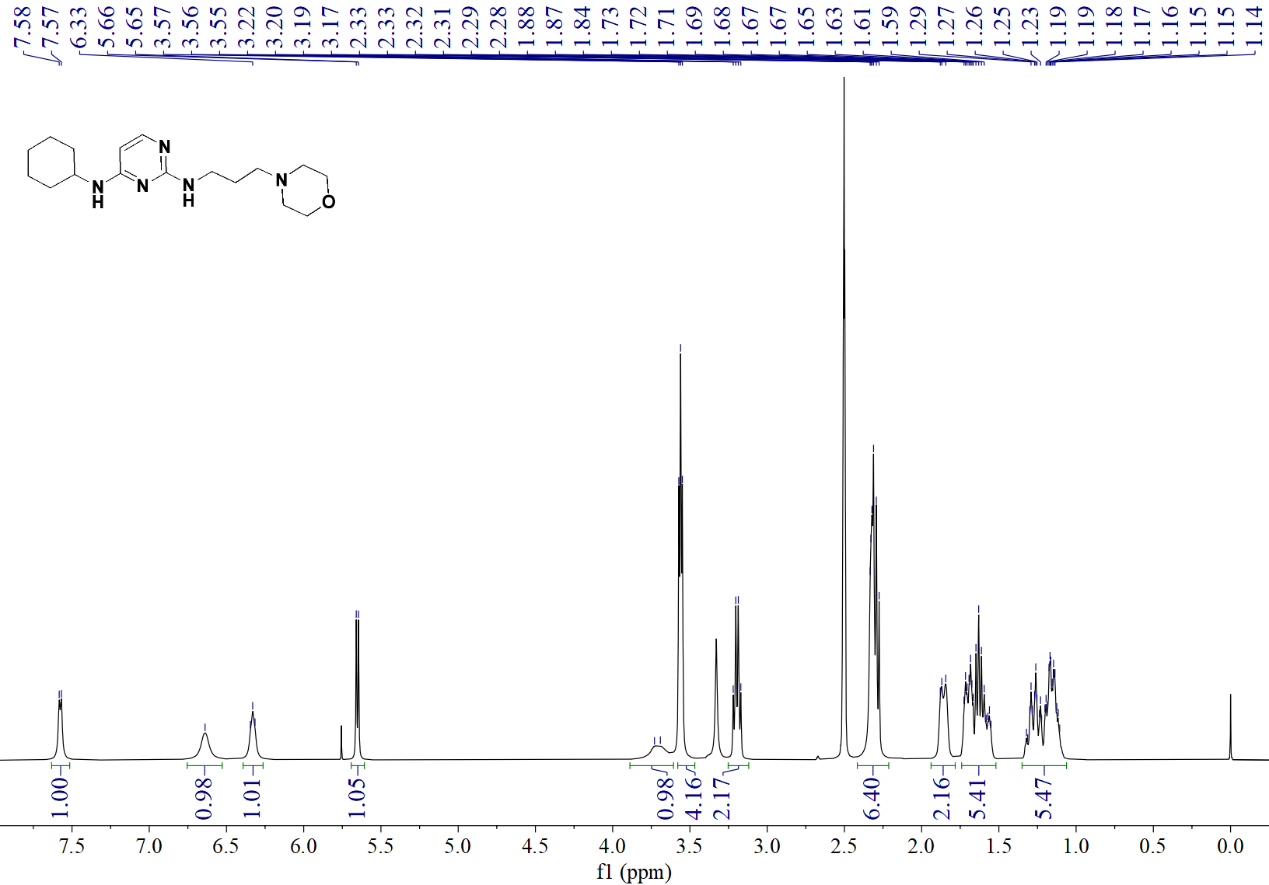


^1^H spectrum of compound **JX3203a** (DMSO-*d_6_*)


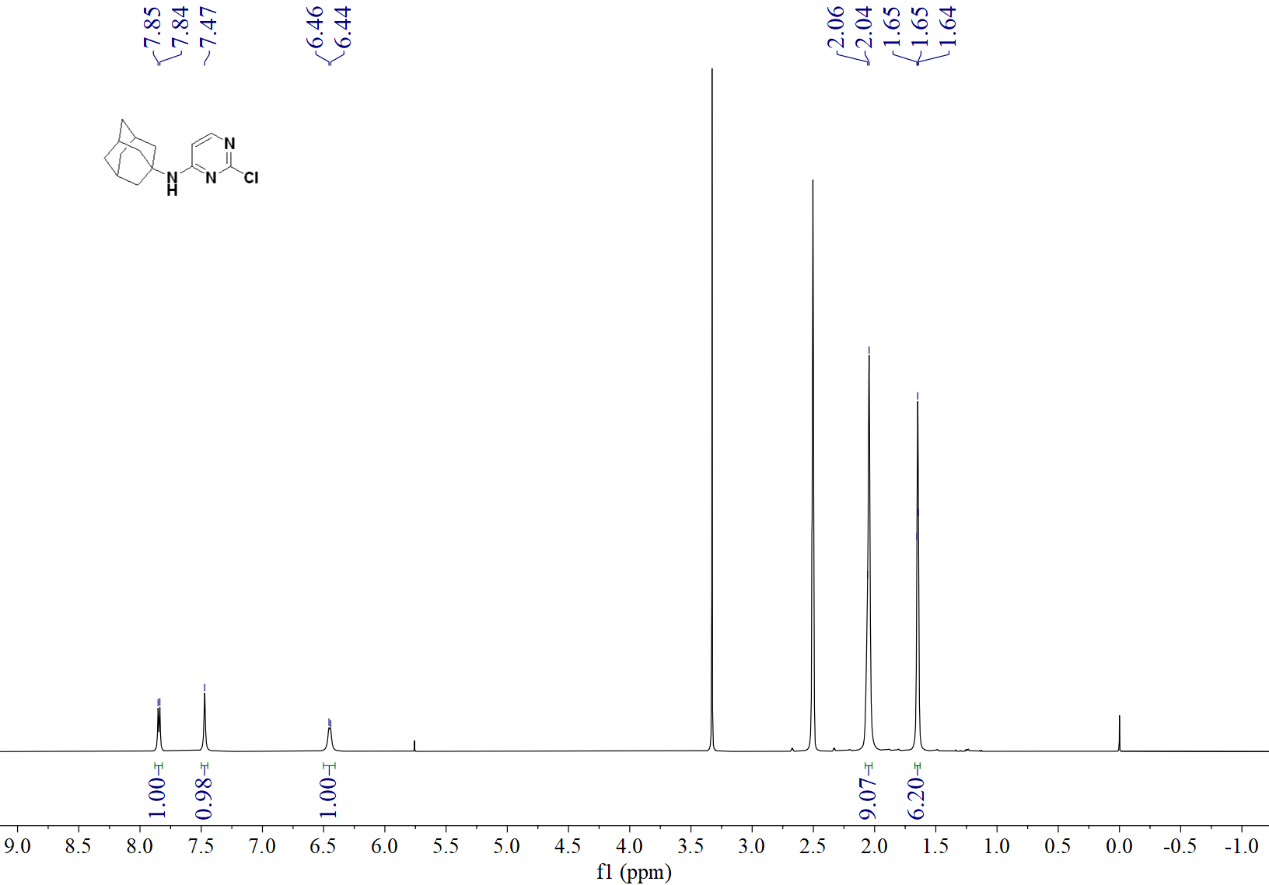


^1^H spectrum of compound **JX3203** (DMSO-*d_6_*)


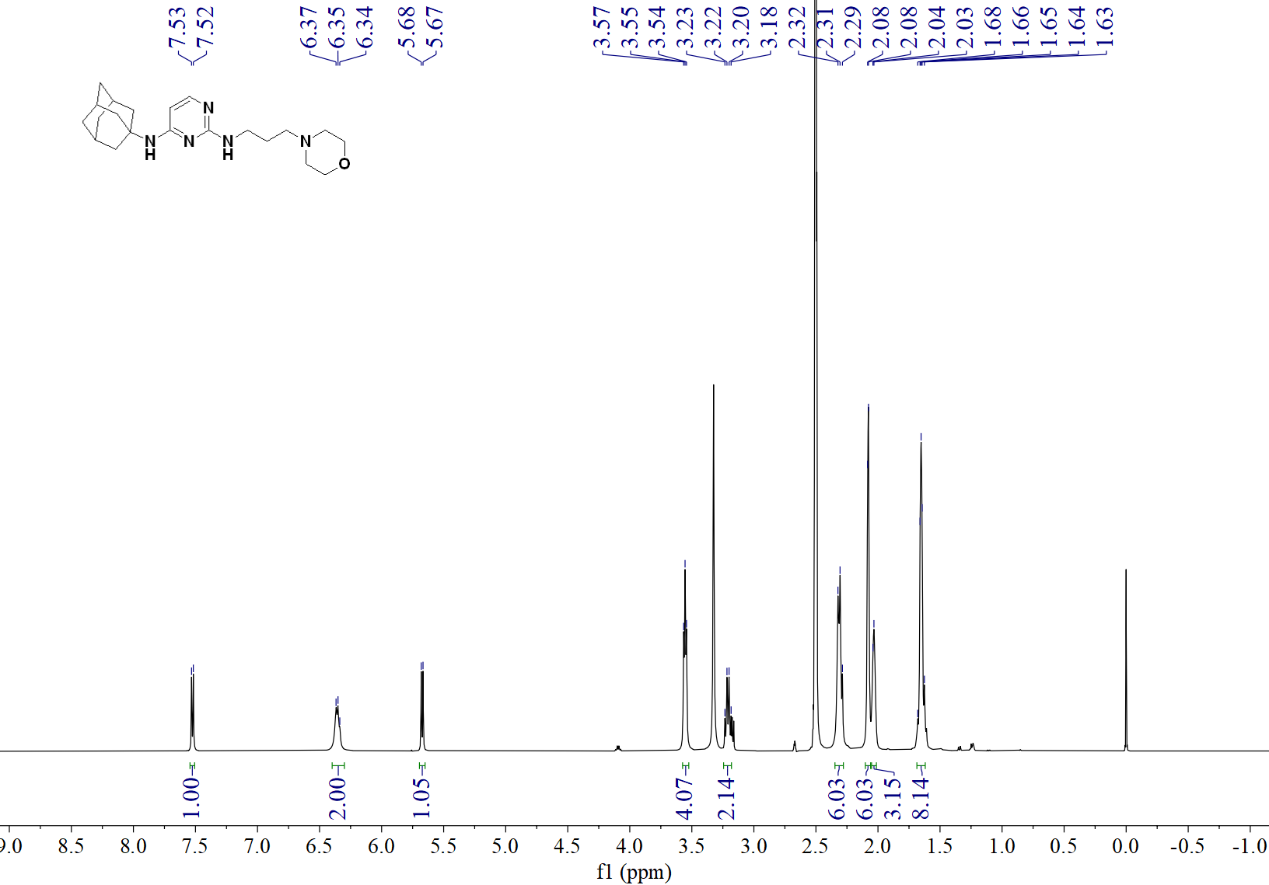


^1^H spectrum of compound **JX3204a** (DMSO-*d_6_*)


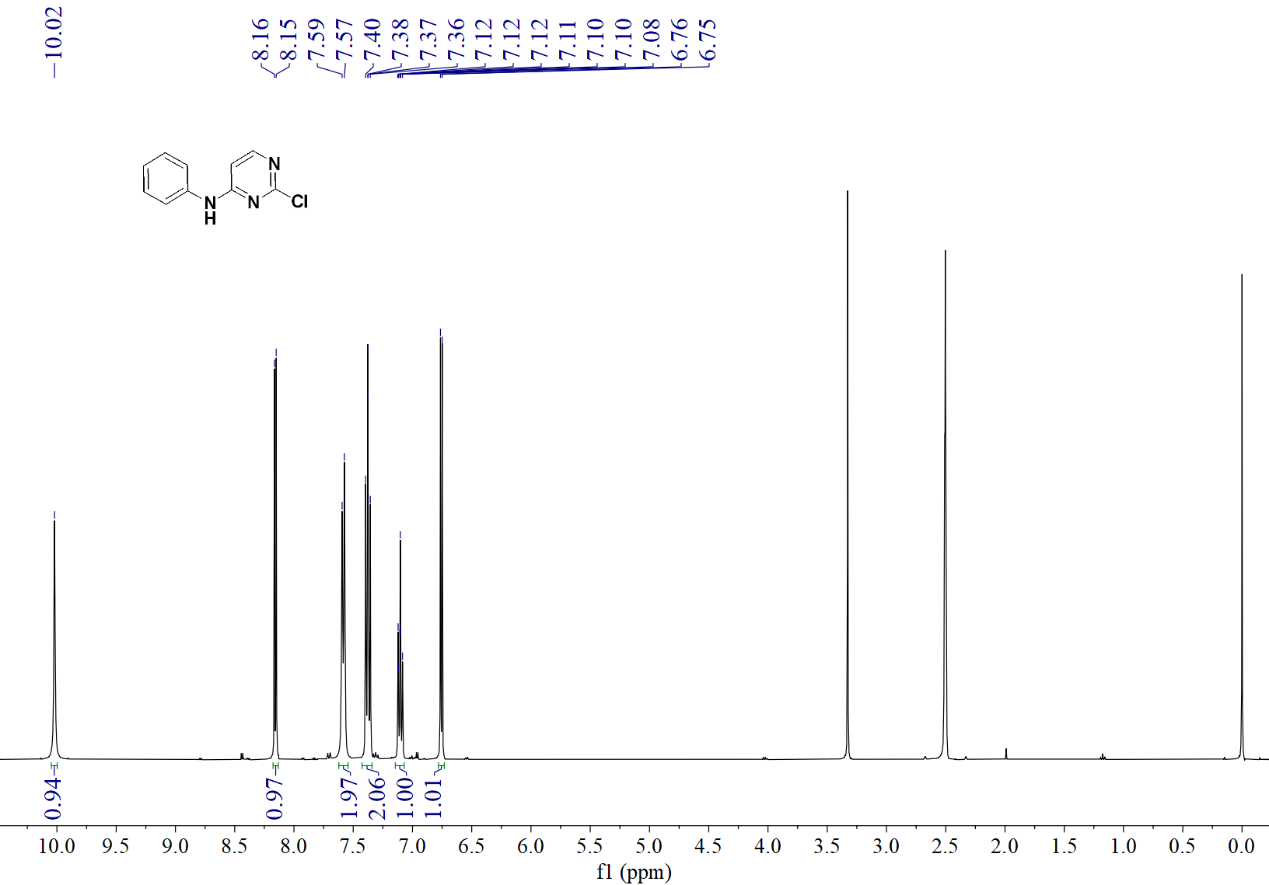


^1^H spectrum of compound **JX3204** (DMSO-*d_6_*)


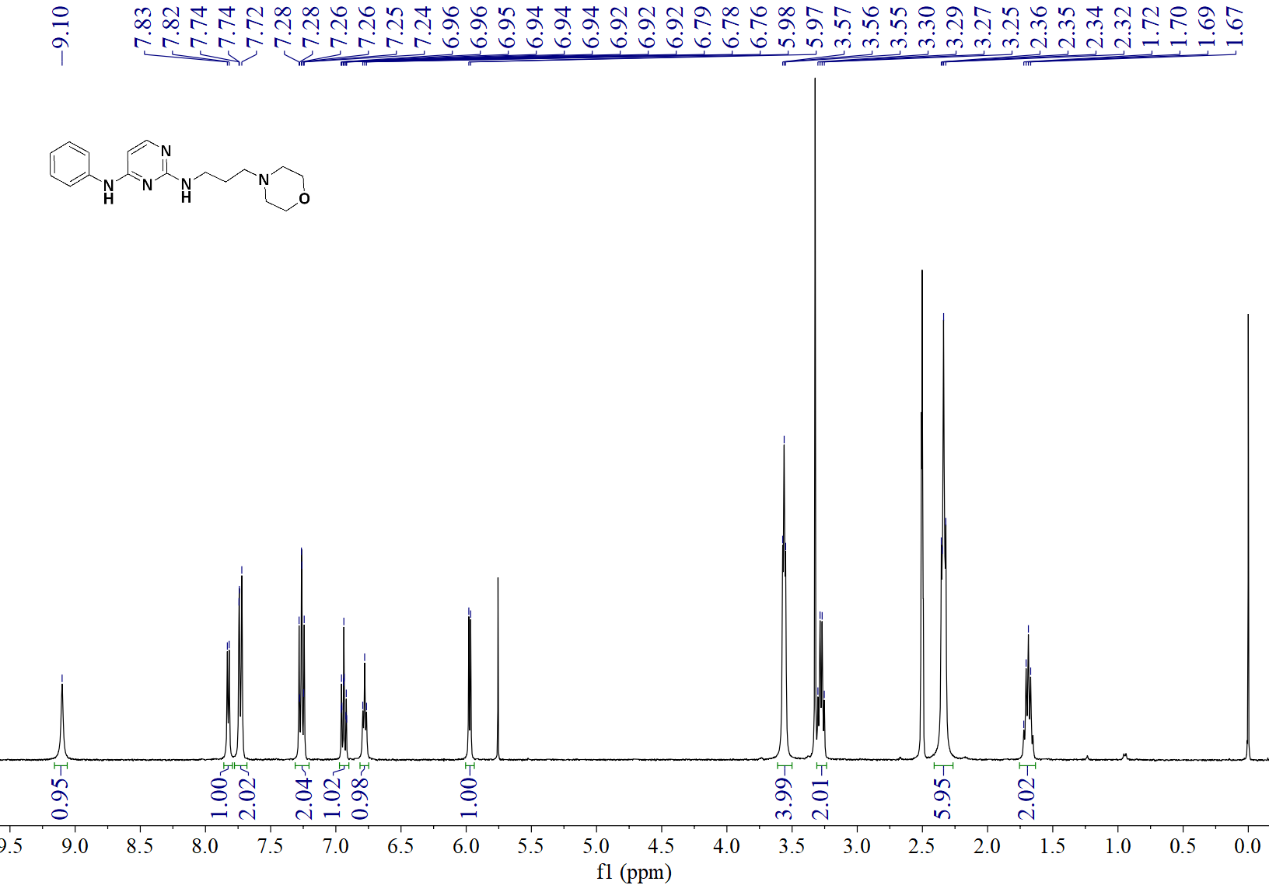


^1^H spectrum of compound **JX3205a** (DMSO-*d_6_*)


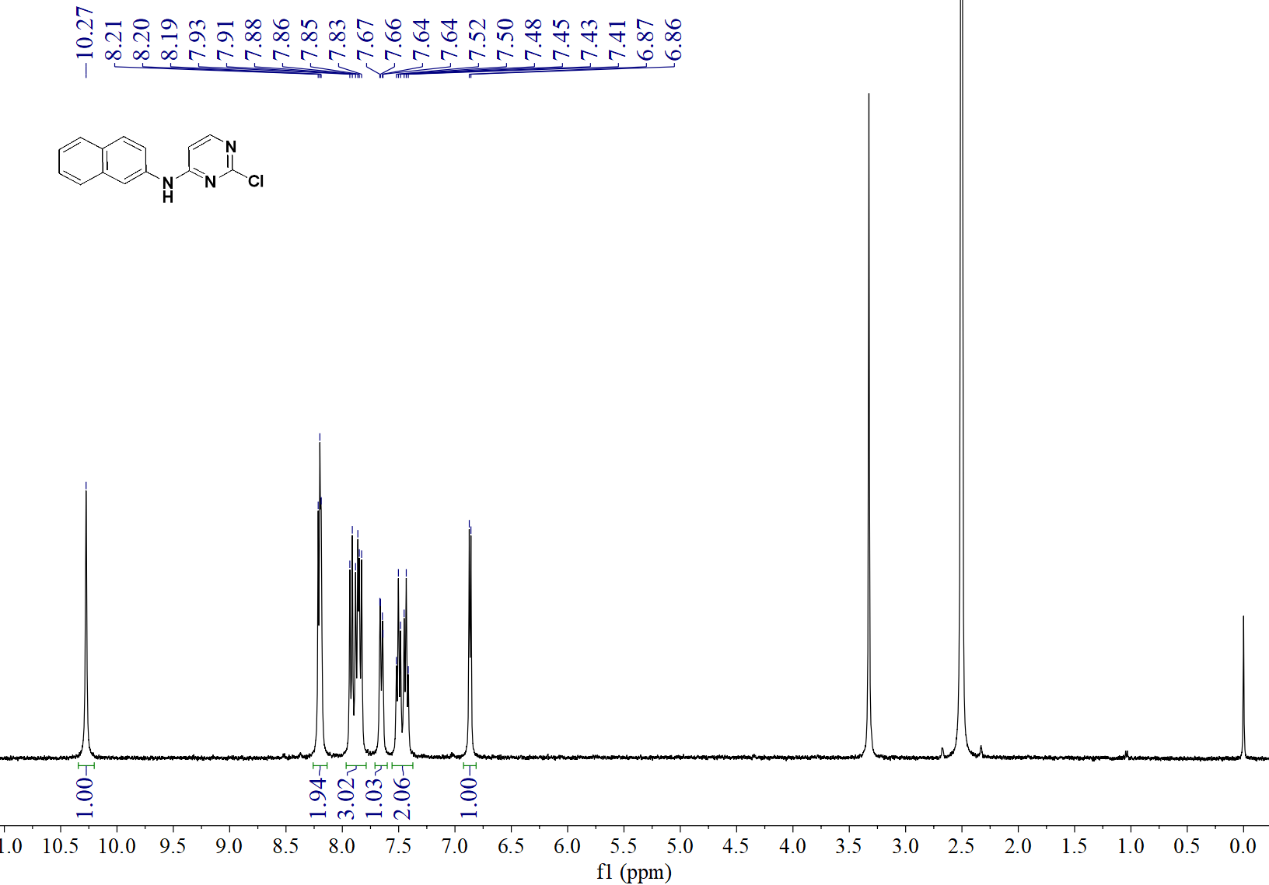


^1^H spectrum of compound **JX3205** (DMSO-*d_6_*)


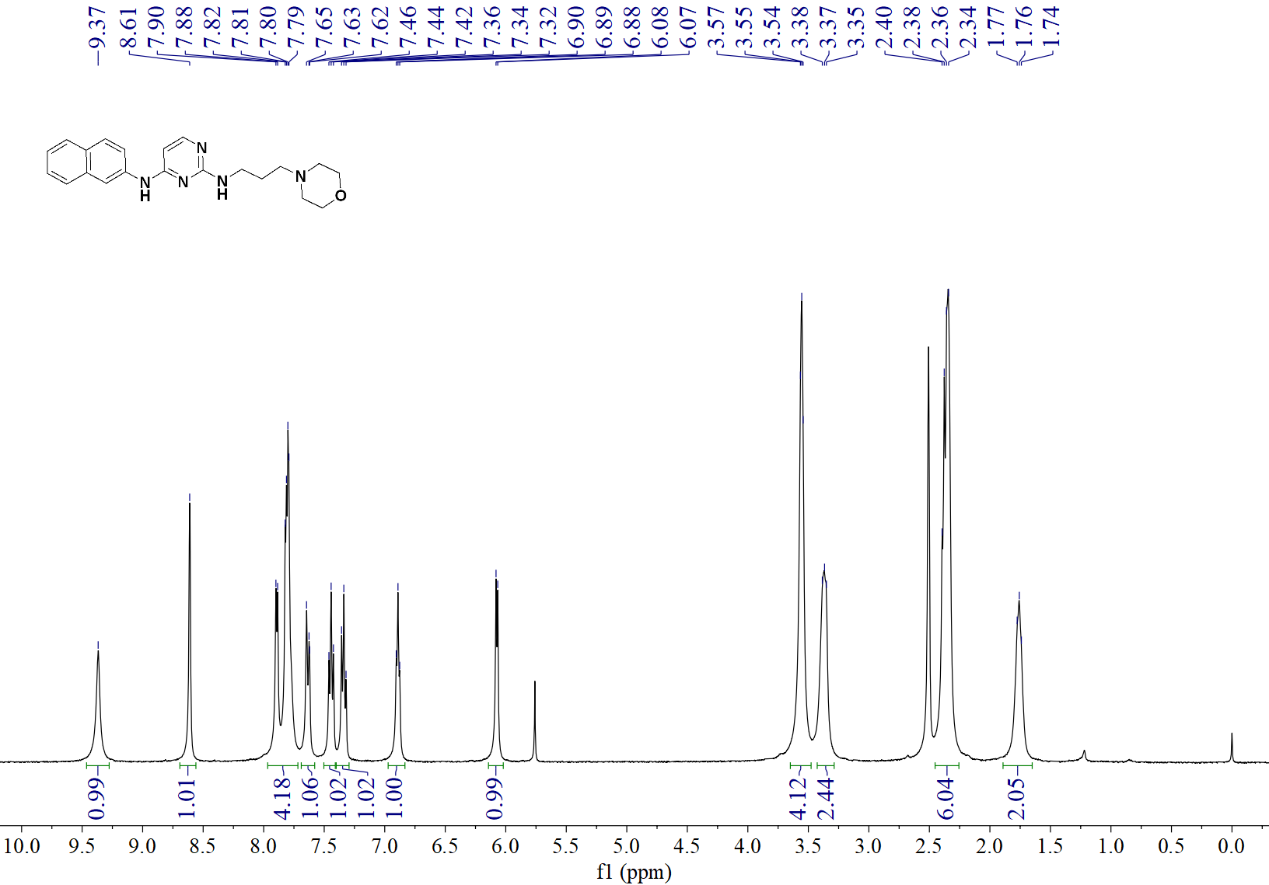


^1^H spectrum of compound **JX3206a** (DMSO-*d_6_*)


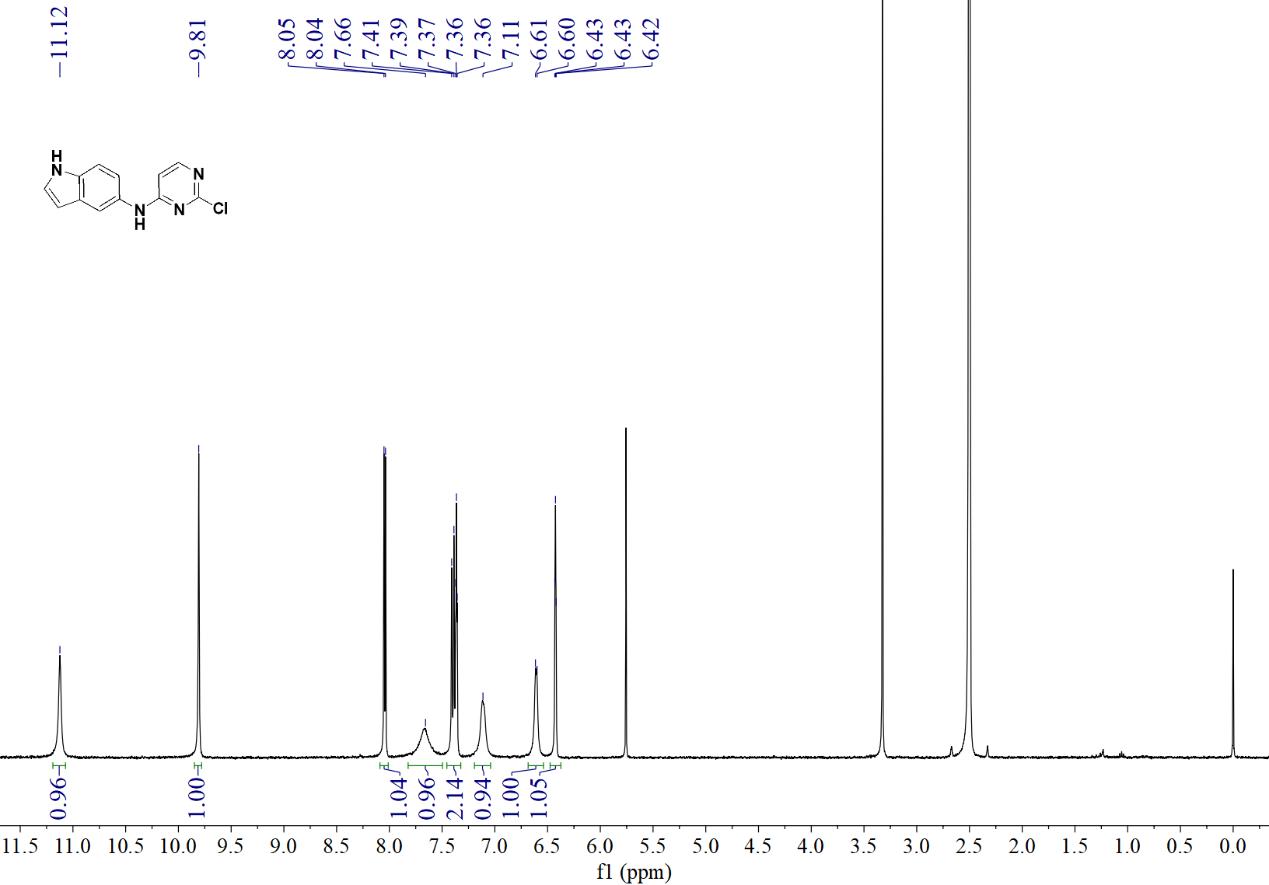


^1^H spectrum of compound **JX3206** (DMSO-*d_6_*)


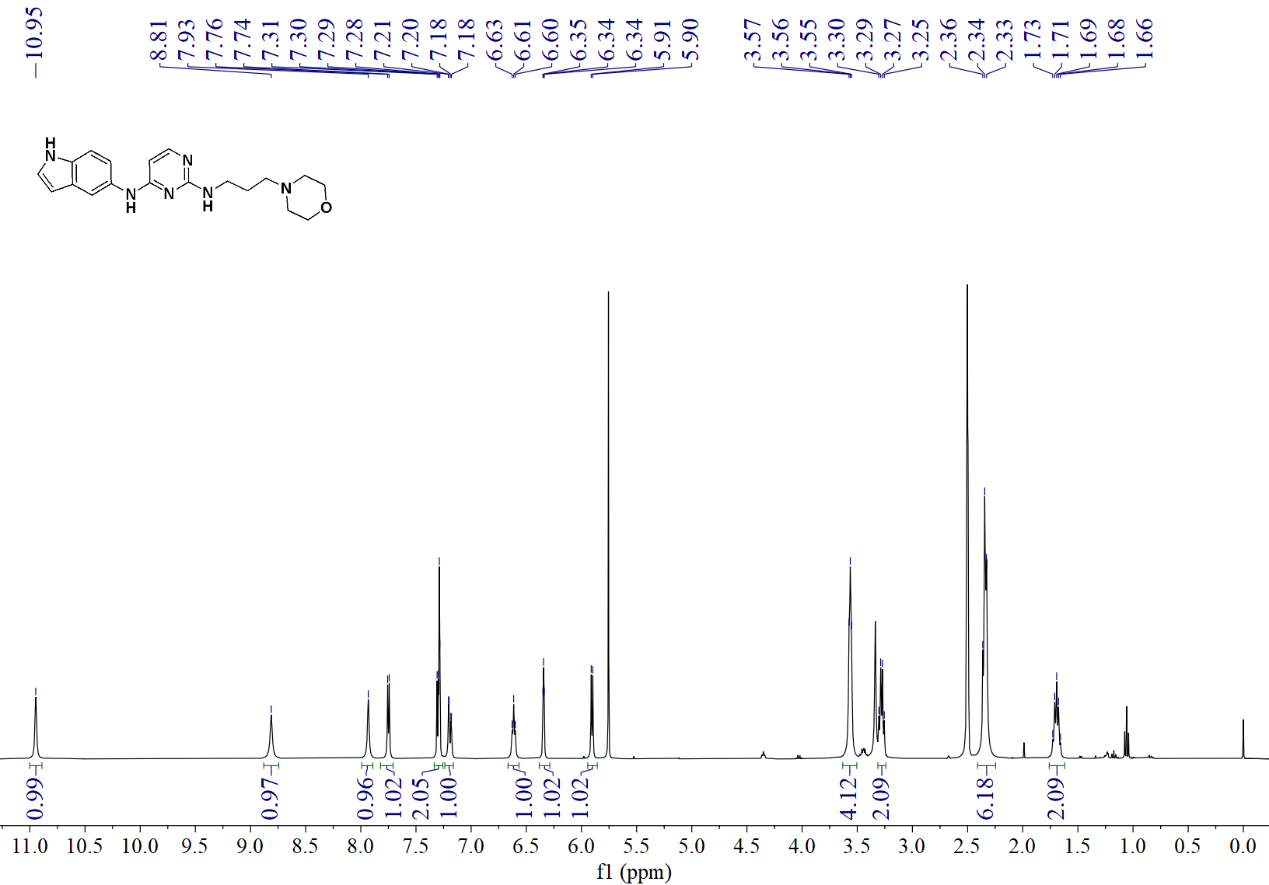


^1^H spectrum of compound **JX3207a** (DMSO-*d_6_*)


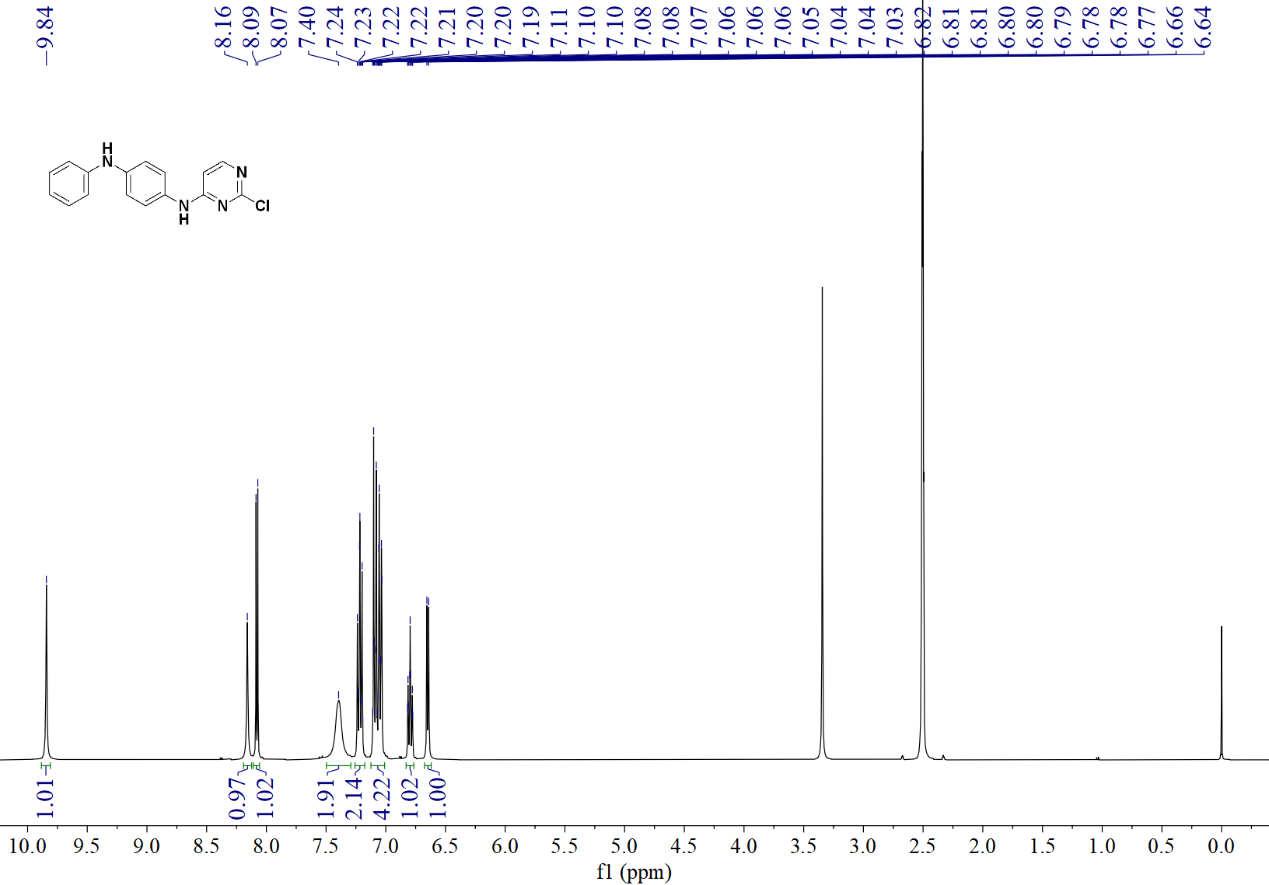


^1^H spectrum of compound **JX3207** (DMSO-*d_6_*)


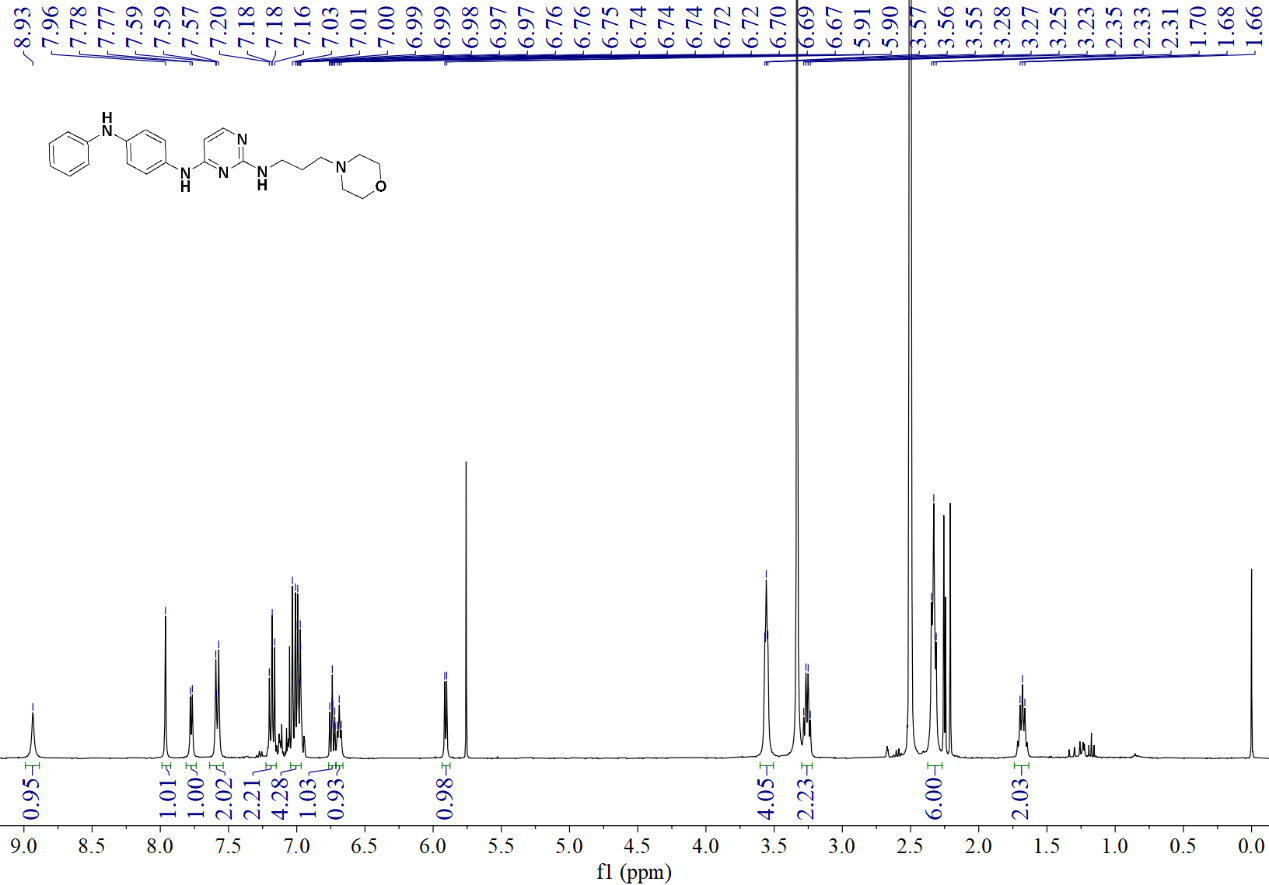


^1^H spectrum of compound **JX3208a** (DMSO-*d_6_*)


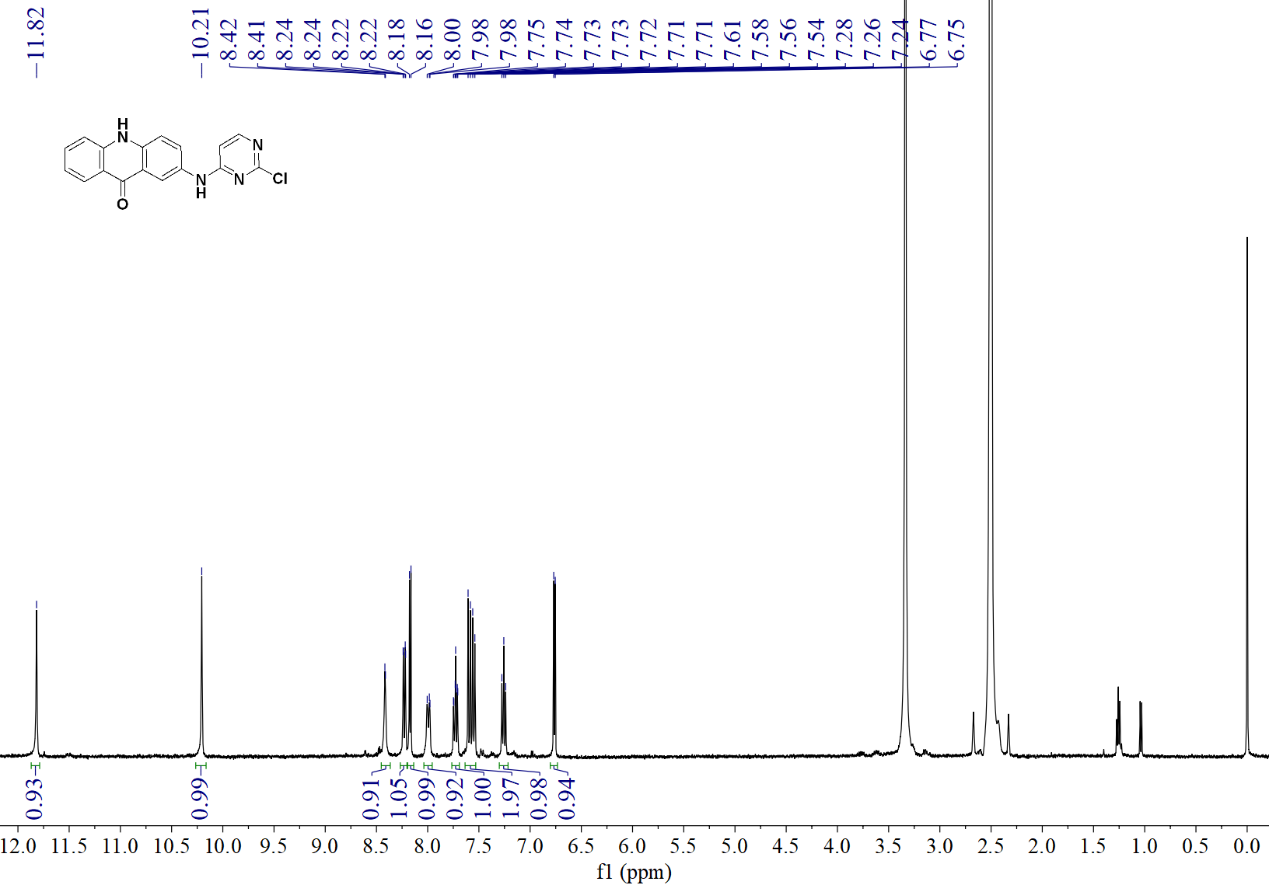


^1^H spectrum of compound **JX3208** (DMSO-*d_6_*)


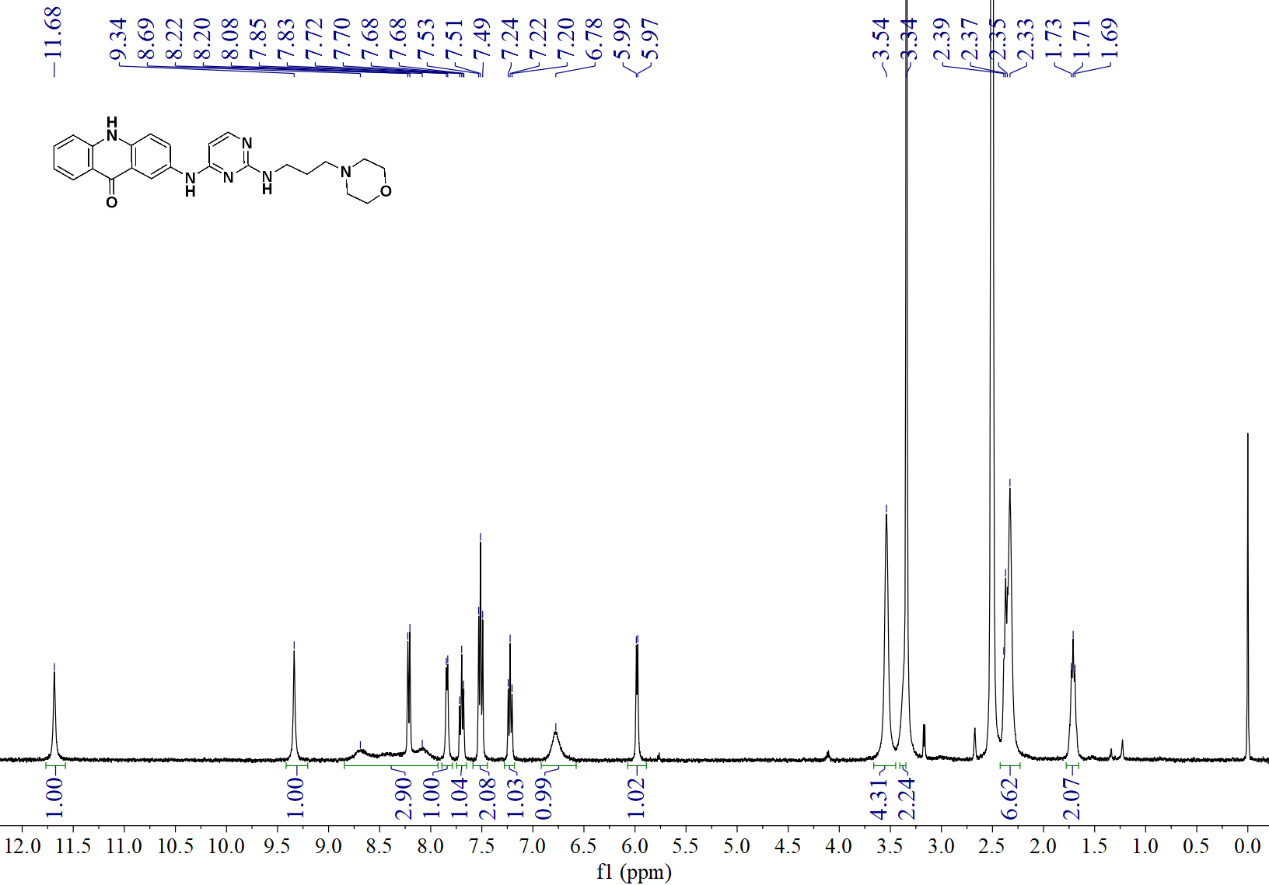


^1^H spectrum of compound **JX3209a** (DMSO-*d_6_*)


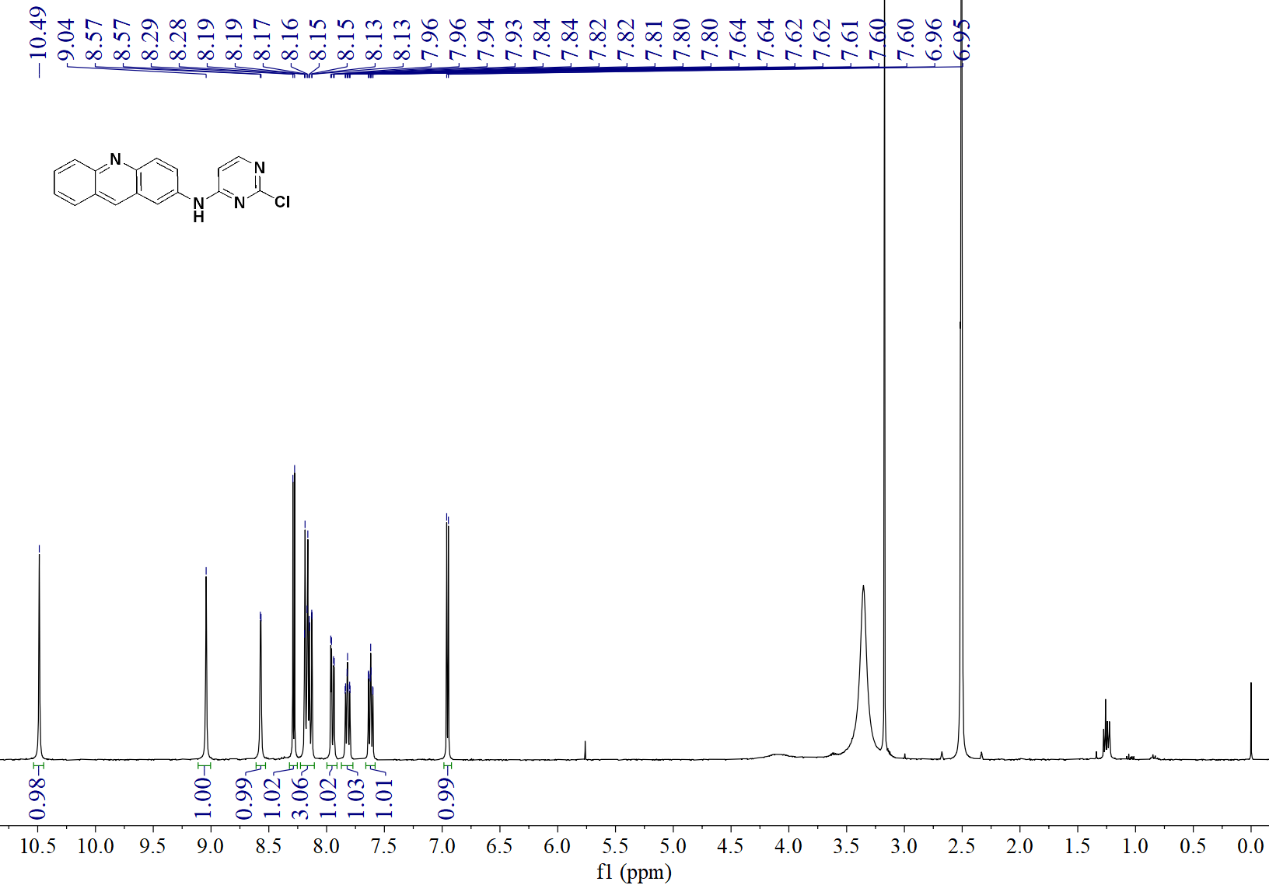


^1^H spectrum of compound **JX3209** (DMSO-*d_6_*)


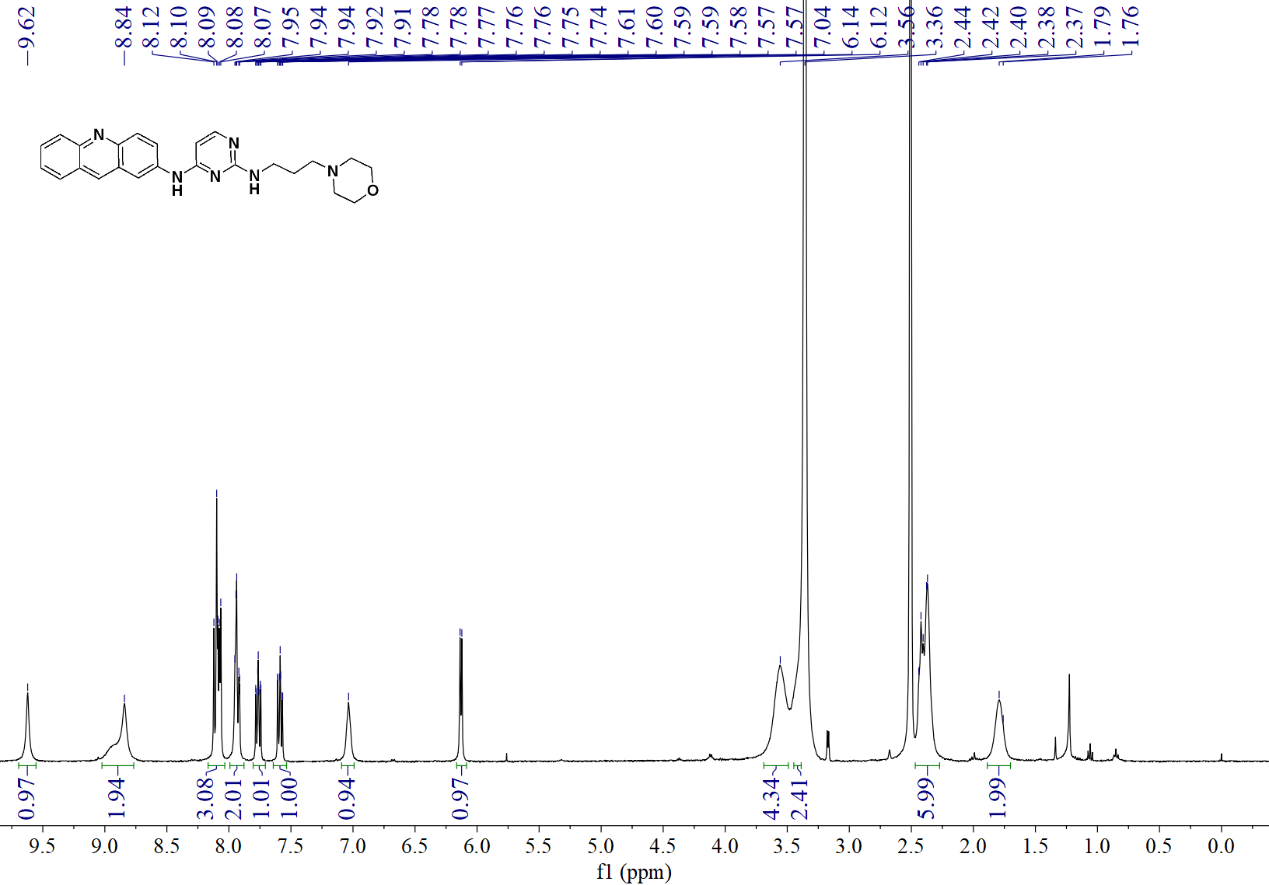


^1^H spectrum of compound **JX3210a** (DMSO-*d_6_*)


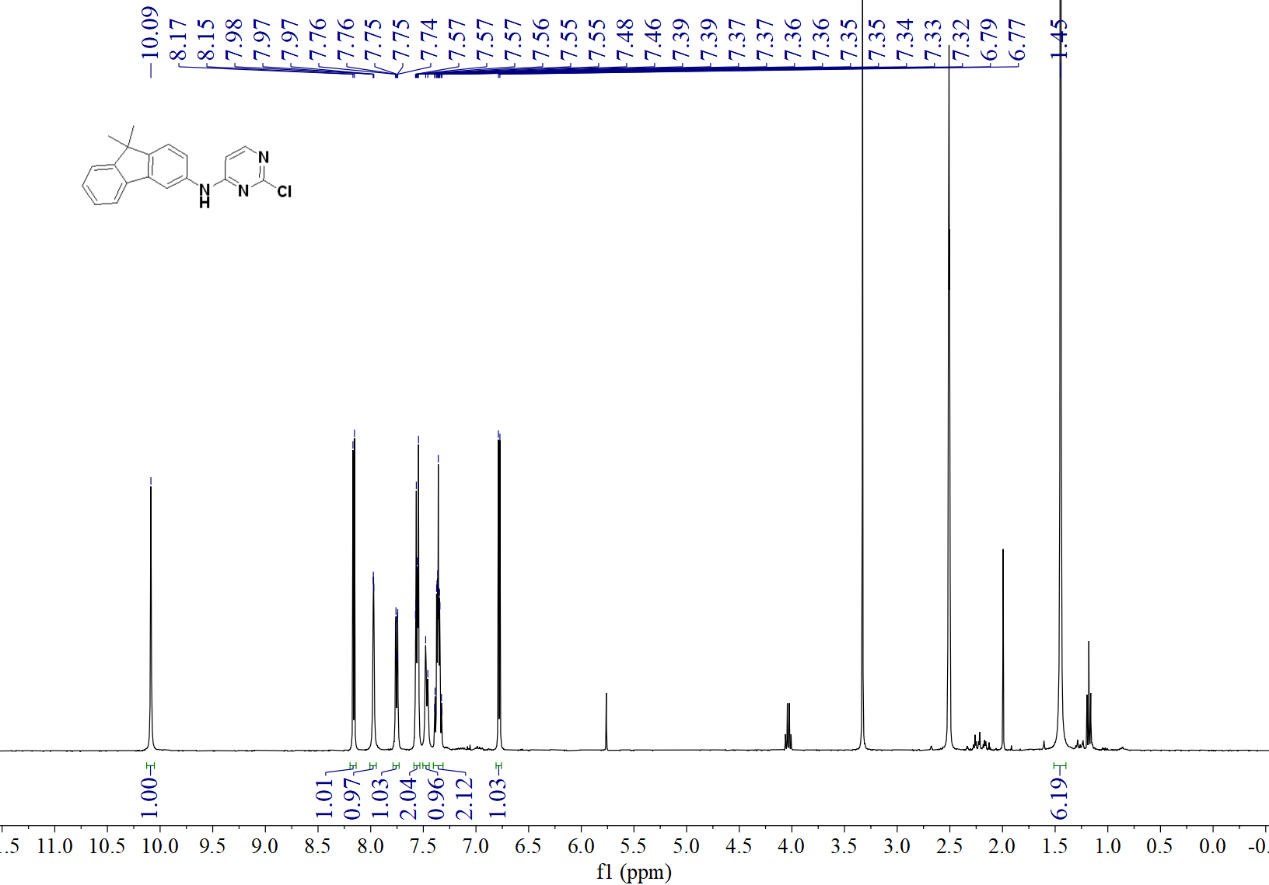


^1^H spectrum of compound **JX3210** (DMSO-*d_6_*)


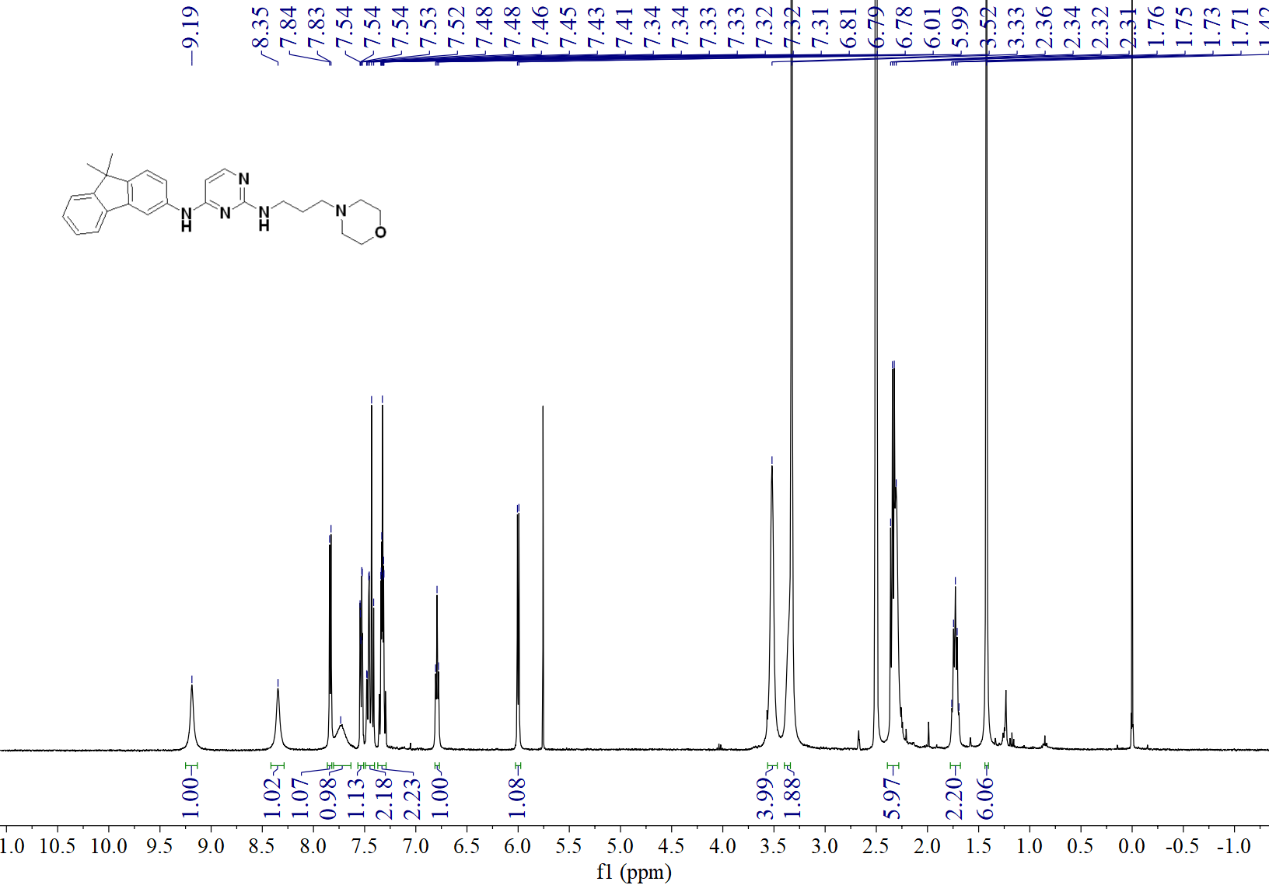


^1^H spectrum of compound **JX3211** (DMSO-*d_6_*)


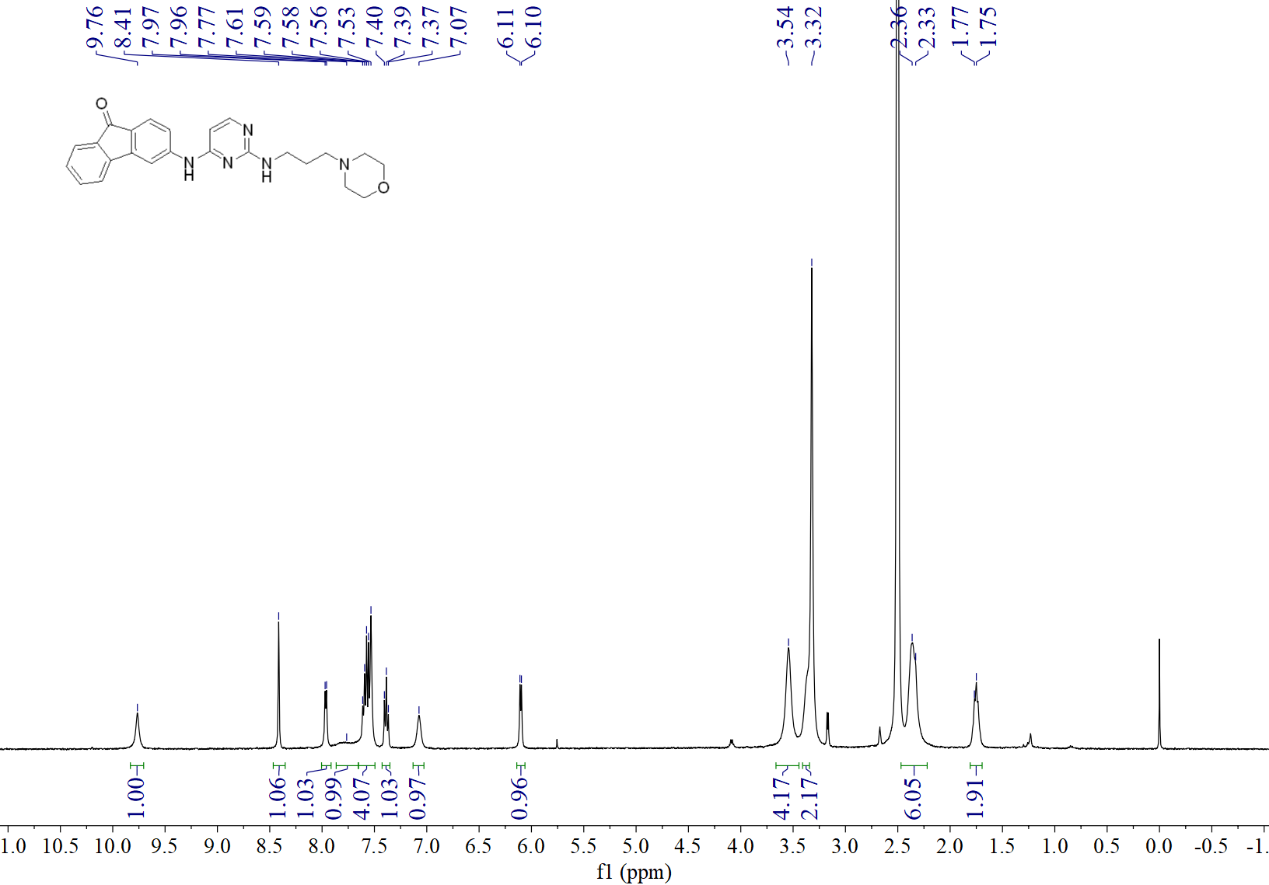


^1^H spectrum of compound **JX3212a** (DMSO-*d_6_*)


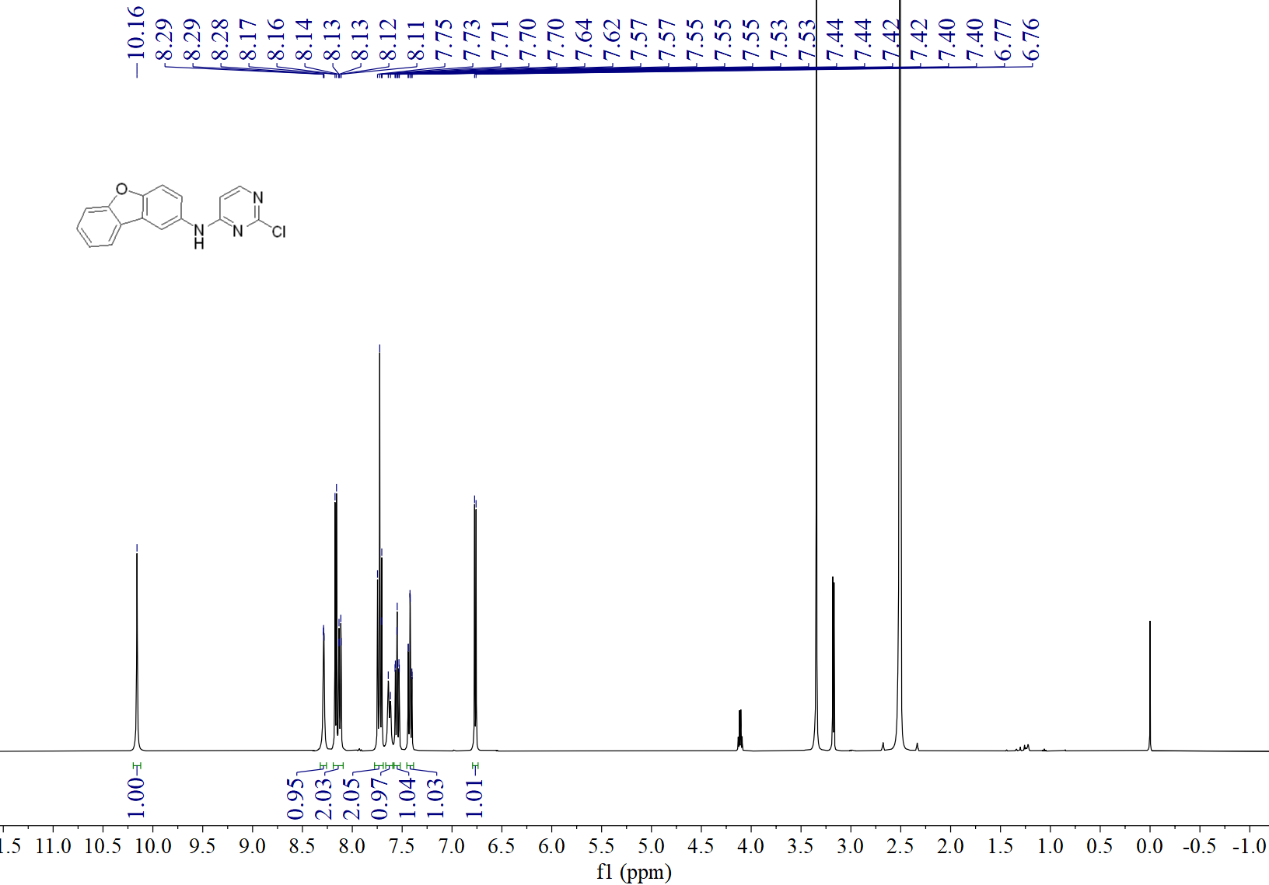


^1^H spectrum of compound **JX3212** (DMSO-*d_6_*)


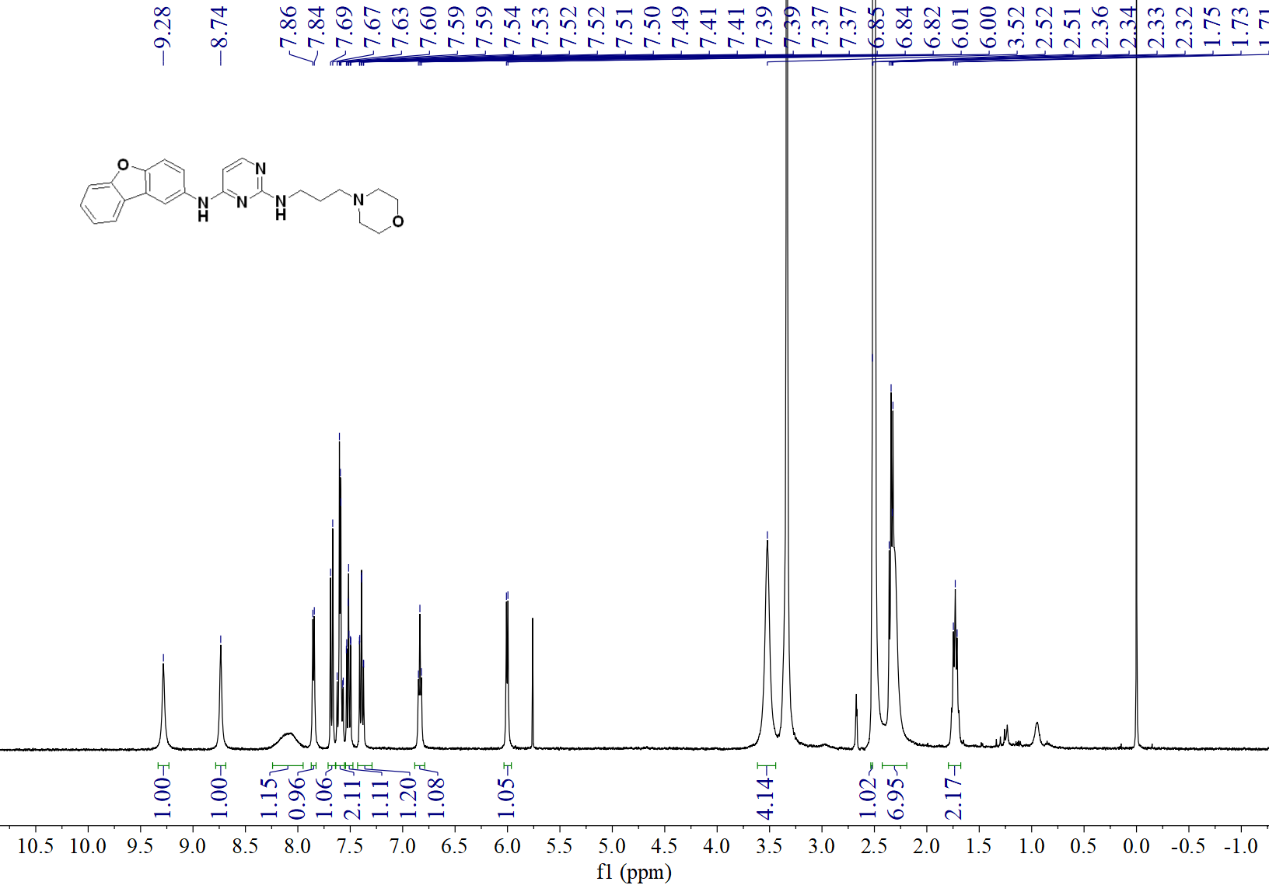


^1^H spectrum of compound **JX3213a** (CDCl_3_)


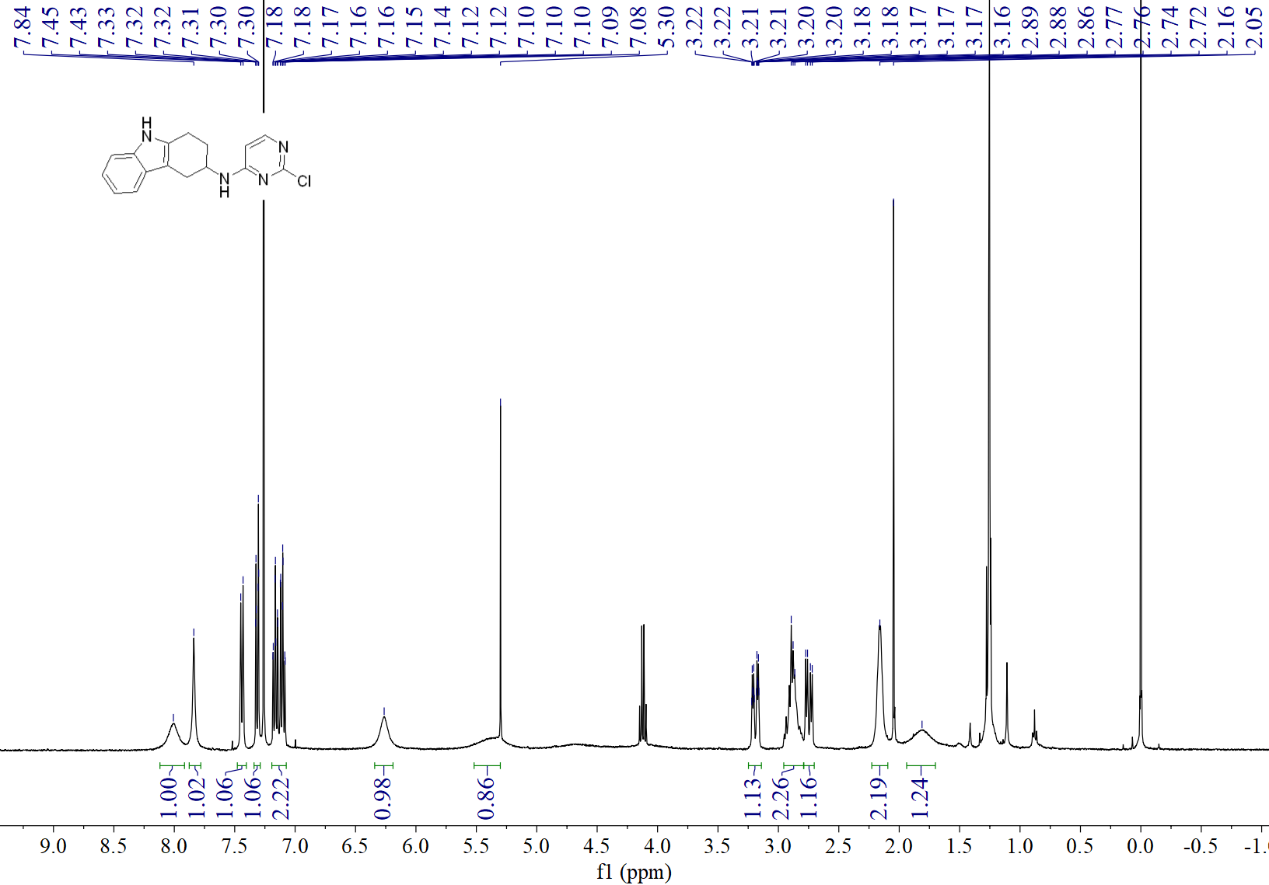


^1^H spectrum of compound **JX3213** (DMSO-*d_6_*)


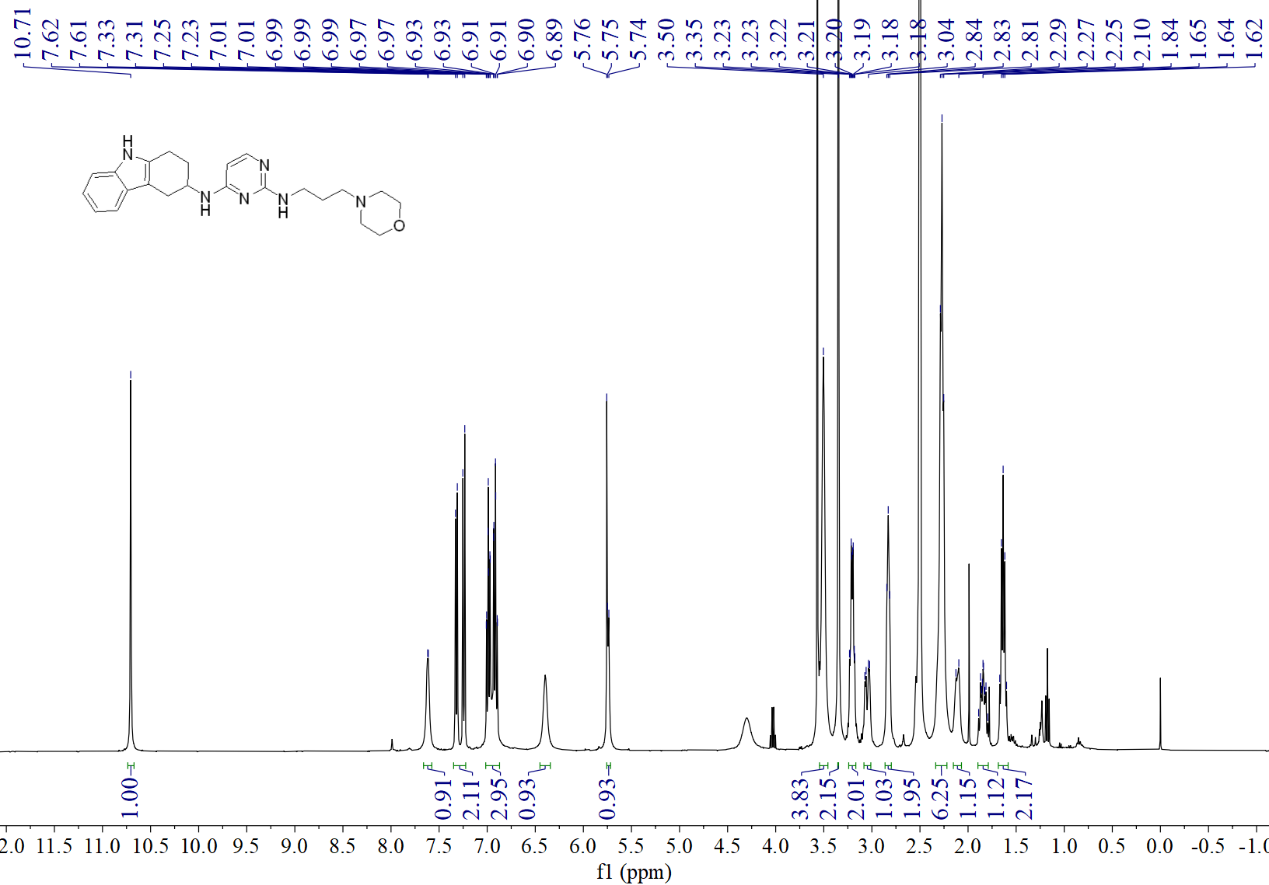


^1^H spectrum of compound **JX3214a** (DMSO-*d_6_*)


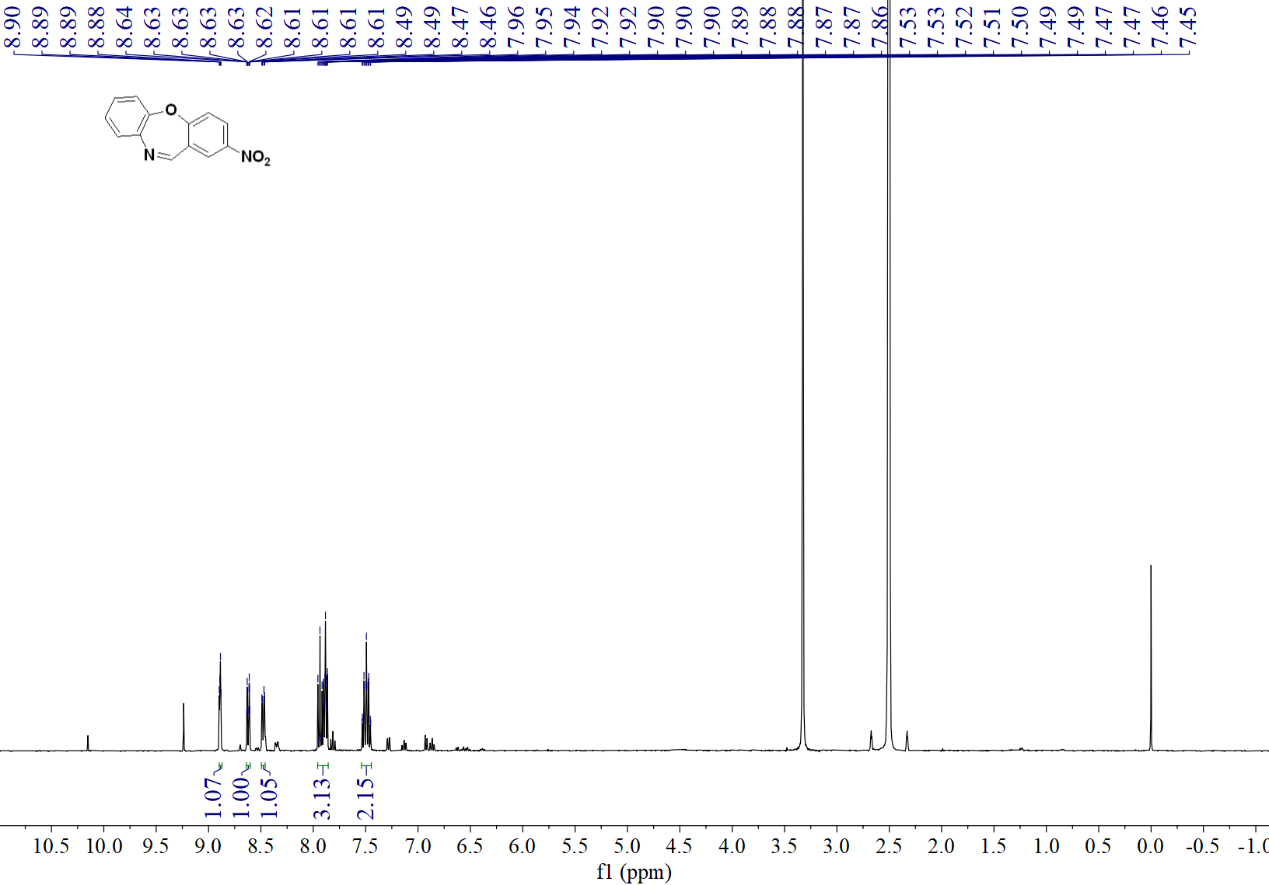


^1^H spectrum of compound **JX3214b** (DMSO-*d_6_*)


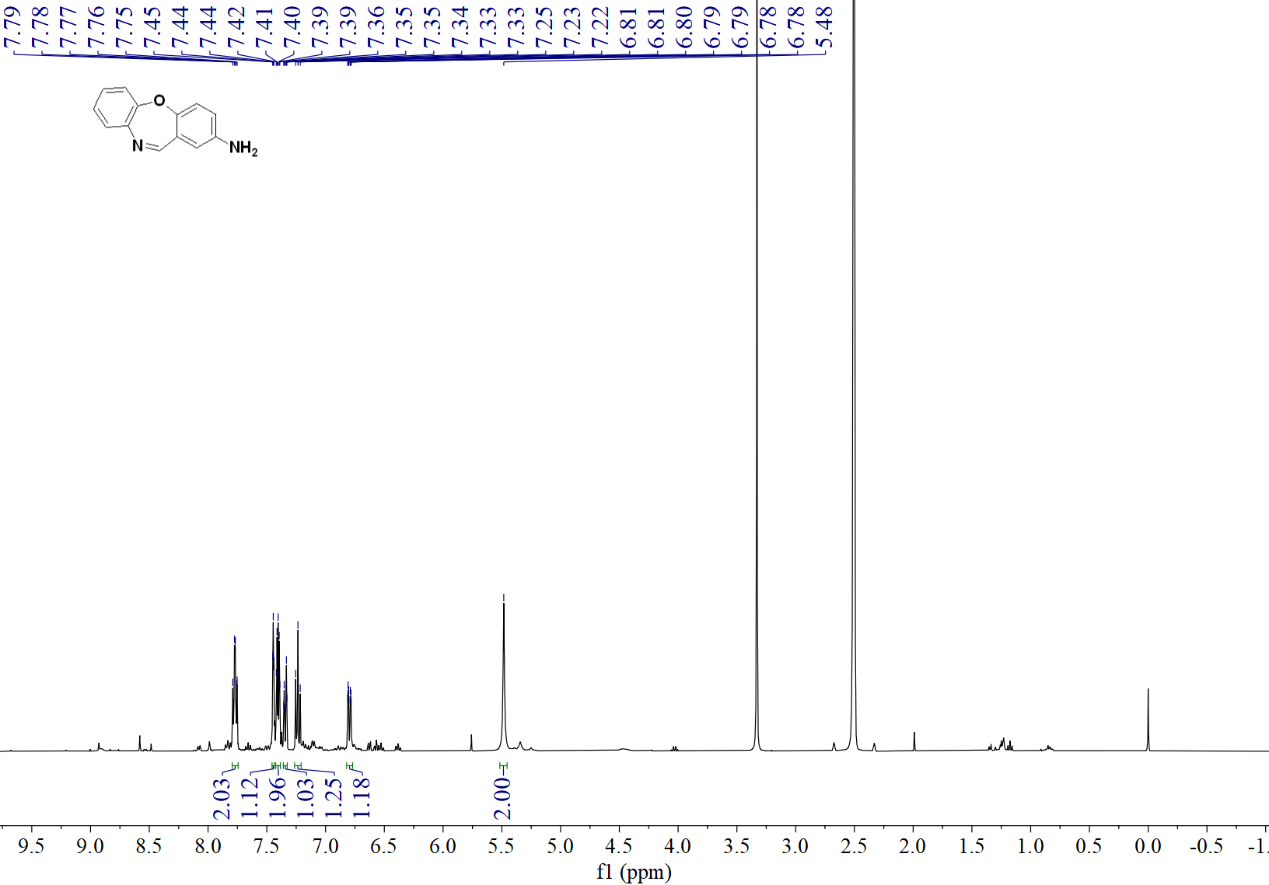


^1^H spectrum of compound **JX3214c** (DMSO-*d_6_*)


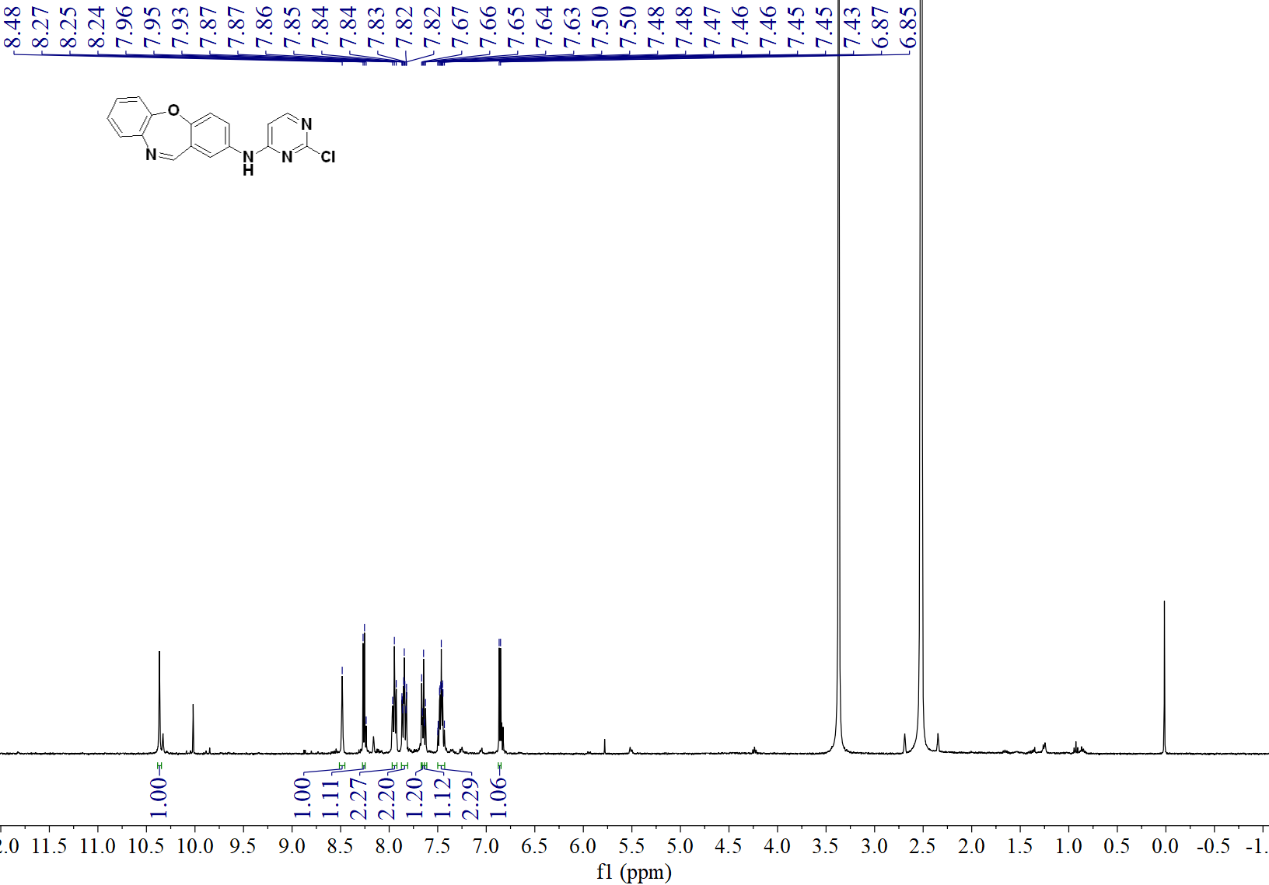


^1^H spectrum of compound **JX3214** (DMSO-*d_6_*)


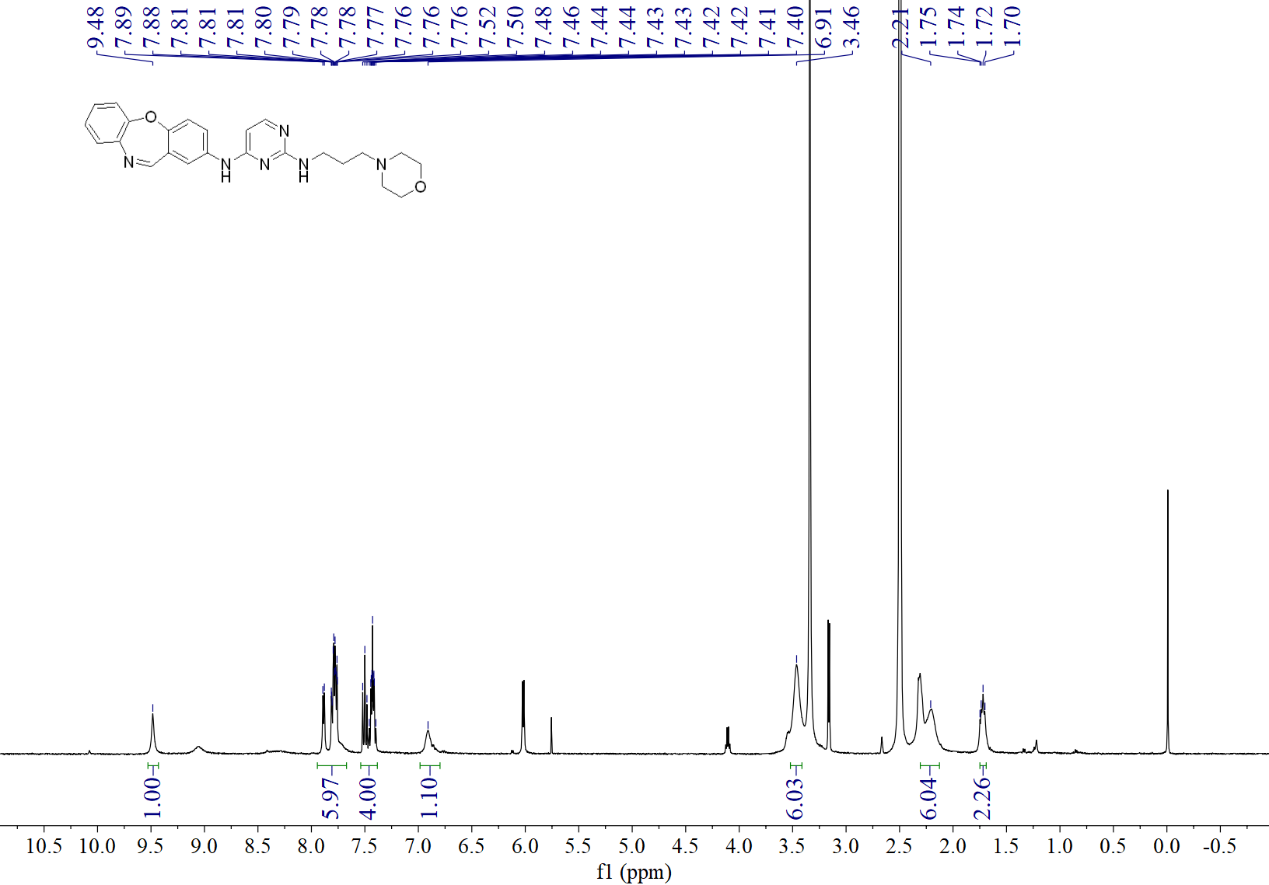


^1^H spectrum of compound **JX3215** (DMSO-*d_6_*)


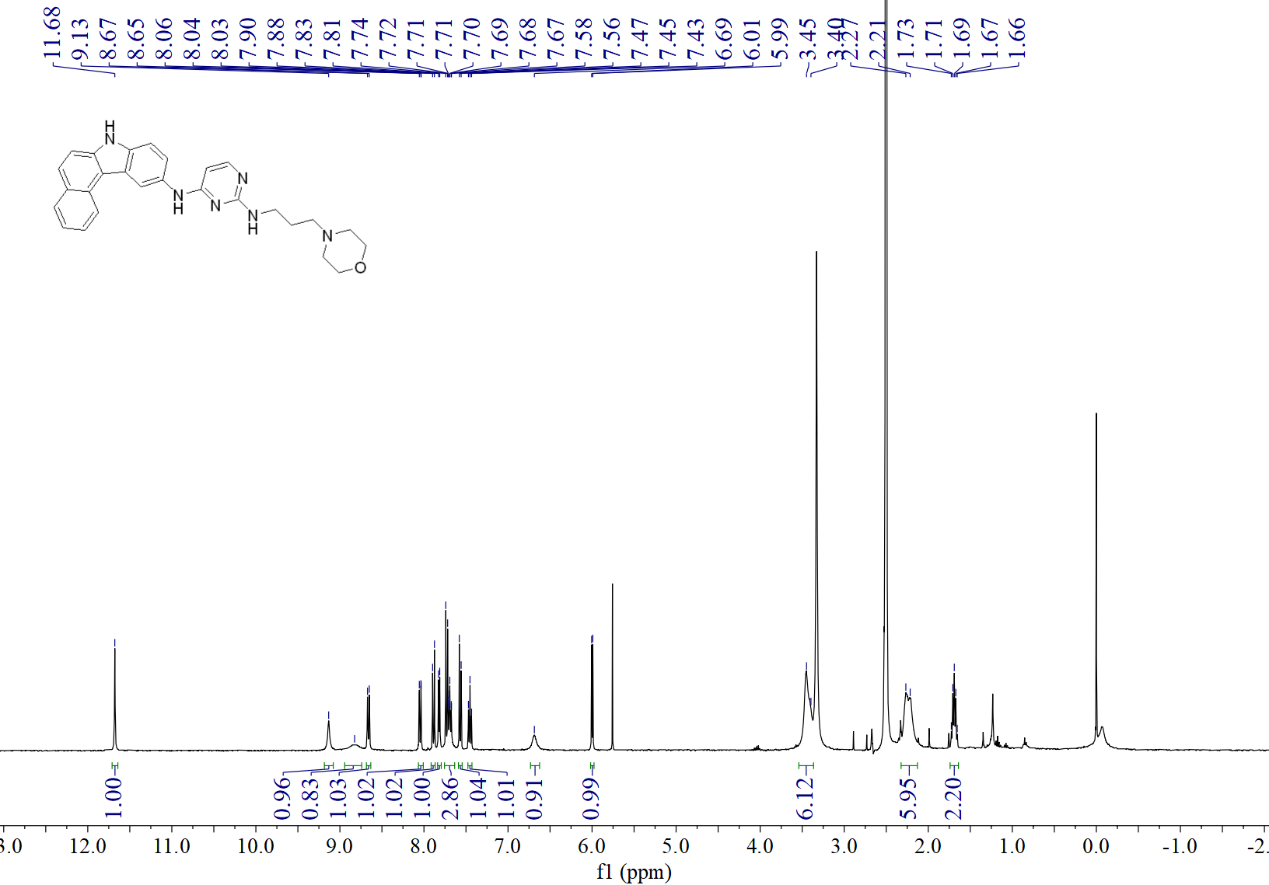


^1^H spectrum of compound **JX3216a** (DMSO-*d_6_*)


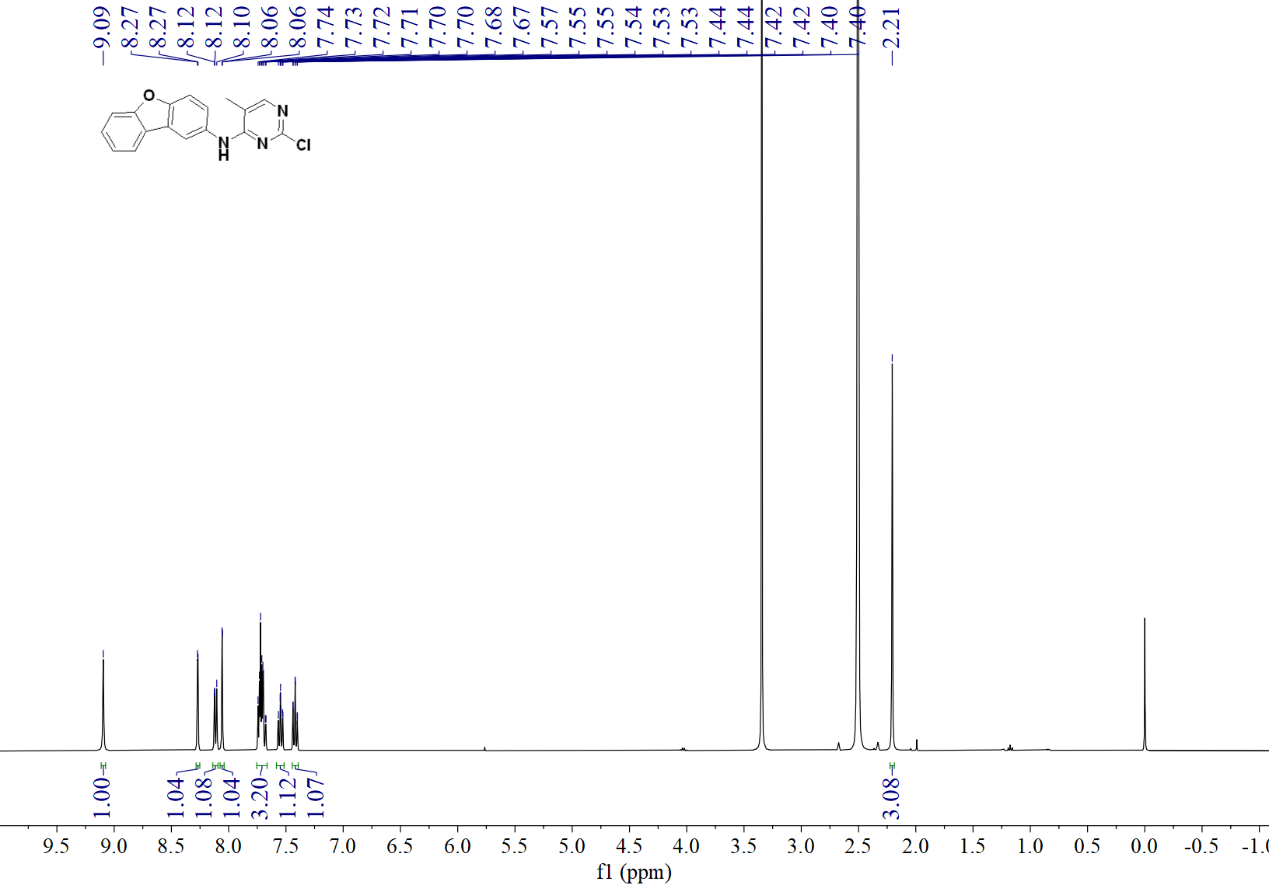


^1^H spectrum of compound **JX3216** (DMSO-*d_6_*)


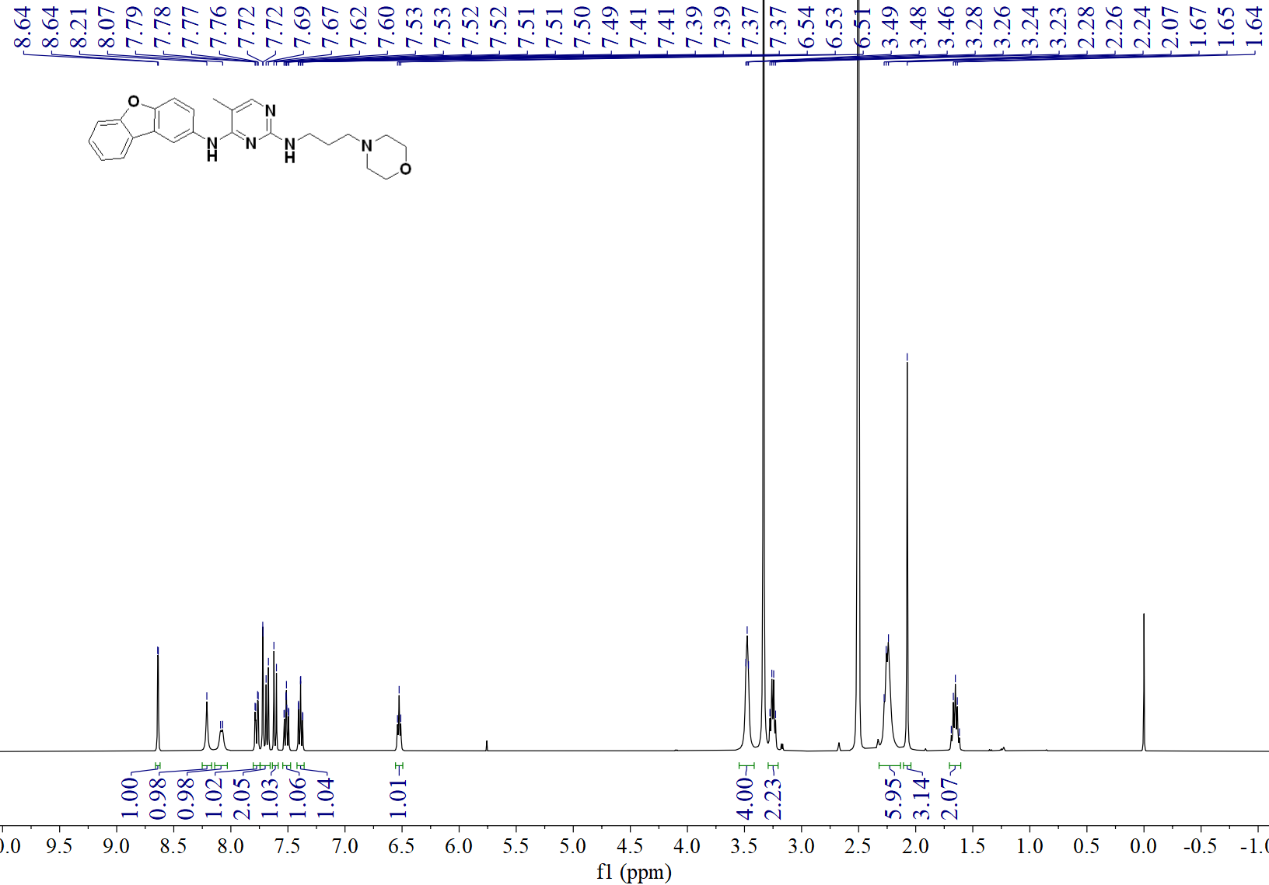


^1^H spectrum of compound **JX3217a** (DMSO-*d_6_*)


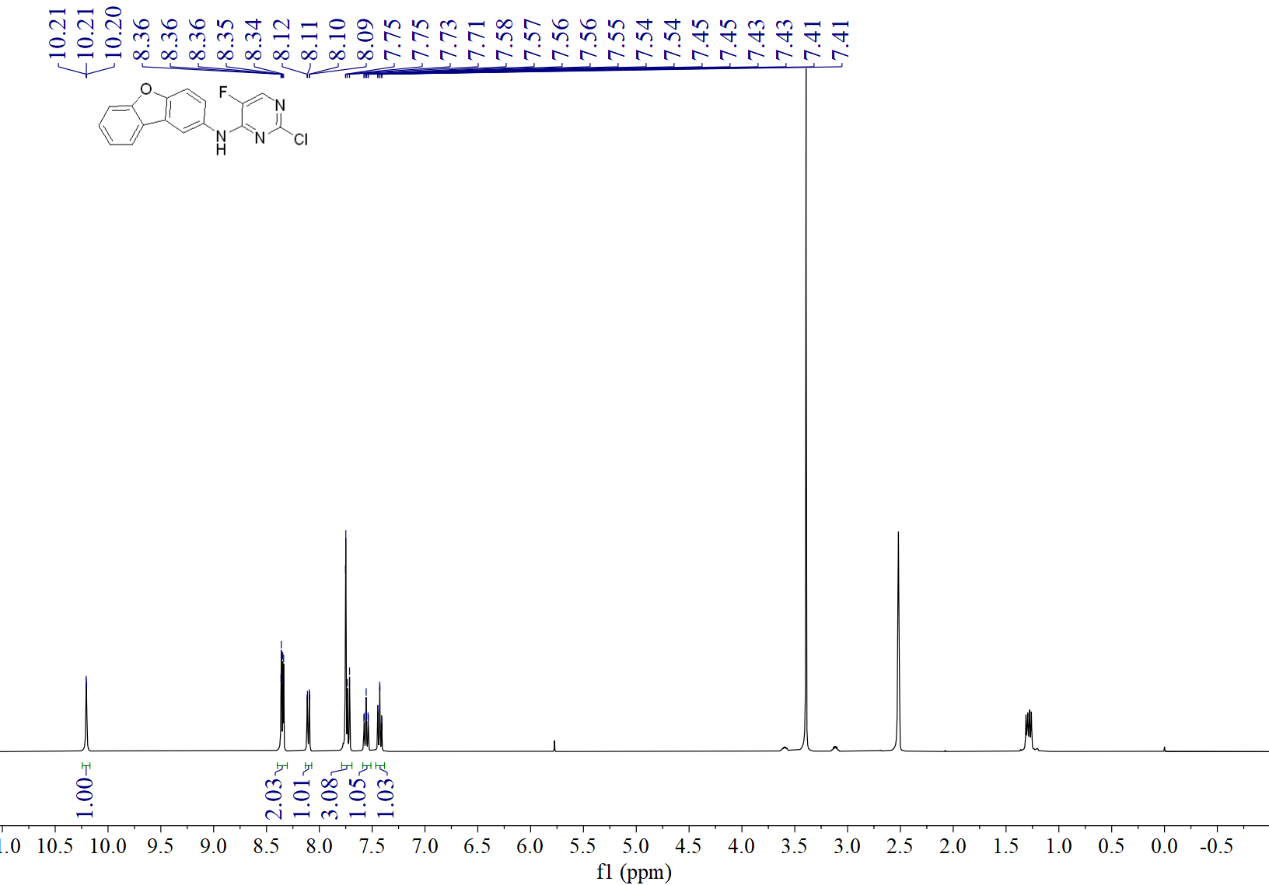


^1^H spectrum of compound **JX3217** (DMSO-*d_6_*)


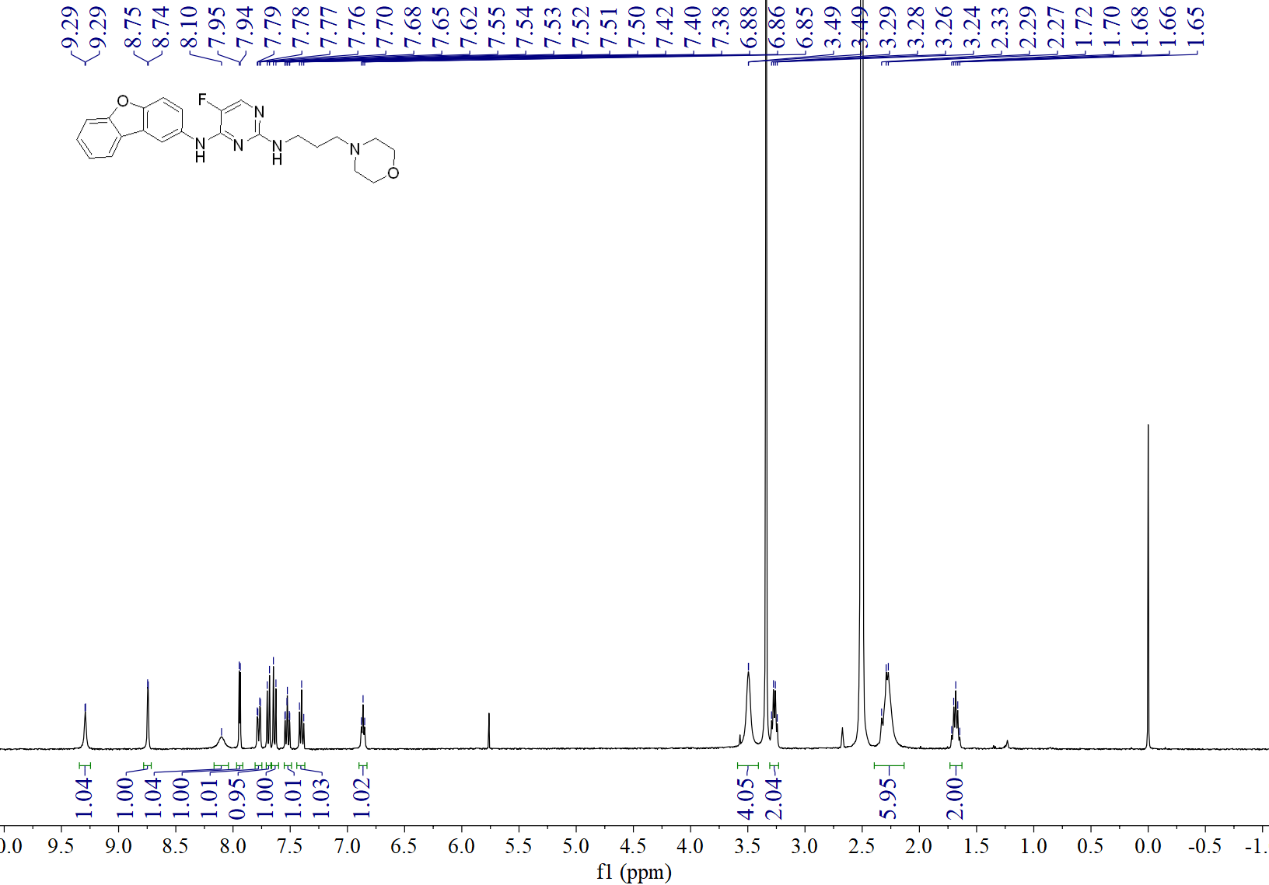


^1^H spectrum of compound **JX3218a** (DMSO-*d_6_*)


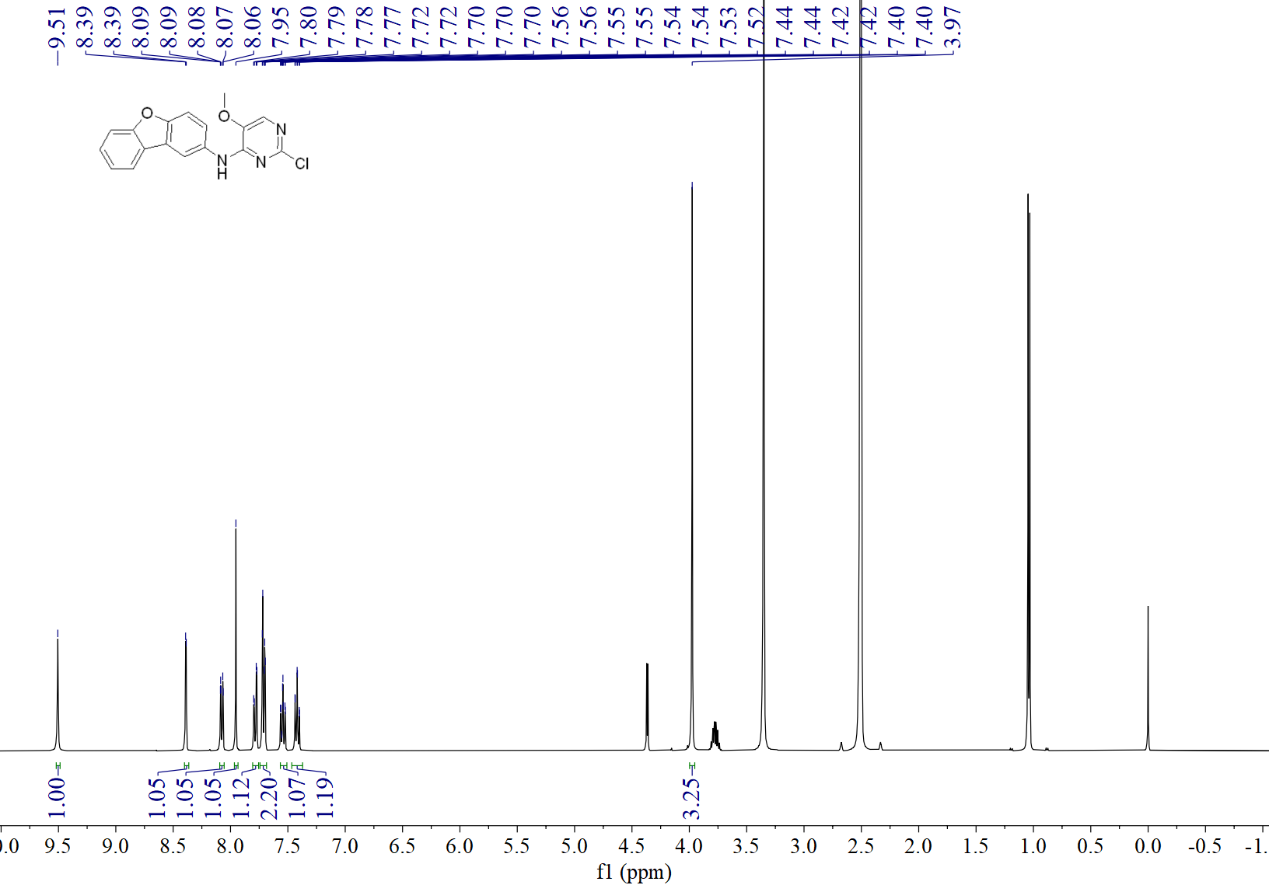


^1^H spectrum of compound **JX3218** (DMSO-*d_6_*)


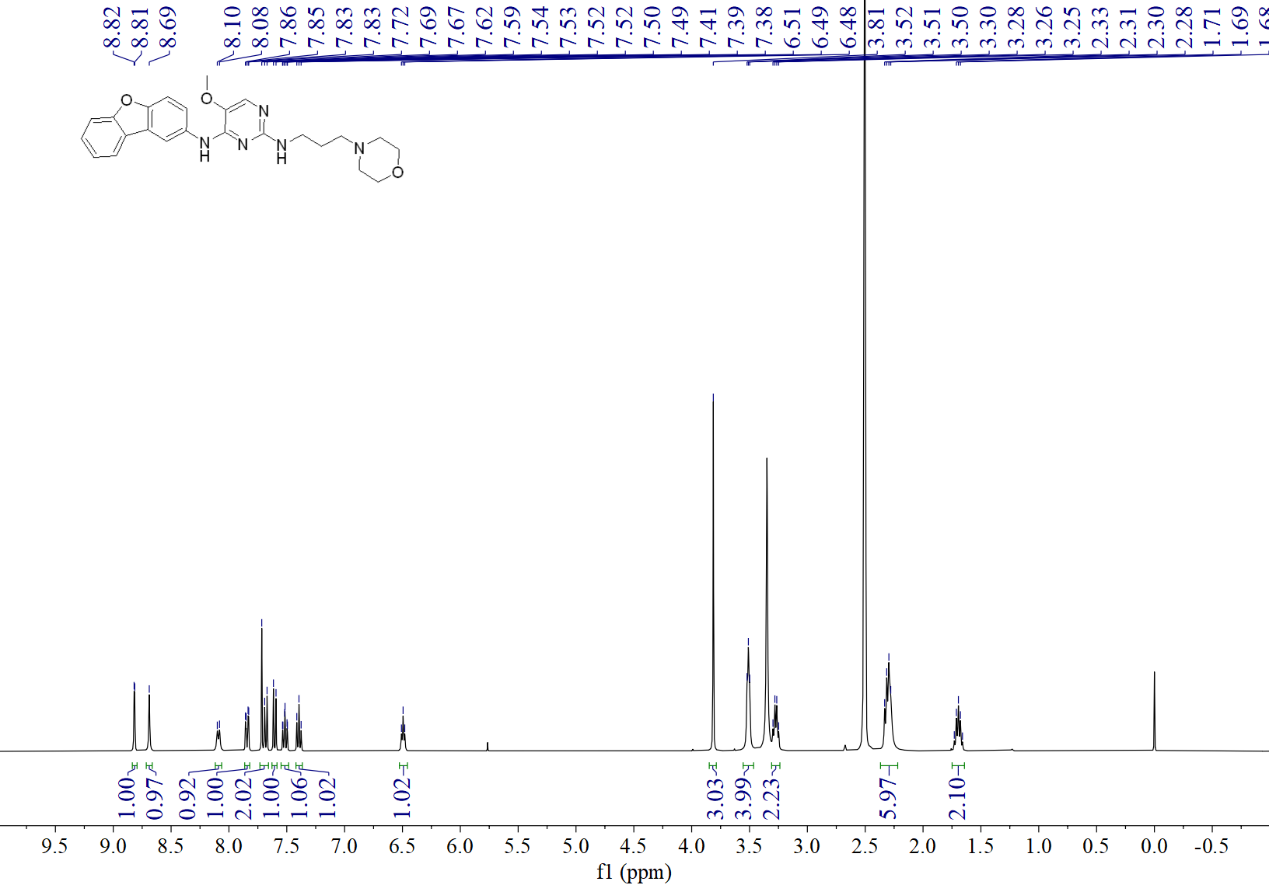


^1^H spectrum of compound **JX3219a** (DMSO-*d_6_*)


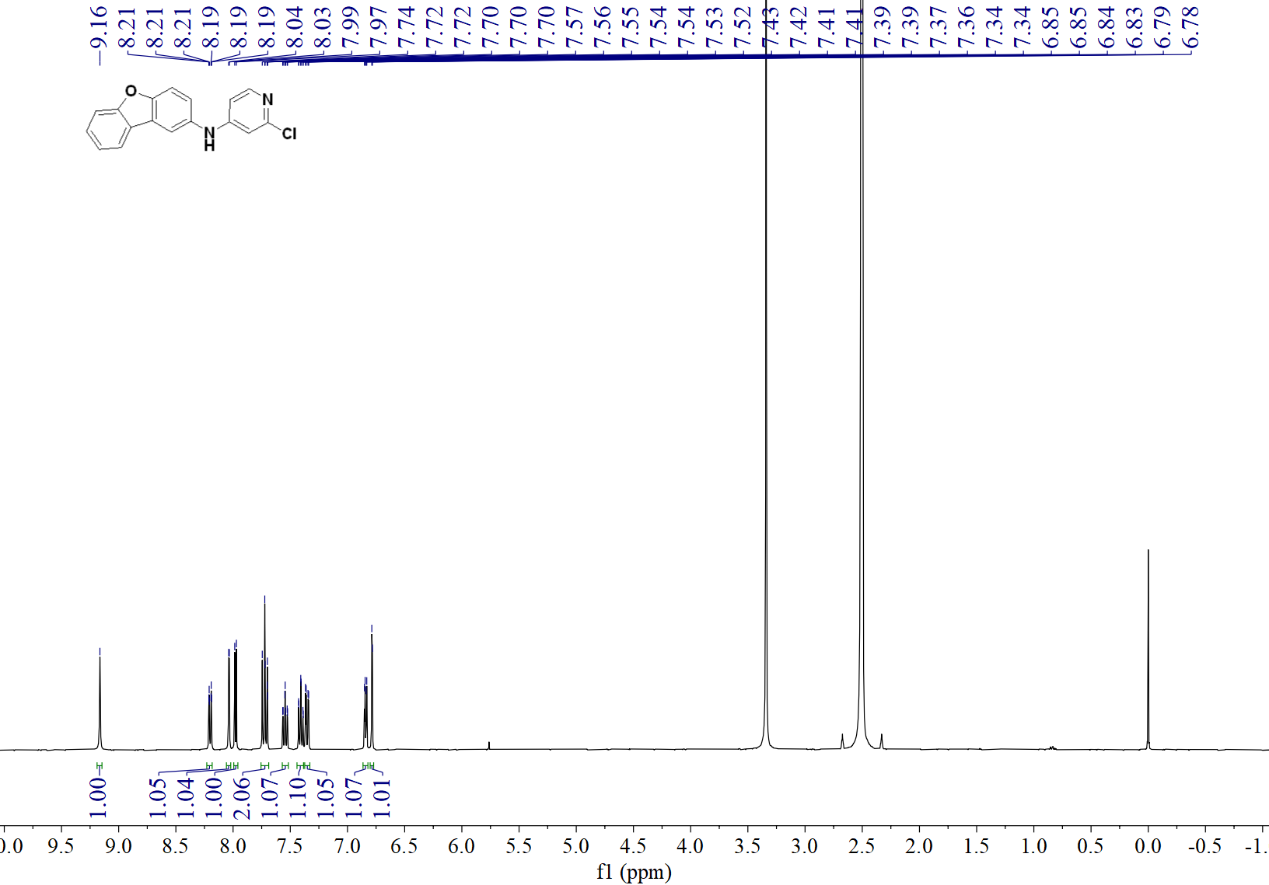


^1^H spectrum of compound **JX3219** (DMSO-*d_6_*)


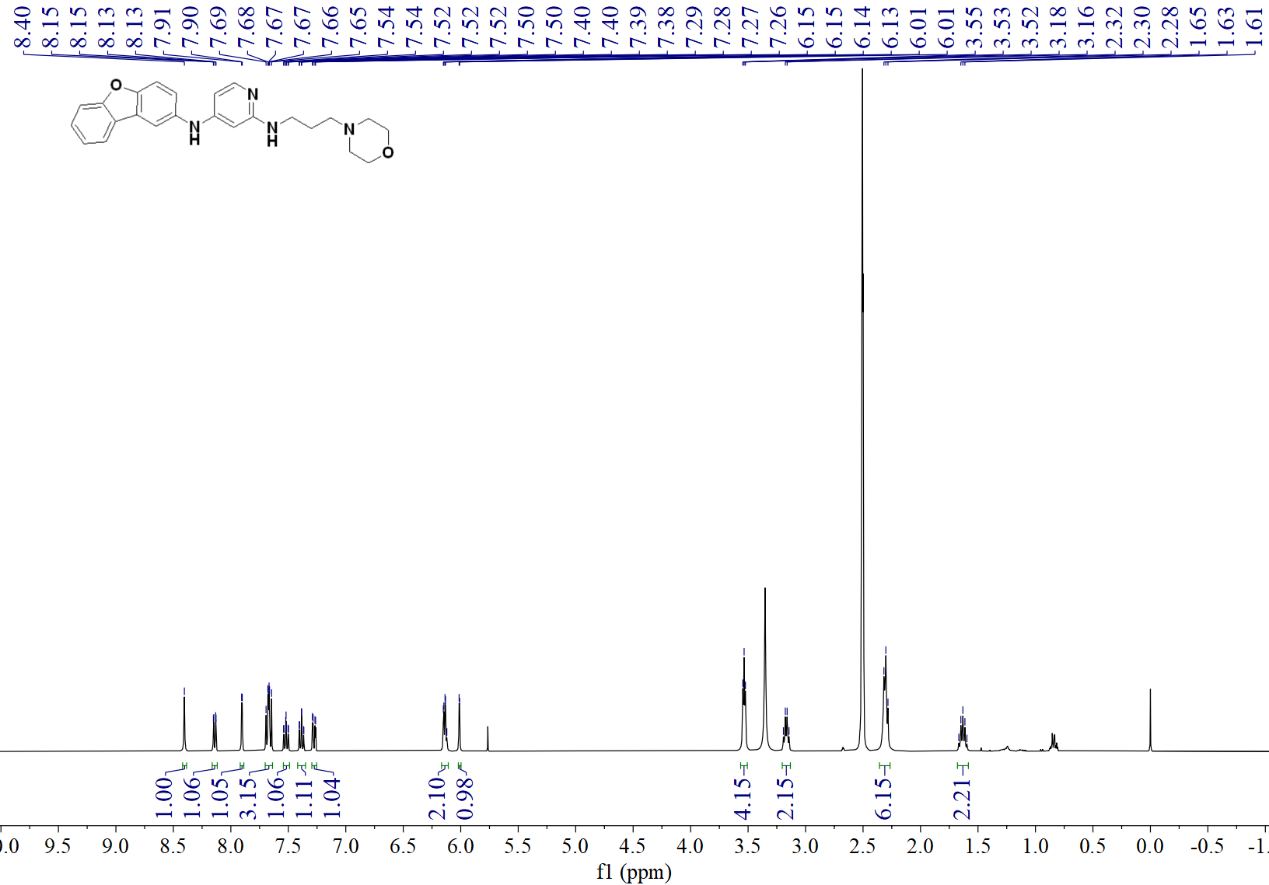


^1^H spectrum of compound **JX3220a** (DMSO-*d_6_*)


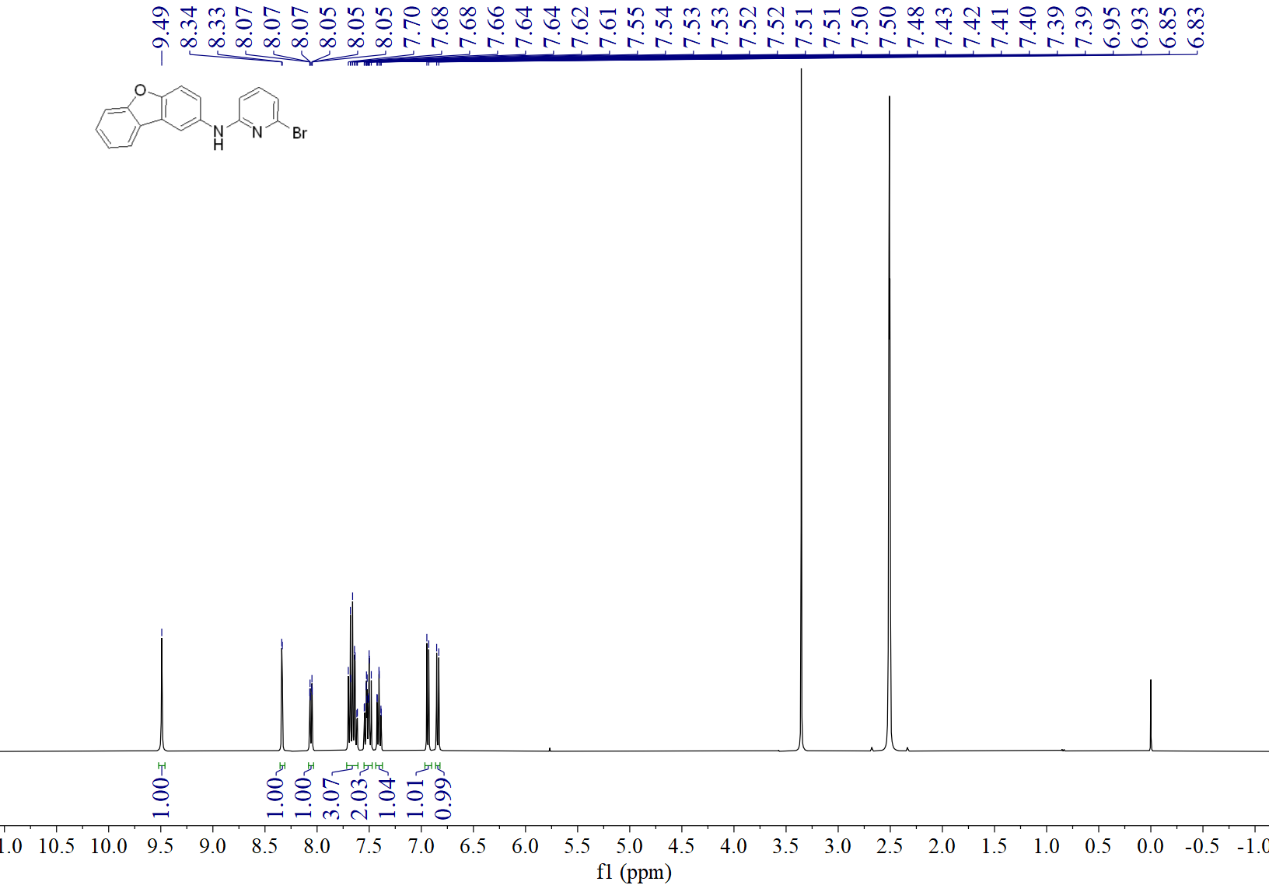


^1^H spectrum of compound **JX3220** (DMSO-*d_6_*)


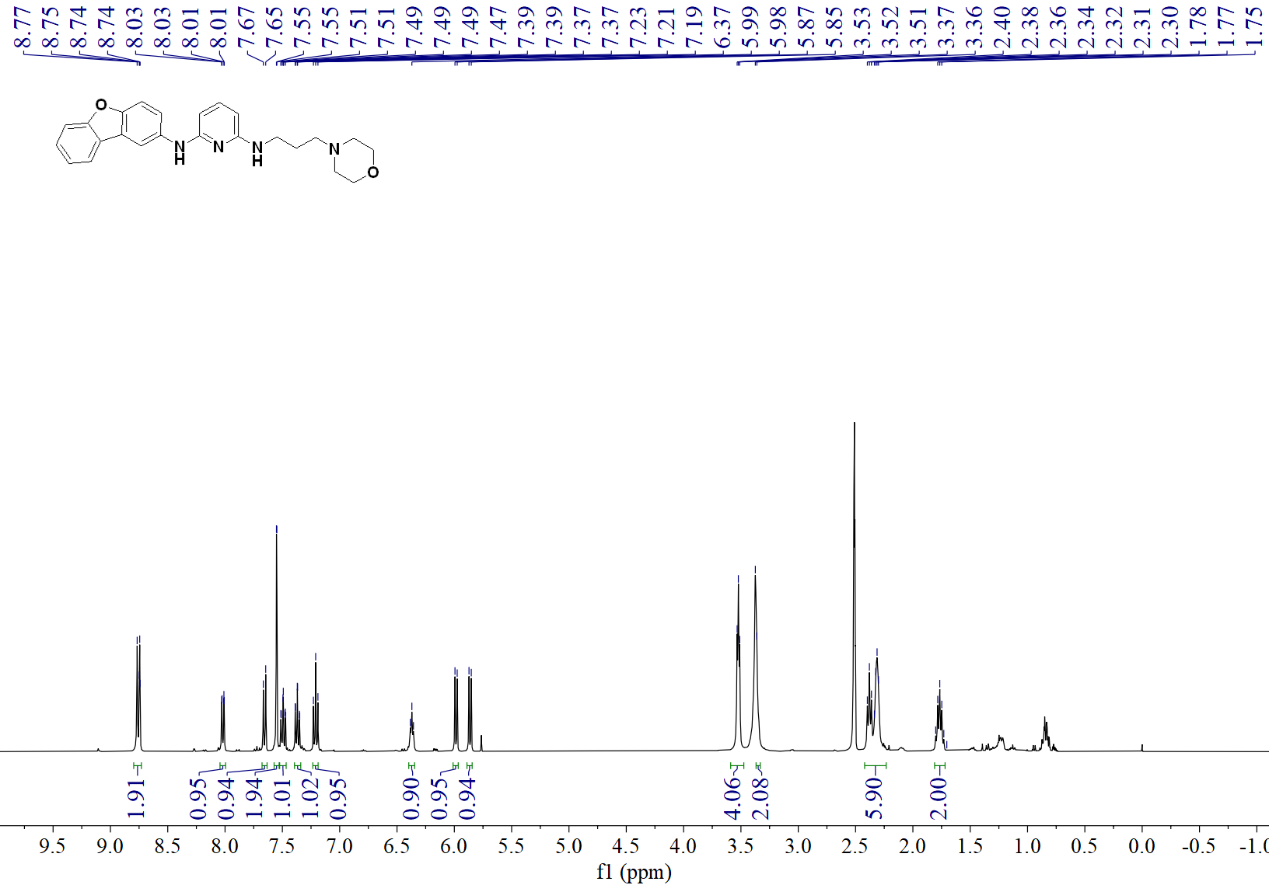


^1^H spectrum of compound **JX3221a** (DMSO-*d_6_*)


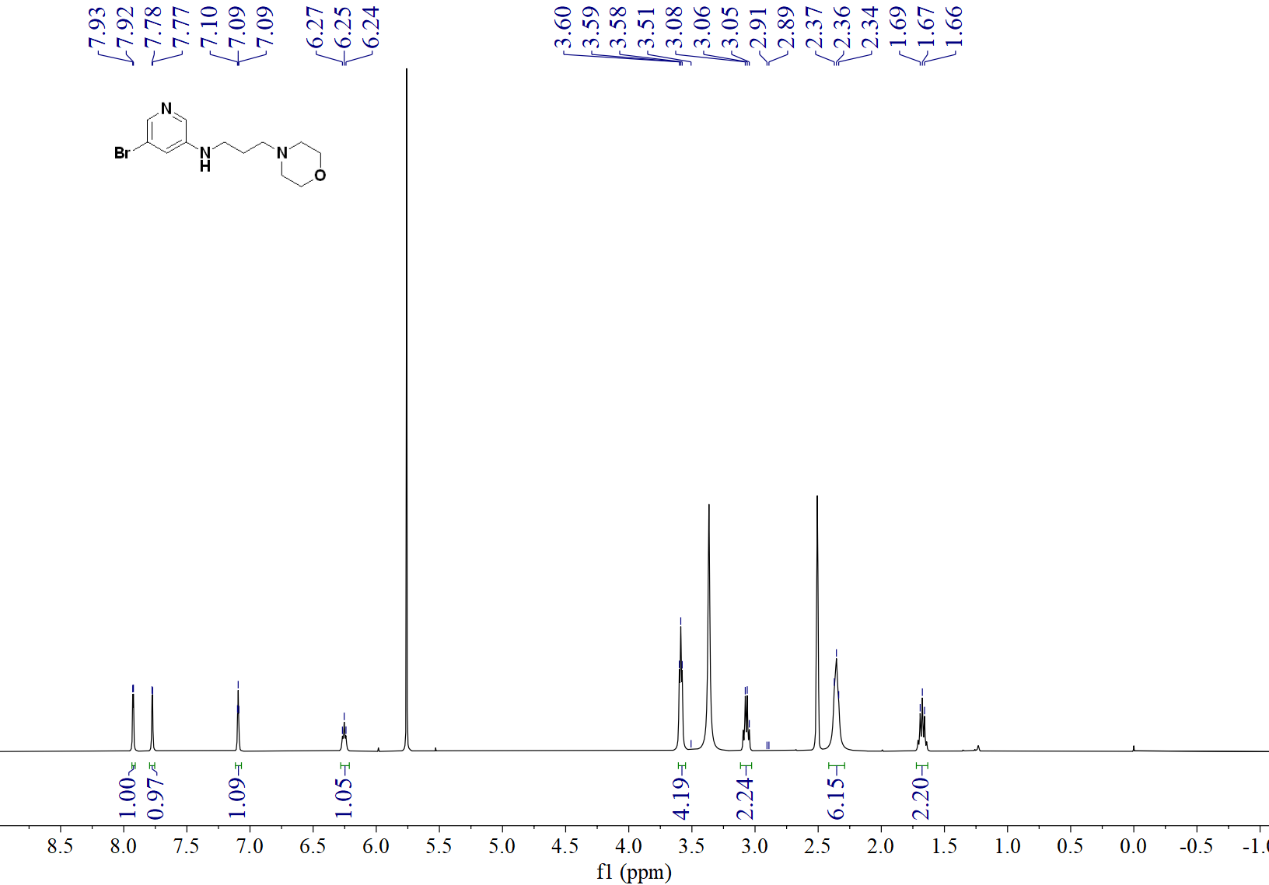


^1^H spectrum of compound **JX3221** (DMSO-*d_6_*)


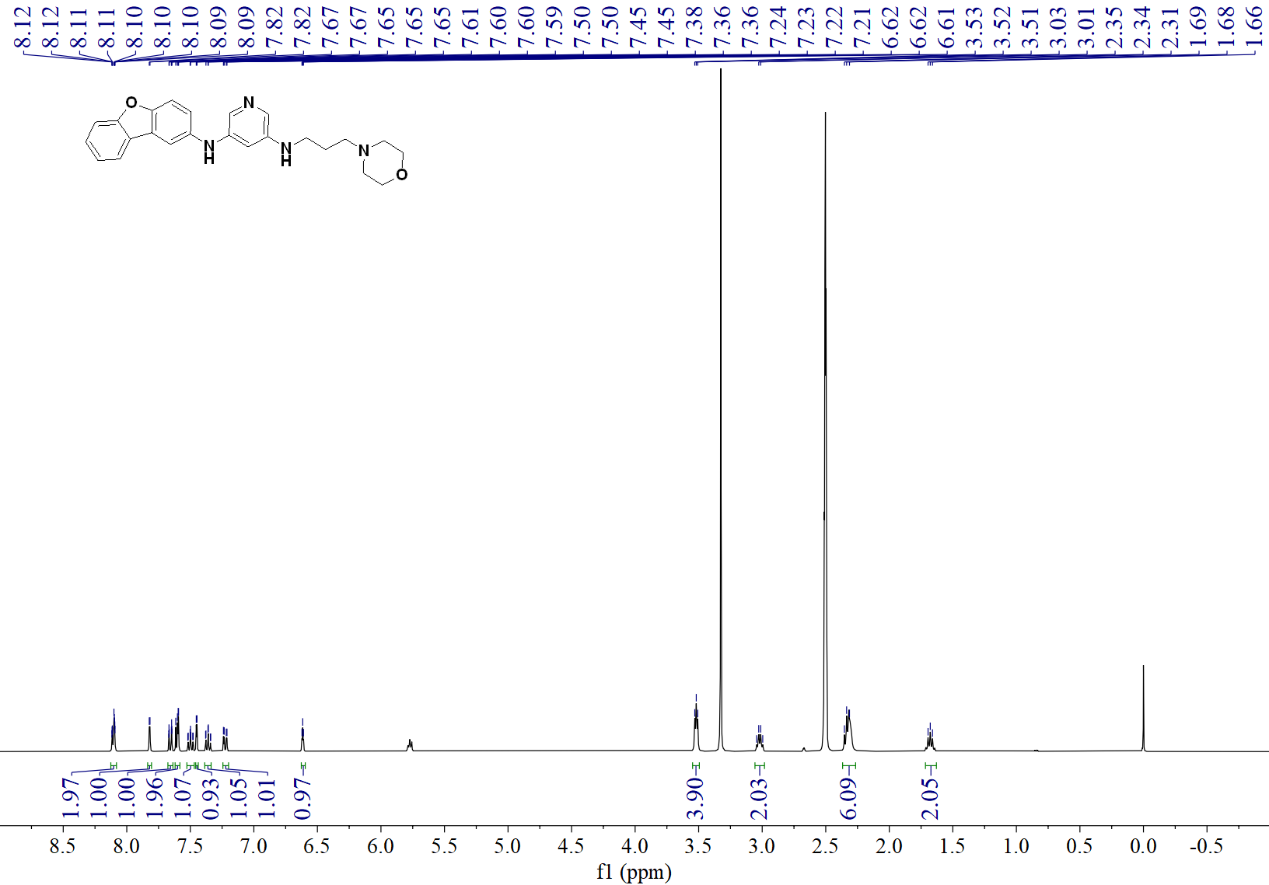


^1^H spectrum of compound **JX3222a** (DMSO-*d_6_*)


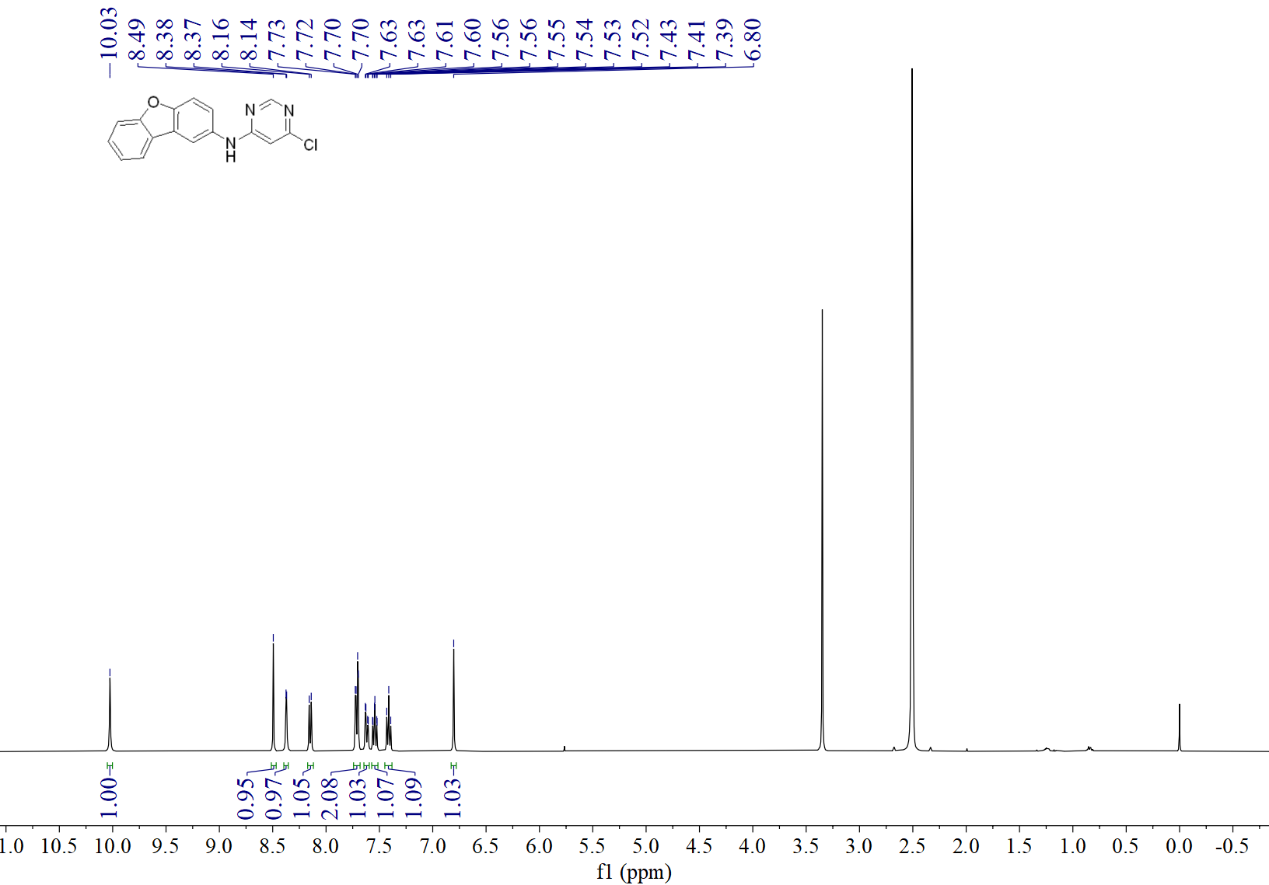


^1^H spectrum of compound **JX3222** (DMSO-*d_6_*)


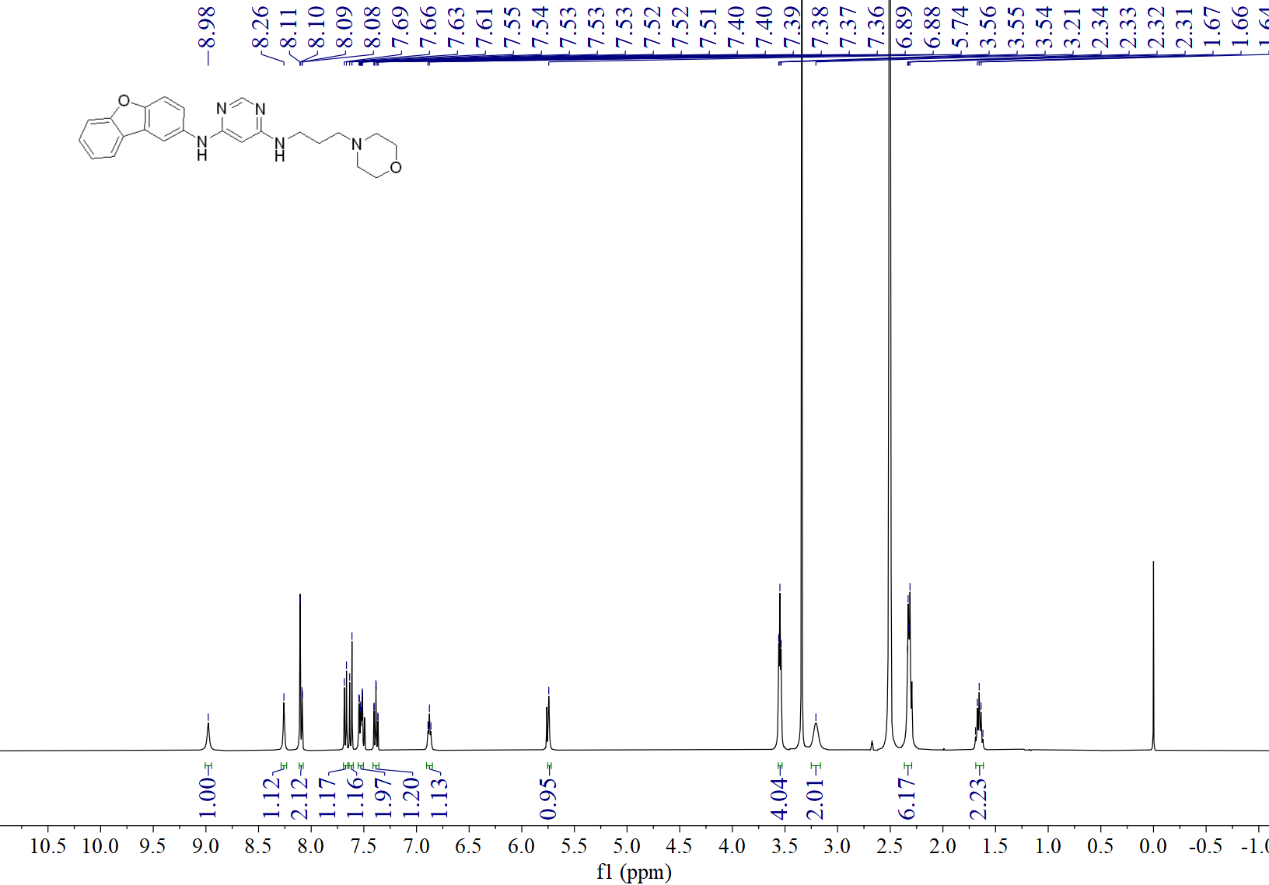


^1^H spectrum of compound **JX3223** (DMSO-*d_6_*)


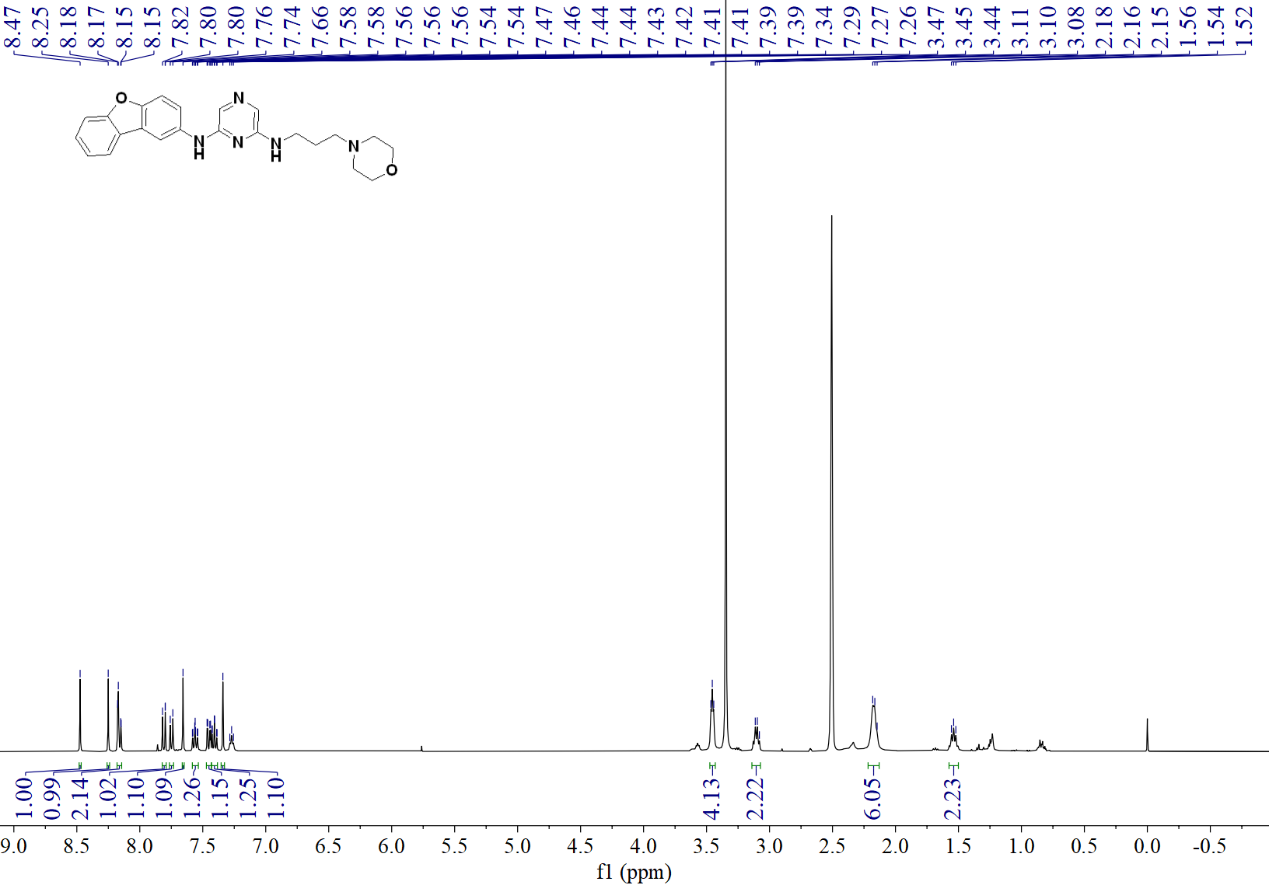


^1^H spectrum of compound **JX3224a** (DMSO-*d_6_*)


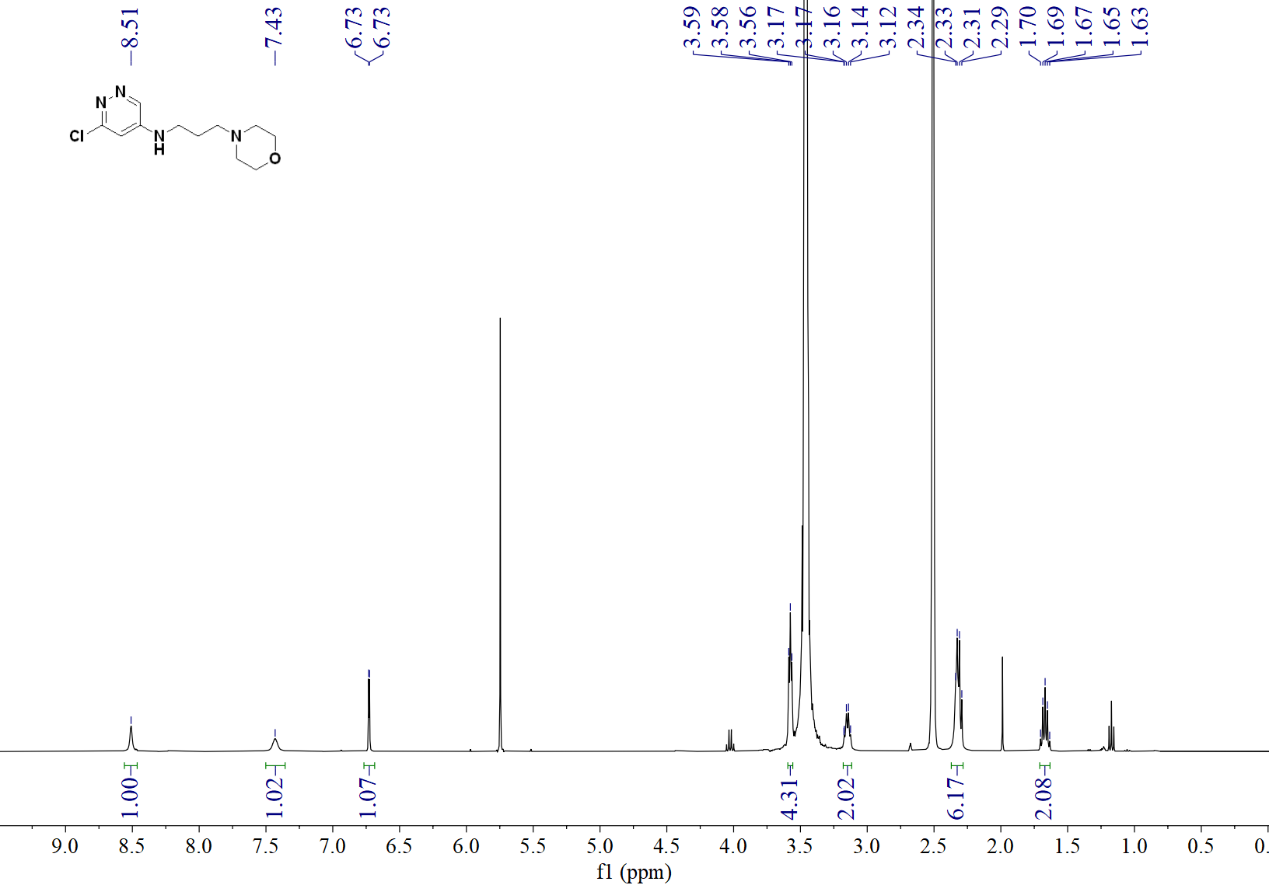


^1^H spectrum of compound **JX3224** (DMSO-*d_6_*)


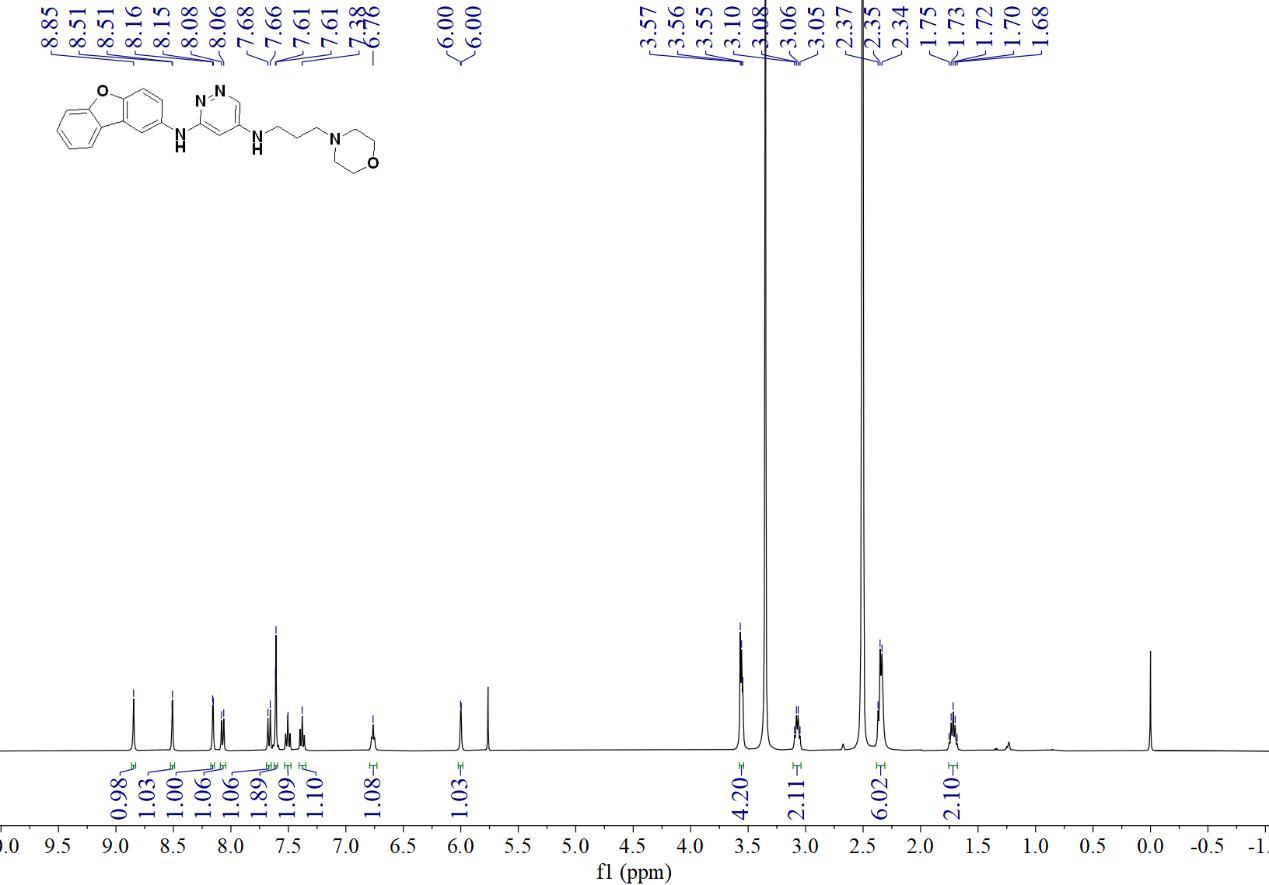


^1^H spectrum of compound **JX3225a** (DMSO-*d_6_*)


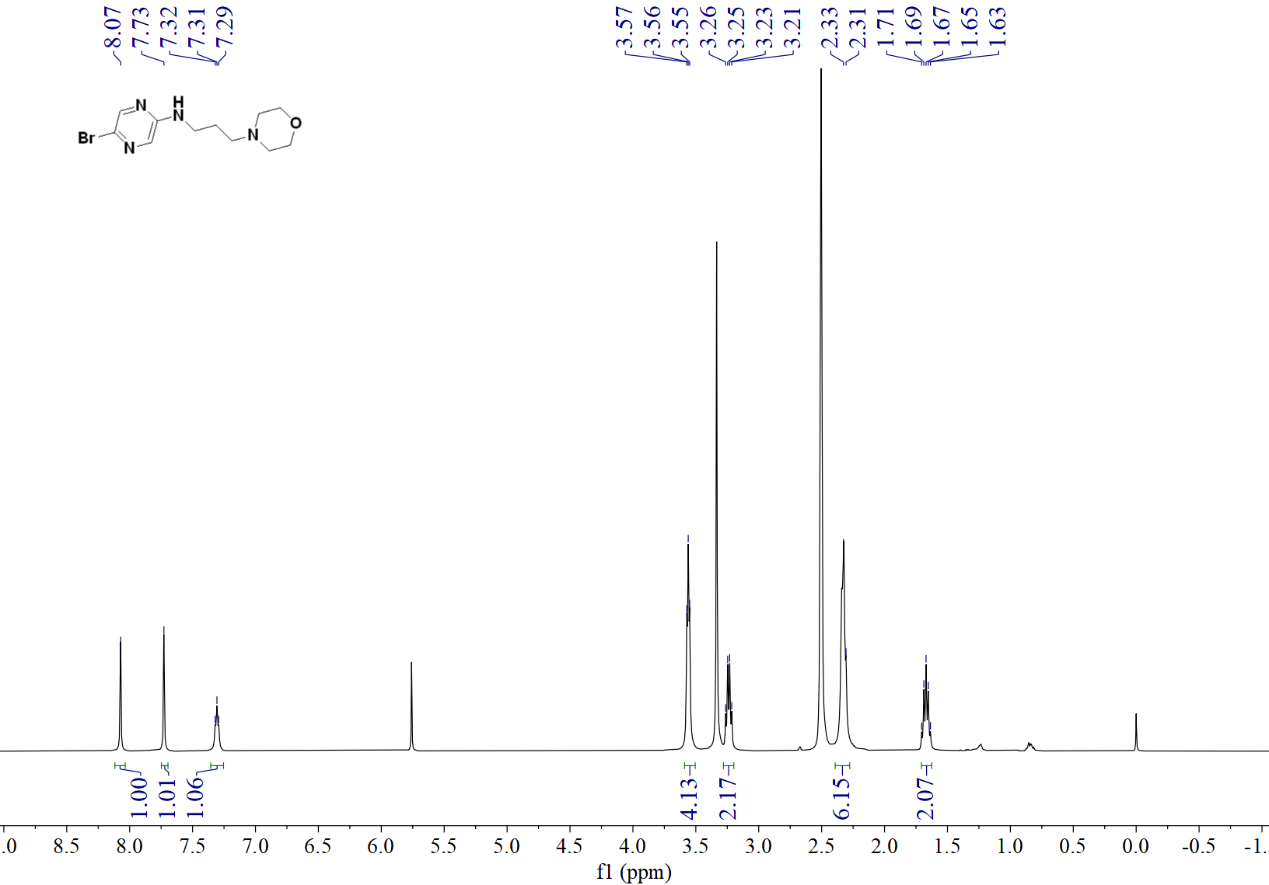


^1^H spectrum of compound **JX3225** (DMSO-*d_6_*)


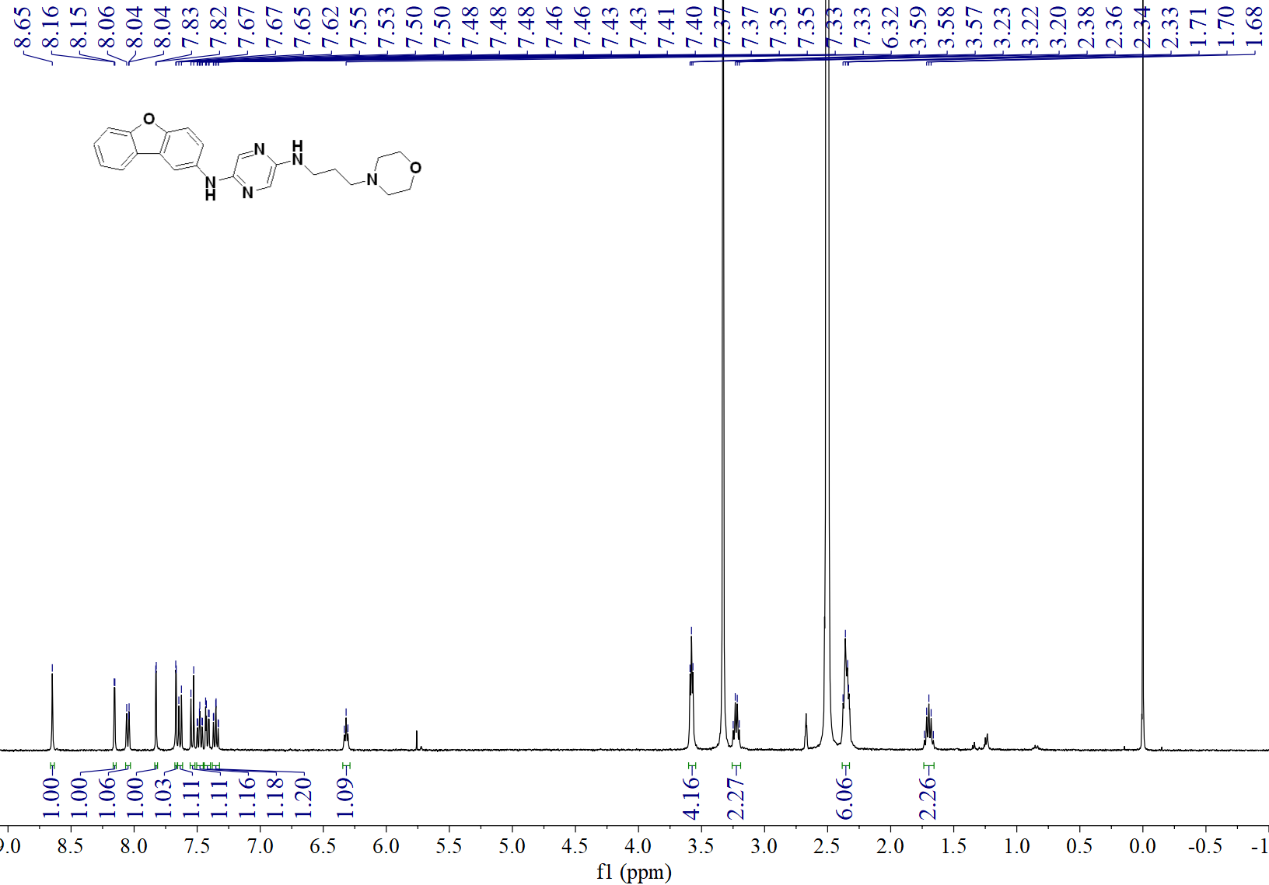


^1^H spectrum of compound **JX3226a** (DMSO-*d_6_*)


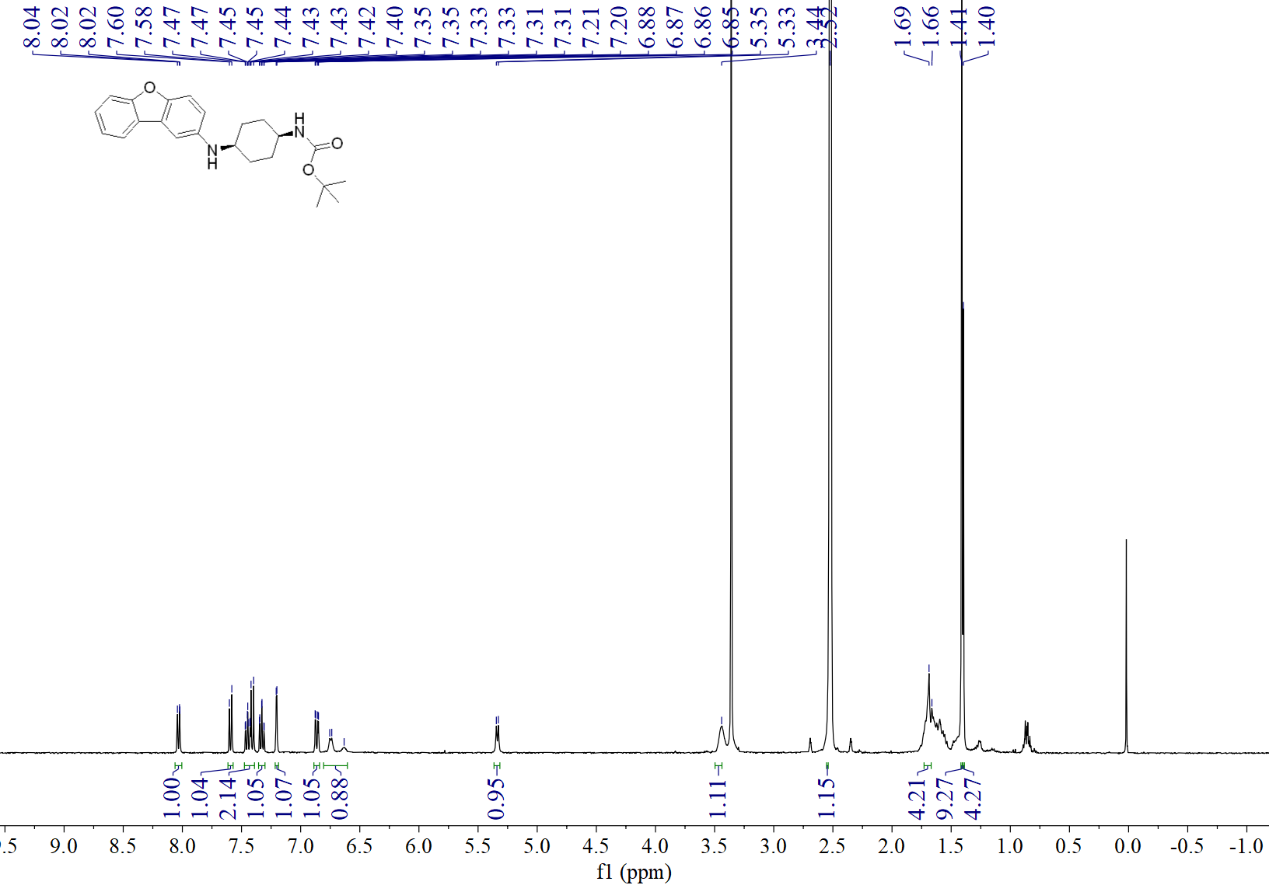


^1^H spectrum of compound **JX3226b** (DMSO-*d_6_*)


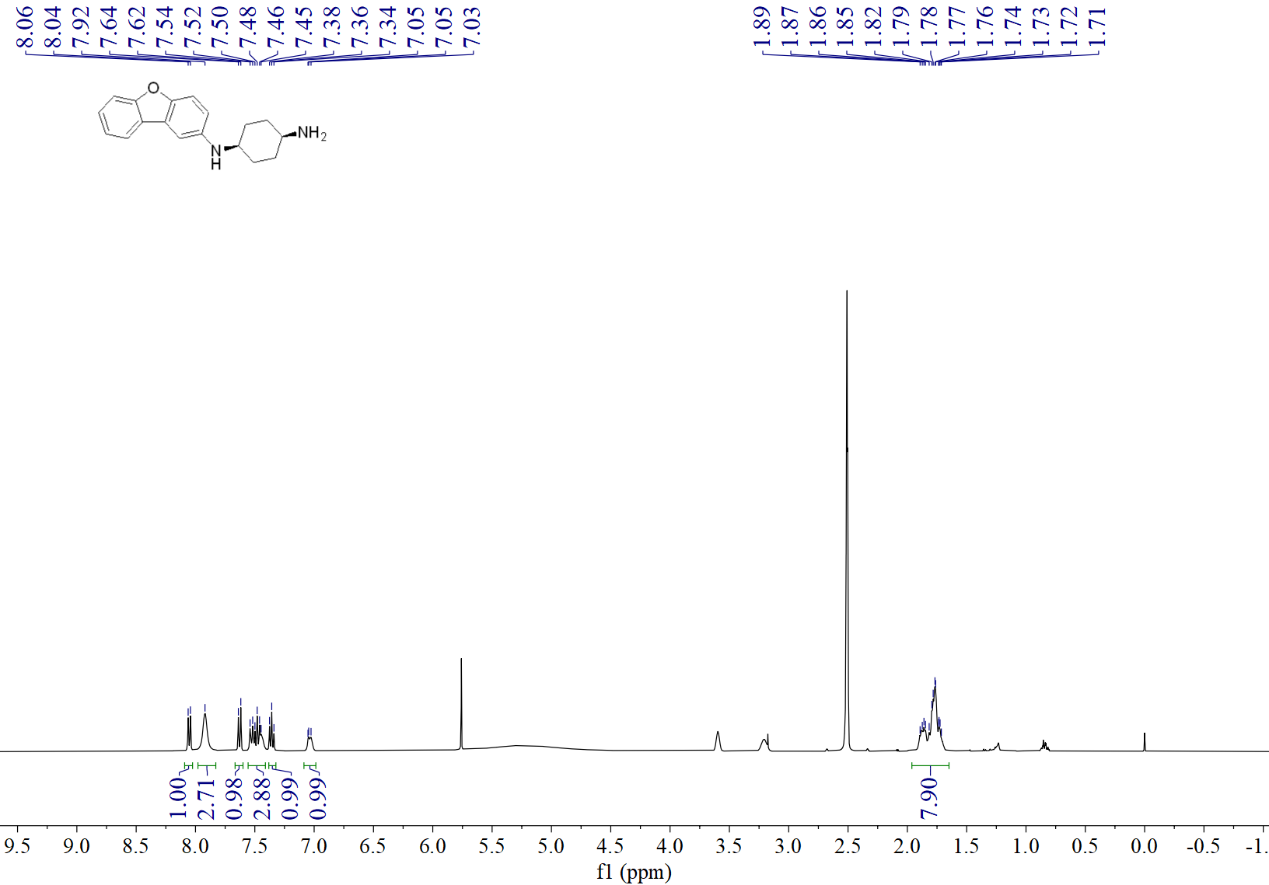


^1^H spectrum of compound **JX3226** (DMSO-*d_6_*)


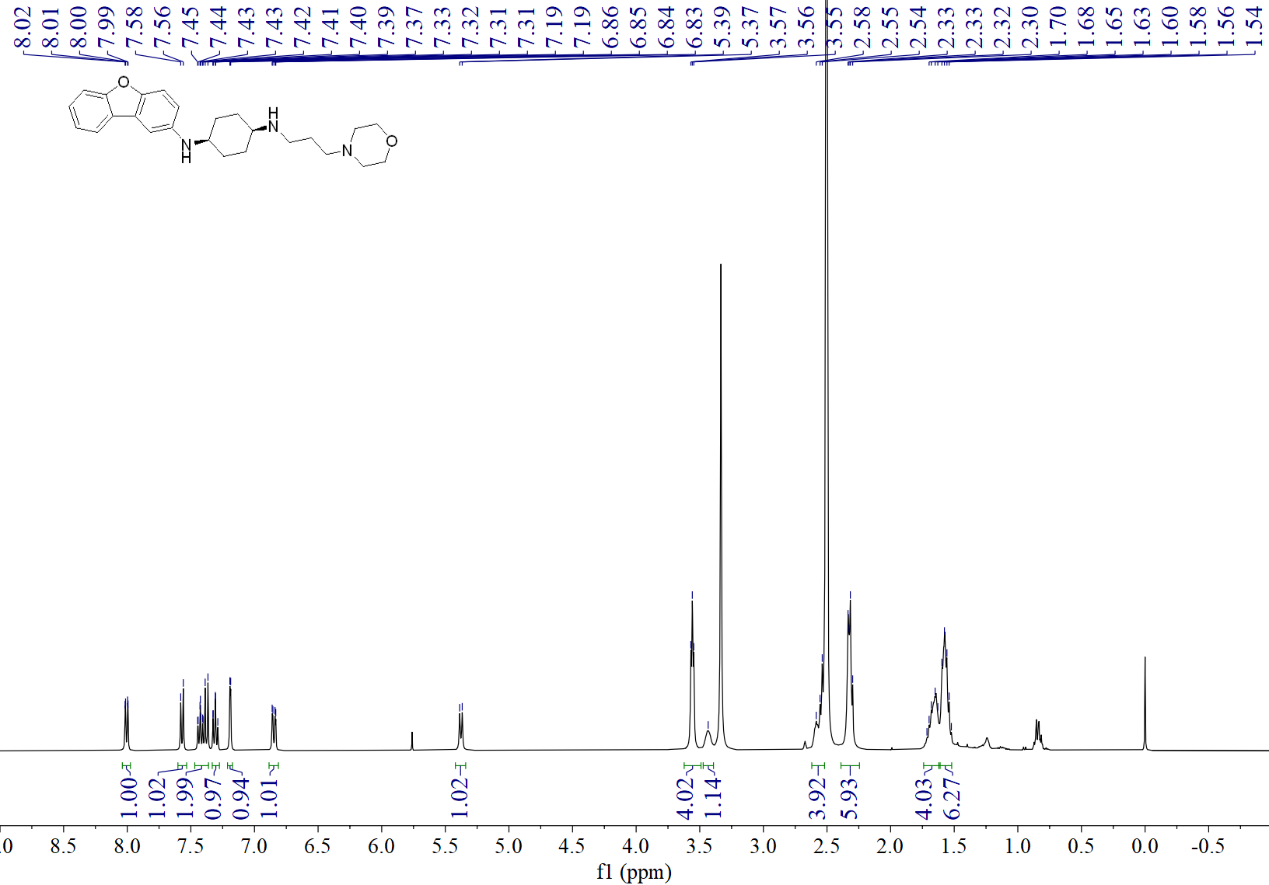


^1^H spectrum of compound **JX3227a** (DMSO-*d_6_*)


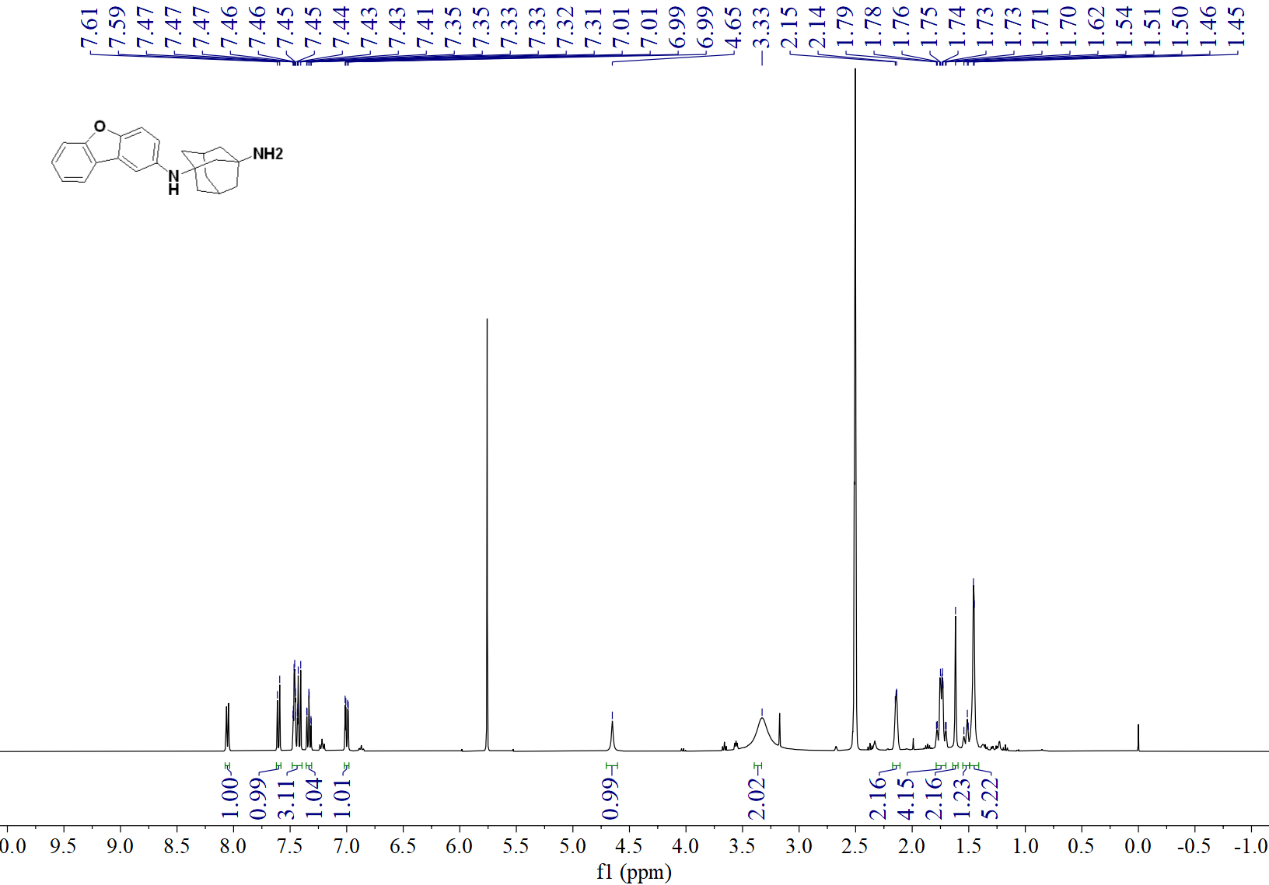


^1^H spectrum of compound **JX3227** (DMSO-*d_6_*)


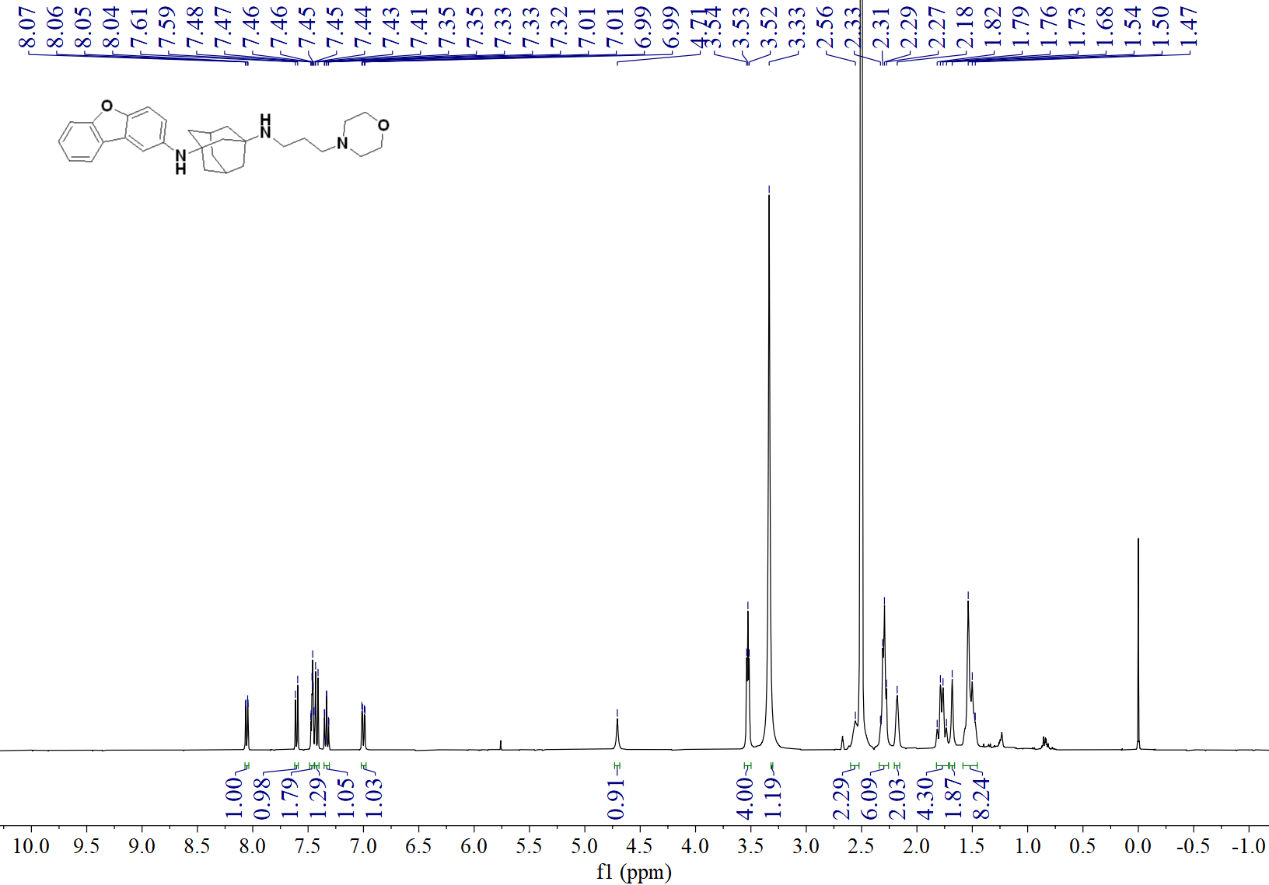


^1^H spectrum of compound **JX3228a** (DMSO-*d_6_*)


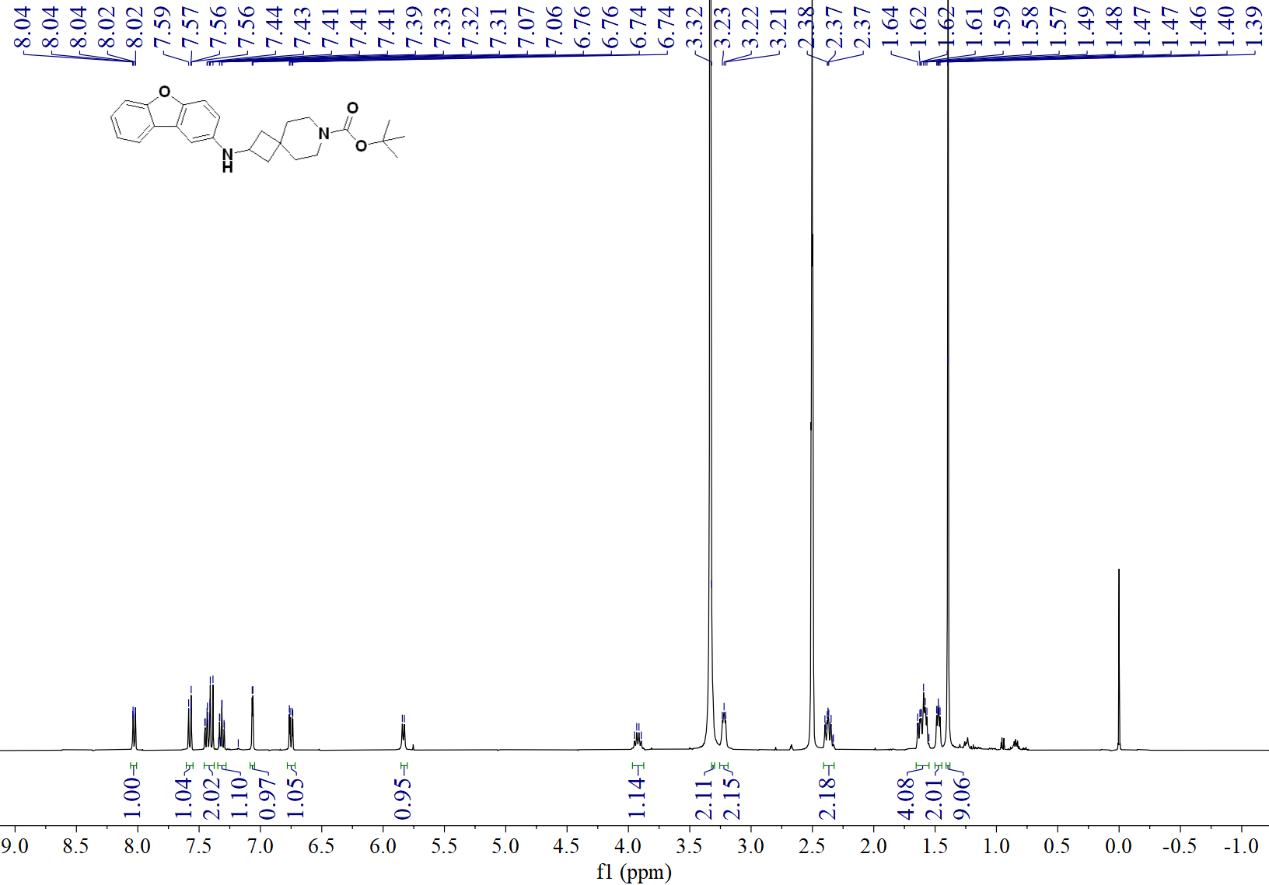


^1^H spectrum of compound **JX3228** (DMSO-*d_6_*)


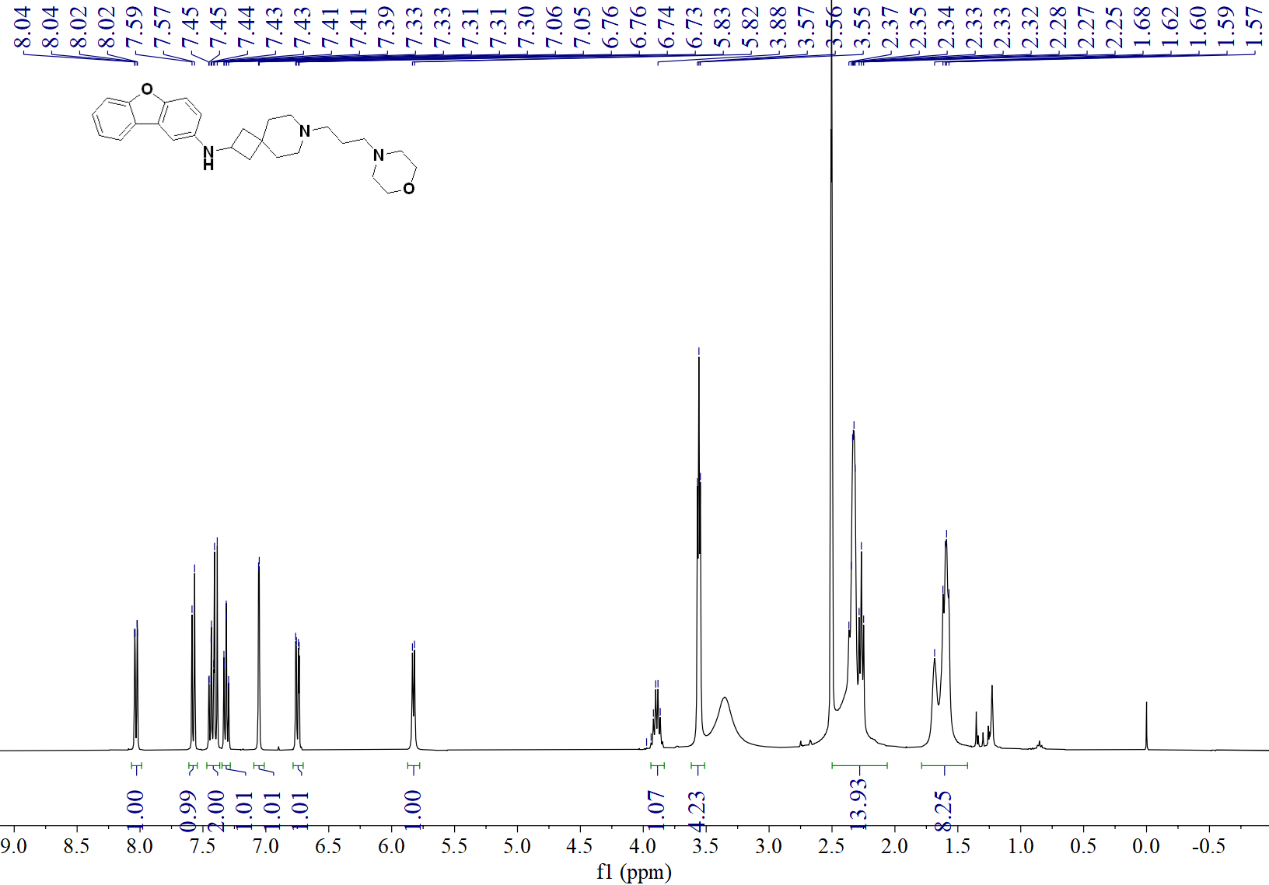


^1^H spectrum of compound **JX3229a** (DMSO-*d_6_*)


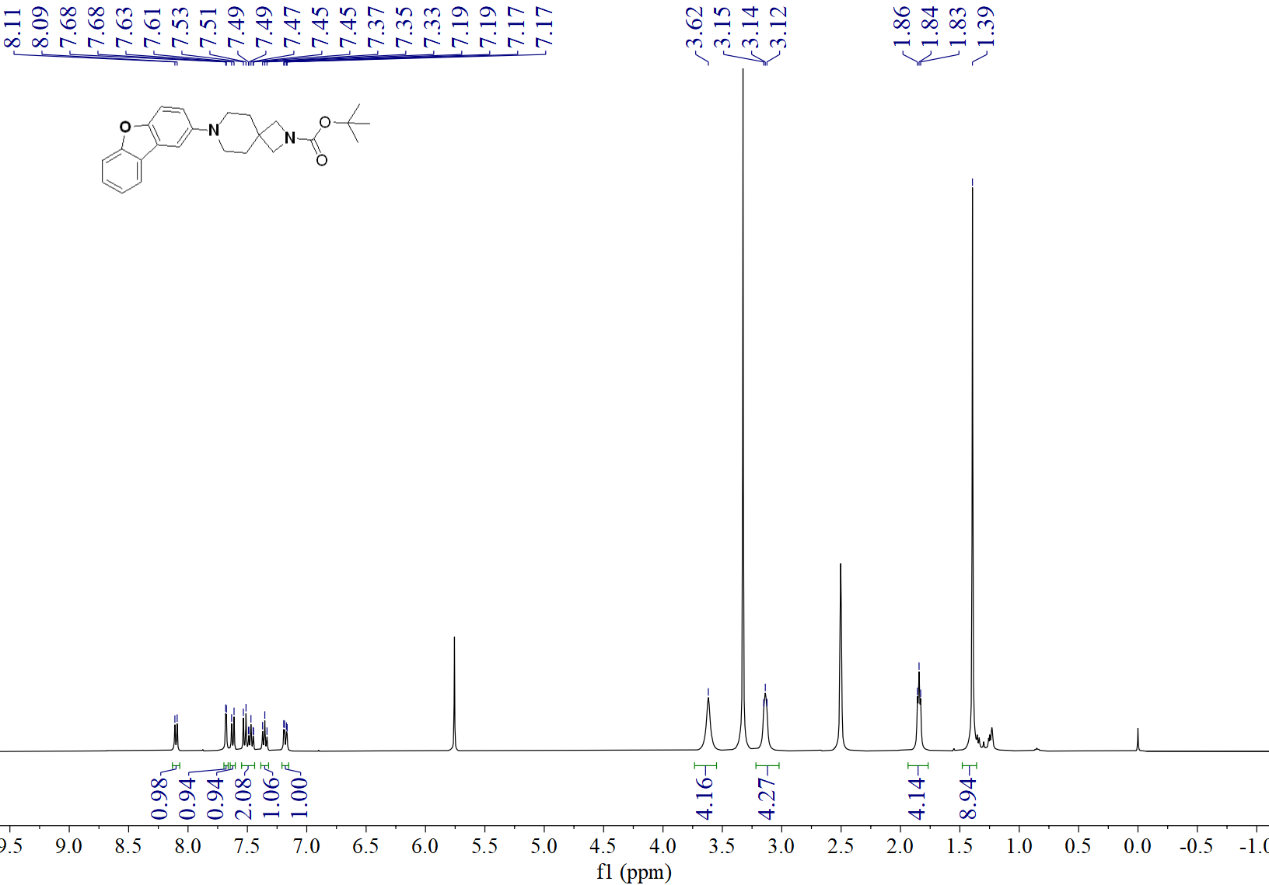


^1^H spectrum of compound **JX3229** (DMSO-*d_6_*)


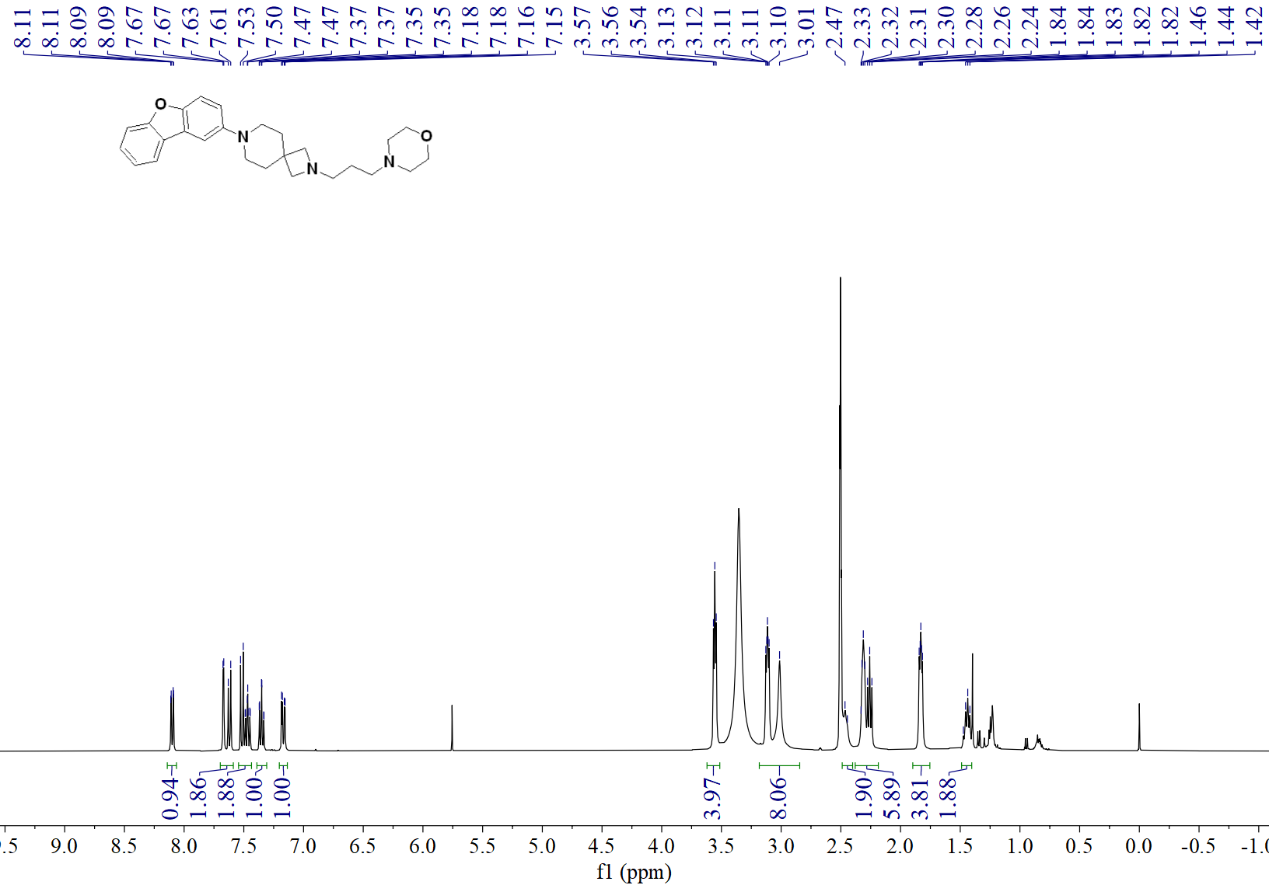


^1^H spectrum of compound **JX3230** (DMSO-*d_6_*)


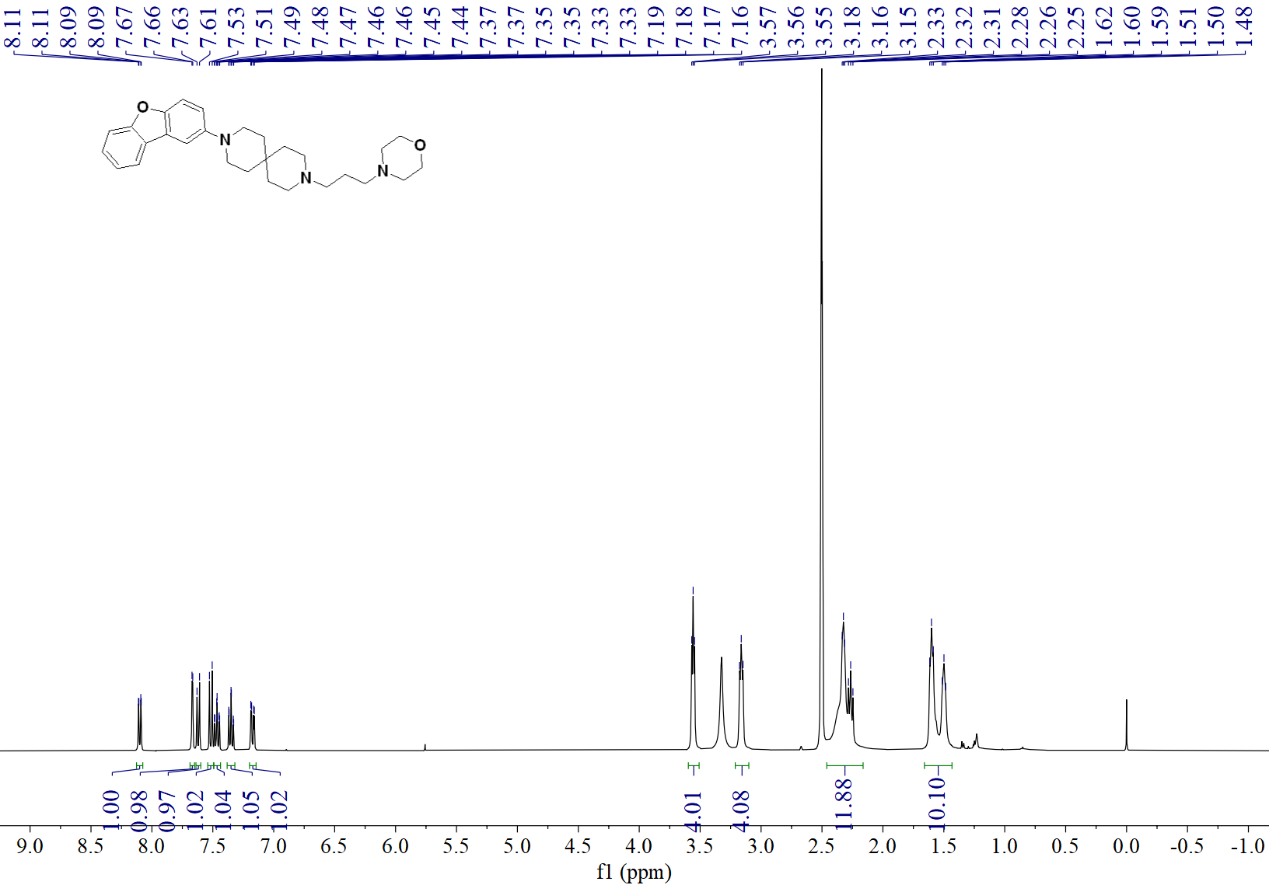


^1^H spectrum of compound **JX3231** (DMSO-*d_6_*)


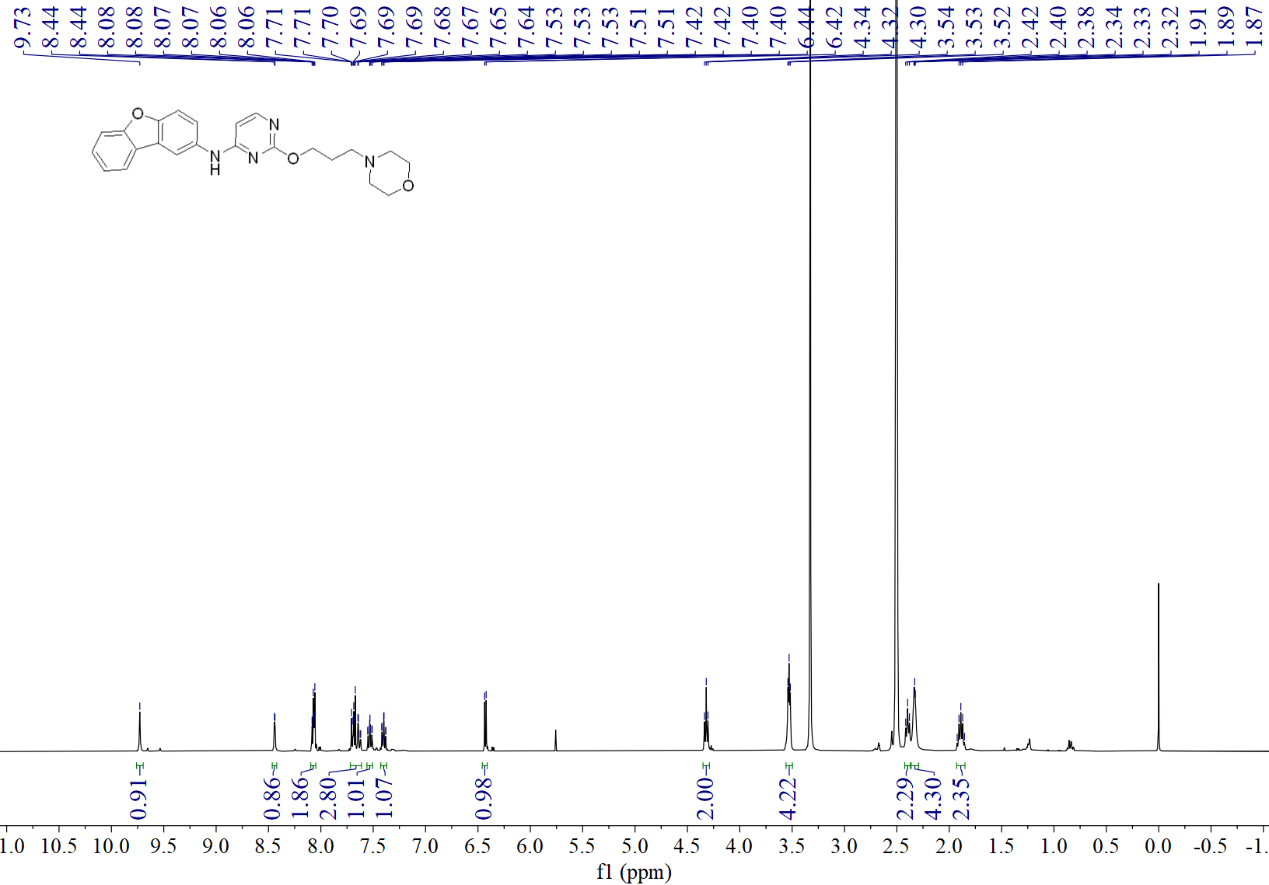


^1^H spectrum of compound **JX3232** (DMSO-*d_6_*)


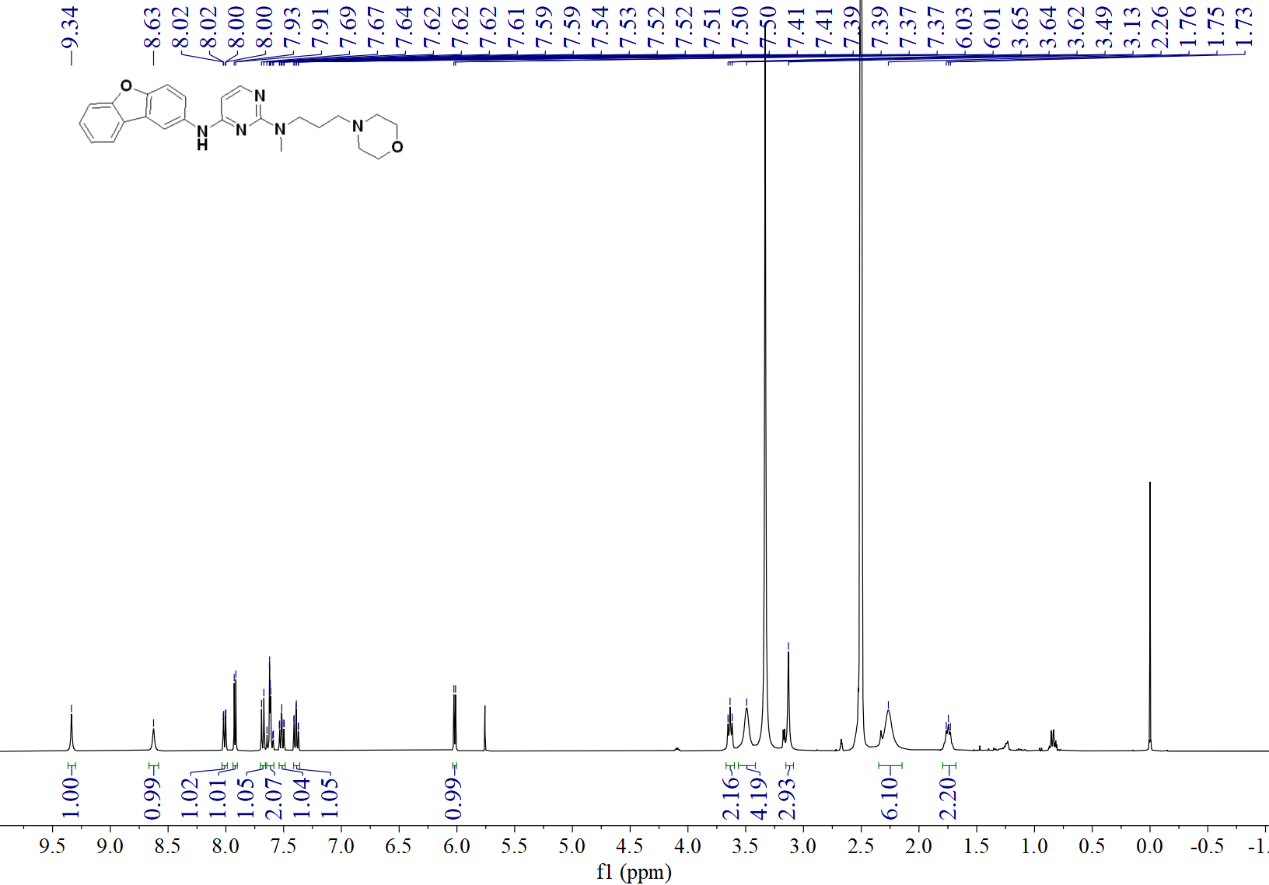


^1^H spectrum of compound **JX3233** (DMSO-*d_6_*)


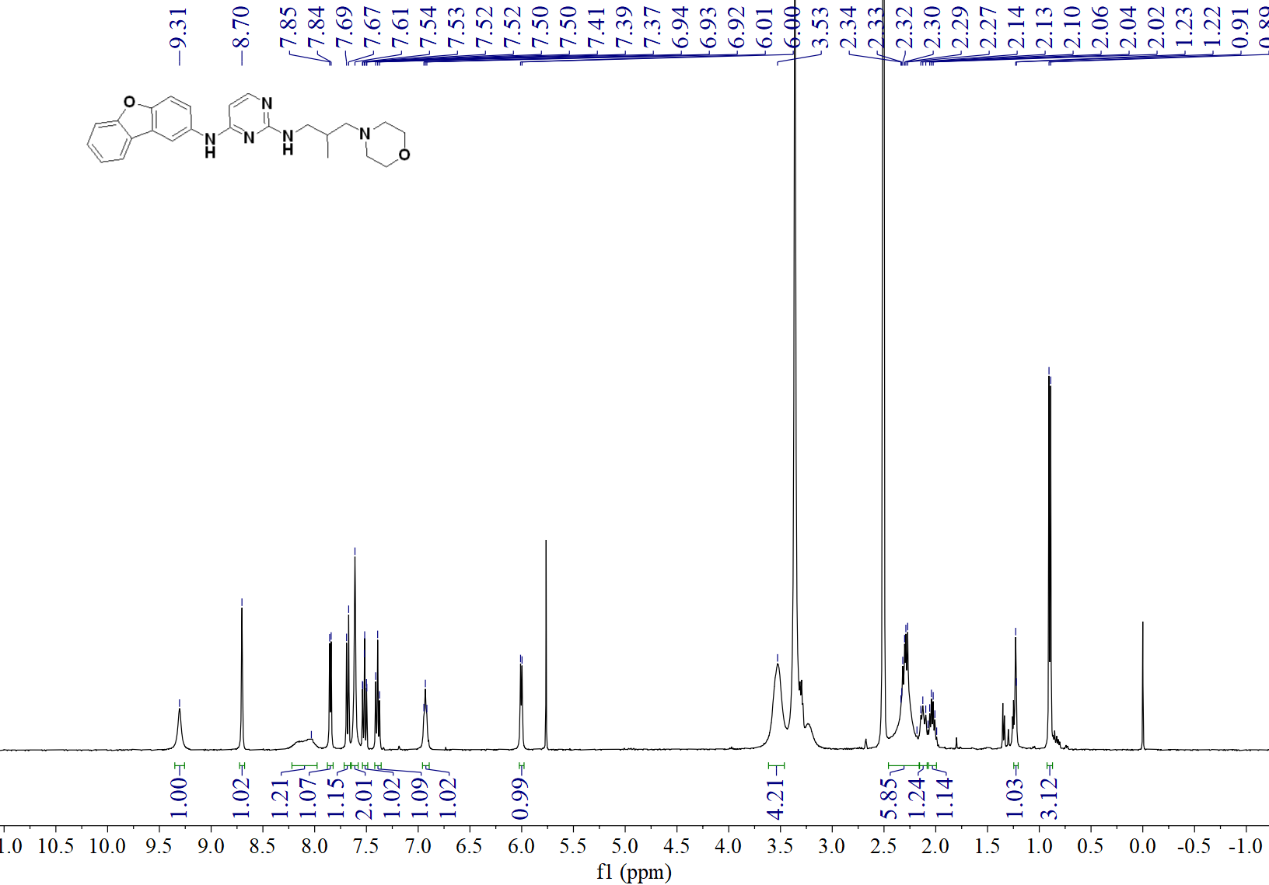


^1^H spectrum of compound **JX3234** (DMSO-*d_6_*)


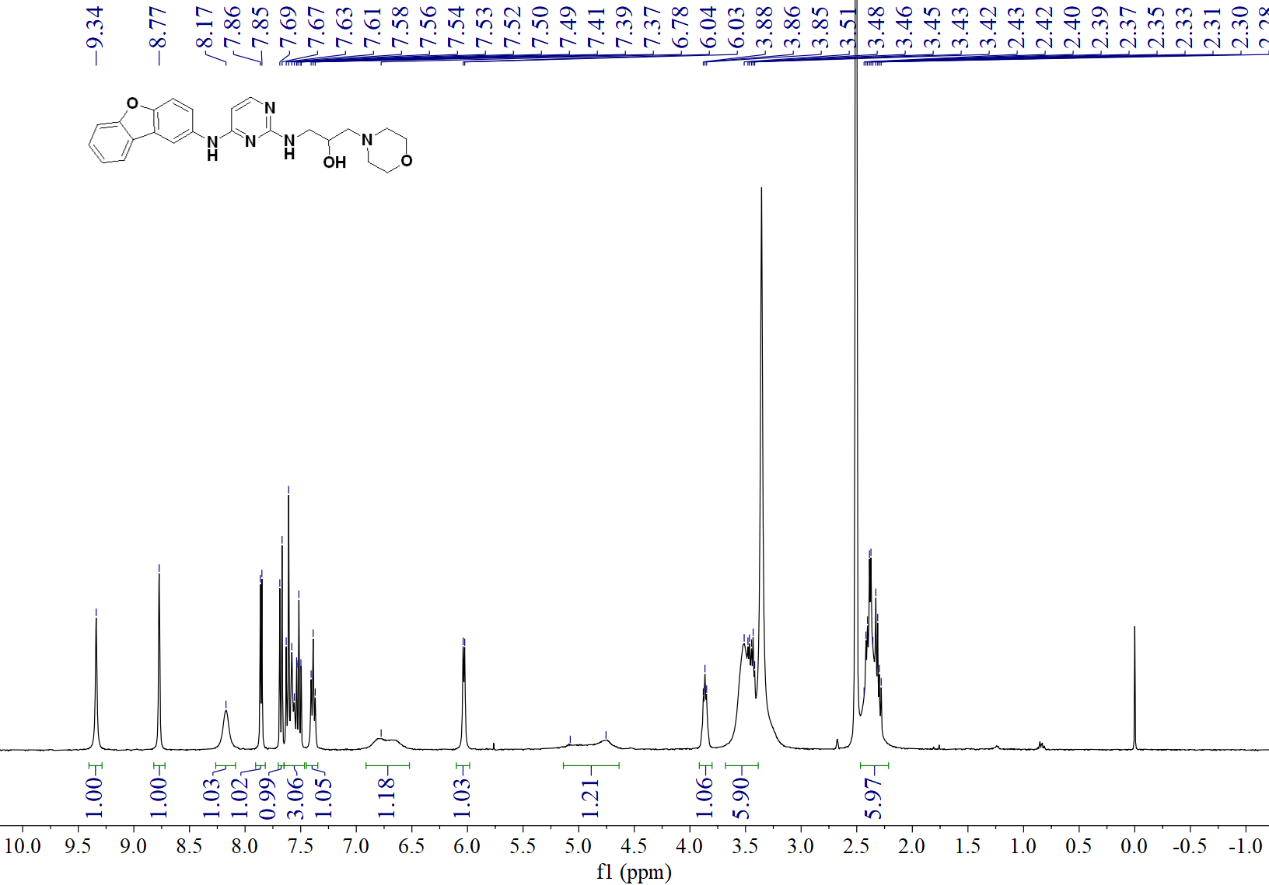


^1^H spectrum of compound **JX3235** (DMSO-*d_6_*)


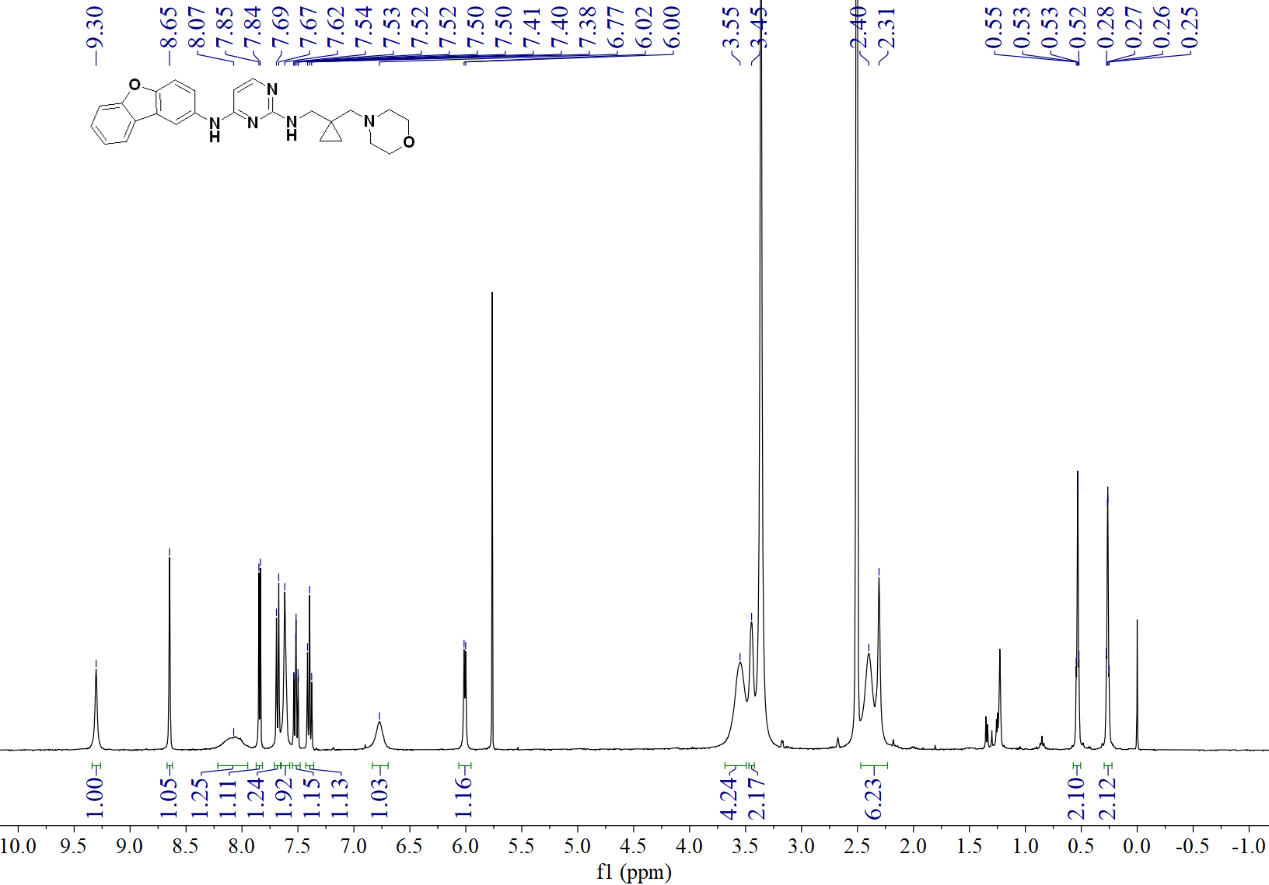


^1^H spectrum of compound **JX3236** (DMSO-*d_6_*)


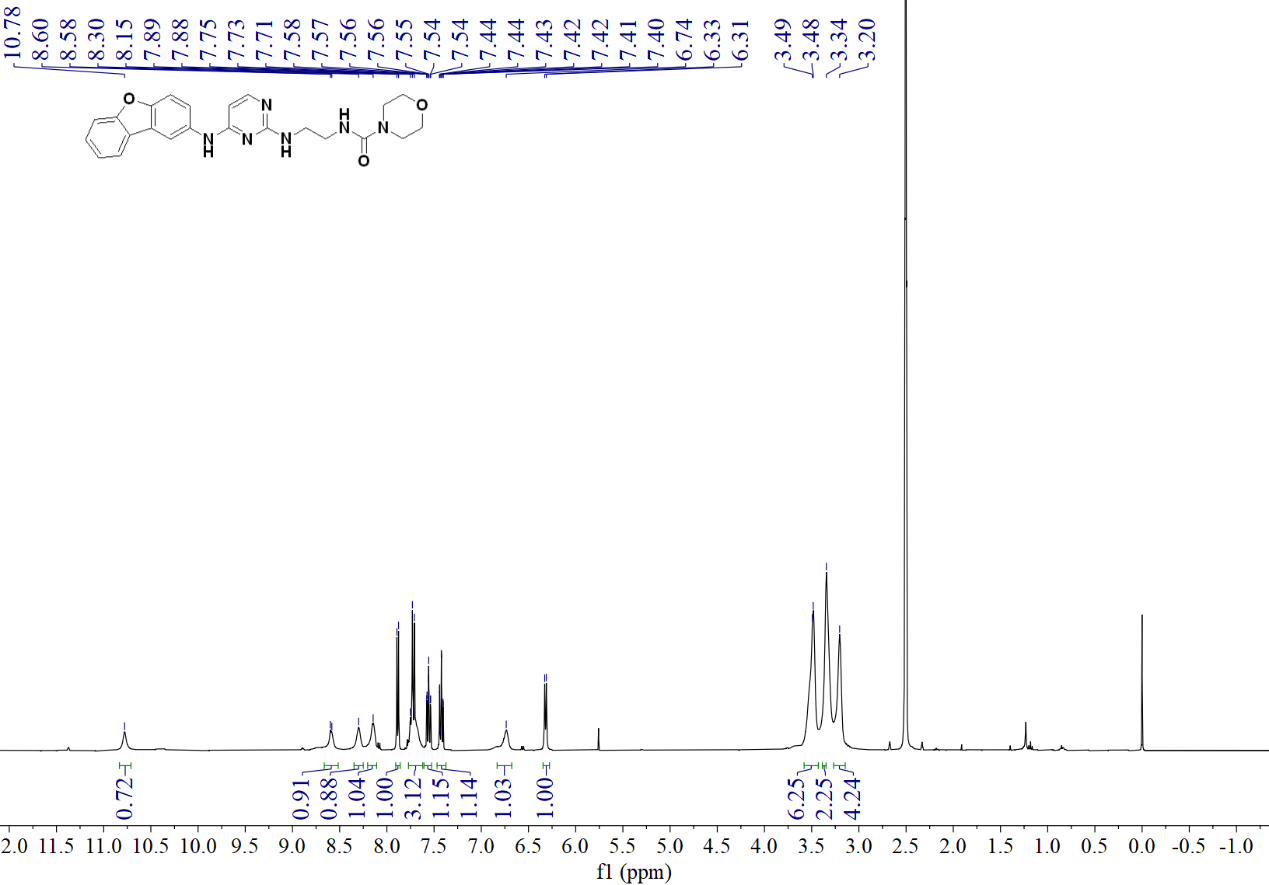


^1^H spectrum of compound **JX3237** (DMSO-*d_6_*)


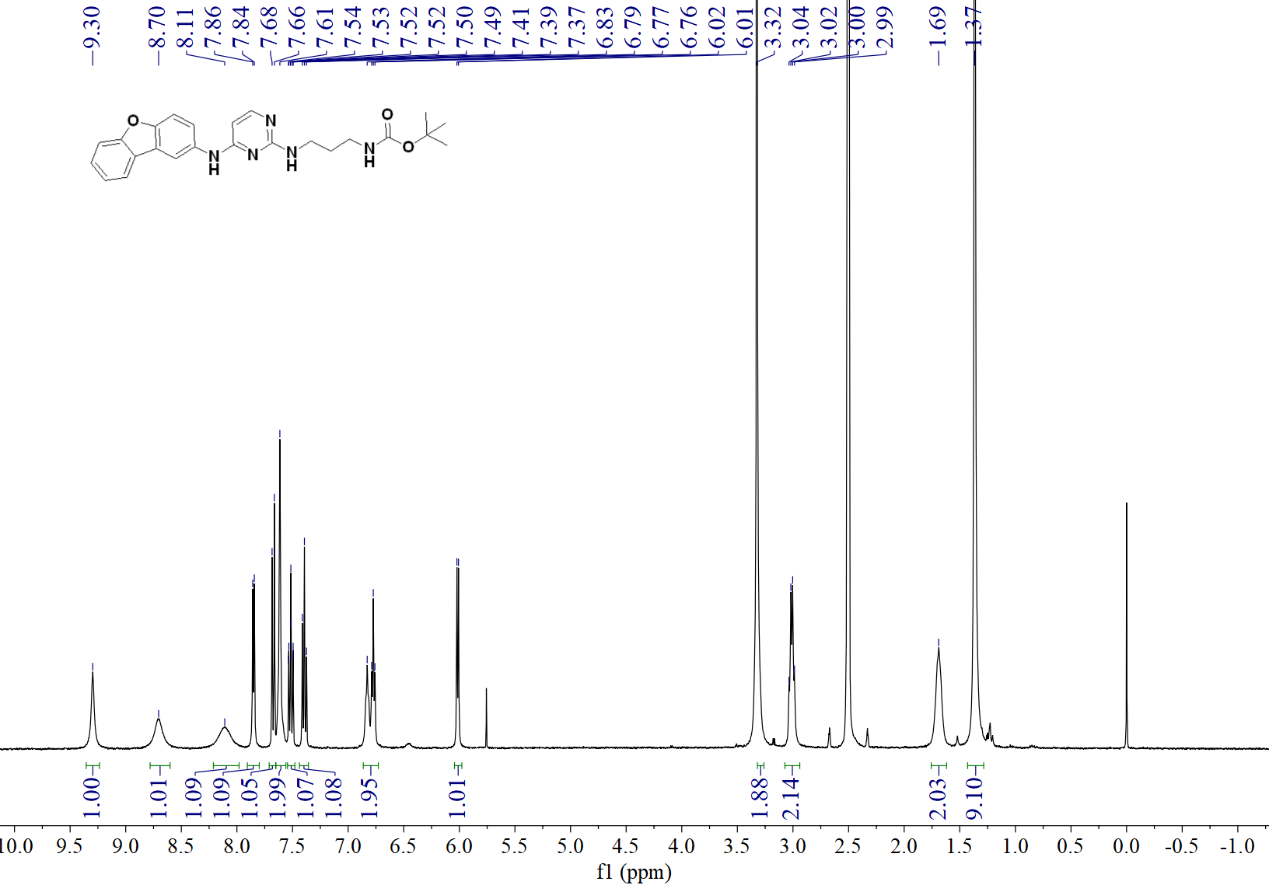


^1^H spectrum of compound **JX3238** (DMSO-*d_6_*)


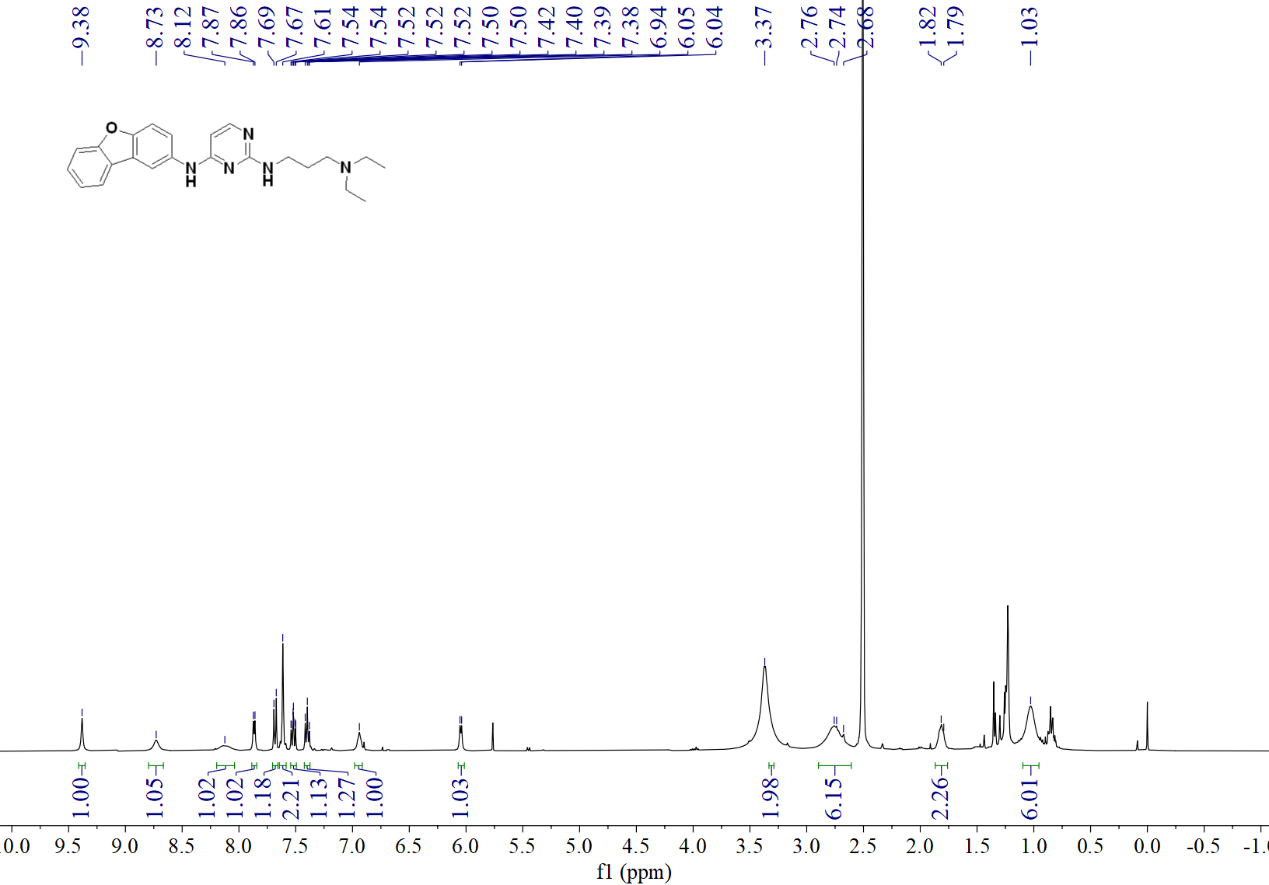


^1^H spectrum of compound **JX3239** (DMSO-*d_6_*)


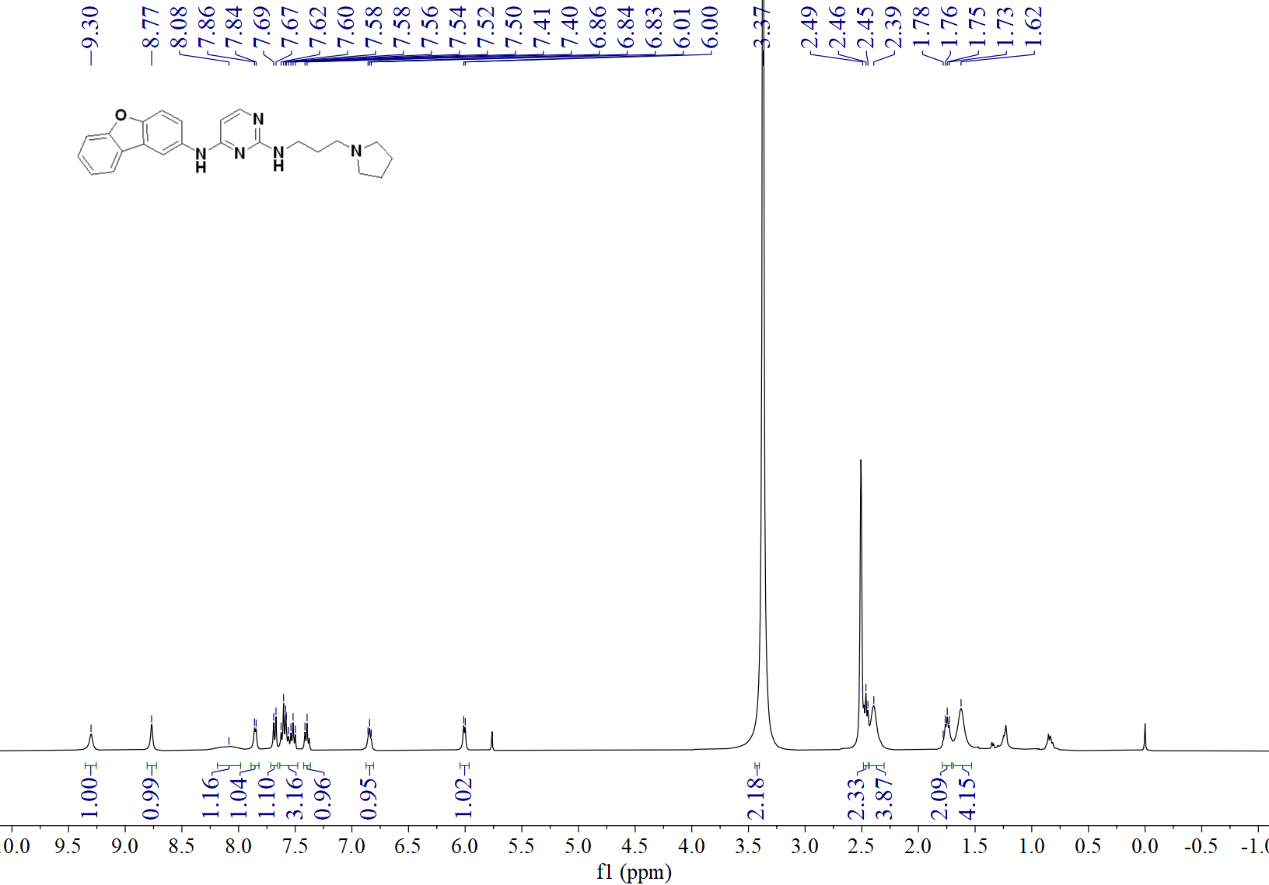


^1^H spectrum of compound **JX3240** (DMSO-*d_6_*)


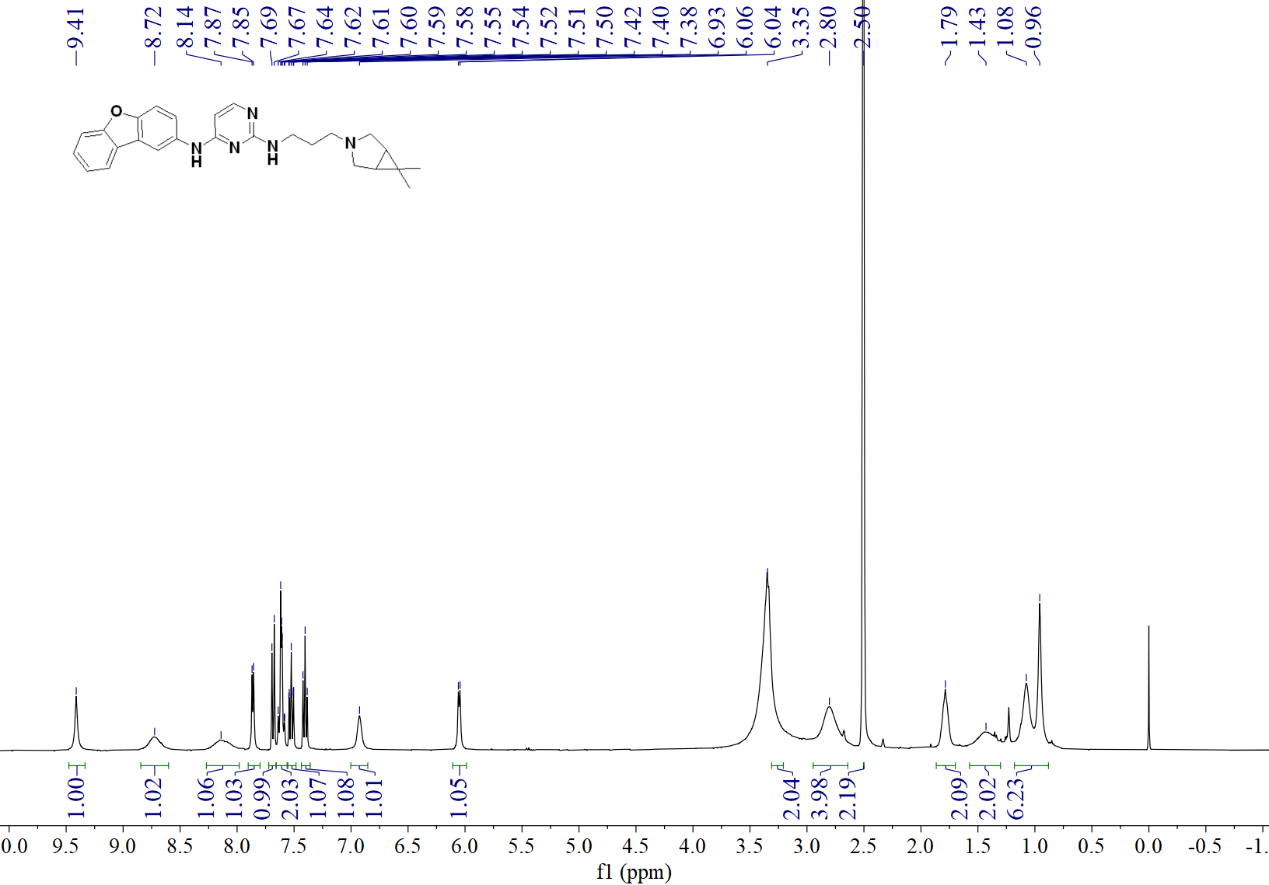


^1^H spectrum of compound **JX3241** (DMSO-*d_6_*)


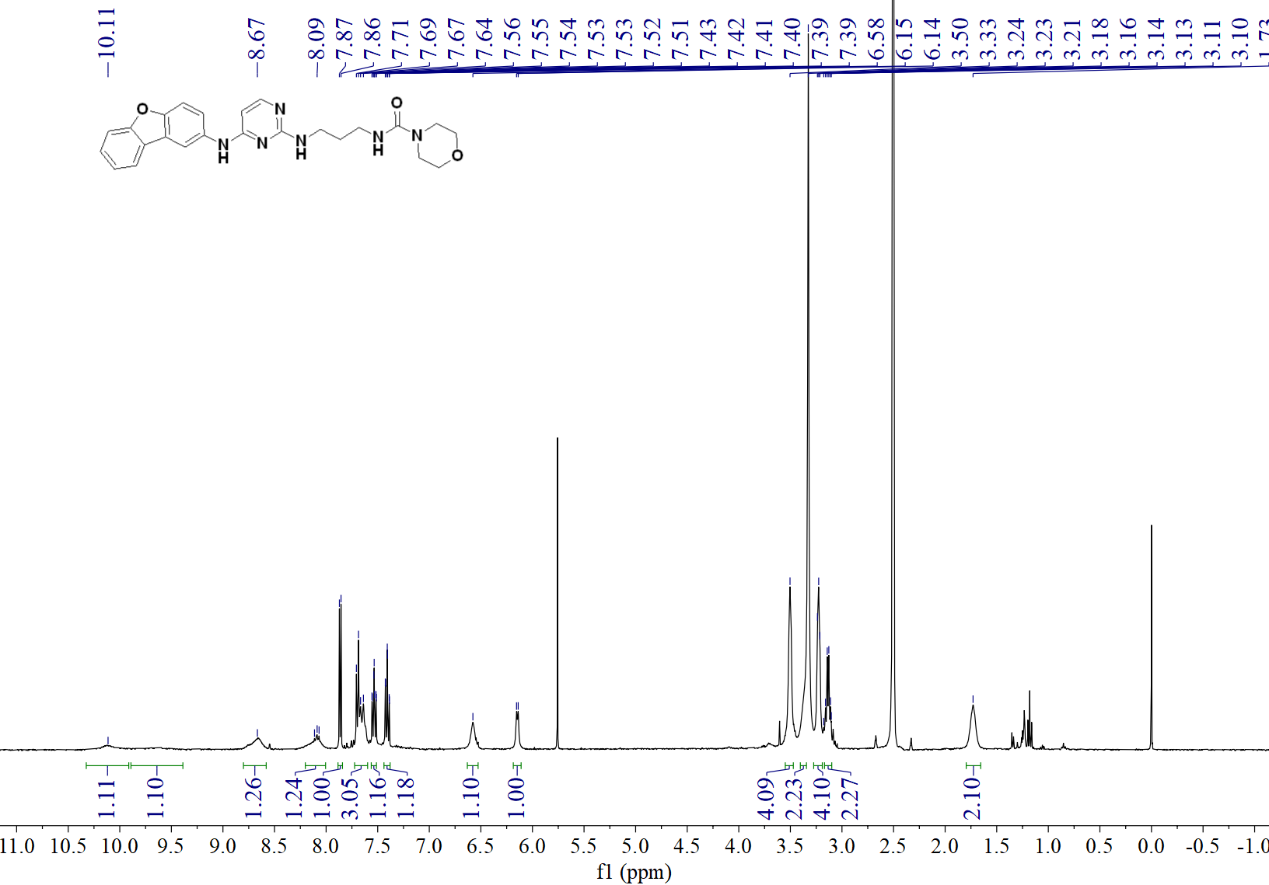


^1^H spectrum of compound **JX3242** (DMSO-*d_6_*)


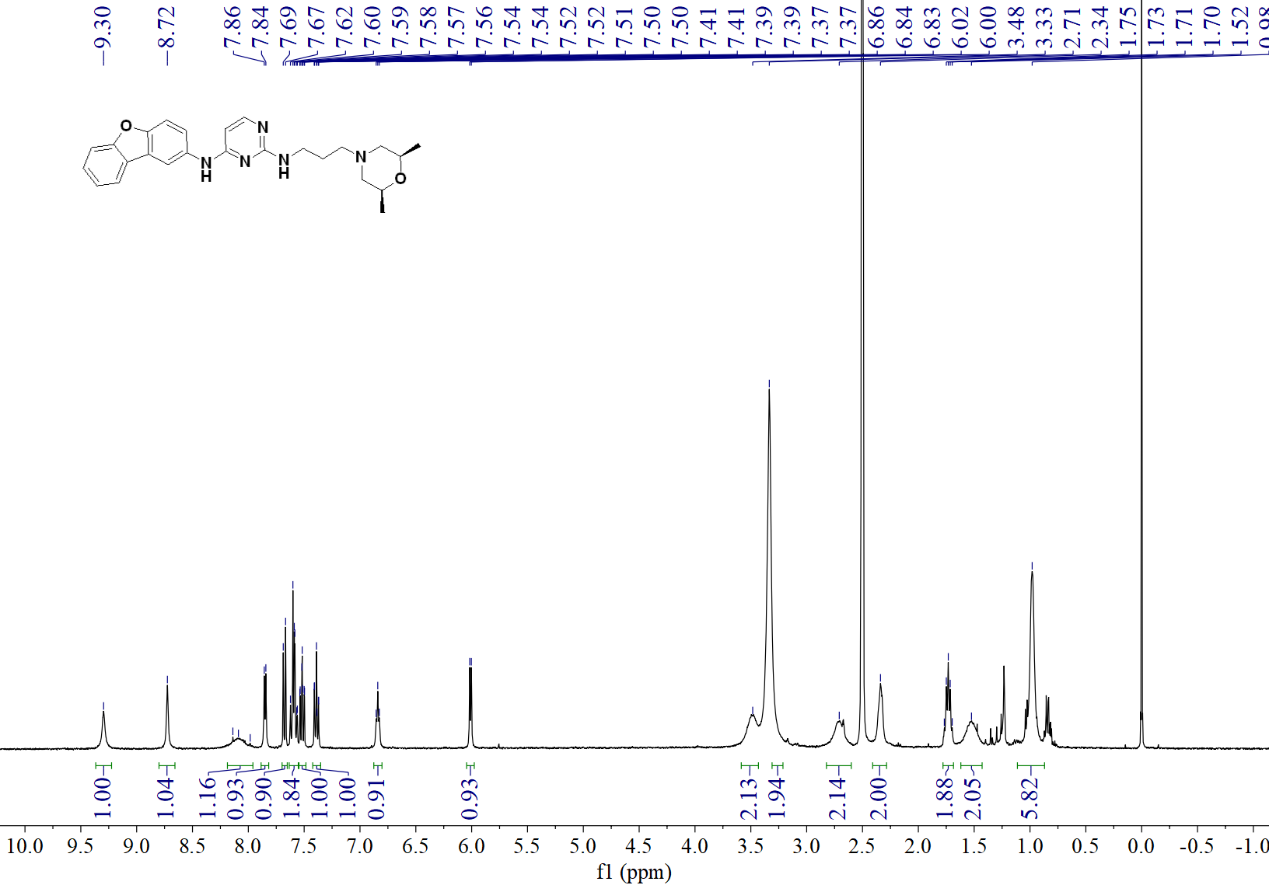


^1^H spectrum of compound **JX3243** (DMSO-*d_6_*)


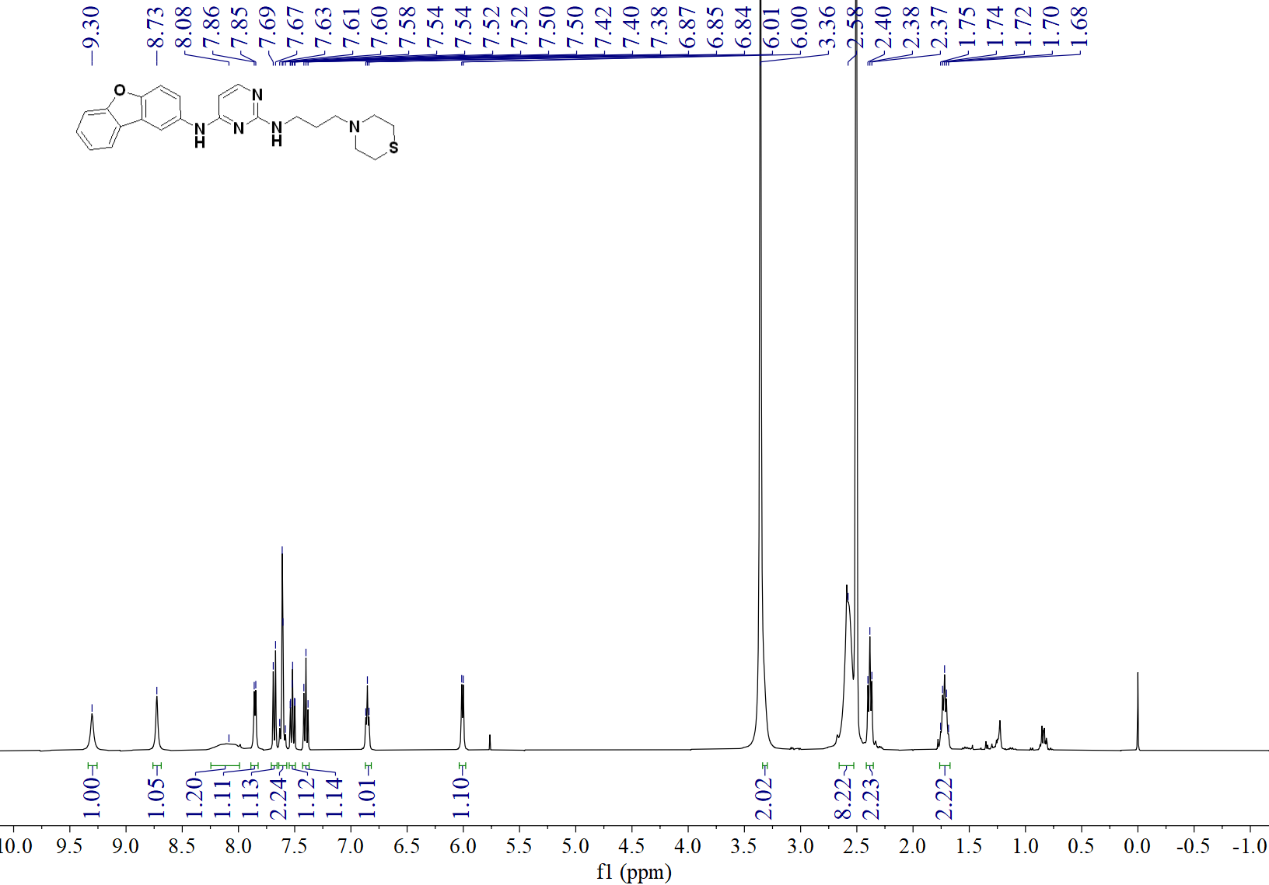


^1^H spectrum of compound **JX3244** (DMSO-*d_6_*)


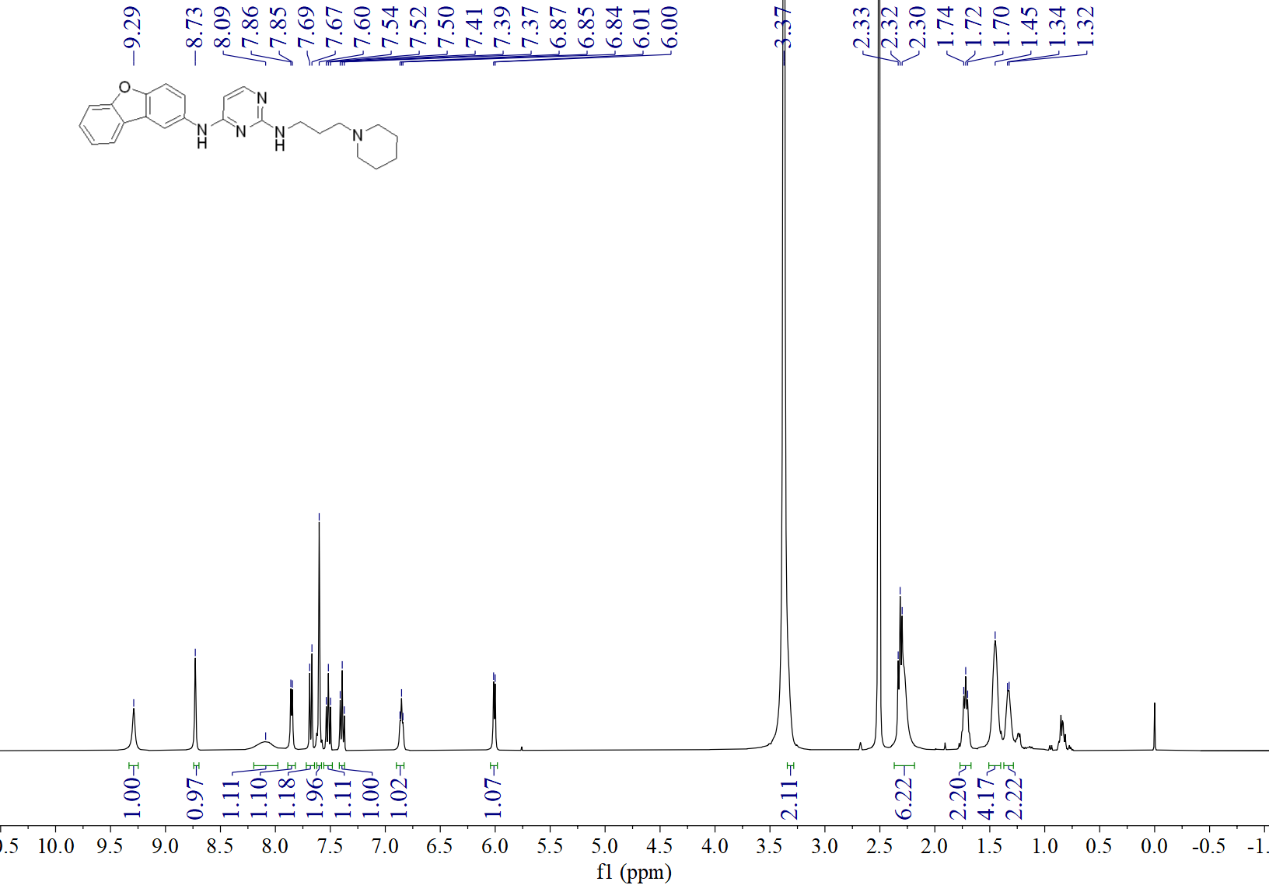


^1^H spectrum of compound **JX3245** (DMSO-*d_6_*)


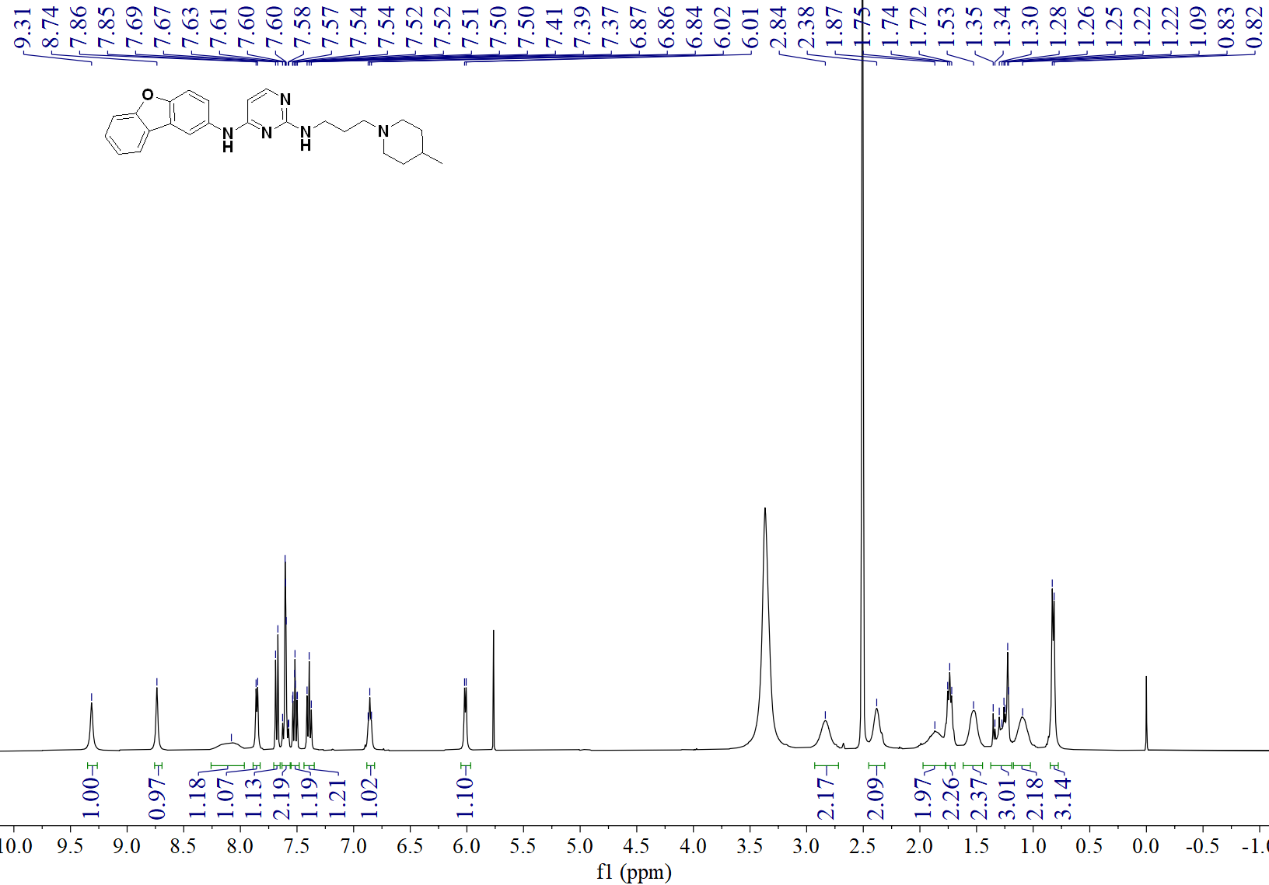


^1^H spectrum of compound **JX3246** (DMSO-*d_6_*)


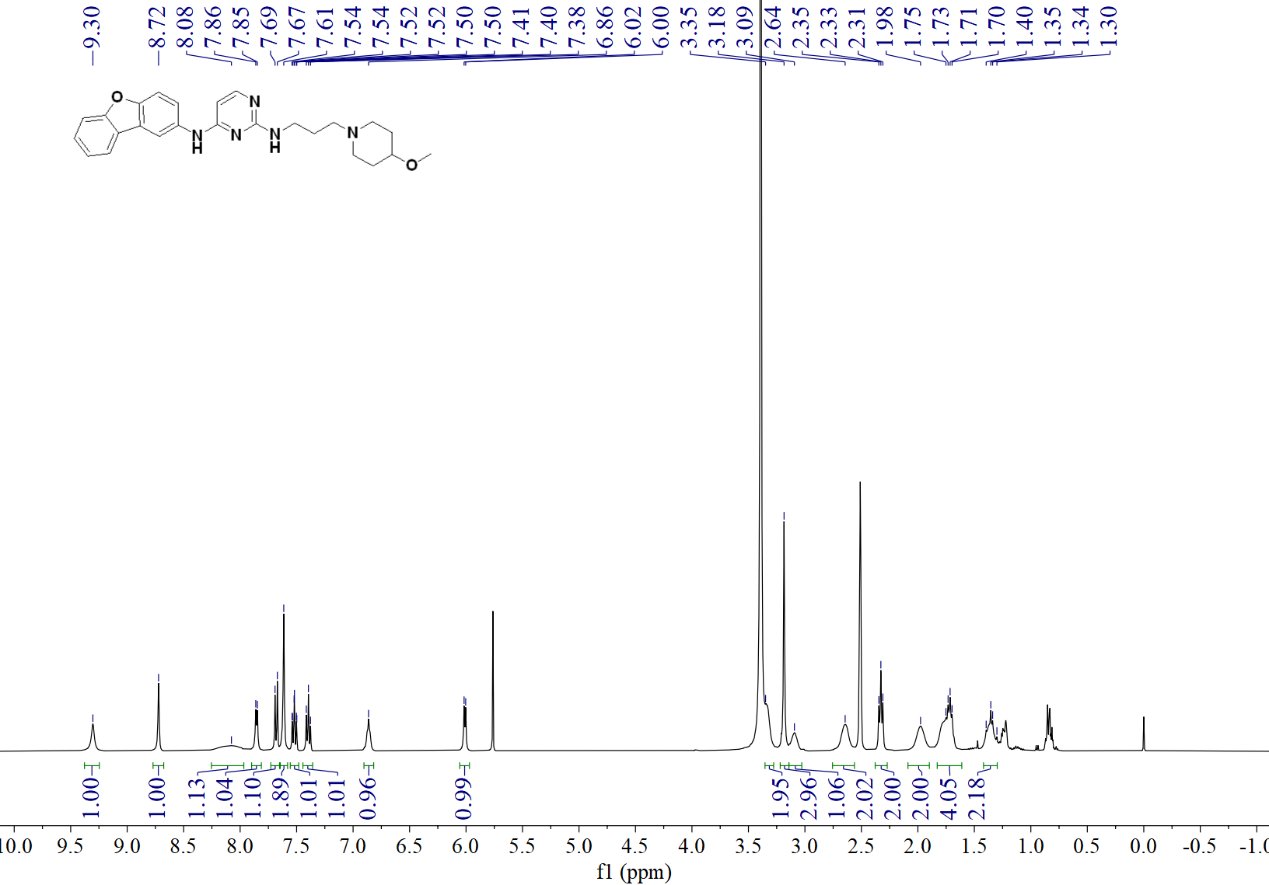


^1^H spectrum of compound **JX3247** (DMSO-*d_6_*)


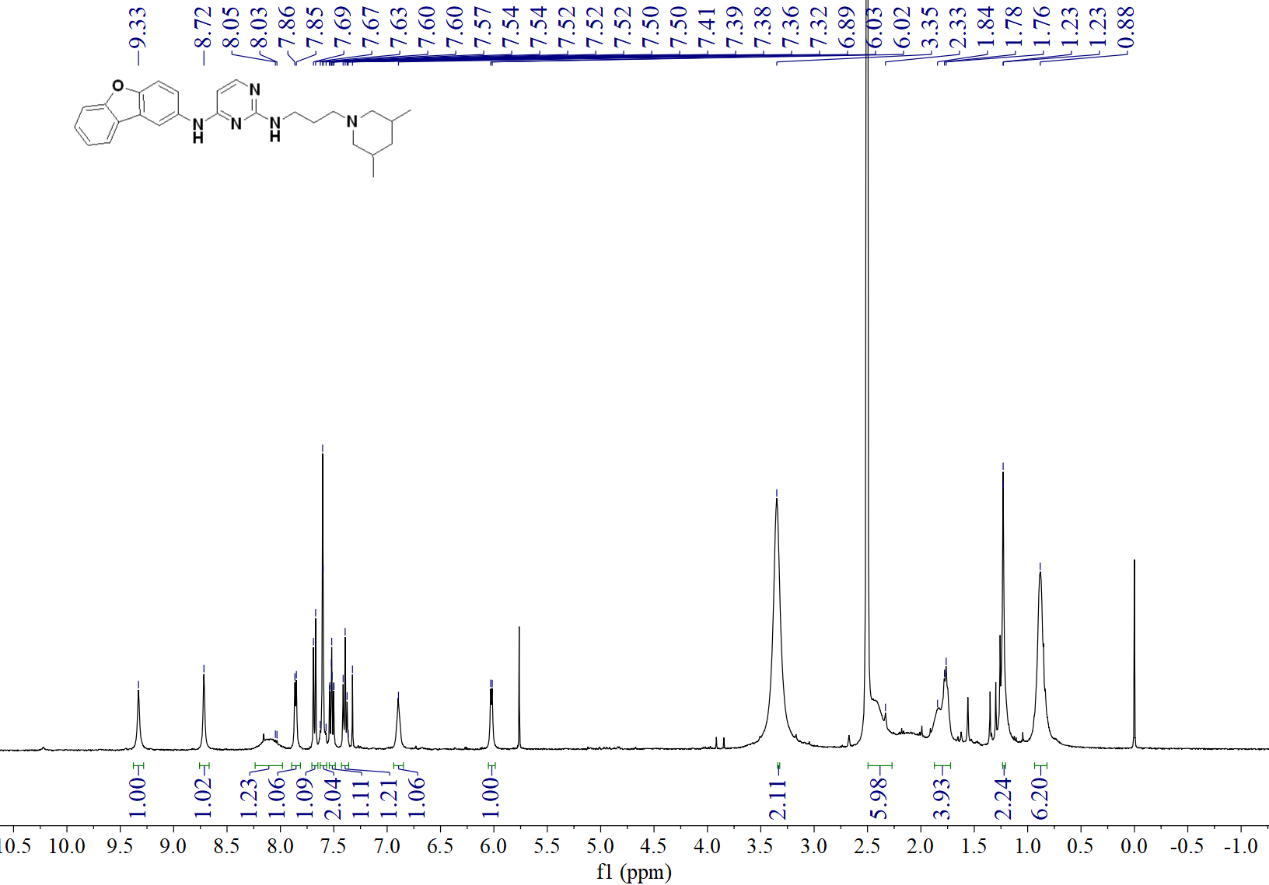


^1^H spectrum of compound **JX3248** (DMSO-*d_6_*)


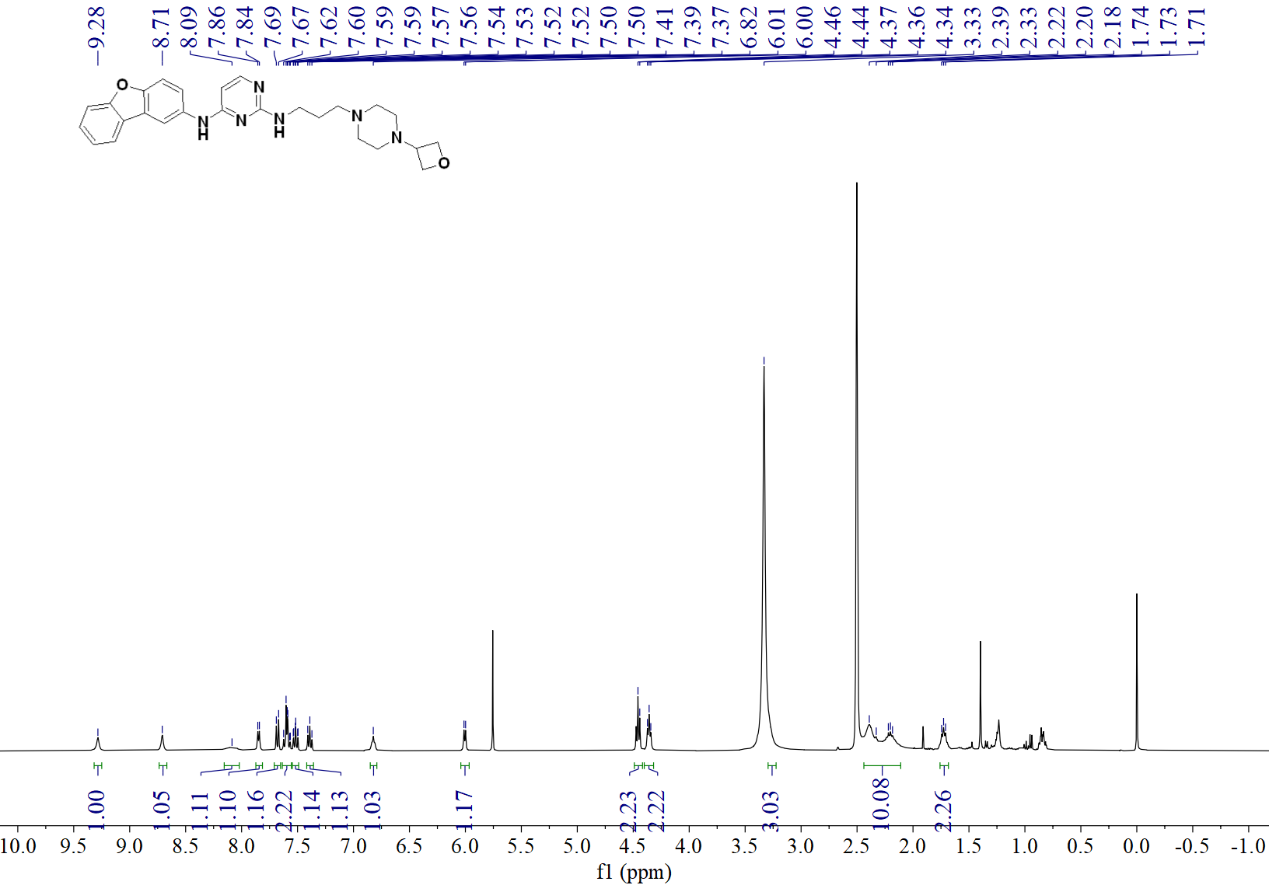


^1^H spectrum of compound **JX3249** (DMSO-*d_6_*)


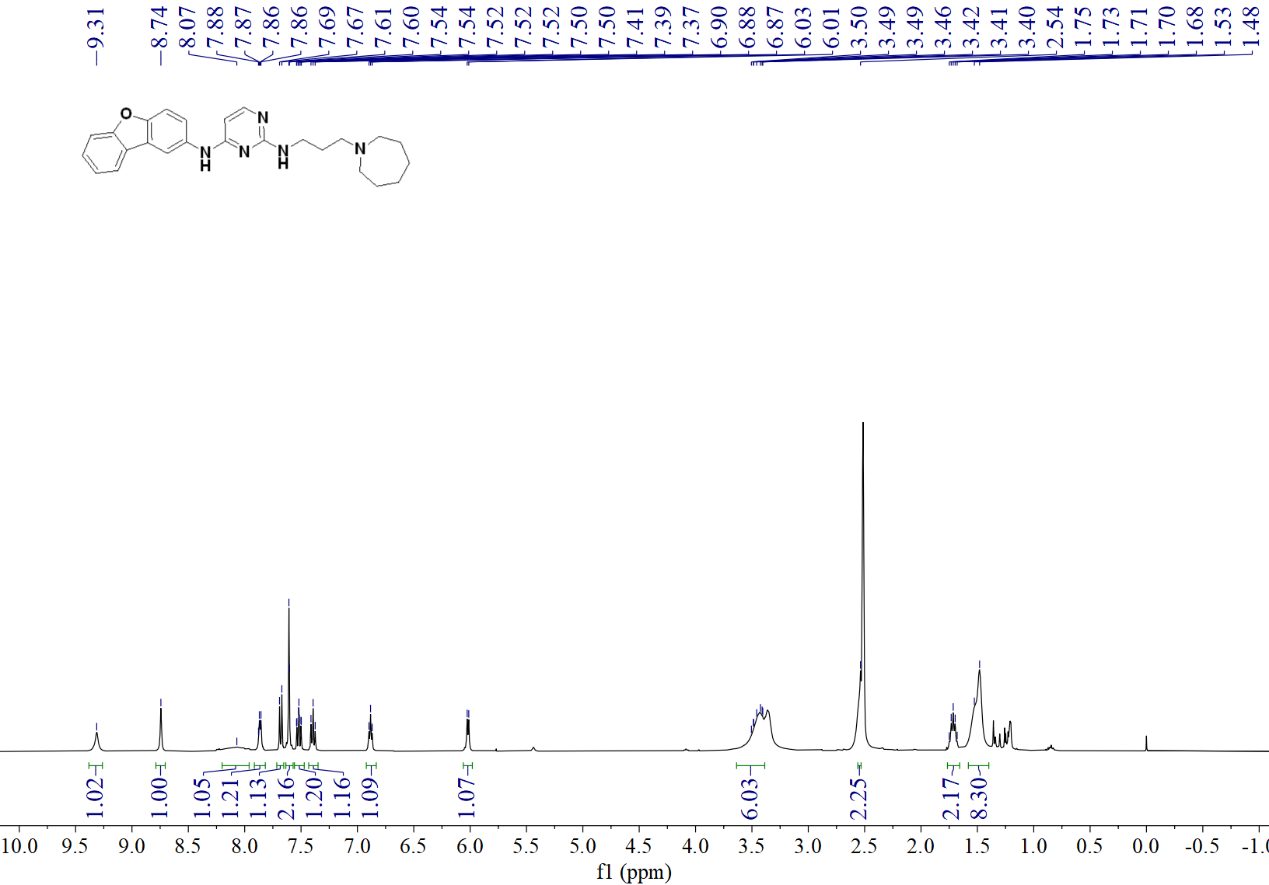


^1^H spectrum of compound **JX3250** (DMSO-*d_6_*)


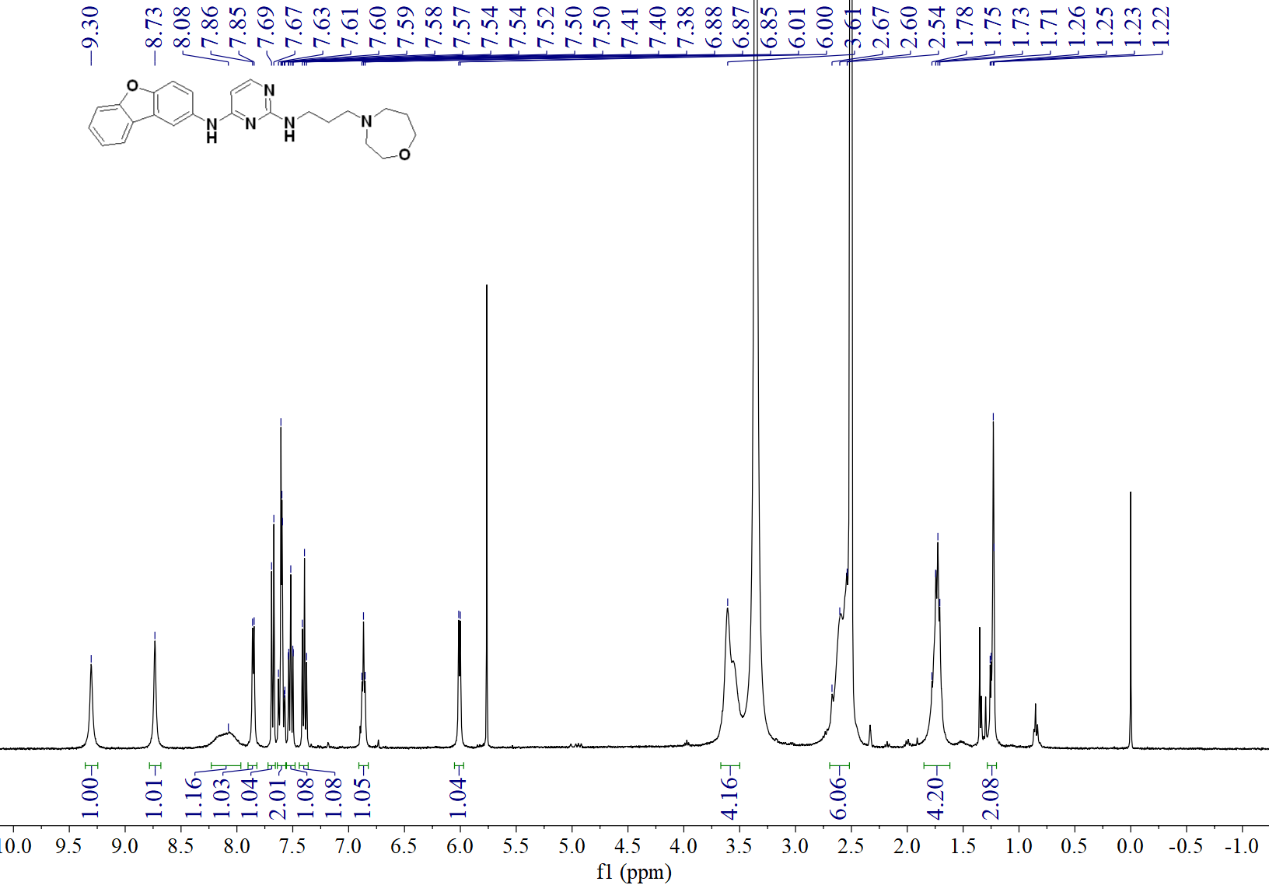


**NMR spectra-^13^C**

^13^C spectrum of compound **JX3201** (DMSO-*d_6_*)


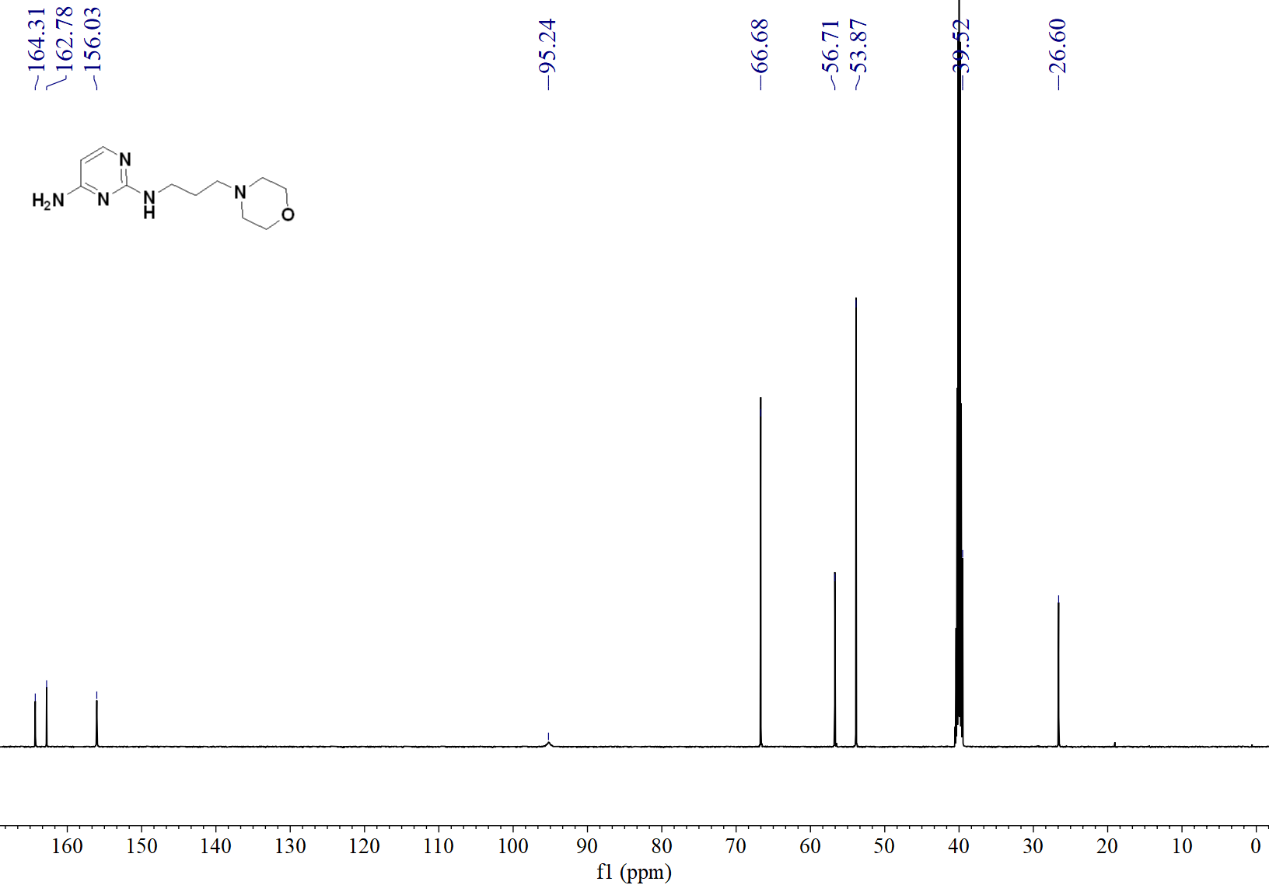


^13^C spectrum of compound **JX3202** (DMSO-*d_6_*)


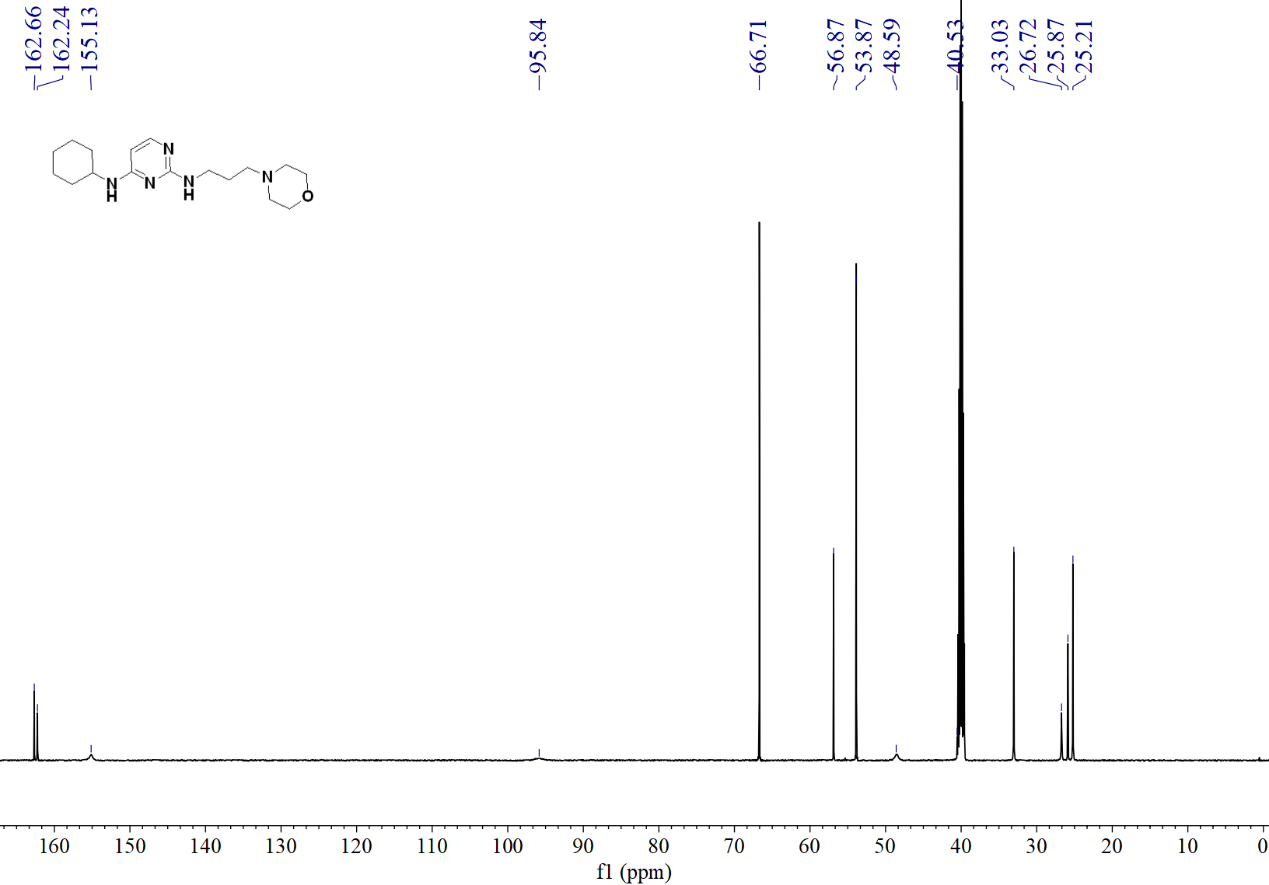


^13^C spectrum of compound **JX3203** (DMSO-*d_6_*)


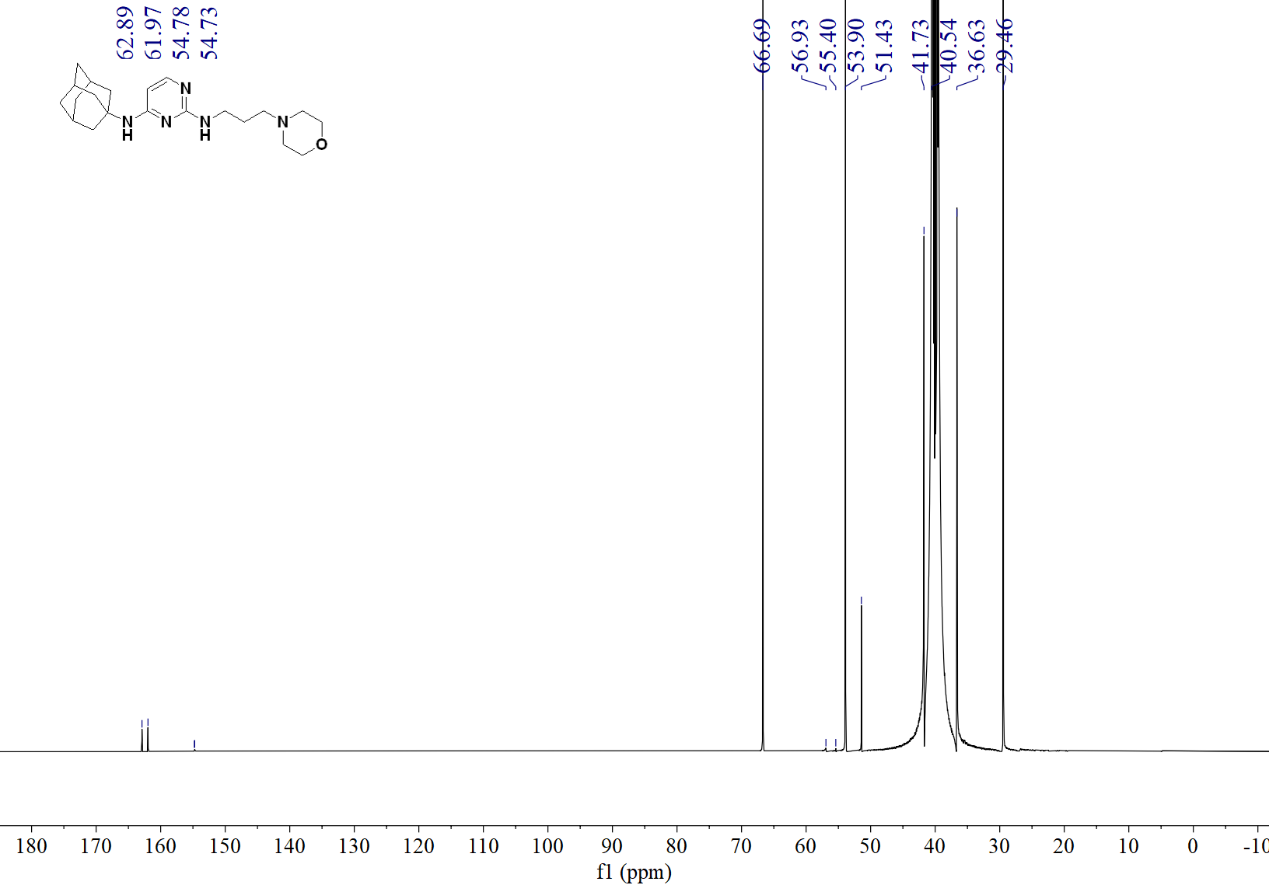


^13^C spectrum of compound **JX3204** (DMSO-*d_6_*)


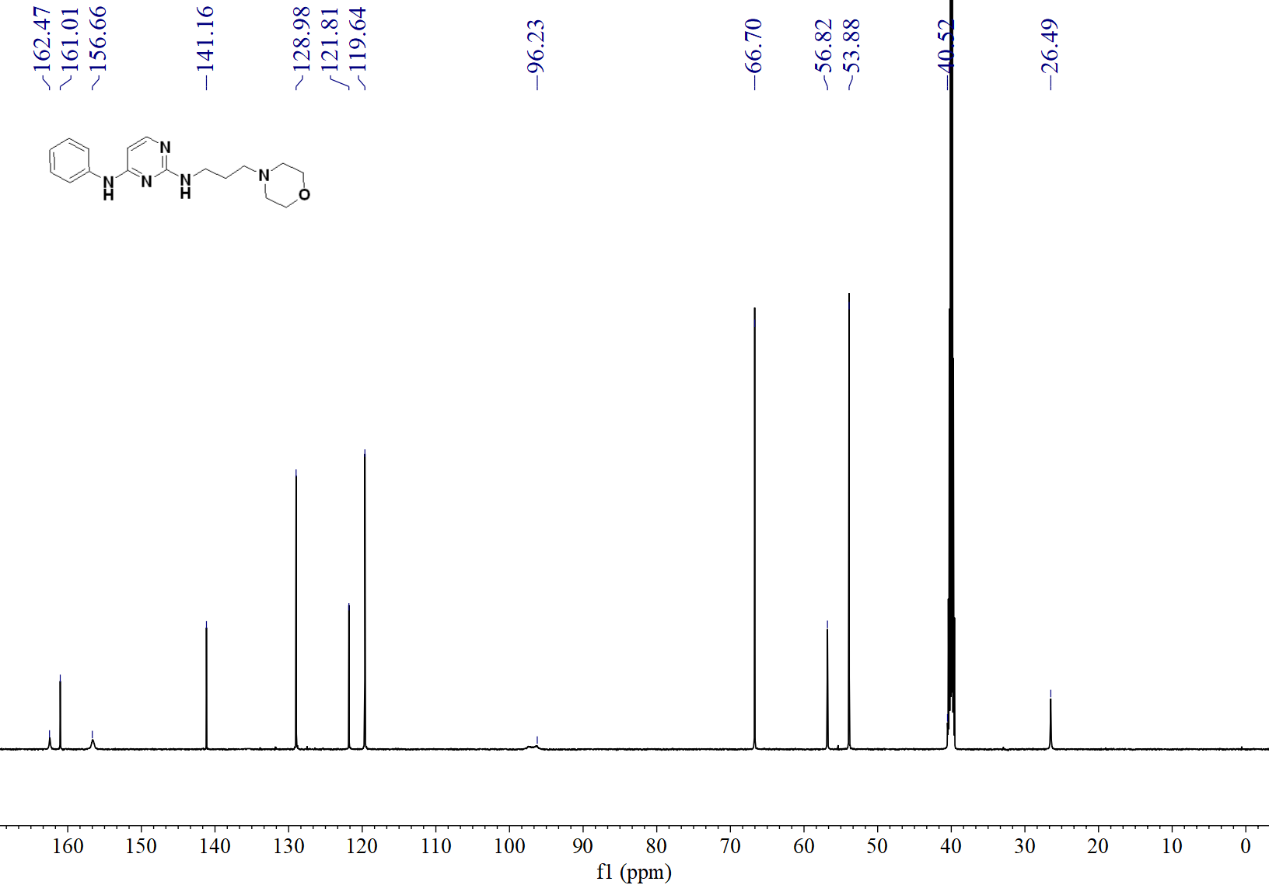


^13^C spectrum of compound **JX3205** (DMSO-*d_6_*)


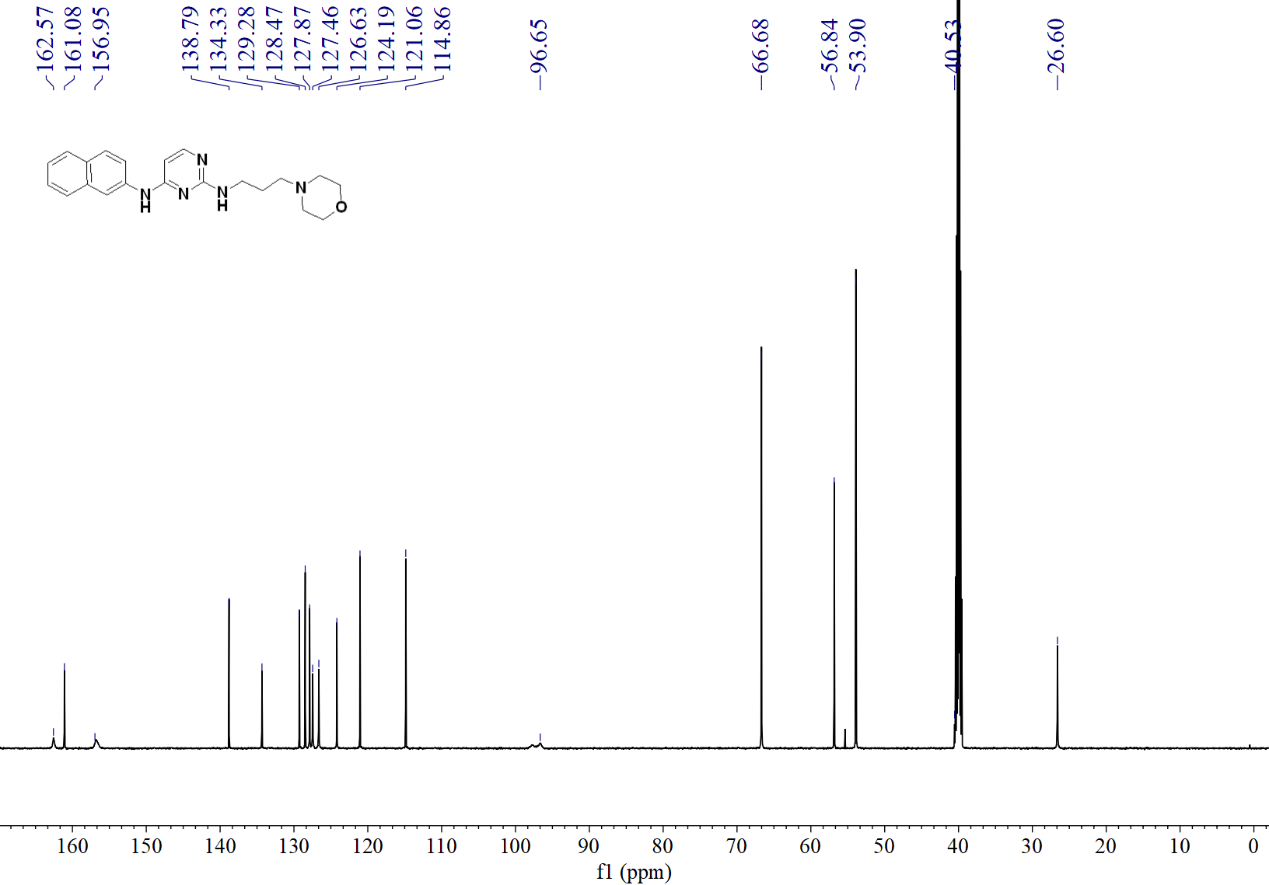


^13^C spectrum of compound **JX3206** (DMSO-*d_6_*)


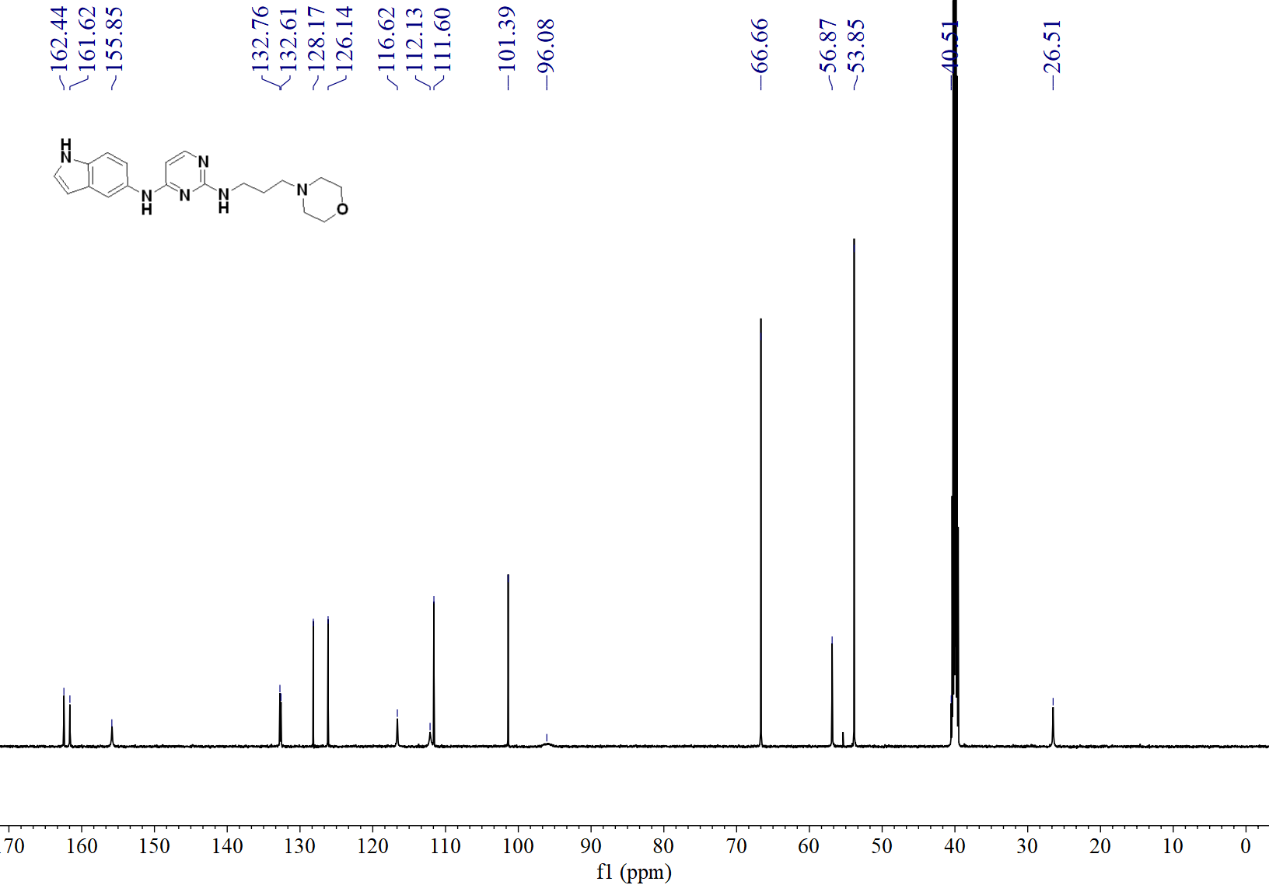


^13^C spectrum of compound **JX3207** (DMSO-*d_6_*)


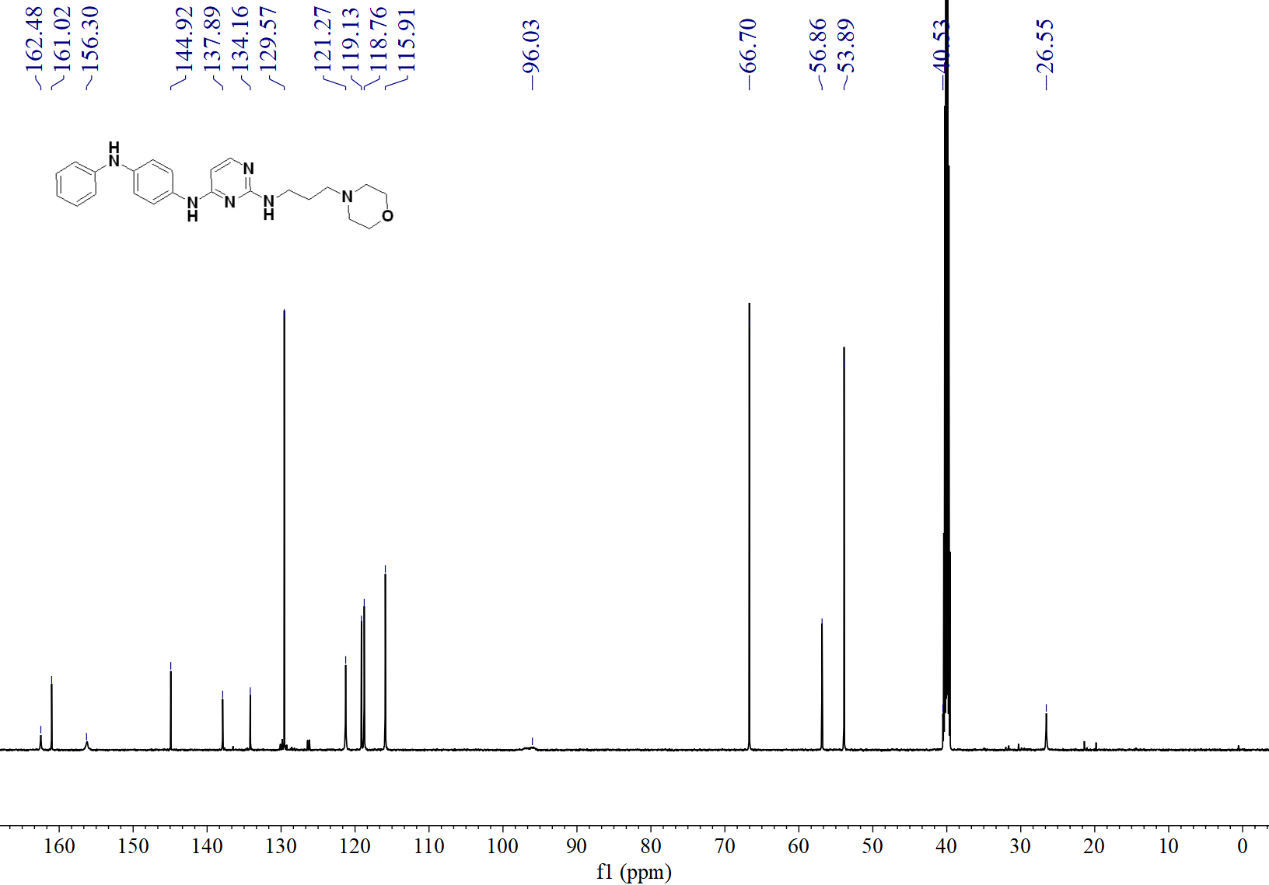


^13^C spectrum of compound **JX3208** (DMSO-*d_6_*)


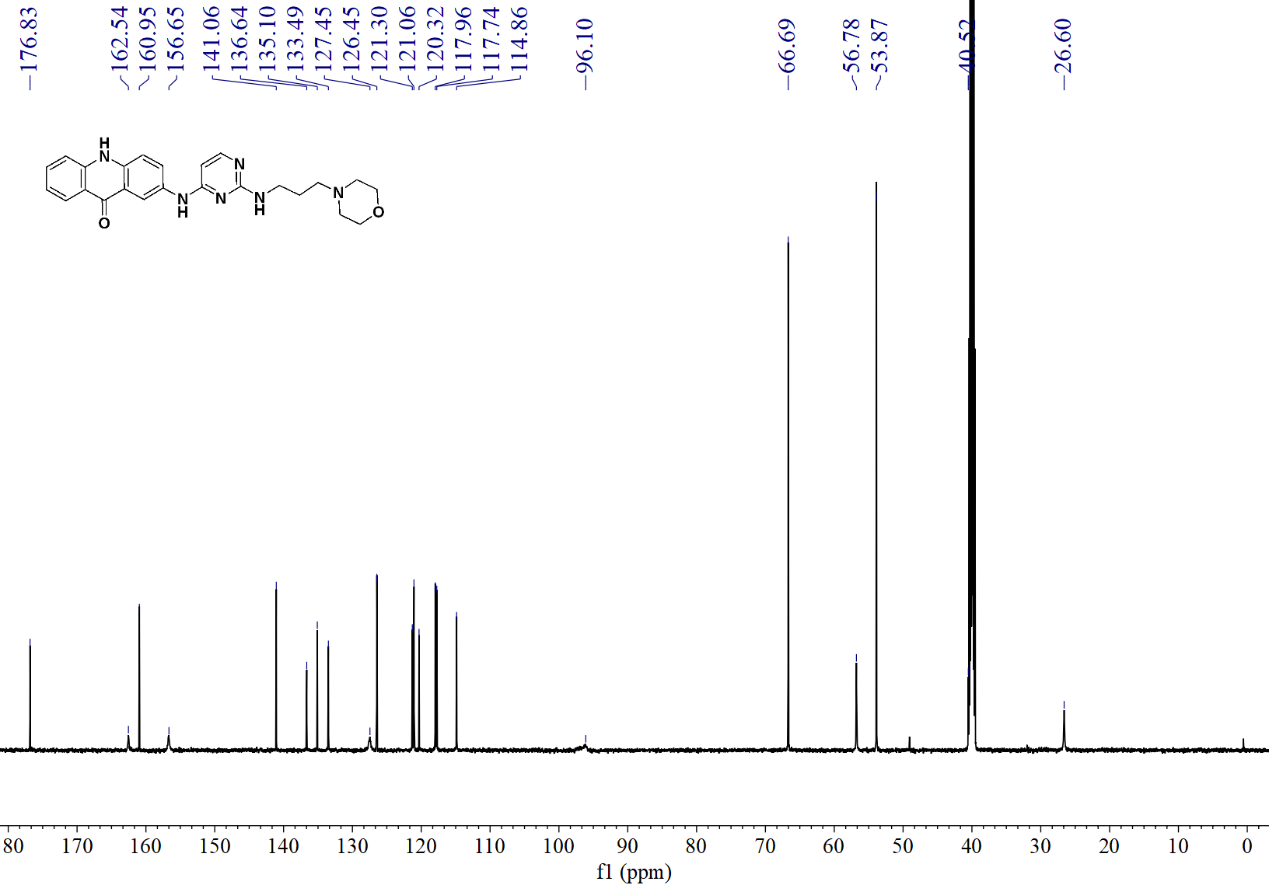


^13^C spectrum of compound **JX3209** (DMSO-*d_6_*)


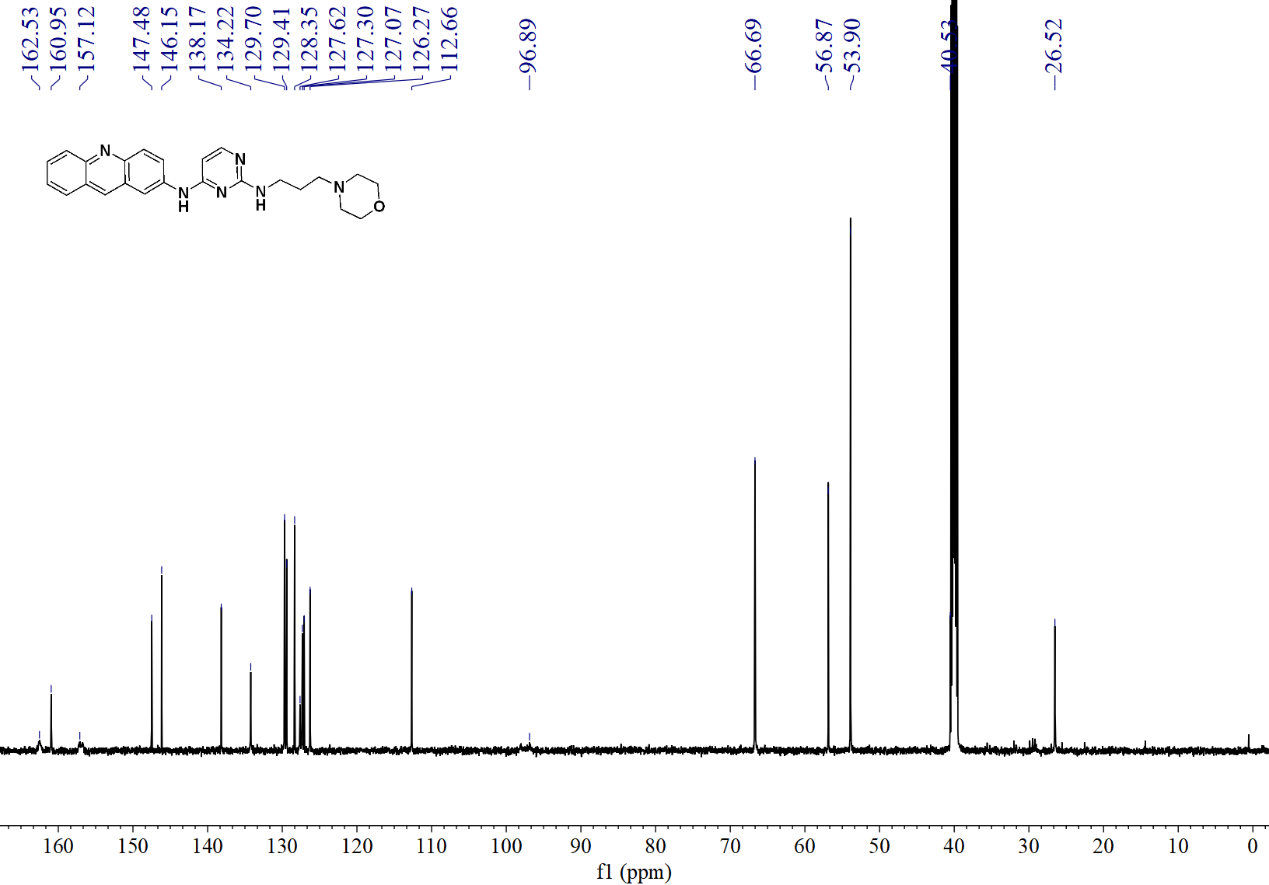


^13^C spectrum of compound **JX3210** (DMSO-*d_6_*)


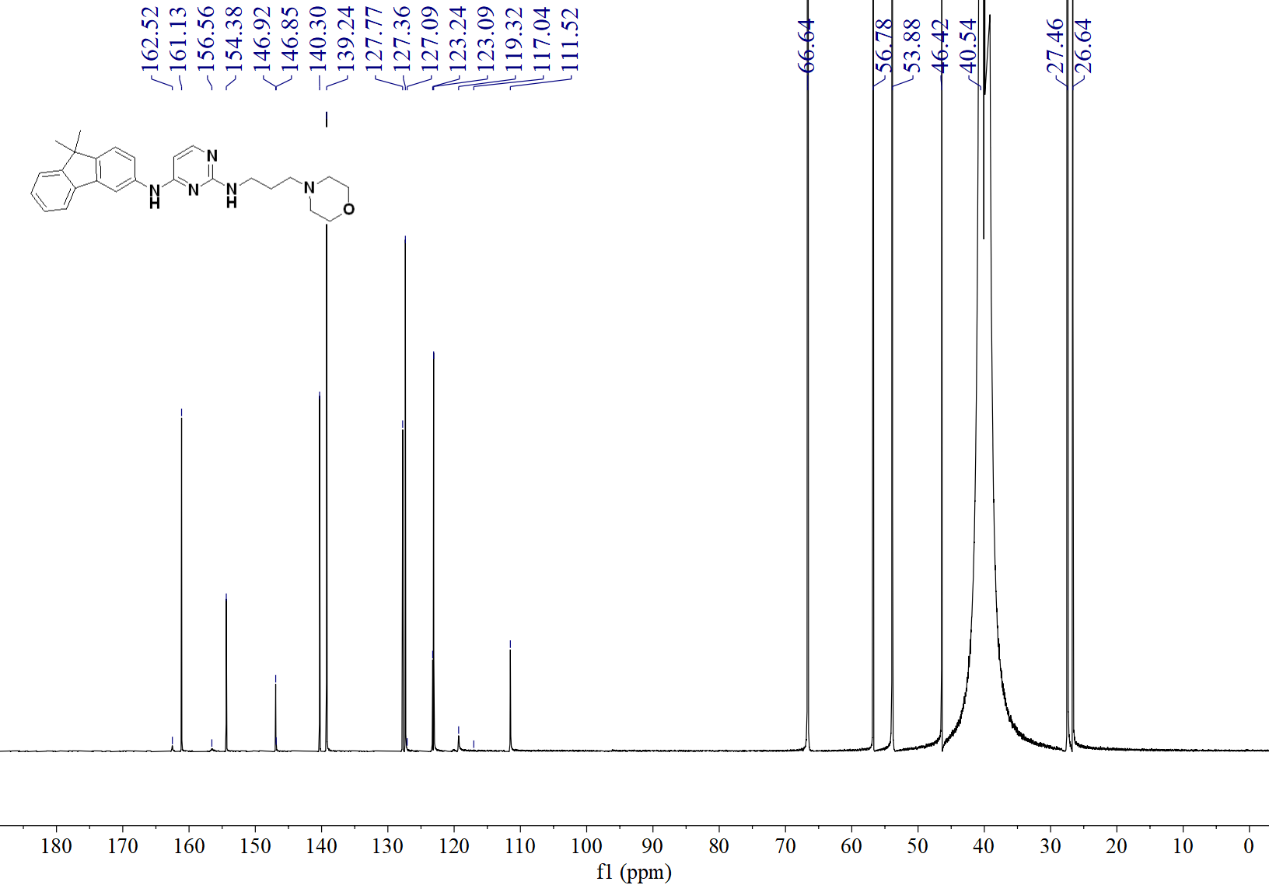


^13^C spectrum of compound **JX3212** (DMSO-*d_6_*)


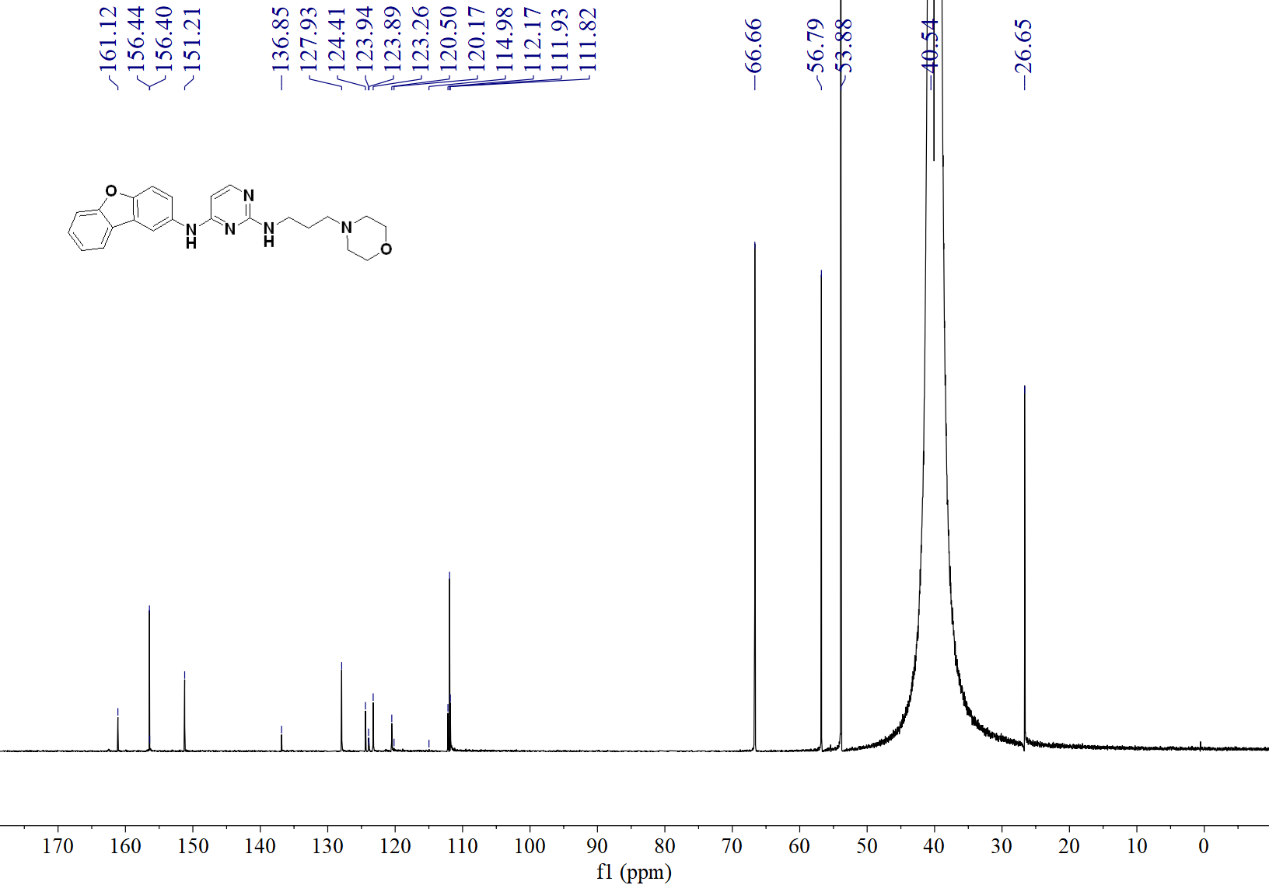


^13^C spectrum of compound **JX3213** (DMSO-*d_6_*)


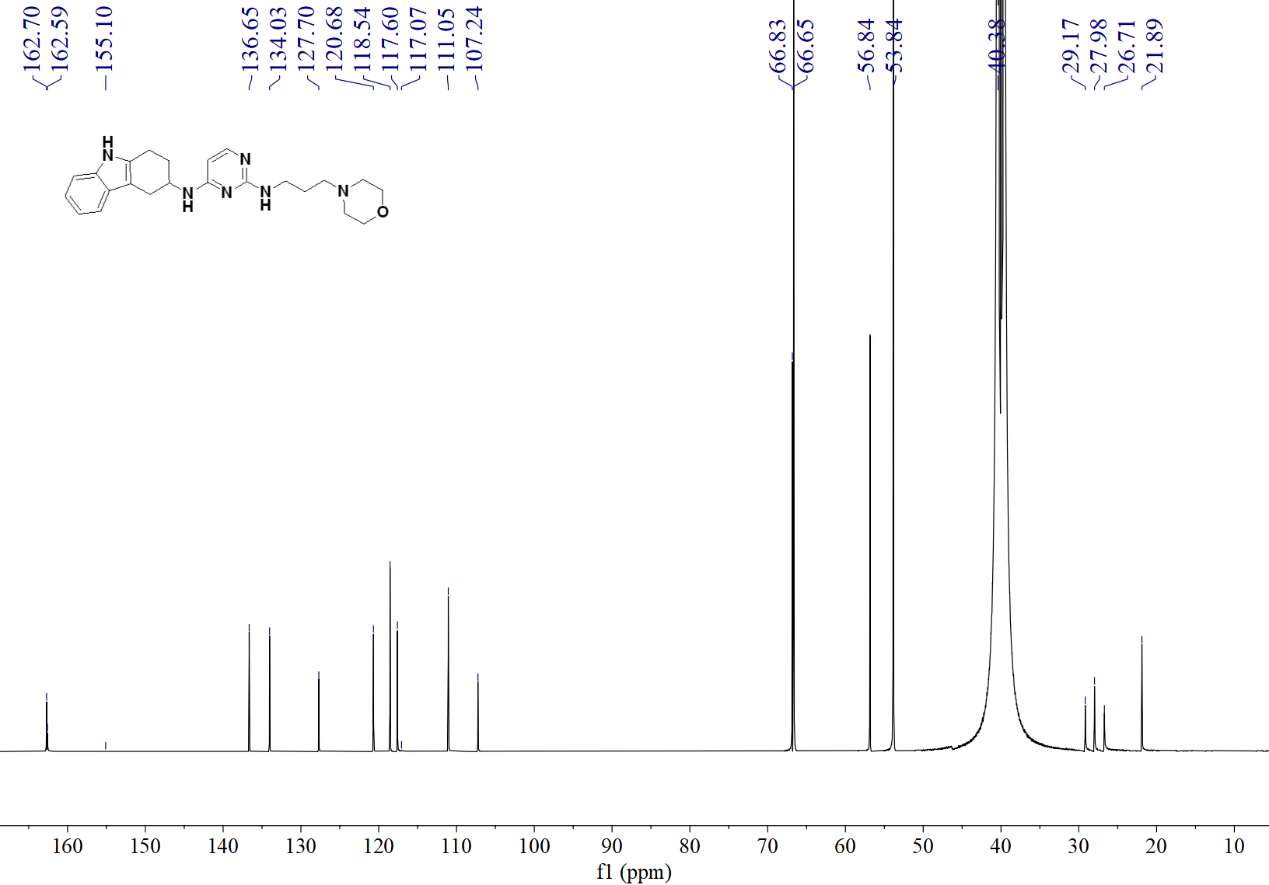


^13^C spectrum of compound **JX3214** (CDCl_3_)

^13^C spectrum of compound **JX3215** (CDCl_3_)

^13^C spectrum of compound **JX3216** (CDCl_3_)

^13^C spectrum of compound **JX3219** (CDCl_3_)

^13^C spectrum of compound **JX3221** (CDCl_3_)

^13^C spectrum of compound **JX3222** (CDCl_3_)

^13^C spectrum of compound **JX3223** (CDCl_3_)

^13^C spectrum of compound **JX3225** (CDCl_3_)

^13^C spectrum of compound **JX3230** (CDCl_3_)

^13^C spectrum of compound **JX3233** (CDCl_3_)

^13^C spectrum of compound **JX3235** (CDCl_3_)

^13^C spectrum of compound **JX3240** (CDCl_3_)

^13^C spectrum of compound **JX3244** (CDCl_3_)

^13^C spectrum of compound **JX3245** (CDCl_3_)

^13^C spectrum of compound **JX3246** (CDCl_3_)

^13^C spectrum of compound **JX3247** (CDCl_3_)

^13^C spectrum of compound **JX3249** (CDCl_3_)

**HRMS spectra**

EI spectrum of compound **JX3201**

EI spectrum of compound **JX3202**

EI spectrum of compound **JX3203**

ESI spectrum of compound **JX3204**

ESI spectrum of compound **JX3205**

ESI spectrum of compound **JX3206**

EI spectrum of compound **JX3207**

ESI spectrum of compound **JX3208**

EI spectrum of compound **JX3209**

EI spectrum of compound **JX3210**

ESI spectrum of compound **JX3211**

EI spectrum of compound **JX3212**

EI spectrum of compound **JX3213**

ESI spectrum of compound **JX3214**

ESI spectrum of compound **JX3215**

ESI spectrum of compound **JX3216**

ESI spectrum of compound **JX3217**

ESI spectrum of compound **JX3218**

ESI spectrum of compound **JX3219**

ESI spectrum of compound **JX3220**

ESI spectrum of compound **JX3221**

ESI spectrum of compound **JX3222**

ESI spectrum of compound **JX3223**

ESI spectrum of compound **JX3224**

ESI spectrum of compound **JX3225**

ESI spectrum of compound **JX3226**

ESI spectrum of compound **JX3227**

ESI spectrum of compound **JX3228**

ESI spectrum of compound **JX3229**

ESI spectrum of compound **JX3230**

ESI spectrum of compound **JX3231**

ESI spectrum of compound **JX3232**

ESI spectrum of compound **JX3233**

ESI spectrum of compound **JX3234**

ESI spectrum of compound **JX3235**

ESI spectrum of compound **JX3236**

ESI spectrum of compound **JX3237**

ESI spectrum of compound **JX3238**

ESI spectrum of compound **JX3239**

ESI spectrum of compound **JX3240**

ESI spectrum of compound **JX3241**

ESI spectrum of compound **JX3242**

ESI spectrum of compound **JX3243**

ESI spectrum of compound **JX3244**

ESI spectrum of compound **JX3245**

ESI spectrum of compound **JX3246**

ESI spectrum of compound **JX3247**

ESI spectrum of compound **JX3248**

ESI spectrum of compound **JX3249**

ESI spectrum of compound **JX3250**

**HPLC traces**

Compound **JX3201**

Compound **JX3202**

Compound **JX3203**

Compound **JX3204**

Compound **JX3205**

Compound **JX3206**

Compound **JX3207**

Compound **JX3208**

Compound **JX3209**

Compound **JX3210**

Compound **JX3211**

Compound **JX3212**

Compound **JX3213**

Compound **JX3214**

Compound **JX3215**

Compound **JX3216**

Compound **JX3217**

Compound **JX3218**

Compound **JX3219**

Compound **JX3220**

Compound **JX3221**

Compound **JX3222**

Compound **JX3223**

Compound **JX3224**

Compound **JX3225**

Compound **JX3226**

Compound **JX3227**

Compound **JX3228**

Compound **JX3229**

Compound **JX3230**

Compound **JX3231**

Compound **JX3232**

Compound **JX3233**

Compound **JX3234**

Compound **JX3235**

Compound **JX3236**

Compound **JX3237**

Compound **JX3238**

Compound **JX3239**

Compound **JX3240**

Compound **JX3241**

Compound **JX3242**

Compound **JX3243**

Compound **JX3244**

Compound **JX3245**

Compound **JX3246**

Compound **JX3247**

Compound **JX3248**

Compound **JX3249**

Compound **JX3250**
