# Supplementary figures and images for: Discovery of a Novel and Potent Kir4.1 Inhibitor as a Safe and Rapid‐Onset Antidepressant Agent in Mice
Source: Adv Sci (Weinh). 2025 Dec 3;13(9):e09506. doi: 10.1002/advs.202509506 (PMC12903969; doi:10.1002/advs.202509506)

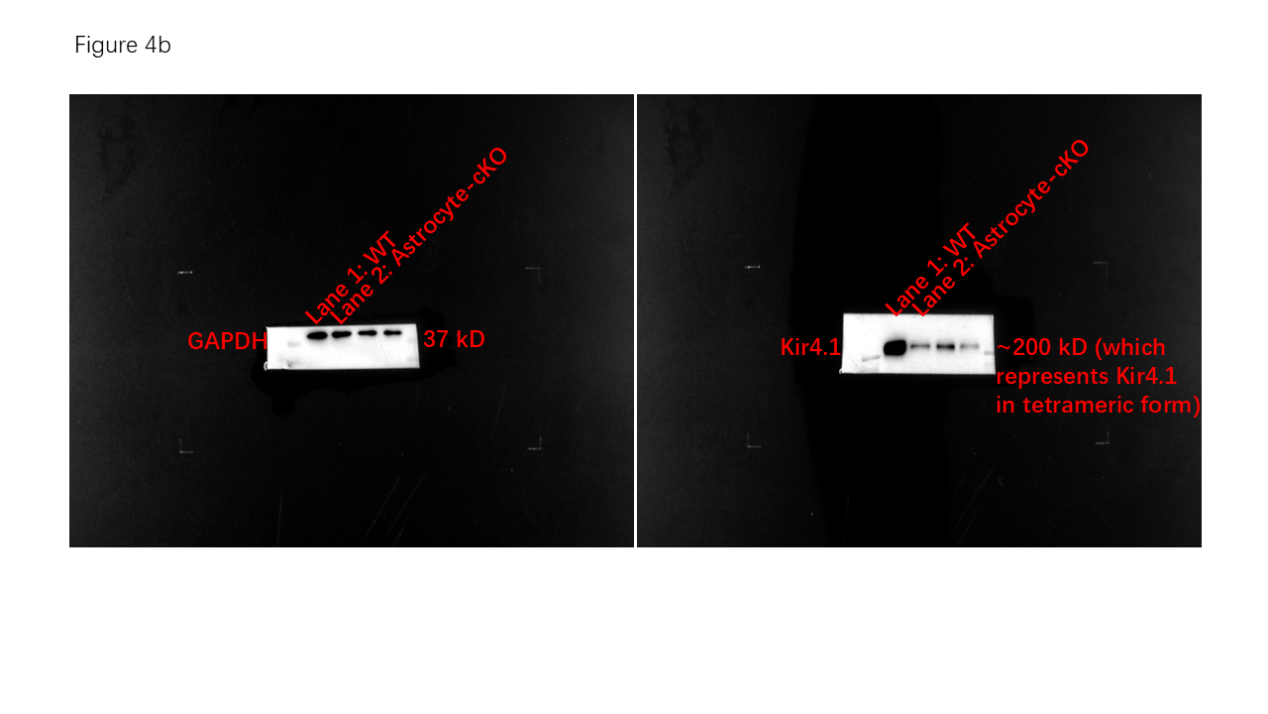


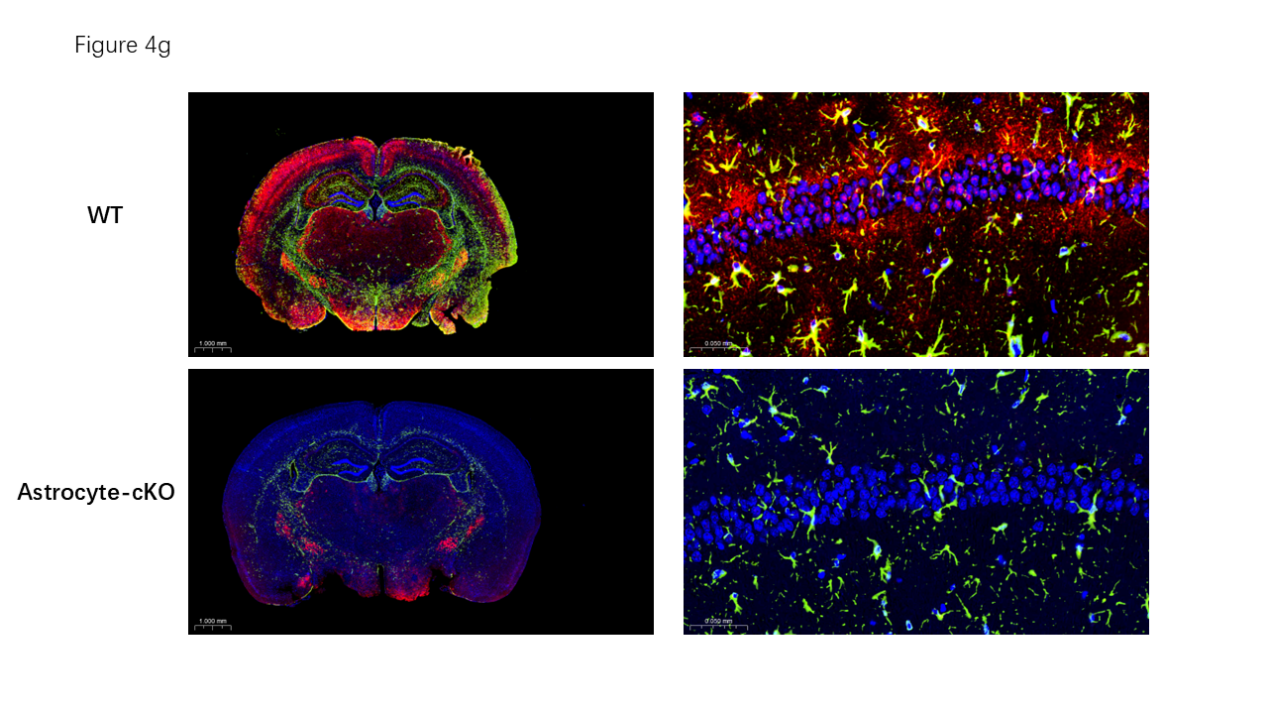


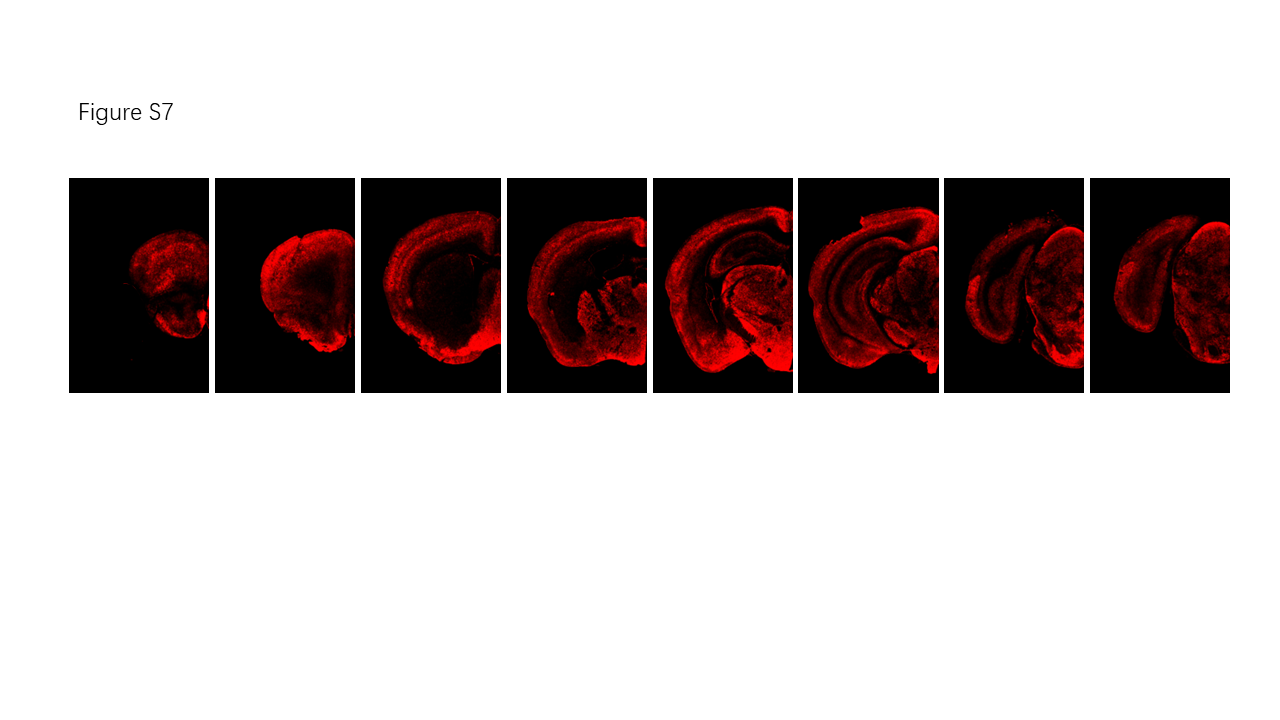

Supplement: Supplementary file 2 — Supporting Information [file ADVS-13-e09506-s001.zip › advs73154-sup-0001-Data/Source Data for wb experiments and fluorescence imaging.docx]
